# Targeting the SARS-CoV‑2 RNA Translation Initiation Element SL1 by Molecules of Low Molecular Weight

**DOI:** 10.1021/jacs.5c05264

**Published:** 2025-08-04

**Authors:** Sabrina Toews, Francesca Donà, Marco Keller, Jürgen Krauß, Franz Bracher, Úrsula López-García, Jörg Pabel, Daniel Merk, Marcel J. J. Blommers, Jan Ferner, Anna Wacker, Christian Richter, Harald Schwalbe

**Affiliations:** † Institute for Organic Chemistry and Chemical Biology, Center for Biomolecular Magnetic Resonance (BMRZ), Goethe University Frankfurt am Main, 60438 Frankfurt am Main, Hesse, Germany; ‡ Pharmaceutical Chemistry, Department of Pharmacy, Center for Drug Research, Ludwig-Maximilians-University Munich, 81377 Munich, Bavaria, Germany; § Department of Pharmacy, Ludwig-Maximilians-University Munich, 81377 Munich, Bavaria, Germany; ∥ Saverna Therapeutics, 4105 Biel-Benken, Switzerland

## Abstract

We present the development of low molecular weight inhibitors that target the 5′-terminal RNA stem-loop 1 (SL1) of the SARS-CoV-2 genome. SL1 is crucial for allowing viral protein synthesis in the context of global translational repression in infected cells. We applied compound- and RNA-detected nuclear magnetic resonance spectroscopy (NMR) experiments to guide a fragment-growth strategy based on two primary NMR screening hits from a diverse fragment library poised for follow-up chemistry. These primary hits with molecular weights of around 200 Da were derivatized with the aim of improving the initial solubility, binding affinity, and target specificity. We used NMR to monitor solubility changes, binding affinity, and specific binding to the SL1 binding pocket during the fragment derivatization campaign. Compounds scoring the best in all three categories were further tested for their inhibitory effect on SL1 in a cell-free translation assay, where the best two compounds, A.2 and A.13, showed both significant and selective inhibition. Our results demonstrate that small molecules targeting translation initiation of SARS-CoV-2 can be rapidly obtained using NMR-guided medicinal chemistry, and that the correlation between affinity, selectivity, and in situ function of the derived compounds is still to be explored.

## Graphical abstract

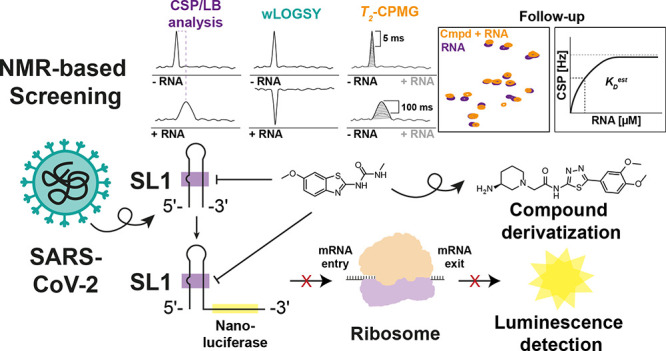

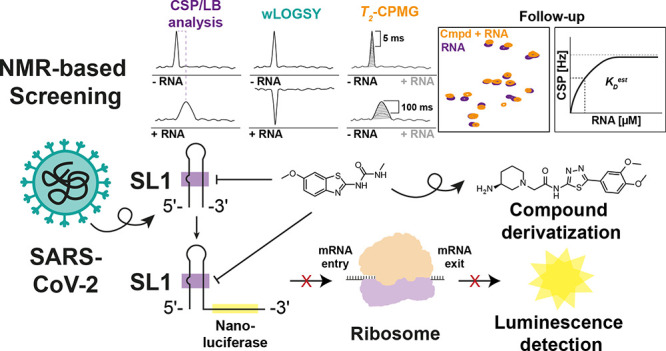

## Introduction

The pandemic outbreak of Coronavirus disease 2019 (COVID-19) highlighted the importance of developing antiviral therapeutics to combat the pandemic state.^1^ This urgent need led to numerous studies for the development of either vaccines as a prevention strategy or antiviral protein-targeting therapeutics in case of progressing infection.^2,3^

Targeting viral infections with low molecular weight compounds (small molecules) is of critical importance, and new antivirals are needed for pandemic preparedness. Most medicinal chemistry campaigns continue to focus on the viral proteome, but targeting the viral genome also holds great potential.^4–6^ Severe acute respiratory syndrome coronavirus 2 (SARS-CoV-2) contains a (+)-strand RNA genome, and after ∼10–24 h of infection, viral RNA dominates the total mRNA content of infected cells.^7^ The single-stranded viral RNA folds into modular stem-loop structures that can be determined rapidly and may serve as targets for small molecules.^8^ Many of these modular structures represent regulatory RNA elements that contain unique noncanonical structure motifs, including pseudoknots as well as single-stranded helix junctions and bulges with different degrees of residual structure. Consequently, the structural elements are attractive targets to interfere with the biological function of the virus.^9^ The targeted biological functions include virus replication or translation of viral proteins, or can directly promote degradation by recruiting nucleases.^10–12^ Small molecules can interact with a diverse set of RNA structural motifs, providing opportunities for combinatorial therapies to enhance antiviral effects.^13,14^ Work published by Disney and co-workers has pioneered innovative strategies using small molecules, including two-dimensional combinatorial screening (2DCS) and ribonuclease targeting chimeras (RiboTACs), to identify and selectively degrade disease-associated RNAs. Their work has advanced RNA-targeted drug discovery and provided a framework for the development of small molecules that modulate RNA function with high specificity.^4–6^ The general viability of targeting RNAs by small molecules was demonstrated by early proof-of-concept studies that demonstrated the antiviral activity of small molecules targeting, for example, the hepatitis C internal ribosome entry site (IRES), the human immunodeficiency virus (HIV) transactivating response region (TAR) RNA, the Influenza A promoter, and the SARS-CoV frameshift element.^15–19^ An overview covering more recent studies exploring small molecules to target viral RNAs is given in Mathez et al.^20^ An increasing amount of research has demonstrated the feasibility of targeting noncoding RNAs with small molecules using nuclear magnetic resonance spectroscopy (NMR)-guided fragment-based screening (FBS) and optimization. Varani and colleagues notably worked on this approach by identifying and structurally optimizing selective small-molecule binders for pre-miR-21, thereby advancing RNA-targeted ligand discovery and providing a framework for the structure-guided optimization of RNA-binding compounds.^21–23^ These advances highlight the potential of FBS for various RNA targets and encourage the application of these strategies to viral regulatory elements.

Recent studies highlight the SARS-CoV-2 stem-loop 1 (SL1) as a possible target to prevent viral propagation in vitro and in vivo.^24^ The current model describes SL1 as playing the key role in the preferential translation of viral mRNA over host RNAs, by circumventing the global inhibition of translation mediated by viral nonstructural protein 1 (Nsp1). Nsp1 has been shown to block the ribosomal mRNA entry channel via its C-terminal domain, thus interrupting host protein synthesis.^25,26^

For viral mRNA, SL1 mediates the release of Nsp1, allowing translation of viral proteins.^28,29^ Notably, it is still unclear whether and how the enhancement of viral translation by SL1 is linked to Nsp1 mechanistically. Thus, the inhibition of isolated SL1 is of great interest. So far, therapeutic approaches have primarily focused on antisense oligonucleotides (ASOs) targeting SL1 or, more generally, the 5′-untranslated region (UTR) leader sequence (SL1-SL3). Stable locked nucleic acid ASOs (LNA ASOs) as well as antisense peptide-conjugated morpholino oligomers (PPMOs) have demonstrated the potential to reduce viral load by binding to these 5′-UTR structured RNA elements, thus disrupting viral translation.^24,30^ As an alternative approach to ASO to inhibit the function of SL1, small molecules would offer advantages, including enhanced delivery, stability, and reduced costs. The viral RNA element SL1 consists of two A-form helical stems interrupted by an asymmetric 1:2 bulge and capped by a UCCC-tetraloop (Figure 1A). We determined its structure by NMR, documenting that the noncanonical asymmetric 1:2 bulge, whose structure can be modulated by pH, represents a binding site for small molecules.^27,31^

**Figure 1:**
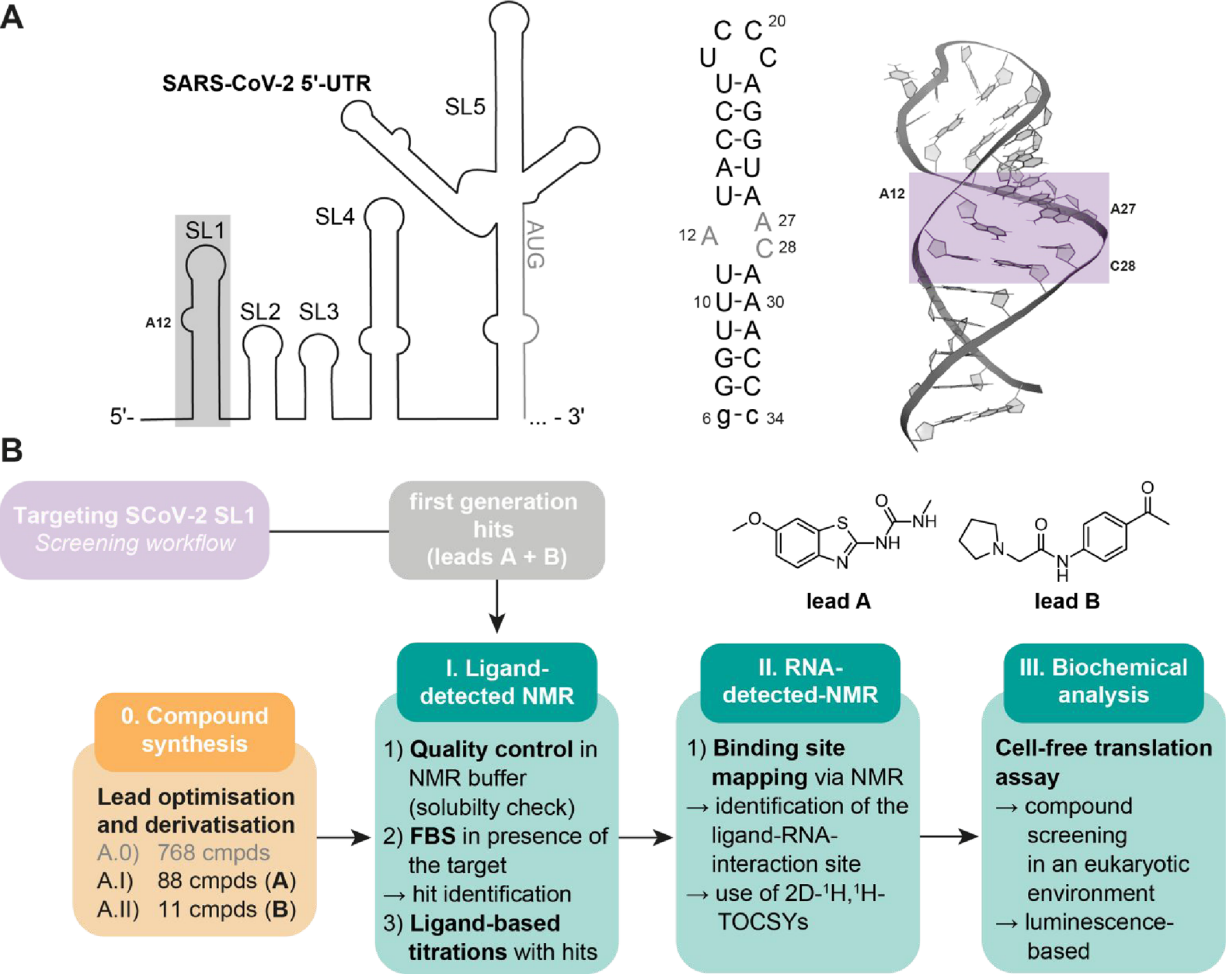
(A) Structure and genomic context of the SARS-CoV-2 SL1 target RNA (gray box). In the NMR-determined structure (PDB: 9EOW^27^), the SL1 structure contains an asymmetric 1:2 bulge as potential binding epitope highlighted in purple. (B) Workflow used to optimize binding affinity and selectivity of the lead compounds consists of four individual pillars: (0) synthesis of (novel) compounds (including the initial library for lead identification A.0, **A** derivatives (A.I), and **B** derivatives (A.II)); (I) ligand-detected NMR screening; (II) RNA-detected NMR for binding site identification; and (III) biochemical analysis via cell-translation reactions for further compound characterization and functional activity assessment.

In previous studies, we have analyzed the druggability of individual viral RNA elements of the SARS-CoV-2 RNA genome by small molecules.^32^ This NMR-based screening of a fragment library consisting of 768 compounds against 15 diverse structured SARS-CoV-2 RNA elements revealed 69 hits across the viral genome. To elucidate the binding characteristics and binding sites of several RNA elements, a series of follow-up experiments was conducted (Figure 1B). For SL1, this information allowed the prioritization of two compounds, **A** and **B**, from the initial five fragment hits (Figure 2).

**Figure 2:**
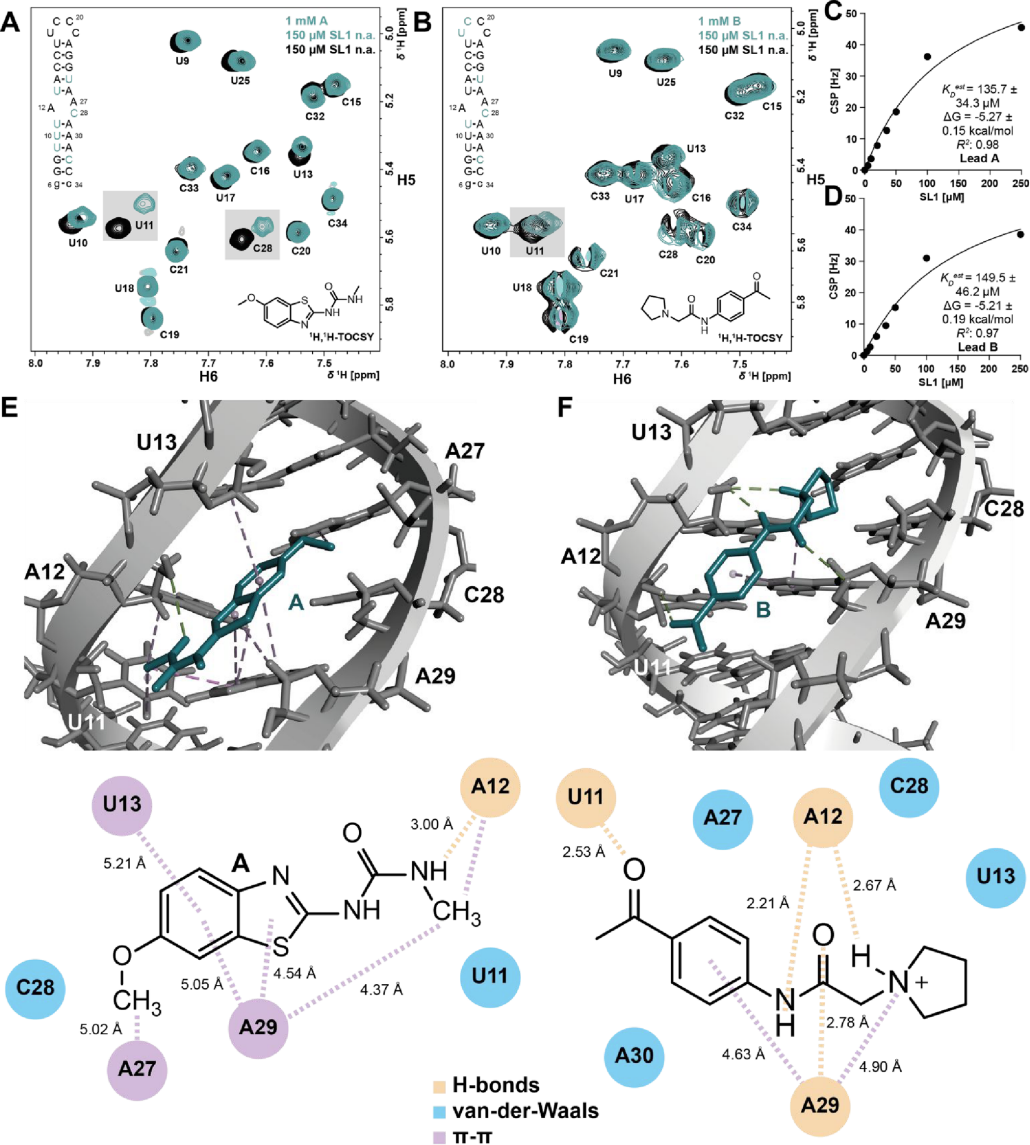
Characterization of the binding interaction between SL1 and initially prioritized hits **A** and **B**. (A,B) 2D-^1^H,^1^H-TOCSY overlay of SL1 in the absence and presence of **A** and **B**, respectively. Major shifts are highlighted in gray boxes. Measurements were performed with an unlabeled RNA (150 μM) sample and a sample containing both the compound (1 mM) and the unlabeled RNA (150 μM). Spectra with **A** were measured at 800 MHz and 298 K; spectra of **B** at 600 MHz and 298 K. (C,D) NMR-based ligand-detected determination of the binding affinities of the leads. The ligand concentration was kept constant at 100 μM as the RNA concentration increased from 0 to 250 μM in eight individual samples. 1D-^1^H spectra were recorded at 600 MHz and 298 K. (E,F) Molecular docking of **A** and **B** was performed with HADDOCK 2.5, from which the lowest energy state was selected for further analysis. H-bond (light orange), van-der-Waals (blue) and π-π (lilac) interactions are highlighted. The lowest energy docking poses highlight residues located within and adjacent to the bulge of SL1 as the binding site.

In our pursuit of improving SL1-targeting compounds **A** and **B**, we aimed to enhance solubility without compromising their biological activity.^33,34^ This process is critical, as solubility directly impacts a compound’s pharmacokinetic profile, bioavailability, and cellular uptake. Chemical modifications must be carefully designed to retain key molecular interactions with the target RNA while improving the solubility. A delicate balance is required to introduce hydrophilic functional groups that facilitate solvation while preserving the hydrophobic contacts involved in binding, which are well analyzed for proteins. During compound optimization, the hydrophilic–hydrophobic balance must be observed during chemical modification. Adjusting this balance can optimize its solubility without compromising its ability to penetrate cell membranes or interact with biological targets.^35,36^

Here, we report the development of a chemical tool compound derived from the initial SL1 screening hit **A** by optimizing the affinity, selectivity, and in situ activity by structure-guided chemistry (Figure 1). To monitor the improvement of affinity and selectivity by NMR, we used a total correlation spectroscopy (TOCSY) experiment, which offers two main advantages: The TOCSY relies on nonexchangeable proton signals, enabling detection of nonbase-paired motifs in the RNA that often serve as binding sites. Next, the TOCSY provides 2D resolution by correlating the H5 and H6 protons of pyrimidines, without the need for cost-intensive RNA labeling. Chemical modifications of **A** were further supported by structure–activity relationship (SAR) analysis guided by NMR.

To determine the impact of optimized compounds on translation initiation, we utilized an optimized cell-free translation-based assay to monitor the compound activity in situ. In this assay, the effect of SL1-targeting compounds on translation efficiency is measured via the activity of the translated reporter protein nanoluciferase. The assay reads out translation efficiency mediated by SL1 itself, independent of Nsp1. It relies on the intrinsic ability of the SARS-CoV-2 5′-UTR to promote robust viral translation, as demonstrated in previous studies showing that genomic RNA (gRNA) translation remains highly efficient and competitive despite the presence of extensive secondary structures.^37^

## Materials and Methods

### RNA Production

The RNA samples were prepared following the methodology previously published by Wacker, Weigand et al.^8^ For in vitro synthesis, previously amplified and linearized double-stranded template DNA was incubated at 37 °C for 6 h with recombinant RNA polymerase T7, along with cofactors and substrates (nucleoside triphosphates, Mg^2+^, dithiothreitol (DTT), spermidine) in a buffer containing 0.2 M Tris-HCl pH 8.0.^38,39^ The in vitro transcribed RNA was purified using denaturing polyacrylamide gel electrophoresis, followed by further purification with reverse-phase high-performance liquid chromatography (RP-HPLC). The final RNA samples were buffer-exchanged to 25 mM potassium phosphate (KP_i_), pH 6.2, and 50 mM potassium chloride (KCl) using 3-kDa molecular weight cutoff (MWCO) VivaSpin filtration units (Sartorius). RNA sequences are presented in Suppl. Table 1.

### Chemical Methods and Analytical Data

All chemicals were purchased from Thermo Fisher Scientific (Schwerte, Germany), TCI (Eschborn, Germany), Sigma-Aldrich (now Merck, Darmstadt, Germany), and Th. Geyer (Renningen, Germany), or ABCR (Karlsruhe, Germany), and were of analytical grade. Solvents, dry and of HPLC grade, were purchased from VWR (Darmstadt, Germany) or Sigma-Aldrich. All of the other solvents were purified by distillation. Thin layer chromatography (TLC) using polyester sheets POLYGRAM SIL G/UV 254, coated with 0.2 mm silica gel, from Macherey-Nagel (Düren, Germany) was used to monitor the progress of the conversion, and UV light (254 nm/365 nm) was used to visualize the compounds. Flash column chromatography (FCC) was performed using silica gel 60 (0.040–0.063 mm) from Macherey-Nagel. High-resolution mass spectra (HRMS) were recorded on a Thermo Finnigan MAT 95 or Joel MStation Sektorfeld instrument at a core temperature of 250 °C and 70 eV for EI or a Thermo Finnigan LTQ FT Ultra Fourier Transform Ion Cyclotron Resonance device at 250 °C for ESI. NMR spectra (^1^H,^13^C-DEPT, H–H–COSY, HMQC/HSQC, HMBC) were recorded at 23 °C on an Avance III HD 400 MHz Bruker BioSpin. All ^1^H NMR spectra and ^13^C NMR spectra were recorded respectively at 400 and 101 MHz, using deuterated chloroform (CDCl_3_), deuterated dimethyl sulfoxide (DMSO-*d*_6_), or deuterated methanol (CD_3_OD) as solvents, whose δ value of peaks was taken as an internal reference for chemical shifts. Chemical shifts δ are reported in parts per million (ppm) relative to tetramethylsilane. ^1^H NMR data are reported as follows: chemical shift δ (multiplicity, coupling constants J, integral). The following abbreviations were used for signal multiplicity: s (singlet), d (doublet), dd (double doublet), ddd (double double doublet), dt (double triplet), t (triplet), q (quartet), m (multiplet), and br (broad). The coupling constants J are reported in Hertz (Hz). ^13^C NMR data are reported as the chemical shift δ. NMR spectra were analyzed by the Software MestReNova 15.0.0–34764 (Mestrelab Research S.L.). Melting points (mp) were measured on Büchi melting point B-540 apparatus, reported in °C, and not corrected. Values for specific rotation [α]_D_^20^ were measured at a wavelength of λ = 589 nm (Na-D-line) at 20 °C using a PerkinElmer 241 Polarimeter instrument (layer thickness = 10 cm). All samples were dissolved in MeOH, and the concentration is stated in g/100 mL. HPLC analytical measurements for the determination of the purities of the final products were carried out with detection at 210 and 254 nm.

### NMR-Based Screening

#### Sample Preparation

Compound powders were dissolved in a solvent mixture consisting of 90% DMSO-*d*_6_ and 10% deuterium oxide (D_2_O) to a concentration of 25 or 50 mM for storage stocks and 5 mM for working stocks. These ligands needed quality control before measuring them in the presence of the target RNA. For this purpose, ^1^H spectra were recorded with 1 mM of the compounds in NMR screening buffer (25 mM KP_i_ pH 6.2 and 50 mM KCl), 5% DMSO-*d*_6_, and 10 μM sodium 3-(trimethylsilyl)­propane-1-sulfonate (DSS). Spectra were checked for impurities and the solubility of the compounds.

For each FBS experiment, samples of 40 μL (for 1.7 mm tubes) or 180 μL (for 3 mm tubes) were prepared by combining 10 μM unlabeled RNA, 10 μM DSS as a reference, and 200 μM various ligands. This mixture resulted in a final DMSO-*d*_6_ concentration of 5% (v/v) with an [RNA]:[ligand] ratio of 1:20. Control samples lacking RNA were also prepared by following the aforementioned conditions.

The preparation of samples for binding site mapping was conducted in a manner analogous to that employed for the FBS. RNA concentrations were increased to a range of 60 to 150 μM for use in RNA-detected NMR experiments. The buffer conditions remained unaltered except for increasing the DMSO-*d*_6_ concentration to 15% for measurements performed with lead **A**.

In order to conduct ligand-detected titrations, eight individual samples were prepared under similar conditions. In this series of samples, the ligand concentration was maintained at 100 μM, while the RNA concentration was increased in a range from 0 to 200 or 250 μM. The previously mentioned NMR buffer was also used for these samples.

#### 1D-NMR Experiments

The 1D-^1^H experiments were recorded using either excitation sculpting with gradients or jump-return echo water suppression pulse sequences.

Water-ligand interaction observed via gradient spectroscopy (wLOGSY) was employed for the identification of RNA-ligand interactions, based on the differential Nuclear Overhauser Effect (NOE) transfer between water and ligands in bound versus free states. In wLOGSY, bound and unbound ligands exhibit opposite phase (antiphase) signals due to differences in NOE transfer pathways. Ligands exhibiting inverted (positive) signals relative to the water peak are indicative of binding to the RNA target.^40^ For a mixture of RNA plus a compound, a positive signal indicates binding of the compound, whereas a negative signal indicates that the compound is unbound. Water signals were suppressed using the solvent-optimized double gradient spectroscopy (SOGGY) sequence.^41^ The wLOGSY factor was determined with eq 1, by defining the difference between the absolute peak intensities of the ligand in the presence (*I*_Target_) and absence (*I*_Reference_) of the RNA target, normalized to the reference intensity (*I*_Reference_).

$$\mathrm{wLOGSY}\;\mathrm{factor}=|\frac{\left(I_{\mathrm{Target}}-(I_{\mathrm{Reference}}\right)}{I_{\mathrm{Reference}}}|$$ 1

Carr–Purcell–Meiboom–Gill (CPMG) experiments were employed to determine the *T*_2_ relaxation time, utilizing a spin–echo pulse sequence.^42^ The measured *T*_2_ relaxation is significantly influenced by the rotational correlation time of the molecule under observation. In the presence of RNA, the *T*_2_-relaxation rates of a compound differ from those observed in the absence of RNA if binding occurs. The increase in *T*_2_ relaxation is attributed to the larger rotational correlation times of the compound in the complex with RNA, which results in signal broadening, in contrast to the sharper signals observed for the free ligand. Water signals were suppressed using the SOGGY sequence.^41^ Quantitative analysis of *T*_2_ relaxation was based on the reduction in relative peak integrals of proton signals measured at 100 and 5 ms CPMG delays. The *T*_2_ reduction, expressed as a percentage, reflects the signal loss observed in the presence versus absence of the RNA target and was calculated using eq 2.

$$T_{2}\;\mathrm{reduction}=\left[1-\frac{\frac{I_{\mathrm{Target}}\;(100\;\mathrm{ms})}{I_{\mathrm{Reference}}\;(100\;\mathrm{ms})}}{\frac{I_{\mathrm{Target}}\;(5\;\mathrm{ms})}{I_{\mathrm{Reference}}\;(5\;\mathrm{ms})}}\right]\times 100$$ 2

#### 2D-NMR Experiments

TOCSY experiments were employed to observe cross peaks between pyrimidine H5 and H6 protons via homonuclear Hartmann–Hahn transfer using the DIPSI2 sequence.^43,44^ Water suppression was achieved by excitation sculpting in the direct dimension. The pulse sequence employed in this experiment was “dipsi2esfbgpph” from the Bruker library, with a TOCSY mixing time of 30 ms. The chemical shift perturbations (CSPs) of RNA H5–H6 cross peaks upon compound addition were determined with eq 3. In this equation, Δδ_H_ represents either the chemical shift of the H5 or the H6 resonance.

$$\mathrm{CSP}\left(\Delta \delta_{\mathrm{HH}}\right)=\sqrt{{\left(\Delta \delta_{H5}\right)}^{2}+{\left(\Delta \delta_{H6}\right)}^{2}}$$ 3

#### NMR-Based *K*_D_ Estimation

The binding affinities of the compounds were assessed via RNA-dependent CSPs observed in compound 1D NMR spectra, as previously described by Sreeramulu, Richter et al.^32^ 1D-^1^H NMR experiments were conducted at 298 K and 600 MHz, employing excitation sculpting for solvent suppression. CSPs of well-resolved compound signals were plotted against RNA concentration and fitted with eq 4, assuming a single site-specific binding as the first approximation (eq 4). In this equation, *X* represents the RNA concentrations in μM, while *B*_max_ denotes the maximum CSP. The estimated dissociation constant (*K*_D_^est^) is regarded as a semiquantitative measure, given that in the concentration regime used for NMR, CSPs often did not reach saturation and nonspecific binding events could not be ruled out.

$$\mathrm{CSP}\;[\mathrm{Hz}]=\frac{B_{\max}X}{K_{D}^{\mathrm{est}}+X}$$ 4

### Docking via HADDOCK

Docking was performed using HADDOCK 2.5 to predict the binding mode of the ligand to the RNA construct.^45,46^ The RNA solution structure with the PDB entry 9EOW was used as the input file.^27^ Based on binding site mapping by NMR, residues U11, A12, U13, C28, and A29 were defined as active residues for the docking calculations. Docking was carried out using the default settings of HADDOCK, including rigid-body docking, semiflexible refinement, and explicit solvent refinement. The resulting docking poses were analyzed based on the HADDOCK score, which integrates van der Waals, electrostatics, and desolvation energy terms, as well as visual inspection of the predicted binding modes. Molecular interactions and binding conformations were visualized using the BIOVIA Discovery Studio Visualizer.

### Similarity Analysis

Compound similarity was assessed with the FragFP fingerprint and Tanimoto coefficient using DataWarrior (version 6.1.0).^47^ A neighborhood similarity threshold of 86% was used to identify structurally related compounds. Similarity charts were used to visualize the relationships. Default settings were used unless otherwise stated.

### Cell-Free Assay for Ligand-Target Interaction Studies

As hits were identified and prioritized, compounds were further tested to determine their capability of interrupting the translation of viral RNA elements in an in vitro cell-free translation assay. The assay setup itself was designed based on experiments published by Tidu et al. and Bäumlin et al.^28,48^ Briefly, DNA templates coding either for different elements of the SARS-CoV-2 5′-UTR or a control 5′-UTR, Nanoluciferase, and a poly-A tail inserted into a pUC19 vector were designed and purchased from Genscript. All RNA sequences are summarized in Suppl. Table 1 and complete vector sequences are available on request. mRNAs were produced via in vitro transcription, as described before. The protocol for post-transcriptional mRNA purification was adapted from Sharma et al.^49^ After in vitro transcription, the reaction volume was adjusted to 50 μL with water. Subsequently, 25 μL of 7.5 M LiCl was added, and the mixture was thoroughly mixed before incubation at −20 °C for at least 1 h. The RNA was pelleted by centrifugation at 16,000×*g* for over 20 min in a precooled tabletop centrifuge (4 °C). The pellet was washed twice with ice-cold 70% ethanol and allowed to air-dry completely with the tube cap open. Finally, the dried RNA pellet was resuspended in water for downstream applications.

Post-transcriptional 5′-end-capping was performed to enhance translation and protect against exonuclease-induced degradation. Capping was achieved using the Vaccina capping enzyme in combination with 2′-*O*-Methyltransferase (both New England BioLabs) for comethylation and the final generation of a 5′-cap1 structure. Finally, modified mRNA constructs were purified using the Monarch RNA Cleanup Kit (New England BioLabs) and eluted in water.

The cell-free translation assay was performed in rabbit reticulocyte lysate (RRL). The crude lysate was purchased from Green Hectares (USA) and first subjected to nuclease treatment to eliminate globin mRNA. Hemin was supplemented to prevent eIF2α phosphorylation by heme-dependent kinases, which can inhibit multiple rounds of translation. To achieve effective translation, a final mRNA concentration of 0.2 μM was used. For a typical test reaction size of 20 μL, all chemical solutions were thawed at room temperature (RT) and subsequently maintained on ice. The in-house prepared cell extract (57% (v/v)) was supplemented with 0.2 μM purified mRNA, 1.2 μM MgCl_2_, 0.5 μM DTT, 1.2 μM spermidine, and 0.8 U/μL RNase inhibitor (RNasin, Promega). For ligand activity screening, the compound was added with a final concentration of 2 μM, equal to a 1:10 [RNA]:[ligand] ratio. Reaction mixtures were incubated at 30 °C for 60 min. Subsequently, samples of 15 μL total volume were transferred to 384-well round-bottom white polystyrene microplates (Corning) and supplemented with an equal volume of the substrate Nano-Glo (furimazine) according to the manufacturer’s protocol (Promega). Luminescence (475–500 nm) was detected at 30 °C (Tecan, Männedorf, Switzerland), including technical replicate measurements of each well. Measurements were repeated three times, normalized to the specific controls, and reported with the respective standard deviation.

## Results and Discussion

### Diverse Synthesis of RNA Binding Small Molecules Combining Insights from Docking and NMR

Based on binding affinity and binding site determination of our initial screen of 15 structured elements of SARS-CoV-2, we prioritized two compounds, **A** and **B**, that target the asymmetric 1:2 bulge of SL1 with sufficient selectivity. To detect binding, in general, RNA detected 1D-^1^H and 2D-^1^H,^1^H-TOCSY experiments of SL1 in the absence and presence of these lead compounds were measured. These experiments provided evidence of CSPs and line broadening effects (LB) relative to blank samples containing the same amount of DMSO-*d*_6_, indicating direct binding to the bulge of SL1 (Figure 2A, B, Suppl. Figure 1). Due to solubility issues, **A** required a total DMSO-*d*_6_ concentration of 15% for measurements, whereas experiments performed with **B** were measured in the presence of 5% DMSO-*d*_6_. Interestingly, the control sample containing only compound **B** at a concentration of 1 mM showed precipitation, whereas the sample containing both SL1 and **B** at the same concentration showed no precipitation, suggesting RNA-mediated solubility.^50^ For both compounds, a novel imino proton signal at around 12.6–12.7 ppm (Suppl. Figure 1) was detected in 1D NMR experiments. Additionally, 1D-NMR analysis revealed several shifts of the target’s aromatic signals, such as the H8 and H2 resonances of residues A29 and A12, respectively (Suppl. Figure 1). The TOCSY experiments revealed chemical shift perturbations (CSPs) for resonances of residues within or adjacent to the bulge, particularly for lead **A** (Figure 2A).

For **A**, notable shifts were observed for U10, U11, U13, and C28, with smaller shifts detected for U9 and U25. For **B**, CSPs were generally weaker with the most pronounced shifts occurring at U11 and C28. Among these, U11 emerged as the most promising reporter residue in the TOCSY experiments for both leads **A** and **B** (Figure 2A, B). This signal originates from a residue adjacent to the loop rather than within it. Therefore, the signal is considered to be a secondary reporter, with respect to the actual binding site, representing a possible structural rearrangement induced by ligand binding. Follow-up NMR analysis also included the estimation of binding affinities by ligand-detected 1D-^1^H spectra at different [RNA]:[ligand] ratios. Here, binding affinities *K*_D_^est^ of 136 ± 34 and 150 ± 46 μM were determined for **A** and **B**, respectively (Figure 2C,D). The results obtained from NMR measurements were compared to docking calculations to provide structural proposals for the binding interactions of the lead compounds and their derivatives with SARS-CoV-2 RNA SL1. We applied computational docking for predicting the molecular recognition of compounds **A** and **B** by SL1. In our previous publication, we demonstrated that the structure of the bulge is sensitive to pH and adopts different conformations, which are not accounted for in the docking calculations.^27^ All studies were performed at a pH of 6.2. Based on previous studies, at pH 6.2, the SL1 RNA exists as a dynamic ensemble of two significant conformations in a ∼3:7 ratio. This equilibrium is due to the differential protonation of adenosine at position 12 (A12), which has a p*K*_a_ of 5.8. At pH 6.2, A12 is transiently protonated, leading to conformational heterogeneity. The conformational heterogeneity at pH 6.2 was particularly relevant to our study, as it may influence ligand binding and contribute to the observed CSPs, highlighting the importance of protonation of A12 in the interaction dynamics. NMR results revealed CSPs for bulge residues, guiding the selection of U11, A12, U13, C28, and A29 as active residues for docking calculations, which were performed with lead structures **A** and **B** using the lowest-energy poses based on the SL1 solution structure (PDB: 9EOW).^27^ Docking calculations were evaluated based on their HADDOCK score. This score is based on a weighted combination of van-der-Waals, electrostatics, and desolvation energies. For **A**, the lowest-energy cluster comprised 30 docking poses (total: 162 structures in 15 clusters), with its lowest-energy structure achieving a score of −22.8 ± 0.4 and a root-mean-square deviation (RMSD) of 1.7 ± 0.0 Å. For **B**, the lowest-energy cluster comprised 22 docking poses (total: 179 structures in 9 clusters), with its lowest-energy structure achieving a score of −26.0 ± 0.5 and a RMSD of 2.5 ± 0.1 Å (Figure 2E,F).

For **A**, a hydrogen bond (H-bond) was observed between the O2’ of SL1 A12 (H-acceptor) and the amide group of **A** (H-donor) with a length of 3.00 Å (Figure 2E). Docking analysis of lead **B** predicted fewer interactions but additional H-bonds in comparison to calculations performed with lead **A**. For example, H-bonds were observed between the HO2’ of SL1 U11 (H-donor) and the carbonyl O of the methoxy group of **B** with a length of 2.53 Å, as well as between the O2’ of SL1 A12 (H-acceptor) and -NH of the linker of **B** (H-donor) at 2.21 Å (Figure 2F). A complete set of the calculated contacts is presented in Suppl. Table 2 including additional H-bonds, π-, and van-der-Waals interactions. It should be noted, however, that the docking calculations were not considered to represent an experimental structure. Calculated interactions support the previously detected interaction between the bulge of SL1 and the lead compounds **A** and **B**.

### NMR Monitoring of Follow-Up Chemical Series

The derivatization series of lead **A** was assessed by an NMR-based quality control (QC) for solubility and purity. Only compounds that passed this control were further investigated for potential binding to SL1. Compounds not passing the QC are further designated with the index x in their nomenclature. Fragment-based screenings (FBS) were performed with compounds that previously passed the 1D-^1^H NMR-based QC. FBS included the following set of NMR experiments: (I) 1D-^1^H; (II) wLOGSY; and (III) *T*_2_-relaxation experiments (Figure 3). The 1D-^1^H NMR measurements were used to examine binding-induced CSPs as well as LB. We applied the same approach as published by Sreeramulu, Richter et al.^32^ These initial tests were performed with a ligand-to-RNA ratio of 20:1, for which a concentration of 10 μM of unlabeled RNA is sufficient.

**Figure 3:**
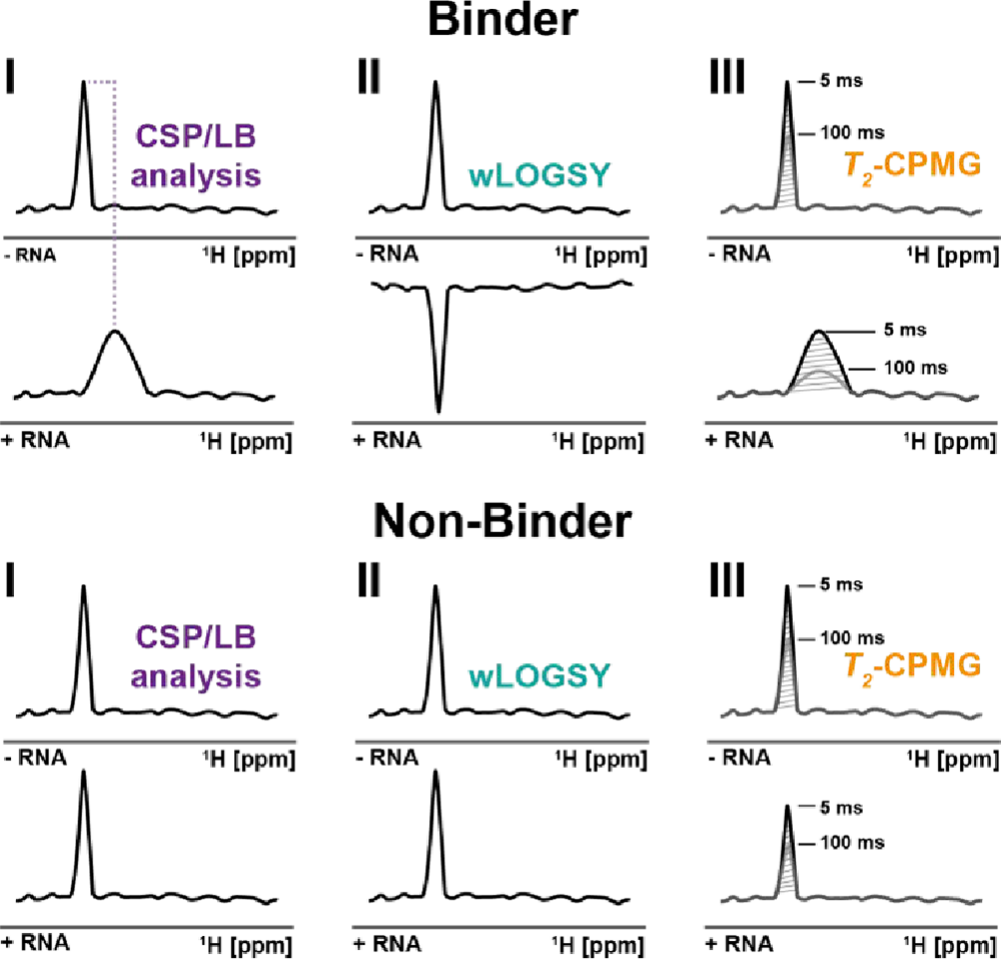
Ligand-based experiments were conducted in the presence and absence of the target RNA. (D) Selected NMR experiments for fragment-based screening: (I) 1D-^1^H NMR for CSP and LB analysis; (II) wLOGSY experiments measuring the transfer of magnetization from water to the ligand; and (III) CMPG-based *T*_2_-relaxation experiments (*T*_2_ = 5 or 100 ms) observed on ligand signals taking advantage of the increasing size of the RNA-ligand complex in case of a binding effect. Spectra for interacting and noninteracting compounds are illustrated for the selected experiments. The experiments work in the case of fast chemical exchange of the free and bound ligand.

### Failed Compound Hit-to-Lead Attempts of Initial Hit Compound **B**

Besides **A**, we engaged on compound **B** as the lead compound for structural optimization, as positive effects in terms of binding interaction were detected with this compound (Supplemental Information, Suppl. Figure 2). In contrast to the effective derivatization of the lead compound **A** (see below), modifications of **B** resulted in a complete loss of binding interactions between SL1 and these compounds. 1D-NMR FBS did not demonstrate any interaction; for some compounds, 2D-NMR experiments were performed, which also did not reveal any effects (data not shown in this manuscript). Thus, from two initially prioritized compound hits, only one compound could be successfully derivatized to increase the number of binding compounds to SL1.

#### NMR-based screening results obtained with lead **A**

**The positive impact of modifications–the derivatization of lead compound A.** in the presence of SL1 were used as a benchmark for the derivatized compounds. Derivatized compounds were therefore classified as hits if the following criteria were met: (I) CSPs of at least 3 Hz, (II) a *T*_2_ reduction of at least 30%, and (III) a wLOGSY factor of at least 1 (Figure 3).

These criteria are based on FBS results obtained with lead **A**. If feasible, an analysis was conducted on multiple ligand resonances. The results of this analysis were then averaged to produce an overall score for an overall hit ranking. The results of the three individual FBS NMR experiments were ranked within the overall data series of all measured compounds and finally summed for each compound. This sum was used for a final ranking within the same data series and hereafter referred to as the score (Table 1).

**Table 1:**
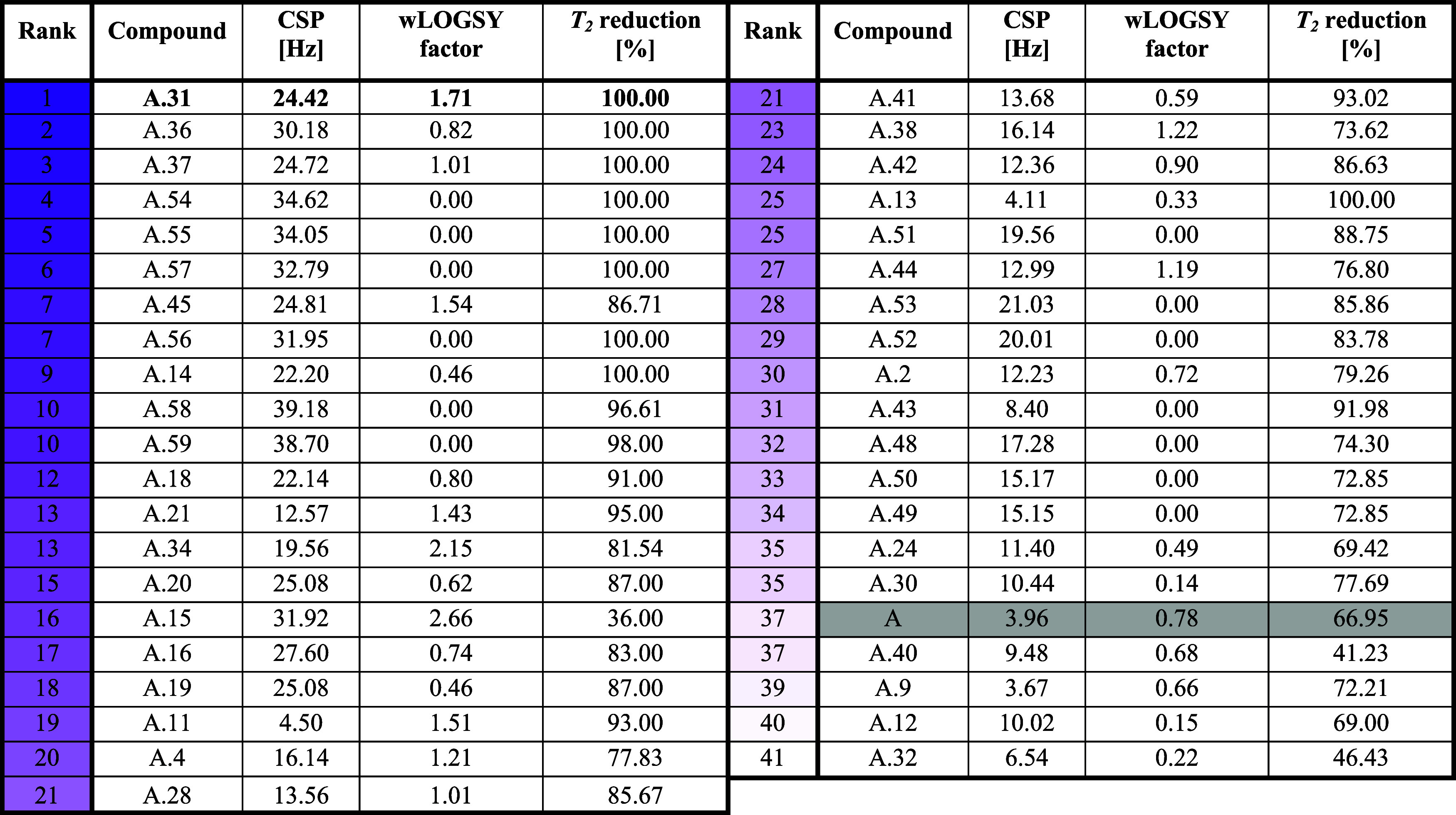
Ranked FBS Results of **A** and Its Derivatives Are Classified As Hits Measured in the Presence of the Viral Target RNA SL1^a^

a The compounds were ranked on their results derived from three individual results: (I) 1D-^**1**^H NMR; (II) wLOGSY experiments; and (III) CMPG-based *T*_2_-relaxation experiments (*T*_2_ = 5 or 100 ms). The higher the score, the stronger the effects found in these experiments. Results obtained with lead **A** are highlighted in gray.

The initially prioritized hit **A** is composed of a benzothiazole scaffold with the functional group *N*-methylurea as a modified amino group (Figure 4). Systematic modifications of **A** were carried out to increase solubility and affinity toward SL1. The modifications can be categorized into four distinct classes, detailed in the following section: (I) the modification of the 2-amino group, including guanidine; urea and amide derivatives; (II) the general substitution of the benzothiazole scaffold by other aromatic rings; (III) the addition of variable substituents to the aromatic scaffold, and (IV) the substitution of the benzothiazole scaffold by a 2-arylthiadiazole (Figure 4). Detailed synthesis procedures are given in the Supporting Information. Information regarding chemical structures of the synthesized derivatives is presented in the Suppl. Tables 3 and 4 as well as in the Suppl. Figures 3 and 4.

**Figure 4:**
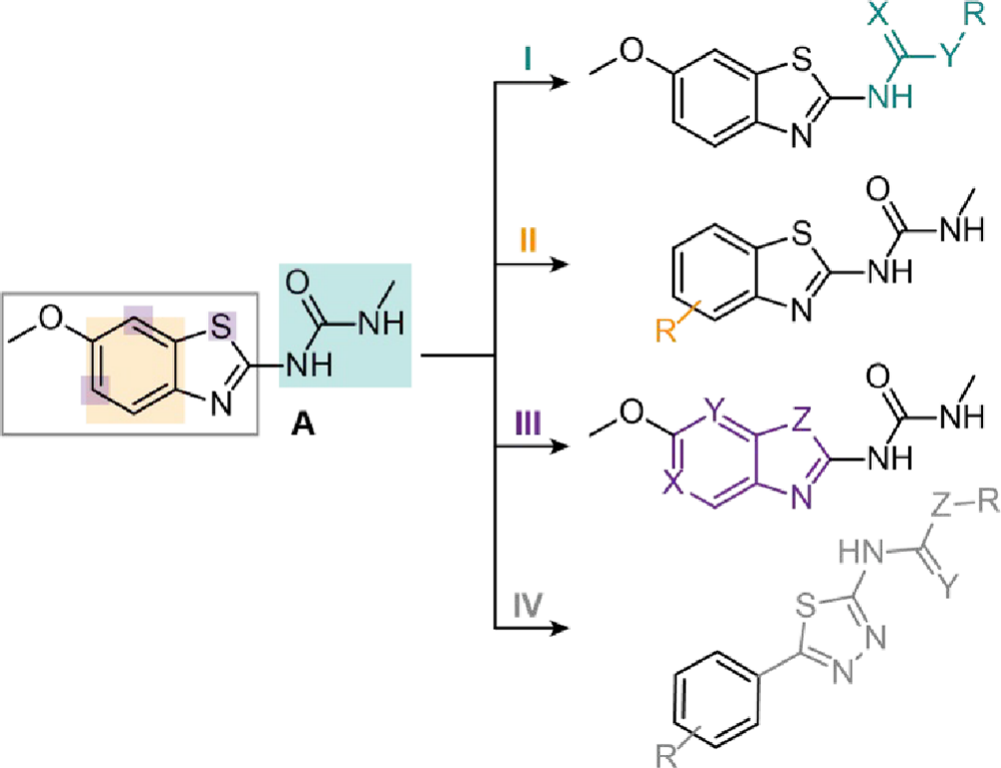
Structural modifications of lead compound **A**: (I) modification of the 2-amino group; (II) addition of variable substituents to the aromatic scaffold; (III) substitution of the benzothiazole scaffold with other aromatic systems; and (IV) substitution of the benzothiazole scaffold by a 2-arylthiadiazole.

#### Modification of the 2-Amino Group and Subsequent Effects on Affinity

Modifications of the 2-amino group in **A** led to a diverse set of compounds shown in Suppl. Figures 3 and 4. An FBS-based analysis for most of the amide compounds, including A.6, A.7, and A.8, revealed only minor effects. The results obtained for the urea-like moieties **A**, A.9, A.11, and A.13 were stronger, with a higher *T_2_* reduction for the 4,6-substituted A.11 and A.13 (Table 1). The strongest FBS effects were observed with guanidine A.4, classifying it as the most effective compound in this modification series. The addition of the N,N-dimethylglycinamide, which is present in compound A.2, exhibited encouraging results, and A.2 was among the most effective compounds in terms of FBS scoring. Additionally, A.2 was found to have an increased solubility compared to other amides, x A.8, A.6, A.7, and A.8, attributable to the incorporation of the aliphatic amine moiety. Thus, A.2 was taken as the starting point for synthesis route (III).

#### Substitution of Benzothiazole by a Benzimidazole Scaffold Results in a Higher FBS SL1 Targeting Score

In a second series of compounds, we focused on modifications within the aromatic ring system of the benzothiazole. This approach included compounds with modifications present in the bicyclic heteroaryl moiety at position 1, 5, and 7′ (Suppl. Figures 3 and 4) and led to new classes of compounds including benzoxazoles (A.35), pyridothiazoles (A.38), pyrimidothiazoles (A.46, A.47) and benzimidazoles (A.20). However, A.35, A.38, A.46, and A.47 did not show any improvement in terms of FBS scoring in comparison to A.2. An exception was A.20, which showed a higher score than that of A.2.

#### Addition of Variable Substituents to the Aromatic Scaffold Retained Original Binding Properties

In a third derivatization route, the 6-methoxy group (6-OCH_3_) was replaced by various modifications while maintaining the *N*,*N*-dimethylglycinamide lateral chain (Suppl. Figures 3 and 4). NMR analysis of the novel compound set, using A.2 as a reference, confirmed binding for all 2-aminobenzothiazoles, indicating that diverse substituents retained activity and validating the role of the *N*,*N*-dimethylamino moiety. A.14 (6-nitro), A.16 (6-bromo), and A.15 (4,6-dimethoxy) showed CSP values 2–3 times higher than A.2. However, A.14, containing a nitro group instead of a methoxy group, was excluded due to poor solubility. Overall, these modifications revealed that various adjustments at the six-membered ring are possible without loss of SL1 binding.

Incorporation of an additional basic site on the lateral chain, e.g., in compounds A.18, A.36, A.37, and A.45, exhibited a positive effect on SL1 binding. Among the various modifications, the incorporation of piperazine or *N-*methylpiperazine residues enhanced affinity, with the greatest effect observed for 3-aminopiperidine, particularly the *S*-isomer. Conversely, bulky substituents, as exemplified by A.33, were detrimental to binding to SL1. To be emphasized, A.33 is less basic than A.18, A.36, A.37, and A.45 (pyrimidine moiety vs aliphatic amines), which may reduce its ability to form electrostatic interactions with RNA and, therefore, reduce its affinity. Further modifications included *N*-methylation of the amide (A.34), which resulted in slightly higher affinity, but involved a more complex synthetic pathway, and a fusion of a piperazine moiety with A.20 (benzimidazole), which resulted in A.40 with reduced activity and was subsequently abandoned (Table 1). A.12 (OH) and A.24 (6-NH_2_,), both hydrogen donors, were among the weakest binders compared to A.2. However, this trend was not always consistent. For instance, A.13 exhibited different binding behavior than its 4,6-dimethoxy substituted analogue A.11, possibly due to differences in interaction types such as urea-based interactions rather than the tertiary basic amine interactions seen in A.2. A.13 has more hydrogen bond donors than does A.11, which could contribute to its distinct binding profile. Comparing A.13 to other compounds, such as A.18, A.36, A.37, and A.45, further differences in binding behavior can be observed. However, the potential for hydrogen bonding between the urea group and RNA remains speculative, as this interaction has not been directly observed. Similarly, while the tertiary basic amine in A.2 may interact with RNA, this is inferred from the structural context and not directly observed. A similar pattern was observed for A.9 compared to the lead **A**, suggesting that 6-OH interactions can be exploited for binding. This could also explain why A.12 and A.24 do not follow the same trend, relative to A.2. The stronger interaction of the *N,N*-dimethylamine in A.2 may prevent effective utilization of 6-OH or NH_2_ for binding, possibly due to insufficient proximity for optimal interaction.

#### Substitution of the Benzothiazole Scaffold by 2-Arylthiadiazole As a Combinatorial Derivatization Approach

A fourth strategy of follow-up synthesis focused on combinatorial derivatization, incorporating the previously discussed beneficial modifications. While some compounds (A.21, A.22, A.23, etc.) already featured these modifications, the approach was further expanded to yield A.58 and A.59; Suppl. Figures 3 and 4. Substitution of benzothiazole by the 2-arylthiadiazole moiety led to an almost 4-fold higher scoring in the FBS ranking when comparing A.58 and A.59 with lead **A**.

Compound A.21 was placed in a similar range. However, compounds A.22 and A.23, which also contain the 2-arylthiadiazole moiety but lack the 2-amino group, resulted in a no hit classification by FBS and, hence, are not present in the FBS ranking. This finding reinforces the beneficial effects of the 2-amino group, which also serves as a linker in compounds A.58 and A.59.

### Structural Similarity Clustering Including Compounds of the Derivatization Series of Lead **A**

A structure similarity analysis of all compounds belonging to the **A** derivatization series was performed using DataWarrior, applying both Tanimoto and FragFP metrics to quantify structural similarities across the data set.^47^ For this study, we used the complete set of ligands that successfully passed the QC process regardless of the presence of positive FBS results. The compounds were then clustered based on these similarity scores, which allowed us to group them into distinct structural families (Figure 5A). Similarity scoring was determined based on the structure of **A**. The compounds most similar to lead **A** are A.4, A.9, A.11, and A.13 (Figure 5B). Most of these compounds were already prominent in the NMR-based experiments, with A.4 showing global shift changes in the detected RNA H5–H6 correlations, and A.11 and A.13 showing similar CSPs in these experiments (Figure 6, Suppl. Figure 5); their FBS scores reflect moderate to low effects. In contrast, the high-scoring derivatives A.31 and A.36 belong to clusters that are very different from the previous cluster, with a similarity score of 0.25–0.5 (Figure 5A). A.31 is part of a cluster based on the shared thiadiazole moiety, whereas A.36 is clustered based on modifications of the 2-amino group while maintaining the overall lead scaffold. Furthermore, the compounds in ranking positions 3–5 (A.37, A.54, A.55) of the FBS experiments are all part of the A.36 cluster (Figure 5A), highlighting the modification of the 2-amino group used here as a linker between the benzothiazole scaffold and the additional aminopiperidine as a promising improvement.

**Figure 5:**
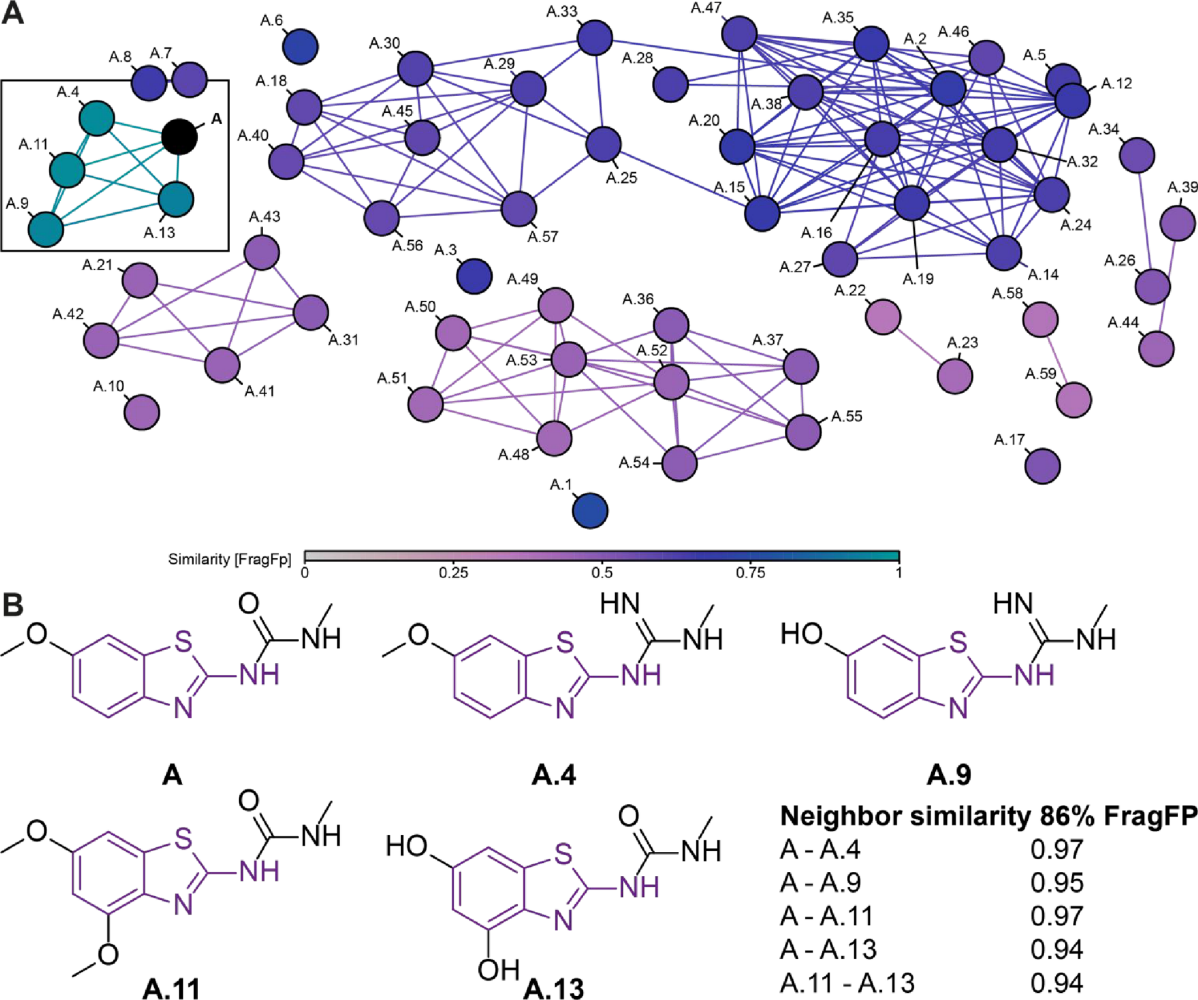
Cluster analysis of derivatives based on lead compound **A** using DataWarrior. (A) Molecular similarity of the derivatives was calculated using the FragFP fingerprint method and visualized as a clustered network, where points represent individual compounds connected by lines indicating similarity. The lead structure **A** is highlighted in black, and the color scale represents the degree of similarity, ranging from light gray (low similarity) to turquoise (high similarity). (B) Chemical structures of the compounds clustered next to lead compound **A** highlighting the shared scaffold in purple.

**Figure 6:**
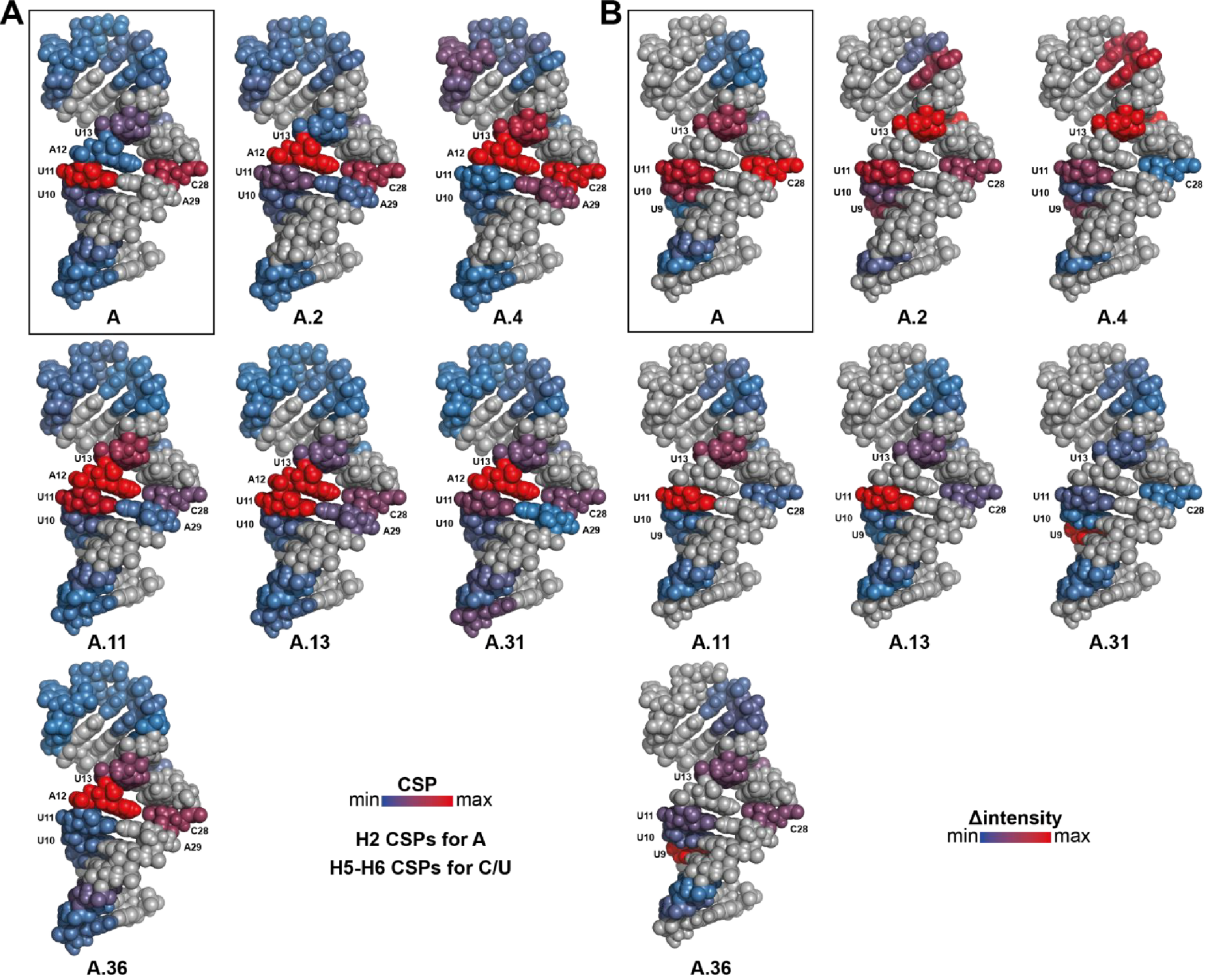
Shifts and line broadening of SL1 resonances upon addition of ligand addition. (A) Ligand-induced pyrimidine (H5–H6) and adenosine (H2) CSPs mapped onto model 1 of the apo SL1 structure (PDB: 9EOW).^27^ The color scale ranges from blue (low CSP) to red (high CSP), normalized to the highest CSP within each measurement set. (B) Ligand-induced intensity changes mapped onto the same SL1 model, with the color scale indicating absolute intensity differences from low (blue) to high (red), normalized to the strongest LB within each measurement set. For (A) and (B), residues located in or adjacent to the bulge are labeled, as it is defined as the major binding epitope of the discussed compounds. Residues highlighted in gray refer to purines (not detected in the 2D-^**1**^H,^**1**^H-TOCSY experiment) or signal overlap of C15 and C32, which prevents correct CSP analysis. Additionally, in (B), gray highlights also include terminal and apical loop residues, which are affected by terminal RNA base fraying and internal dynamics. Effects obtained with measurements performed with lead **A** are highlighted in a black box.

### Compound **A** and Its Derivatives Bind to the Bulge of SL1

2D-^1^H,^1^H-TOCSY experiments were performed to characterize the actual ligand binding epitope of SL1. We use the compound of interest in excess of the RNA target to guarantee complete saturation. The bulge of SL1 contains residues A12:A27C28 surrounded by U11 and U13, both of which act as useful probes in the addition of the compounds. The measurements were performed using compounds that had obtained a high-ranking position in the preceding screening routine, as well as compounds that had obtained a moderate score and were used as controls. The range of chemical shifts was utilized to make a statement regarding the binding efficacy of the compound under investigation in the presence of the target RNA (Figure 6, Suppl. Figure 5, Suppl. Tables 5 and 6). When comparing TOCSYs of the moderate compounds with the high scoring ones, A.31 and A.36, it is striking that especially the addition of A.2 and A.4 results in global general shift changes (Figure 6, Suppl. Figure 5). For A.11, A.13, A.31, and A.36, especially the signals of residues within or adjacent to the bulge exhibit moderate CPSs. This observation provides strong evidence that high-scoring compounds target the bulge, rather than engaging in nonspecific binding like the other tested compounds. Mapping the CSPs and LB effects onto the SL1 structure suggests that **A** binds selectively to the bulge. A.13 exhibits a similar binding profile compared to **A**. A.31 and A.36 displayed weaker binding characteristics compared to those of **A** and A.13. In contrast, A.2 and A.4 bound the bulge less selectively, as reflected by the more dispersed CSPs and LB patterns (Figure 6). Further supporting this distinction, the FBS results discussed in Table 1 indicate that broad RNA-detected changes correlate with average ligand shifts, leading to lower binding scores and reinforcing the presence of nonspecific interactions. Estimation of binding affinities revealed similar *K*_D_^est^ values for A.31 (146 ± 13 μM) and A.36 (218 ± 90 μM) when compared to A.2 (144 ± 31 μM; Suppl. Table 7, Suppl. Figure 6). As this method is based on ligand detection, it is subject to bias from signal overlap with RNA signals, which becomes more pronounced during the rising RNA concentrations. However, it is a useful method that allows a quick assessment of the range of binding affinities. In addition, we have applied an RNA-based fluorescence-detected assay using a 2-aminopurine (2AP)-labeled SL1 construct (labeled at position A27) to further probe compound-RNA interactions with compound **A**, A.2 and A.13. The results revealed a consistent trend of increased affinity for the derivatives A.2 and A.13 compared to the lead **A** (Suppl. Figure 7). While the absolute affinities obtained from this fluorescence-based approach differ from the NMR-derived *K*_D_^est^ values, likely due to methodological differences, the observed trend supports the NMR-guided optimization strategy and further confirms the improved RNA interaction properties of the evolved derivative.

### Counterscreen Analysis with an SL1 Construct Missing the Bulge As Proof of Principle

When targeting biomolecular targets in general, a high degree of selectivity needs to be achieved to avoid the off-target effect. Such an improvement of selectivity is particularly daunting for RNA targets, as the chemical diversity of the RNA nucleotide building blocks is sparse compared to proteins. Nevertheless, selectivity can be achieved, as the discovery of RNA riboswitches has documented.^51,52^ For the preliminary selectivity screening, we employed an RNA construct missing the asymmetric 1:2 bulge, which we identified as the major binding site of SL1. In the modified construct, A27 and C28 are replaced with U at position 27 (Figure 7A). Compounds were investigated for their differential binding, thus selectivity, to SL1 compared to binding to the SL1 mutant, further termed SL1 Δ_bulge_. FBS was performed, and specific thresholds for hit classification were not considered, as general effect detection was required for detailed comparison with results obtained with SL1 wt. We observed that 27% of the tested compounds showed residual binding to SL1 Δ_bulge_ (Figure 7B). Generally, CSPs observed in NMR experiments were significantly lower in the presence of SL1 Δ_bulge_ compared to SL1 wt. This decrease in CSPs indicated a generally reduced binding affinity of the evolved compounds toward SL1 Δ_bulge_, further validating the bulge region as the key binding site and, at the same time, demonstrating the selectivity for all the compounds (Figure 7C). Out of the 73% nonbinders, approximately half of the selected compounds originated from the final round of syntheses, which aimed for larger compounds (Suppl. Figures 3 and 4; Suppl. Table 8). This observation suggests that additional RNA functional units in the vicinity of the asymmetric loop can be targeted and exploited to improve binding affinity and selectivity. In addition, the compounds A.2 and A.4 are displayed in the hit selection of the counterscreen, for which CSPs were additionally observed in the apical loop region, and the residual affinity likely originates from here. Interestingly, screening A.13 against SL1 Δ_bulge_ resulted in a complete loss of ligand peaks in all three NMR experiments, indicating an intermediate exchange regime (Figure 7C). This suggests a strong interaction with SL1, which may involve binding to AU base pairs rather than the bulge, as SL1 Δ_bulge_ lacks this structural feature.^53^

**Figure 7:**
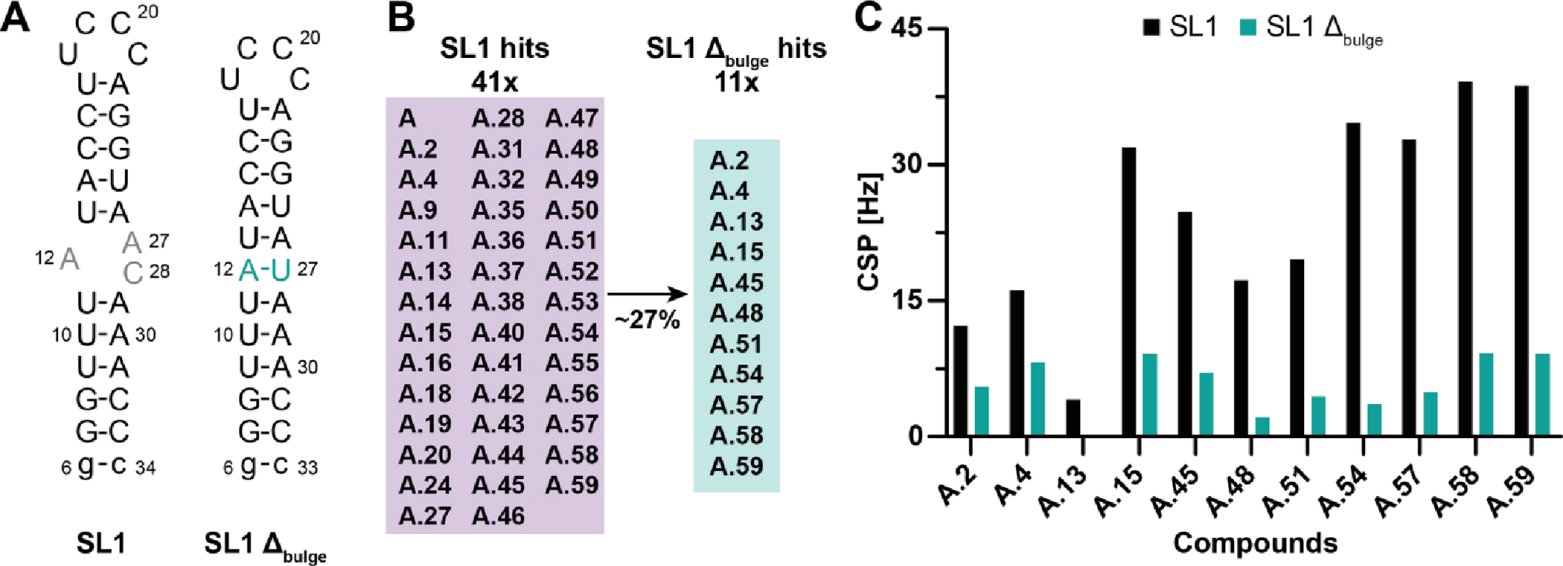
Counterscreen analysis of selected SL1 hits. (A) Specificity for binding to the bulge of SL1 was tested with a construct in which A27 and C28 were replaced by an additional U at position 27 compared to the SL1 wt construct. (B) 43 SL1 ligands were chosen for NMR-based testing against the SL1 Δ_bulge_ construct. Of these 41 compounds, only 11 showed potential binding of the SL1 Δ_bulge_ reflected by the effects detected in the three experiments (1D-^**1**^H NMR, wLOGSY, and CMPG-based *T*_2_-relaxation experiments). (C) Comparative analysis of the CSPs of ligand resonances upon RNA addition between the two constructs, SL1 wt and SL1 Δ_bulge_. CSPs of A.13 in the presence of SL1 Δ_bulge_ are not shown as RNA addition resulted in severe line broadening beyond detection. The samples were measured at a [RNA]:[ligand] ratio of 1:20, with 10 μM of the target RNA and 200 μM of the respective compound.

### Ligand Activity Screening in a Eukaryotic Environment Reveals the Impact of SL1 Ligands on Viral mRNA Translation

To explore the biological effects of the SL1 ligands and validate the potential of our approach, we next determined the effect of the optimized compounds on the translation efficiency of three mRNA reporter constructs. The translation efficiency was evaluated in rabbit reticulocyte lysate by measuring the luminescence of Nanoluciferase. The 5′-UTR of Nanoluciferase mRNA contained either SL1, SL1 Δ_bulge_, or the β-globin-5′-UTR. The inhibitory effect of each small molecule was determined by normalizing translation rates to the untreated RNA-only control. Both SARS-CoV-2-derived constructs, SL1 and SL1 Δ_bulge_, exhibited high “baseline” translation efficiency in the absence of the test compounds compared to the β-globin control construct. In general, the viral constructs showed a sequence-specific tendency toward increased translation efficiency, consistent with similar previous studies.^26^ This inherent property may contribute to their regulatory role in the viral lifecycle and should be considered when analyzing translation modulation in the presence of potential inhibitors. Consequently, the effects of inhibitors need to be interpreted relative to the construct’s intrinsic translation efficiency to accurately assess their impact. Thus, all constructs were individually normalized to their performance in the absence of ligands; they exhibited the same normalized translation efficiency. We used the known antisense DNA oligonucleotide inhibitor ASO#26 targeting the SL1 secondary structure as a positive control for SL1-specific translation inhibition. ASO addition led to a translation inhibition of 92%, confirming the role of the correctly folded SL1 for efficient translation (Figure 8A), even in the absence of the viral protein Nsp1.^30^ Notably, the SL1 Δ_bulge_ construct exhibited only a partial reduction in luminescence in the presence of ASO#26 compared to that of the SL1 wt, underscoring the importance of this structural motif in ASO-mediated inhibition (Figure 8B). In contrast, translation of the control β-globin mRNA remained largely unaffected by ASO treatment, demonstrating the sequence- and structure-specificity of the ASO effect. Based on the results obtained from our previously discussed NMR experiments, we have chosen a set of compounds for the activity-based analysis in RRL. To mention, the functional assays were carried out at physiological pH (7.4–8.0) in contrast to the NMR screening performed at pH 6.2, which captured protonation-dependent conformational exchange. We are aware that this pH difference may contribute to discrepancies in compound activity observed between the two assays. The compounds tested included the high-scoring compounds A.31 and A.36, and compounds with moderate to low effects serving as controls. The tested compounds exhibited varying degrees of translation inhibition, with the most potent compound A.13, reducing translation by ∼50% relative to the untreated control. In contrast, the SL1 Δbulge and β-globin constructs were less affected, with inhibition rates of ∼ 0.5% and ∼10%, respectively (Figure 8B). Interestingly, some small molecules, e.g., A, A.2, and A.36, unexpectedly increased translation efficiency, particularly in the β-globin construct and SL1 Δbulge, suggesting potential unspecific effects in the cell-free translation system or alternative mechanisms influencing translational regulation. Generally, the translation efficiency of the β-globin mRNA did not drop below ∼ 80% in the presence of the SL1-targeting compounds, if not even boosted, thus emphasizing the target specificity of the compounds investigated.

**Figure 8:**
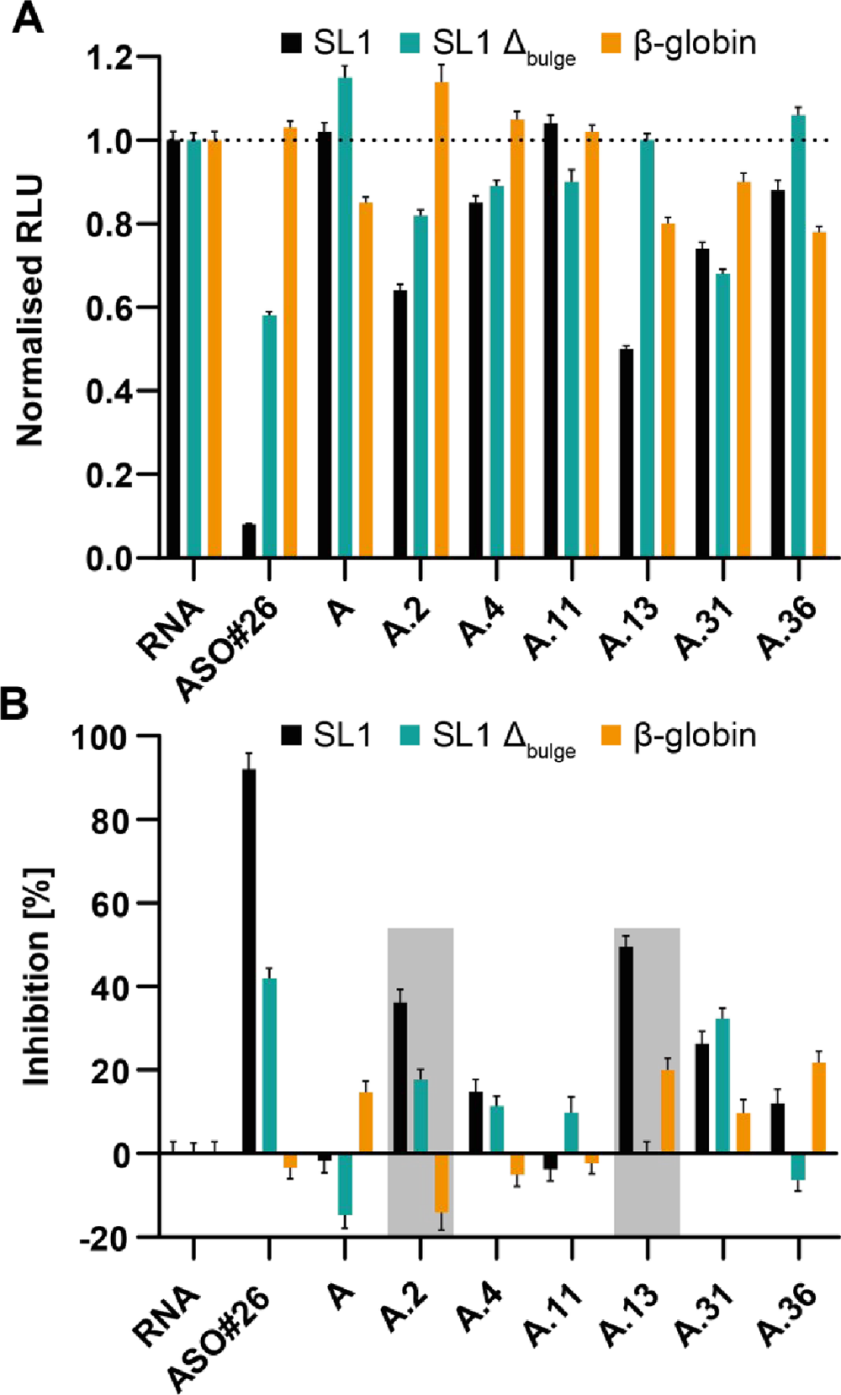
Cell-free translation-readout for compound screening. (A) Normalized luminescence of translated mRNAs encoding Nanoluciferase in the absence and presence of the tested ligands. Samples were normalized to the individual mRNA-only containing reaction. (B) Inhibition rate relative to reactions containing the purified mRNA constructs without compounds or control ASO. Gray boxes highlight compounds A.2 and A.13, the most effective translation-inhibiting compounds. Error bars represent the standard deviation. The samples were measured at a [RNA]:[ligand] ratio of 1:10, with 0.2 μM of the target mRNA and 2 μM of the respective compound.

A comparative evaluation demonstrated that A.2 and A.13 exhibited the highest inhibition efficiency, outperforming lead compound **A**. These results highlight A.2 and A.13 as the most promising candidates for targeted translational inhibition. The counterscreen analysis shown in Figure 7 showed residual affinity of A.2 toward SL1 Δ_bulge_, which was in line with CSPs observed for the apical loop residues in the TOCSY (Suppl. Figure 5). Accordingly, A.2 showed an inhibitory effect on the SL1 Δ_bulge_-mRNA, which was about half as pronounced as that for SL1 wt. Moreover, A.4, also showing minor effects in the counterscreen, displayed an inhibitory effect on both SL1 wt and SL1 Δ_bulge_ mRNA, though its magnitude was smaller than that for A2. This suggests that A.4 may exert a more generalized SL1 inhibition rather than a highly specific interaction. Interestingly, β-globin translation exhibited a boost in the presence of A.2 and A.4, as well as ASO#26. To further characterize the influence on translation by A.2 and A.13, we conducted time-resolved translation assays, which revealed a compound-dependent modulation of translation kinetics: A.13 caused the most pronounced delay and suppression in SL1-driven translation, while **A** and A.2 exhibited more subtle temporal effects (Suppl. Figure 8). Interestingly, translation of SL1 Δ_bulge_ and β-globin mRNAs was consistently enhanced over time in the presence of all tested compounds, further underscoring the sequence- and structure-specific nature of SL1-targeted inhibition.

While A11, A31, and A36 showed either no inhibition (A11) or nonspecific effects (A31 and A36), A13 showed both the greatest translation inhibition and the highest functional specificity. Collectively, these findings reinforce the importance of the bulge as a functional binding site for SL1-targeted translation inhibitors and highlight the need for further investigation of the correlation of selective binding and functional specificity. Another interesting observation was robust translation-enhancing effects by certain compounds. While NMR-based experiments identified A.31 and A.36 as promising SL1-targeting small molecules, the cell-free translation assay revealed that A.2 and A.13 exhibited the highest inhibition rates. Notably, in TOCSY spectra, A.2 showed general CSPs, whereas A.13 displayed small but distinct CSPs and LB of residues within or adjacent to the bulge (Figure 6, Suppl. Figure 5). Furthermore, both compounds were identified as hits for the SL1 Δ_bulge_ mutant, suggesting nonspecific binding to SL1.

## Conclusions

### Final Compound Evaluation Reveals the Need To Optimize between General Targeting and Specificity

We report here on an extensive process to reach low molecular weight compounds that bind to a key RNA element of SARS-CoV-2. In this study, we demonstrated the derivatization of lead **A**, which resulted in significant enhancements in its interactions with SL1 and ultimately in an effective and SL1-specific translation inhibitor. Our group has previously reported on targeting different SARS-CoV-2-derived RNA elements, including the 5′-UTR constructs SL1 to SL4, the isolated SL4 construct, the s2m element in the 3′-UTR, and the PK construct.^54–57^ Notably, these RNAs were generally larger and contained more or larger non-A-form regions suitable for more specific targeting. In our current work, we expanded these earlier investigations by incorporating more extensive derivatization of the start compounds and applied a broader range of tests, specifically focusing on refining the targeting of the SL1 RNA. This approach highlights the versatility and enhanced potential of our pipeline for application to smaller RNAs.^54–57^

This study identified key structural modifications that influenced the binding affinities of RNA-binding small molecules. Specifically, the derivatization of lead structure **A** by methylation of the amide and 4,6-dihydroxy substitution led to a significant improvement in the binding affinities and activity in a biological readout. In general, the derivatization series proved to be effective in enhancing solubility while maintaining or improving the existing binding affinity.

Out of 60 compounds screened against SL1, 41 were identified as hits, with the majority belonging to a set of compounds with increased molecular weight (Figure 9, Suppl. Figure 3). The analysis of FBS results from both SL1 wt and SL1 Δ_bulge_ demonstrated that, e.g., compound A.4 exhibits reduced specificity for the bulge but retains residual biological activity, suggesting that the apical loop also contributes to SL1-mediated translation enhancement. The NMR-estimated binding affinity of A.4 for SL1 wt, in the low micromolar range (102 ± 8 μM; Suppl. Table 7, Suppl. Figure 6), aligns with other compounds tested. Together with the binding site mapping and translation activity data, these findings highlight that affinity alone is not a sufficient criterion for classifying a binder as “good” in terms of biological activity. In conclusion, it was shown that the specific targeting of the bulge is important to link the structure with function, and compound A.4 is a good example of this.

**Figure 9:**
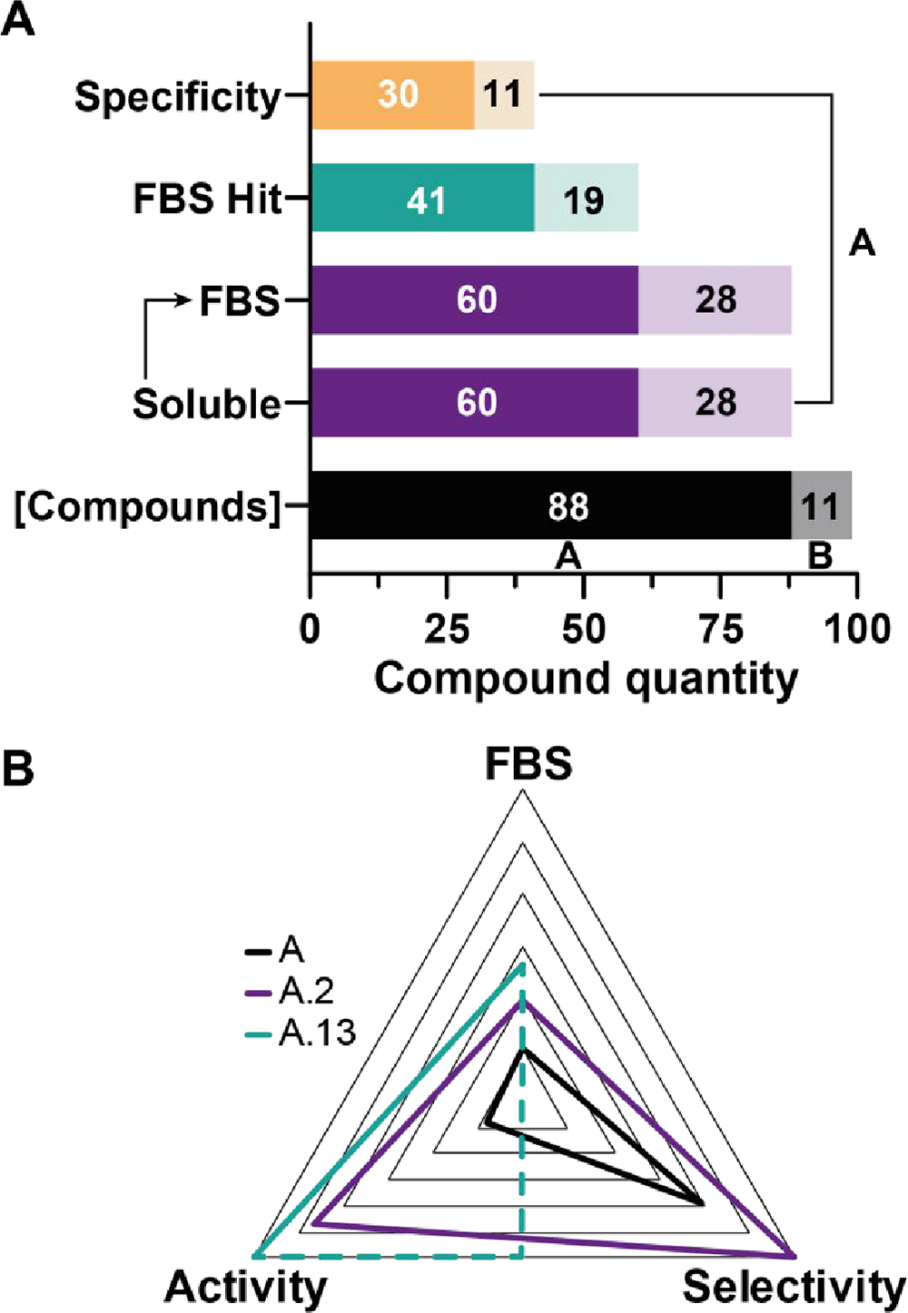
Summarizing Screening Analysis of SARS-CoV-2 SL1. (A) Total set of compounds included in NMR-based studies performed with SL1 (including QC), divided into soluble and insoluble, and FBS results derived from experiments performed with SL1 wt and SL1 Δ_bulge_. (B) Radar chart comparing the FBS, selectivity, and activity scores of lead compound **A** and its derivatives A.2 and A.13. The FBS score obtained in the presence of SL wt was normalized, with 0 representing the lowest and 1 representing the highest score. Selectivity was determined by comparing ^1^H–CSPs of SL wt and SL1 Δ_bulge_, normalizing the absolute CSP differences across the different RNA constructs. Higher values indicate greater selectivity. The selectivity of A.13 could not be assessed due to severe line broadening beyond detection in the presence of SL1 Δ_bulge_. The activity score was based on the normalized inhibition rate from cell-translation assay experiments using SL1 wt Nanoluciferase mRNA, where 0 or lower indicates no inhibition, and 1 represents total inhibition.

Collectively, our findings demonstrate the necessity of achieving a balance between the general targeting of SL1 and the bulge-specific interaction when addressing the, to date, poorly explored SL1 impact on the translation initiation of RNA. Regarding affinity, binding selectivity, and specific translation inhibition, our study resulted in compounds A.2 and A.13 as the most promising candidates for medicinal chemistry campaigns (Figure 9B). In direct comparison with lead **A**, an improvement in terms of FBS scoring, selectivity, and activity was achieved for these compounds, with the exception of selectivity for A.13, as we detected strong LB in all FBS experiments. As mentioned before, the compound might have a tendency to interact with AU base pairs, as seen before for the SL1 wt, and the mutated version carries an additional AU base pair. However, the activity-related experiment showed selective inhibition of SL1 wt mRNA, indicating that A.13 retains specificity for SL1 wt despite the additional AU base pair in the mutant version. This suggests that its interaction with the RNA target remains selective and results in effective inhibition of translation, although its selectivity score could not be determined due to line broadening in FBS experiments.

Our approach, combining compound derivatization with stringent, structure-based binding analysis, on the one hand, and a functional readout, on the other hand, reflects the importance of balancing the specificity and biological activity of RNA ligands. A previous structural motif search of an asymmetric 1:2 P:PY bulge has revealed a rare abundance of solved structures containing this specific pattern.^27^ We must consider the possibility of specific binding to this motif, which might be present in the ribosome and, thus, could impact general translation efficiency. However, if this binding were to occur, it would likely not pose a significant concern, as ribosomes are more abundant in the extract than in the mRNA, meaning the compound would likely be sequestered by the ribosomes, minimizing its impact on translation efficiency.

Based on these results, follow-up strategies for affinity enhancement could be applied. As an example, compound derivatization with favorable functional groups at multiple sites, introducing multivalency, could increase the affinity by avidity. For more complex targets, identification of compounds binding to specific sites would be a basis for a structure-guided compound-fusion strategy.^58,59^ As a follow-up, after the here presented prioritization, the compounds presented here, future studies could explore the broad spectrum of detectable correlations in unlabeled RNA samples to a greater extent to further refine our understanding of RNA-ligand interactions. Lessons learned from this study will also guide future optimization efforts by balancing structural modifications and functional requirements. Moving forward, integrating these techniques with improved computational and experimental workflows will enhance our understanding of RNA-small molecule interactions and guide the design of next-generation RNA binders with optimized properties.

## Supplementary Material





## Data Availability

Experimental raw data are available at 10.25716/gude.1stg-f7ms (Goethe University Data Repository, GUDe).

## Notes

Supplementary methods and experiments mentioned in the main text (PDF)
Experimental details of the compound synthesis (PDF)

## Abbreviations

br: broad
CD_3_OD: deuterated methanol
CDCl_3_: deuterated chloroform
COVID-19: coronavirus disease 2019
CPMG: Carr–Purcell–Meiboom–Gill
CSP: chemical shift perturbation
d: doublet
dd: double doublet
ddd: double double doublet
DMSO-*d*_6_: deuterated dimethyl sulfoxide
DSS: 3-(trimethylsilyl)­propane-1-sulfonate
dt: double triplet
DTT: dithiothreitol
FBS: fragment-based screening
FCC: flash column chromatography
gRNA: genomic RNA
H-bond: hydrogen bond
HIV: human immunodeficiency virus
HRMS: high resolution mass spectra
Hz: Hertz
KCl: potassium chloride
KP_i_: potassium phosphate
LB: line broadening
LiCl: lithium chloride
LNA ASOs: locked nucleic acid antisense oligonucleotides
m: multiplet
mp: melting points
MWCO: molecular weight cutoff
NMR: nuclear magnetic resonance spectroscopy
NOE: Nuclear Overhauser Effect
Nsp1: nonstructural protein 1
ppm: parts per million
PPMOs: antisense peptide-conjugated morpholino oligomers
q: quartet
QC: quality control
RiboTAC: ribonuclease targeting chimeras
RP-HPLC: reverse-phase high-performance liquid chromatography
RRL: rabbit reticulocyte lysate
RT: room temperature
s: singlet
SAR: structure–activity relationships
SARS-CoV-2: severe acute respiratory syndrome coronavirus 2
SL1: stem-loop 1
SOGGY: solvent-optimized double gradient spectroscopy
t: triplet
TAR: transactivating response region
TOCSY: total correlation spectroscopy
UTR: untranslated region
wLOGSY: water-ligand observed via gradient spectroscopy
wt: wild-type
