# Supplementary material for: Targeting the SARS-CoV‑2 RNA Translation Initiation Element SL1 by Molecules of Low Molecular Weight
Source: J Am Chem Soc. 2025 Aug 4;147(32):28783–98. doi: 10.1021/jacs.5c05264 (PMC12356538; doi:10.1021/jacs.5c05264)
Supplement: Supplementary file 1 [file ja5c05264_si_001.pdf]

Targeting the SARS-CoV-2 RNA translation initiation element SL1 by molecules of low molecular weight

Sabrina Toews[1], Francesca Donà[2], Jürgen Krauß[2], Franz Bracher[2], Úrsula López-García[3], Jörg Pabel[3], Daniel Merk[3], Marcel J.J Blommers[4], Anna Wacker[1], Jan Ferner[1], Christian Richter[1] and Harald Schwalbe[1,*]

[1]Institute for Organic Chemistry and Chemical Biology and Center for Biomolecular Magnetic Resonance (BMRZ), Goethe University Frankfurt am Main, Frankfurt/Main, Hesse 60438, Germany

[2]Pharmaceutical Chemistry, Department of Pharmacy, Center for Drug Research, Ludwig-Maximilians-University Munich, Munich, Bavaria 81377, Germany

[3]Department of Pharmacy, Ludwig-Maximilians-University Munich, Munich, Bavaria 81377, Germany

[4]Saverna Therapeutics, Biel-Benken, 4105, Switzerland

*To whom correspondence should be addressed. Tel: +49 (0)69 / 798-29737; Fax: +49 (0)69 / 798-29515; Email: schwalbe@nmr.uni-frankfurt.de

KEYWORDS: NMR spectroscopy, RNA, SARS-CoV-2, Screening, Drug design

Table of Contents

RNA constructs 2
Docking calculations 3
Derivatives of lead compound A 4
NMR-based studies performed in the presence of the synthesized small molecules 6
Derivatives of lead compound B 11
Chemical structures of the derivatives of lead compound A 12
NMR-based binding site mappings 14
Binding affinities 15
Cell-free translation assay – time-resolved control experiment 17
References 18

SUPPLEMENTARY INFORMATION I - Main text

RNA constructs

Suppl. Table 1. Sequences of the used RNA constructs.

Construct	RNA sequence [5' → 3']
SL1 (wt)	GGGUUUAUACCUUCCCAGGUAACAAACCC
SL1 Δ_{bulge}	GGGUUUAUACCUUCCCAGGUAUAAACCC
SL1 (wt)-Nanoluciferase PolyA	AUUAAAGGUUUAUACCUUCCCAGGUAACAAACCAACCAACUUUCACCAUGGUCUUCA<br>CACUCGAAGAUUUCGUUGGGGACUGGCGACAGACAGCCGGCUACAACCUGGACCAAG<br>UCCUUGAACAGGGAGGUGUGUCCAGUUUGUUUCAGAAUCUCGGGGUGUCCGUAACUC<br>CGAUCCAAAGGAUUGUCCUGAGCGGUGAAAAUGGGCUGAAGAUCGACAUCCAUGUCA<br>UCAUCCCGUAUGAAGGUCUGAGCGGCGACCAAAUGGGCCAGAUCGAAAAAAUUUUUA<br>AGGUGGUGUACCCUGUGGAUGAUCAUCACUUUAAGGUGAUCCUGCACUAUGGCACAC<br>UGGUAAUCGACGGGGUUACGCCGAACAUGAUCGACUAUUUCGGACGGCCGUAUGAAG<br>GCAUCGCCGUGUUCGACGGCAAAAAGAUCACUGUAACAGGGACCCUGUGGAACGGCA<br>ACAAAAUUAUCGACGAGCGCCUGAUCAACCCCGACGGCUCCCUGCUGUUCCGAGUAA<br>CCAUCAACGGAGUGACCGGCUGGCGGCUGUGCGAACGCAUUCUGGCGUAAAAAAAAA<br>AAAAAAAAAAAAAAAAAAAAAAAAAAAAAAAAAAAAAAAAAAAAAAAAAA
SL1 Δ_{bulge}-Nanoluciferase PolyA	AUUAAAGGUUUAUACCUUCCCAGGUAUAAACCAAACCAUGGUCUUCACACUCGAAGA<br>UUUCGUUGGGGACUGGCGACAGACAGCCGGCUACAACCUGGACCAAGUCCUUGAACA<br>GGGAGGUGUGUCCAGUUUGUUUCAGAAUCUCGGGGUGUCCGUAACUCCGAUCCAAAG<br>GAUUGUCCUGAGCGGUGAAAAUGGGCUGAAGAUCGACAUCCAUGUCAUCAUCCCGUA<br>UGAAGGUCUGAGCGGCGACCAAAUGGGCCAGAUCGAAAAAAUUUUUAAGGUGGUGU<br>ACCCUGUGGAUGAUCAUCACUUUAAGGUGAUCCUGCACUAUGGCACACUGGUAAUCG<br>ACGGGGUUACGCCGAACAUGAUCGACUAUUUCGGACGGCCGUAUGAAGGCAUCGCCG<br>UGUUCGACGGCAAAAAGAUCACUGUAACAGGGACCCUGUGGAACGGCAACAAAAUUA<br>UCGACGAGCGCCUGAUCAACCCCGACGGCUCCCUGCUGUUCCGAGUAACCAUCAACG<br>GAGUGACCGGCUGGCGGCUGUGCGAACGCAUUCUGGCGUAAAAAAAAAAAAAAAAAA<br>AAAAAAAAAAAAAAAAAAAAAAAAAAAAAAAAAAAAAAA
β-Globin-Nanoluciferase PolyA	ACAUUUGCUUCUGACACAACUGUGUUCACUAGCAACCUCAAACAGACACCAUGGUCU<br>UCACACUCGAAGAUUUCGUUGGGGACUGGCGACAGACAGCCGGCUACAACCUGGACC<br>AAGUCCUUGAACAGGGAGGUGUGUCCAGUUUGUUUCAGAAUCUCGGGGUGUCCGUAA<br>CUCCGAUCCAAAGGAUUGUCCUGAGCGGUGAAAAUGGGCUGAAGAUCGACAUCCAUG<br>UCAUCAUCCCGUAUGAAGGUCUGAGCGGCGACCAAAUGGGCCAGAUCGAAAAAAUUU<br>UUAAGGUGGUGUACCCUGUGGAUGAUCAUCACUUUAAGGUGAUCCUGCACUAUGGCA<br>CACUGGUAAUCGACGGGGUUACGCCGAACAUGAUCGACUAUUUCGGACGGCCGUAUG<br>AAGGCAUCGCCGUGUUCGACGGCAAAAAGAUCACUGUAACAGGGACCCUGUGGAACG<br>GCAACAAAAUUAUCGACGAGCGCCUGAUCAACCCCGACGGCUCCCUGCUGUUCCGAG<br>UAACCAUCAACGGAGUGACCGGCUGGCGGCUGUGCGAACGCAUUCUGGCGUAAAAAA<br>AAAAAAAAAAAAAAAAAAAAAAAAAAAAAAAAAAAAAAAAAAAAAAAAAAAAA

Docking calculations

Suppl. Table 2. HADDOCK 2.5 results of dockings performed with SL1 and the respective compounds (**A** and **B**). Dockings were performed the SARS-CoV-2 SL1 solution structure (PDB: 9EOW)[1]. Shown are the interactions between the ligands and SL1 of the lowest energy docking pose.

	Interaction type	From	From Chemistry	To	To Chemistry	Distances [Å]
**Compound A**	Conventional Hydrogen Bond	A:H24	H-Donor	SL1:A12:O2'	H-Acceptor	3.00
	π-π Stacked	SL1:U13	π-Orbitals	A	π-Orbitals	5.21
	π-π Stacked	SL1:A29	π-Orbitals	A	π-Orbitals	4.75
	π-π Stacked	SL1:A29	π-Orbitals	A	π-Orbitals	4.46
	π-π Stacked	SL1:A29	π-Orbitals	A	π-Orbitals	5.35
	π-π Stacked	A	π-Orbitals	SL1:A29	π-Orbitals	4.61
	π-Alkyl	SL1:U11	π-Orbitals	A:C16	Alkyl	4.57
	π-Alkyl	SL1:A12	π-Orbitals	A:C16	Alkyl	5.09
	π-Alkyl	SL1:A27	π-Orbitals	A:C11	Alkyl	5.02
	π-Alkyl	SL1:A29	π-Orbitals	A:C16	Alkyl	4.38
**Compound B**	Conventional Hydrogen Bond	SL1:U11:HO2'	H-Donor	B:O18	H-Acceptor	2.53
	Conventional Hydrogen Bond	SL1:A29:HO2'	H-Donor	B:O17	H-Acceptor	2.78
	Conventional Hydrogen Bond	B:HAA	H-Donor	SL1:A12:O2'	H-Acceptor	2.67
	Conventional Hydrogen Bond	B:H19	H-Donor	SL1:A12:O2'	H-Acceptor	2.21
	π i-Cation	B:N2	Positive	SL1:A29	π-Orbitals	4.90
	π-π T-shaped	SL1:A29	π-Orbitals	B	π-Orbitals	4.63

Derivatives of lead compound A

Suppl. Table 3. Compounds part of the derivatization series of lead **A** which passed 1D-^{1}H-NMR-based quality control (QC).

Cmpd	SMILES	QC passed?
**A**	COC1=CC=C(N=C(NC(NC)=O)S2)C2=C1	**yes**
**A.1**	O=C(NCC)NC1=NC2=CC=CC=C2S1	**yes**
**A.2**	O=C(CN(C)C)NC1=NC(C=C2)=C(C=C2OC)S1	**yes**
**A.3**	COC1=CC2=C(C=C1)N=C(/N=C3NC(CS\3)=O)S2	**yes**
**A.4**	COC1=CC2=C(C=C1)N=C(NC(NC)=N)S2	**yes**
**A.5**	COC(CC(NC1=NC2=C(S1)C=CC=C2)=O)=O	**yes**
**A.6**	CC(NC1=NC2=C(S1)C=CC=C2)=O	**yes**
**A.7**	O=C(C(C)(C)C)NC1=NC2=C(C=CC=C2)S1	**yes**
**A.8**	O=C(C(C)(C)C)NC1=NC2=C(C=C(Br)C=C2)S1	**yes**
**A.9**	OC1=CC=C(N=C(NC(NC)=O)S2)C2=C1	**yes**
**A.10**	CCN1C2=CC=CC=C2N=C1CC#N	**yes**
**A.11**	CNC(Nc1nc(c(OC)cc(OC)c2)c2s1)=O	**yes**
**A.12**	CN(C)CC(Nc1nc(ccc(O)c2)c2s1)=O	**yes**
**A.13**	CNC(Nc1nc(c(O)cc(O)c2)c2s1)=O	**yes**
**A.14**	CN(C)CC(Nc1nc(ccc([N+]([O-])=O)c2)c2s1)=O	**yes**
**A.15**	COC1=CC(SC(NC(CN(C)C)=O)=N2)=C2C(OC)=C1	**yes**
**A.16**	BrC1=CC=C2C(SC(NC(CN(C)C)=O)=N2)=C1	**yes**
**A.17**	O=C(CN(C)C)NCC1=NC2=CC=CC=C2S1	**yes**
**A.18**	COC1=CC=C2C(SC(NC(CN3CCN(C)CC3)=O)=N2)=C1	**yes**
**A.19**	O=C(CN(C)C)NC1=NC2=CC=C(SC)C=C2S1	**yes**
**A.20**	COC1=CC=C(N=C(NC(CN(C)C)=O)N2)C2=C1	**yes**
**A.21**	COC(C=C1)=CC=C1C(S2)=NN=C2NC(CN(C)C)=O	**yes**
**A.22**	NC1=NN=C(C2=CC=C(OC)C=C2)S1	**yes**
**A.23**	NC1=NN=C(C2=CC=C(OC)C(OC)=C2)S1	**yes**
**A.24**	NC1=CC=C(N=C(NC(CN(C)C)=O)S2)C2=C1	**yes**
**A.25**	O=C(CN(CC1=CC=CC=C1)C)NC2=NC3=C(OC)C=C(OC)C=C3S2	**yes**
**A.26**	O=C(CN(C)C1=O)N1C2=NC3=C(OC)C=C(OC)C=C3S2	**yes**
**A.27**	O=C(CN(C)C)NC1=NC2=CC=C(C(F)(F)F)C=C2S1	**yes**
**A.28**	FC(F)(F)OC1=CC=C2C(SC(NC(CN(C)C)=O)=N2)=C1	**yes**
**A.29**	O=C(CN(C)CCN(C)C)NC1=NC2=CC=C(OC)C=C2S1	**yes**
**A.30**	COC1=CC=C2C(SC(NC(CN(C)CCOC)=O)=N2)=C1	**yes**
**A.31**	COC1=CC(C2=NN=C(NC(CN(C)C)=O)S2)=CC=C1OC	**yes**
**A.32**	CS(C1=CC=C2C(SC(NC(CN(C)C)=O)=N2)=C1)(=O)=O	**yes**
**A.33**	COC1=CC=C(N=C(NC(CN(C)CC2=CN=CN=C2)=O)S3)C3=C1	**yes**
**A.34**	COC1=CC=C2C(SC(N(C(CN(C)C)=O)C)=N2)=C1	**yes**
**A.35**	COC1=CC=C2C(OC(NC(CN(C)C)=O)=N2)=C1	**yes**
**A.36**	COC1=CC=C2C(SC(NC(CN3C[C@@H](N)CCC3)=O)=N2)=C1	**yes**
**A.37**	COC1=CC=C2C(SC(NC(CN3C[C@H](N)CCC3)=O)=N2)=C1	**yes**
**A.38**	COC1=CC=C2C(SC(NC(CN(C)C)=O)=N2)=N1	**yes**
**A.39**	COC1=CC=C2C(SC(NC(CNC3CC(C)(C)NC(C)(C)C3)=O)=N2)=C1	**yes**
**A.40**	COC1=CC=C2C(NC(NC(CN3CCN(C)CC3)=O)=N2)=C1	**yes**
**A.41**	O=[N+]([O-])C1=CC(C2=NN=C(NC(CN(C)C)=O)S2)=CC([N+]([O-])=O)=C1	**yes**
**A.42**	[O-][N+](C1=CC=C(C=C1)C2=NN=C(NC(CN(C)C)=O)S2)=O	**yes**
**A.43**	ClC1=C(F)C=C(C(Cl)=C1[N+]([O-])=O)C2=NN=C(NC(CN(C)C)=O)S2	**yes**
**A.44**	COC1=CC=C2C(SC(NC(CN(C)C3CC(C)(C)NC(C)(C)C3)=O)=N2)=C1	**yes**
**A.45**	COC1=CC=C2C(SC(NC(CN3CCNCC3)=O)=N2)=C1	**yes**
**A.46**	ClC1=NC=C2C(SC(NC(CN(C)C)=O)=N2)=N1	**yes**

**A.47**	O=C(CN(C)C)NC1=NC2=CN=C(OC)N=C2S1	**yes**
**A.48**	OC1=CC(N(C)CCN[C@@H](CCC2)CN2CC(NC3=NC4=CC=C(OC)C=C4S3)=O)=CC=C1	**yes**
**A.49**	OC1=CC(N(CC)CCN[C@@H](CCC2)CN2CC(NC3=NC4=CC=C(OC)C=C4S3)=O)=CC=C1	**yes**
**A.50**	OC1=CC(N(C)CCN[C@H](CCC2)CN2CC(NC3=NC4=CC=C(OC)C=C4S3)=O)=CC=C1	**yes**
**A.51**	OC1=CC(N(CC)CCN[C@H](CCC2)CN2CC(NC3=NC4=CC=C(OC)C=C4S3)=O)=CC=C1	**yes**
**A.52**	COC1=CC=C(N=C(NC(CN2C[C@@H](NCC3=CC=CC(O)=C3)CCC2)=O)S4)C4=C1	**Yes**
**A.53**	COC1=CC=C(N=C(NC(CN2C[C@H](NCC3=CC=CC(O)=C3)CCC2)=O)S4)C4=C1	**yes**
**A.54**	COC1=CC(OC)=C2C(SC(NC(CN3C[C@@H](N)CCC3)=O)=N2)=C1	**yes**
**A.55**	COC1=CC(OC)=C2C(SC(NC(CN3C[C@H](N)CCC3)=O)=N2)=C1	**yes**
**A.56**	COC1=CC(OC)=C2C(SC(NC(CN3CCN(C)CC3)=O)=N2)=C1	**yes**
**A.57**	COC1=CC(OC)=C2C(SC(NC(CN3CCNCC3)=O)=N2)=C1	**yes**
**A.58**	COC1=CC(C2=NN=C(NC(CN3C[C@@H](N)CCC3)=O)S2)=CC=C1OC	**yes**
**A.59**	COC1=CC(C2=NN=C(NC(CN3C[C@H](N)CCC3)=O)S2)=CC=C1OC	**yes**

Suppl. Table 4. Compounds part of the derivatization series of lead **A** which did not pass 1D-^{1}H-NMR-based QC.

Cmpd	SMILES	QC passed?
**x.1**	O=C(NC1=NC2=C(C=CC=C2)S1)NC(C)(C)C	**no**
**x.2**	O=C(NC1=NC2=C(C=C(OC)C=C2)S1)NC(C)(C)C	**no**
**x.3**	O=C(NC1=NC2=C(C=C(Br)C=C2)S1)NC(C)(C)C	**no**
**x.4**	S=C(NC)NC1=NC2=C(S1)C=CC=C2	**no**
**x.5**	COC1=CC2=C(C=C1)N=C(NC3=CN=C(N)S3)S2	**no**
**x.6**	COC1=CC2=C(C=C1)N=C(NC(OC)=O)S2	**no**
**x.7**	COC1=CC2=C(C=C1)N=C(NS(=O)(C)=O)S2	**no**
**x.8**	COC1=CC(SC(NC(C)=O)=N2)=C2C=C1	**no**
**x.9**	COC1=CC2=C(C=C1)N=C(NC(CC(OC)=O)=O)S2	**no**
**x.10**	COC1=CC2=C(C=C1)N=C(NC(C3CC3)=O)S2	**no**
**x.11**	NC(NC1=NC2=C(C=C(OC)C=C2)S1)=O	**no**
**x.12**	O=C(C1CC1)NC2=NC3=C(C=CC=C3)S2	**no**
**x.13**	CC(NC1=NC2=C(S1)C=C(Br)C=C2)=O	**no**
**x.14**	CC(NC1=NN=C(S1)S(N)(=O)=O)=O	**no**
**x.15**	Nc1nc(ccc(N)c2)c2s1	**no**
**x.15**	COc(cc1OC)cc2c1nc(N)s2	**no**
**x.16**	CNC(NCc1nc(cccc2)c2s1)=O	**no**
**x.17**	CNC(NCc1nc(cccc2)c2s1)=S	**no**
**x.18**	CNC(Nc1nc(ccc(O)c2)c2s1)=N	**no**
**x.19**	COC1=CC2=C(C=C1)N=C(NC(OCC3=CC=CC=C3)=O)S2	**no**
**x.20**	NC(NC1=NC2=C(C=CC=C2)S1)=O	**no**
**x.21**	BrC1=CC=C(N=C(NC(NC)=O)S2)C2=C1	**no**
**x.22**	NC(NC1=NC2=C(C=C(Br)C=C2)S1)=O	**no**
**x.23**	OC1=CC(O)=C2C(SC(NC(CN(C)C)=O)=N2)=C1	**no**
**x.24**	S=C(NC1=CN=CS1)NC1=CC=CC=C1	**no**
**x.25**	IC1=CC=C(NC(=S)NC2=CN=CS2)C=C1	**no**
**x.26**	COC(C=C1)=CC=C1C(S2)=NN=C2NC(CCl)=O	**no**
**x.27**	COC(C=C1)=CC=C1C(S2)=NN=C2NC(C)=O	**no**

NMR-based studies performed in the presence of the synthesized small molecules

Suppl. Table 5. Chemical shift perturbations (CSPs) in ppm of SL1's pyrimidine resonances detected by 2D-$^{1}H,^{1}H$ TOCSY experiments. Unassignable resonances are marked with -.

	$^{1}H,^{1}H$ CSPs [ppm]							
**Assignment**	**A**	**A.2**	**A.4**	**A.11**	**A.13**	**A.31**	**A.36**	**B**
**U9H5-H6**	0.0117	0.0247	0.0130	0.0040	0.0067	0.0104	0.0100	0.0063
**U10H5-H6**	0.0179	0.0231	0.0146	0.0072	0.0112	0.0112	0.0089	0.0122
**U11H5-H6**	0.0824	0.0462	0.0030	0.0311	0.0702	0.0256	0.0063	0.0220
**U13H5-H6**	0.0242	0.0110	0.0999	0.0216	0.0209	0.0206	0.0308	0.0256
**C15H5-H6**	0.0081	-	-	0.0010	0.0030	0.0032	0.0010	0.0208
**C16H5-H6**	0.0050	0.0166	0.0103	0.0020	0.0032	0.0073	0.0076	0.0120
**U17H5-H6**	0.0100	0.0143	0.0288	0.0010	0.0051	0.0067	0.0045	0.0061
**U18H5-H6**	0.0010	0.0071	0.0191	0.0043	0.0022	0.0021	0.0039	0.0064
**C19H5-H6**	0.0050	0.0190	0.0293	0.0036	0.0022	0.0042	0.0036	0.0092
**C20H5-H6**	0.0050	0.0143	0.0526	0.0045	0.0022	0.0021	0.0029	0.0030
**C21H5-H6**	0.0095	0.0230	0.0441	0.0061	0.0045	0.0010	0.0010	0.0058
**U25H5-H6**	0.0160	0.0361	0.0099	0.0045	0.0020	0.0122	0.0120	0.0085
**C28H5-H6**	0.0502	0.0813	0.1355	0.0130	0.0278	0.0228	0.0359	0.0263
**C32H5-H6**	0.0124	-	-	0.0063	0.0082	0.0130	0.0186	0.0336
**C33H5-H6**	0.0042	0.0085	0.0050	0.0010	0.0032	0.0067	0.0057	0.0110
**C34H5-H6**	0.0054	0.0081	0.0072	0.0025	0.0087	0.0204	0.0079	0.0021

Suppl. Table 6. CSPs in ppm of SL1's resonances detected by 1D-^{1}H experiments. Unassignable resonances are marked with -.

	^{1}H CSPs [ppm]						
**Assignment**	**A**	**A.2**	**A.4**	**A.11**	**A.13**	**A.31**	**A.36**
A12H2	0.0041	0.1420	0.1560	0.0400	0.0630	0.0630	0.0727
A29H2	-	0.0190	0.0670	0.0040	0.0190	0.0000	-

Suppl. Table 7. Estimation of binding affinities (K_D^{est}) via ligand-detected 1D-^{1}H-NMR titrations. Titrations were performed with eight individual samples keeping the ligand concentration constant at 100 μM and varying the SL1 wt RNA concentration from 0 up to 200 or even 250 μM. Ligand resonances could not be traced through all eight measurements for some the ligands as shown by the number of analyzed points.

RNA concentration	Compound	K_D^{est} [μM]	Std. error [μM]	analyzed points
**0-250 μM**	A	135.7	34.3	8
	A.2	143.8	31.4	8
	A.4	101.8	7.5	8
	A.8	253.9	53.1	8
	A.9	435.9	44.1	8
	A.13	311.9	48.6	8
	B	149.5	46.2	8
**0-200 μM**	A.15	108.2	29.5	8
	A.31	145.9	13.4	8
	A.36	217.8	89.9	8
	A.37	171.4	20.5	8
	A.45	161.7	16.1	8
	A.48	304.1	20.1	8
	A.49	529.8	245.1	7
	A.50	ambiguous		8
	A.51	739.9	92.2	8
	A.54	109.4	79.0	8
	A.55	81.1	17.5	6
	A.56	75.3	24.8	6
	A.57	83.7	14.5	6
	A.58	77.5	12.2	8
	A.59	90.7	14.1	7

Suppl. Table 8. Ranked FBS results of **A** and its derivatives classified as hits measured in the presence of the counterscreen RNA SL1 Δ_{bulge}. The compounds were ranked on their results derived from three individual results: I) 1D-^{1}H NMR; II) wLOGSY experiments; and III) CMPG-based T_2-relaxation experiments (T_2 = 5 or 100 ms). The higher the score, the stronger the effects found in these experiments.

Rank	Compound	CSP [Hz]	wLOGSY factor	T_2 reduction [%]
**no effects**	A	**no effects**		
	A.8			
	A.9			
	A.11			
	A.14			
	A.16			
	A.18			
	A.19			
	A.20			
	A.24			
	A.25			
	A.27			
	A.28			
	A.31			
	A.32			
	A.35			
	A.36			
	A.37			
	A.38			
	A.40			
	A.41			
	A.42			
	A.43			
	A.44			
	A.46			
	A.47			
	A.49			
	A.50			
	A.52			
	A.53			
	A.55			
	A.56			
**1**	A.4	8.19	0.69	90.18
**x**	**A.13**	**severe line broadening; signal disappearance**		
**2**	A.15	9.18	0.47	84.62
**2**	A.58	9.24	0.00	73.56
**4**	A.59	9.18	0.00	65.47
**5**	A.2	5.52	0.00	67.46
**5**	A.54	3.60	0.00	88.68
**7**	A.45	7.08	0.00	60.81
**8**	A.57	4.92	1.07	47.32
**9**	A.48	2.16	10.82	0.59
**10**	A.51	4.44	0.00	42.57

Suppl. Table 9. Values used for the radar chart comparing the FBS, selectivity, and activity scores of lead compound **A** and its derivatives A.2 and A.13. The FBS score obtained in the presence of SL wt was normalized, with 0 representing the lowest and 1 the highest score. Selectivity was determined by comparing ^{1}H-CSPs of SL wt and SL1 Δ_{bulge}, normalizing the absolute CSP differences across the different RNA constructs. Higher values indicate greater selectivity. The selectivity of A.13 could not be assessed due to severe line broadening beyond detection in the presence of SL1 Δ_{bulge}. The activity score was based on the normalized inhibition rate from cell-translation assay experiments using SL1 wt Nanoluciferase mRNA, where 0 or lower indicates no inhibition and 1 represents total inhibition.

	**Normalized selectivity score**				**Normalized activity score**		**Normalized FBS score**		
**Compound**	**SL1 wt CSP [Hz]**	**SL1 Δ_{bulge} CSP [Hz]**	**abs. CSP SL1 wt - CSP SL1 Δ_{bulge}**	**Normalized abs. CSP SL1 wt - CSP SL1 Δ_{bulge}**	**SL1 wt inhibition rate [%]**	**Normalized inhibition rate**	**FBS score**	**Normalized FBS score**	**1 - normalized FBS score**
**A**	3.96	0.00	3.96	0.59	-1.66	-0.03	37	1.00	0.00
**A.2**	12.23	5.52	6.71	1.00	36.23	0.73	30	0.81	0.19
**A.13**	4.11	**x**	**x**	**x**	49.50	1.00	25	0.68	0.32

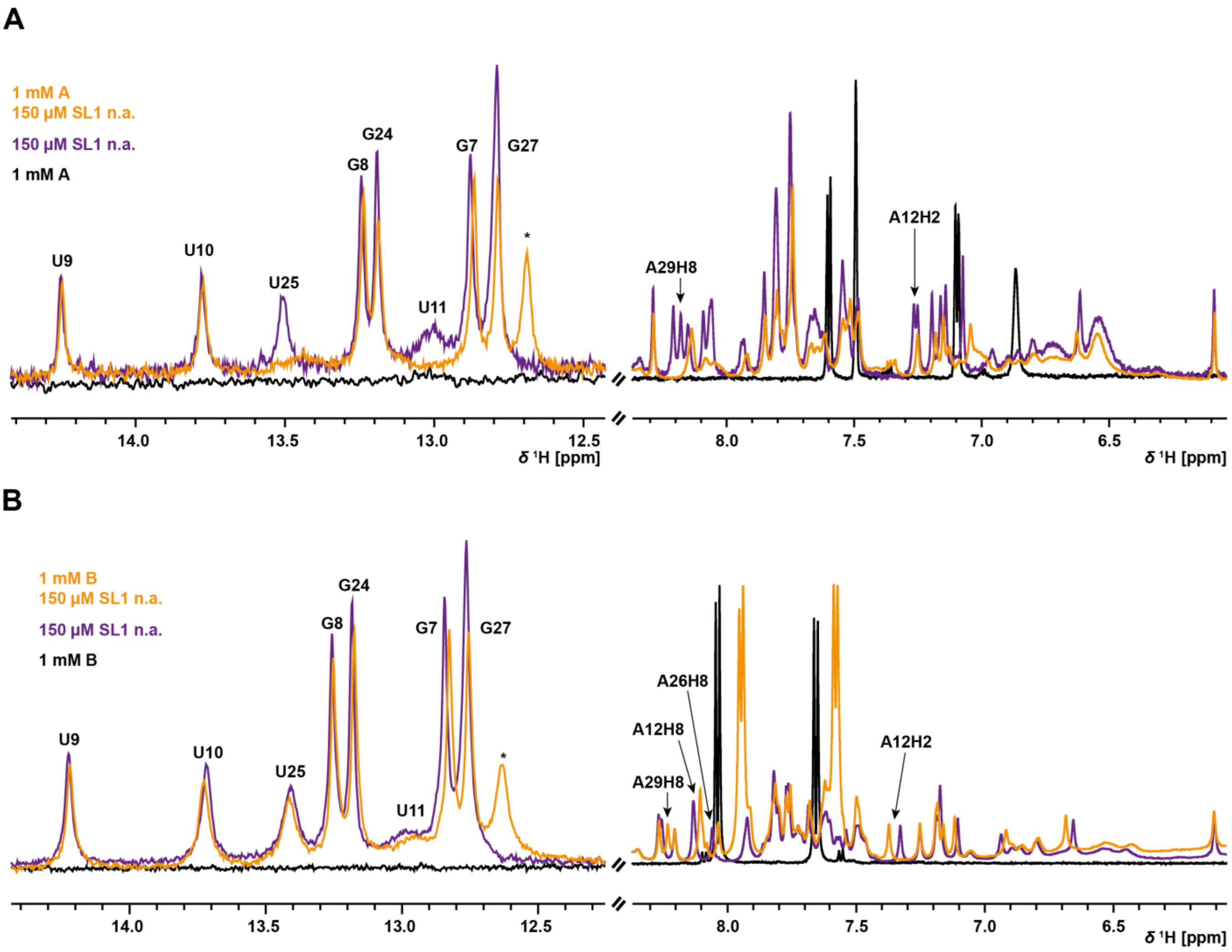

Suppl. Figure 1. A-B 1D-^{1}H overlays of SL1 and SL1 in the presence of **A** and **B**, respectively. NMR spectra with compound **A** were recorded at 800 MHz and 298 K, and spectra with compound **B** at 600 MHz and 298 K. Measurements were performed with an unlabeled RNA (150 µM) sample and a sample containing both the compound (1 mM) and the unlabeled RNA (150 µM).

Derivatives of lead compound B

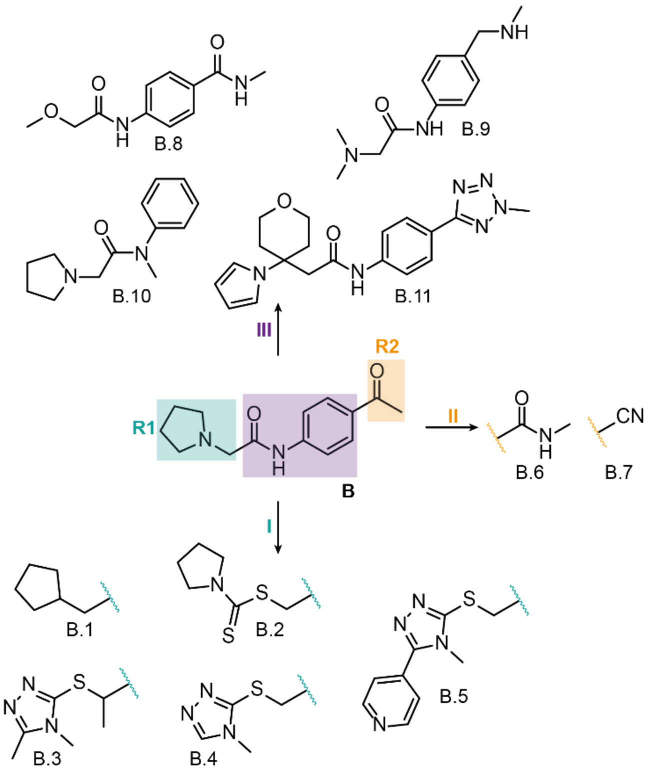

Suppl. Figure 2. Depicted sites for synthetic modification of the lead compound B: I) substitution of R1 II) substitution of R2; and III) complete modification of the lead besides preserving the 4-amidoacetophenone motif.

The modifications of lead **B** were introduced in three different ways: I) substitution of the pyrrolidine moiety, II) substitution of the ketone moiety, and III) broad variation of the amide substituent on the 4-amidoacetophenone motif. (Suppl. Fig. 2). All modifications on **B** such as replacing the methyl ketone with an *N*-methyl carboxamide, altering the pyrrolidine moiety through ring extension or substitution, removing the pyrrolidine nitrogen, or introducing an aromatic residue all resulted in a loss of interaction with SL1. Similarly, small heterocyclic, hydrophobic, and bulky modifications disrupted binding, emphasizing the structural constraints necessary for maintaining SL1 interaction.

We additionally concluded that the 4-amidoacetophenone scaffold bearing a polar, potentially basic substituent was essential. Thus, the 4-amidoacetophenone structure of **B** was fixed and the backbone was further explored with structurally diverse alternative groups (B.1, B.7, B.8, B.9 and B.11, Fig. 3). Despite sharing the 4-amidoacetophenone motif, B.1, B.7, B.8, B.9, and B.11 covered a broad chemical space. The introduction of several alternative small heterocyclic motifs, together with more hydrophobic and bulky groups, resulted in a total loss of function.

With this outcome, we want to emphasize that previous binding characteristics were not recovered or improved which led us to the final decision to classify results obtained with this compound as a negative example and stop further derivatization.

Chemical structures of the derivatives of lead compound A

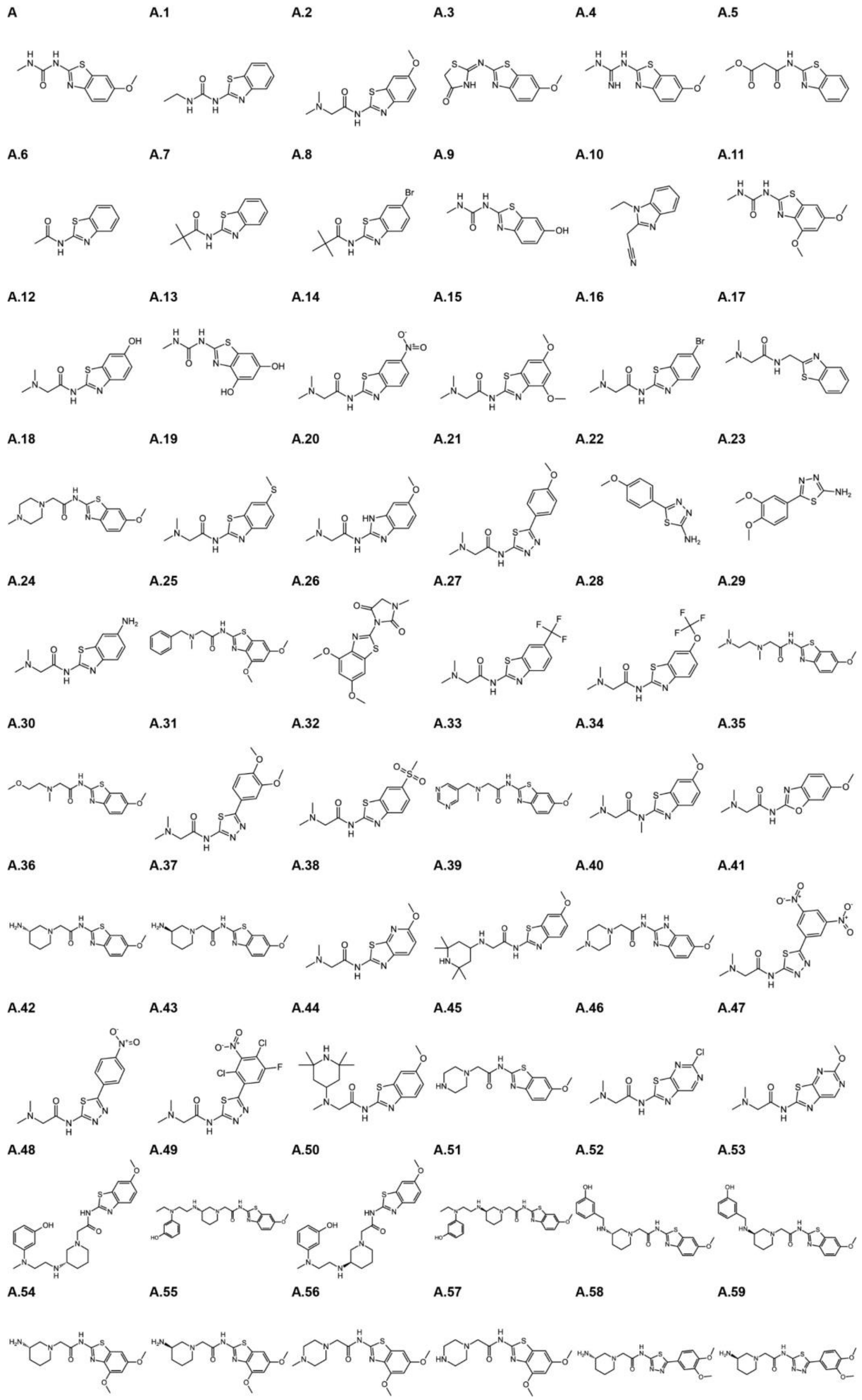

Suppl. Fig. 3. Chemical structures of the compounds part of the derivatization series of lead **A** which passed 1D-^{1}H-NMR-based QC.

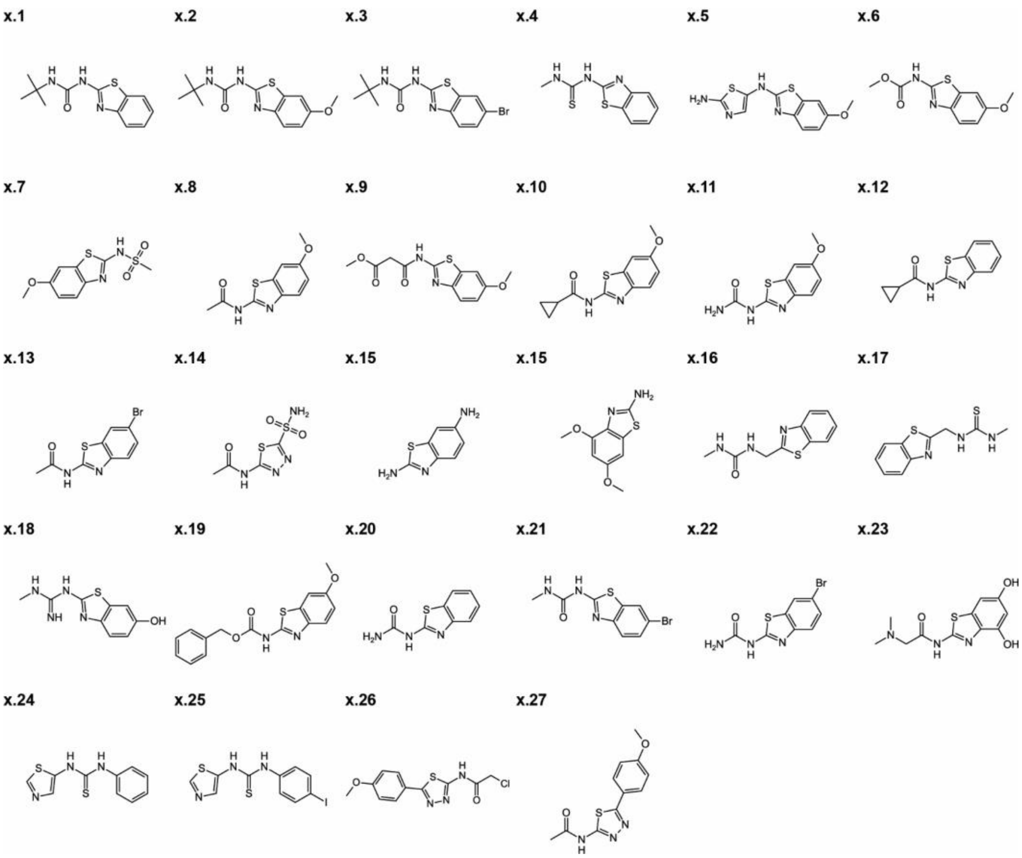

Suppl. Fig. 4. Chemical structures of the compounds part of the derivatization series of lead **A** which did not pass 1D-^{1}H-NMR-based QC.

NMR-based binding site mappings

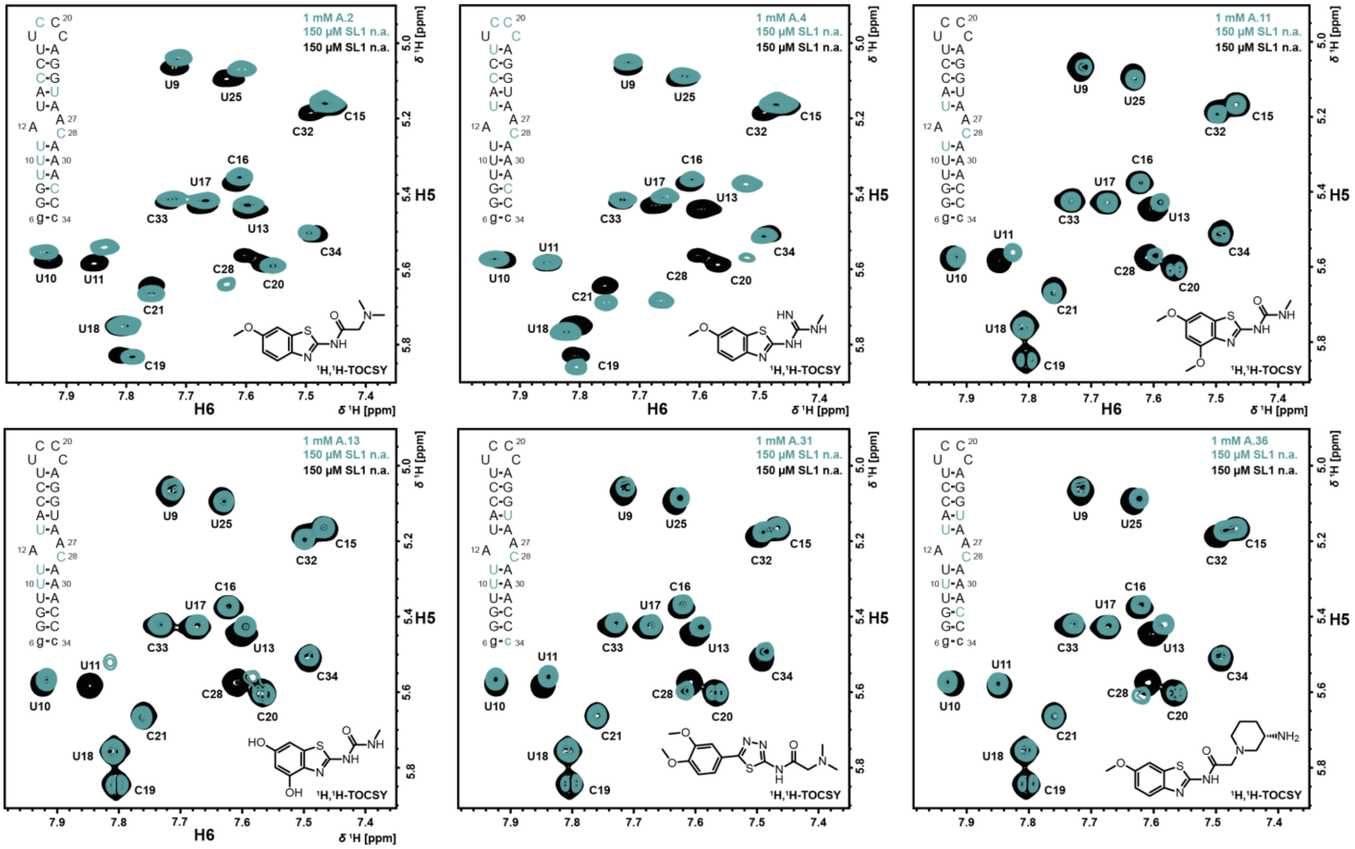

Suppl. Fig. 5. 2D-^{1}H,^{1}H-TOCSY overlay of SL1 in the absence and presence of **A** derivatives. A.2 and A.4 were measured at 600 MHz and 298 K; A.11, A.13, A.31 and A.36 were measured at 700 MHz and 298 K. Experiments were performed with an unlabeled RNA (150 µM) sample and a sample containing both the compound (1 mM) and the unlabeled RNA (150 µM).

Binding affinities

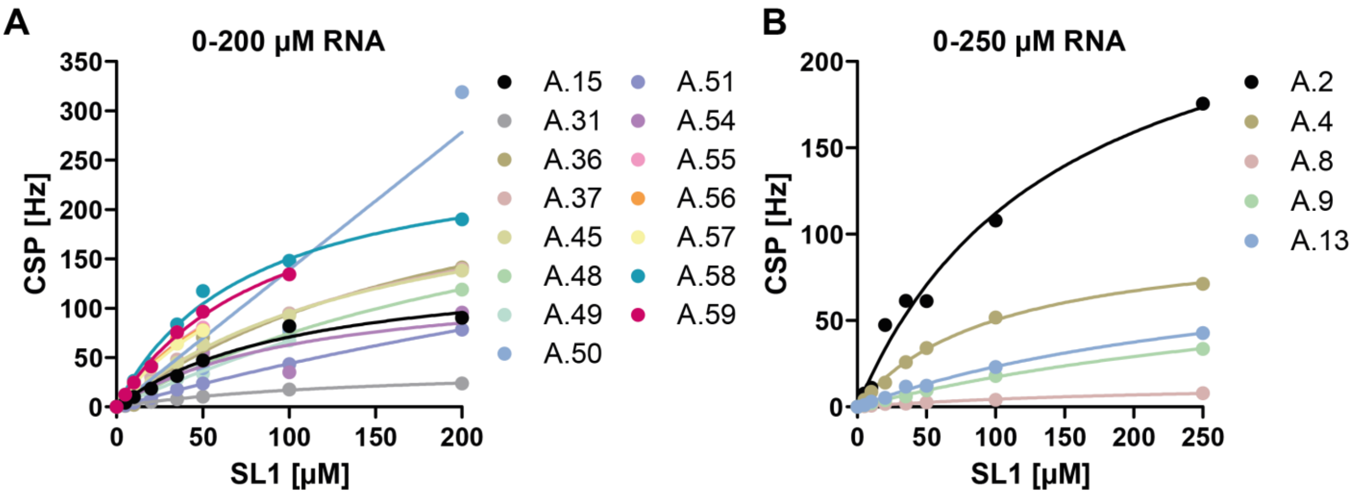

Suppl. Figure 6. Determination of NMR-based, ligand-detected binding affinities of the lead **A** derivatives. The ligand concentration was kept constant at 100 µM while the RNA concentration increased from 0 to 250 µM in eight individual samples. ^{1}H NMR spectra were recorded at 600 MHz and 298 K; however, not every ligand signal could be traced throughout the complete set of spectra due to overlap with the RNA signal (see Suppl. Table 7). The estimated binding affinities are shown in Suppl. Table 7.

2-Aminopurine in vitro assay for the determination of binding affinities

For NMR-complementary studies, binding affinities were further determined using a fluorescence-based technique as previously published in Toews, Wacker *et al.*[1] Briefly, a 2-aminopurine (2AP) fluorescence assay was employed using SL1 RNA modified at position 27, where adenine was substituted with 2AP. The RNA was synthesized by Horizon Discovery and exchanged into the NMR buffer (25 mM KP_i pH 6.2, 50 mM KCl). Binding was assessed by monitoring changes in intrinsic 2AP fluorescence upon titration with increasing concentrations of test compounds (0-400 µM), while maintaining a constant RNA concentration (0.5 µM). Samples (30 µL) were incubated for 30 minutes at 25 °C and prepared in triplicates, which were transferred to black 384-well plates. Fluorescence emission at 372 nm (excitation at 308 nm, 10 nm bandwidth) was measured using a Tecan Spark® plate reader. Binding affinities were calculated by fitting the data in GraphPad Prism 5.0 using Equation [4] (main text).

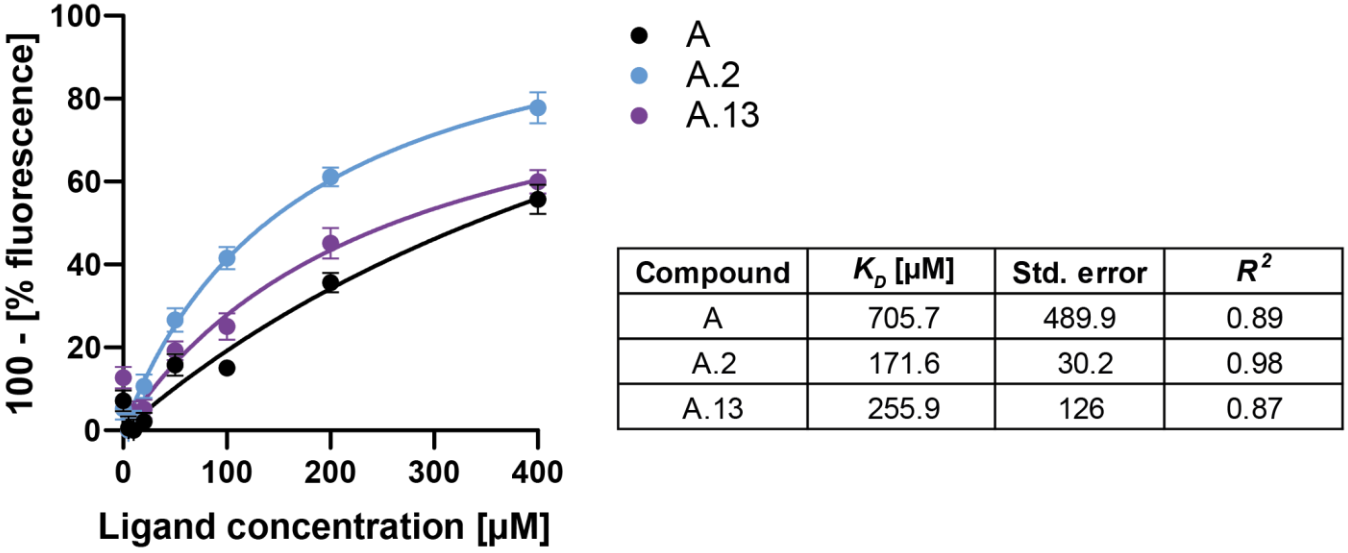

Compound	K_D [µM]	Std. error	R^2
A	705.7	489.9	0.89
A.2	171.6	30.2	0.98
A.13	255.9	126	0.87

Suppl. Figure 7. Fluorescence-based determination of RNA-binding affinities for lead **A** and its derivatives A.2 and A.13. Binding was assessed using SL1 RNA labeled with 2AP at a constant concentration of 0.5 µM, while compound concentrations ranged from 0 to 400 µM across eight individual samples. Fluorescence was measured at 372 nm (excitation at 308 nm) at 25 °C and normalized to the RNA-only control. Plotted is the inverse of the fluorescence decrease observed upon compound binding. Error bars represent the standard error of the mean (SEM) resulting from triplicate measurements.

Cell-free translation assay – time-resolved control experiment

Translation samples were prepared according to the protocol for the translation assay performed with pre-incubation and measured at a certain time point after incubation, as presented in the methods section. For the time-resolved experiments, the protocol was modified to include the addition of the Nano-Glo Luciferase substrate furimazine at a final concentration of 1% (v/v) from the outset, without prior dilution in Nano-Glo buffer (Promega). Measurements were started immediately after the samples were transferred to 384-well round-bottom white polystyrene microplates (Corning). Measurements were carried out at the same wavelengths and temperature as previously described, but repeated every ~4.5 min. Measurements were performed in technical and biological triplicates.

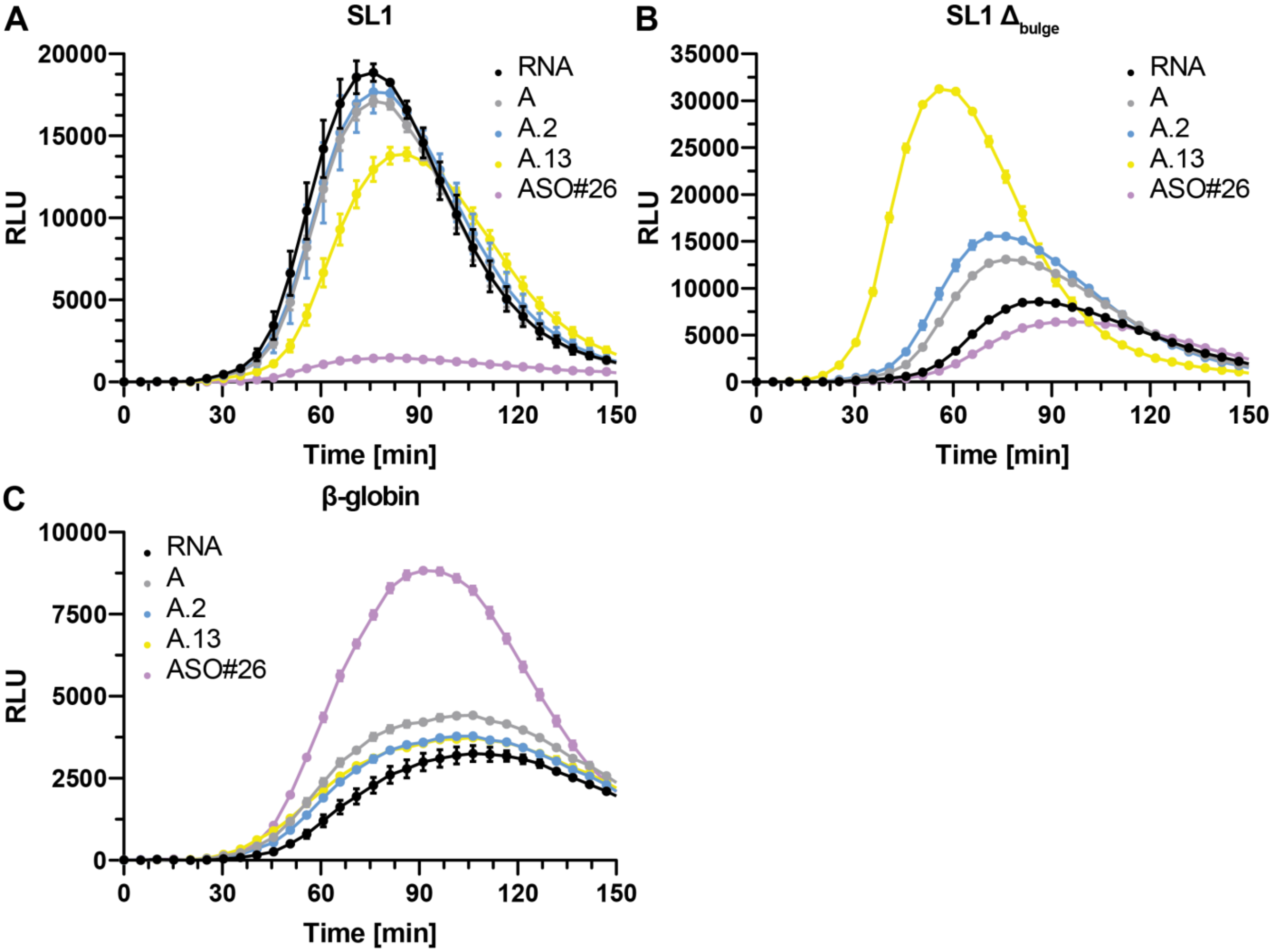

Suppl. Figure 8. Luminescence-based measurements of the translation of viral (A-B) and host (C) mRNAs in the presence of the lead **A** and its derivatives A.2 and A.13. Error bars represent the SEM. The samples were measured at a [RNA]:[ligand] ratio of 1:10, with 0.2 µM of the target mRNA and 2 µM of the respective compound.

REFERENCES

(1) Toews, S.; Wacker, A.; Faison, E. M.; Duchardt-Ferner, E.; Richter, C.; Mathieu, D.; Bottaro, S.; Zhang, Q.; Schwalbe, H. The 5′-Terminal Stem-Loop RNA Element of SARS-CoV-2 Features Highly Dynamic Structural Elements That Are Sensitive to Differences in Cellular PH. *Nucleic Acids Res.* **2024**, *52* (13), 7971–7986. https://doi.org/10.1093/nar/gkae477.
