# Supplementary material for: Targeting the SARS-CoV‑2 RNA Translation Initiation Element SL1 by Molecules of Low Molecular Weight
Source: J Am Chem Soc. 2025 Aug 4;147(32):28783–98. doi: 10.1021/jacs.5c05264 (PMC12356538; doi:10.1021/jacs.5c05264)
Supplement: Supplementary file 2 [file ja5c05264_si_002.pdf]

Targeting the SARS-CoV-2 RNA translation initiation element SL1 by molecules of low molecular weight

Sabrina Toews[1], Francesca Donà[2], Jürgen Krauß[2], Franz Bracher[2], Úrsula López-García[3], Jörg Pabel[3], Daniel Merk[3], Marcel J.J Blommers[4], Anna Wacker[1], Jan Ferner[1], Christian Richter[1] and Harald Schwalbe[1,]*

[1]Institute for Organic Chemistry and Chemical Biology and Center for Biomolecular Magnetic Resonance (BMRZ), Goethe University Frankfurt am Main, Frankfurt/Main, Hesse 60438, Germany

[2]Pharmaceutical Chemistry, Department of Pharmacy, Center for Drug Research, Ludwig-Maximilians-University Munich, Munich, Bavaria 81377, Germany

[3]Department of Pharmacy, Ludwig-Maximilians-University Munich, Munich, Bavaria 81377, Germany

[4]Saverna Therapeutics, Biel-Benken, 4105, Switzerland

*To whom correspondence should be addressed. Tel: +49 (0)69 / 798-29737; Fax: +49 (0)69 / 798-29515; Email: schwalbe@nmr.uni-frankfurt.de

KEYWORDS: NMR spectroscopy, RNA, SARS-CoV-2, Screening, Drug design

Table of Contents

Chemical methods and analytical data 2

General procedures 3

- General procedure A: Thiourea synthesis. 3
- General procedure B: N-Methylurea synthesis. 3
- General procedure C: O-Demethylation of methoxybenzothiazoles 3
- General procedure D: N-Acetylation of 2-aminobenzothiazoles. 3
- General procedure E: Amide synthesis from 2-aminobenzothiazoles. 3
- General procedure F: Nucleophilic substitution of alkyl chlorides with amines. 4
- General procedure G: Reduction of nitroarenes to primary amines. 4
- General procedure H: Boc deprotection. 4
- General procedure I: Reductive alkylation of primary amines with NaBH3CN. 4
- General procedure J: N-Alkylation of amino phenols with alkyl iodides. 4
- General procedure K: Nosyl protection of phenols. 4
- General procedure L: Acetal deprotection. 4
- General procedure M: Reductive alkylation of primary amines with NaBH(OAc)3. 5
- General procedure N: Nosyl deprotection. 5

Precursor synthesis 5

Depicted product synthesis 15

References 75

SUPPLEMENTARY INFORMATION II - Experimental section

Chemical methods and analytical data

All chemicals were purchased from Thermo Fisher Scientific (Schwerte, Germany), TCI (Eschborn, Germany), Sigma-Aldrich (now Merck, Darmstadt, Germany), Th. Geyer (Renningen, Germany), or abcr (Karlsruhe, Germany) and were of analytical grade. Solvents, dry and of HPLC grade, were purchased from VWR (Darmstadt, Germany) or Sigma-Aldrich. All other solvents were purified by distillation. Thin layer chromatography (TLC) using polyester sheets POLYGRAM® SIL G/UV 254, coated with 0.2 mm silica gel, from Macherey-Nagel (Düren, Germany) were used to monitor the progress of the conversion and UV light (254 nm/365 nm) was used to visualize the compounds. Flash column chromatography (FCC) was performed using silica gel 60 (0.040 - 0.063 mm) from Macherey-Nagel. The fractions containing the final compound were unified and the volatiles were removed under reduced pressure, using Laborota 4001-efficient from Heidolph Instrument (Schwabach, Germany) rotary evaporators and membrane vacuum pumps MD 4C 2006 from VACUUBRAND GMBH (Wertheim, Germany). High resolution mass spectra (HRMS) were performed by the Department Chemistry and Pharmacy of the Ludwig-Maximilians-University of Munich applying a Thermo Finnigan MAT 95 or Joel MStation Sektorfeld instrument at a core temperature of 250 °C and 70 eV for EI or a Thermo Finnigan LTQ FT Ultra Fourier Transform Ion Cyclotron Resonance device at 250 °C for ESI. NMR spectra (^{1}H, ^{13}C, DEPT, H-H-COSY, HMQC/HSQC, HMBC) were recorded at 23 °C on Avance III HD 400 MHz Bruker BioSpin. All ^{1}H NMR-spectra and ^{13}C NMR-spectra were recorded respectively at 400 MHz and 101 MHz, using $CDCl_3$, DMSO-d_6, or CD_3OD as solvents, whose δ value of peaks were taken as internal reference for chemical shifts. Chemical shifts δ are reported in parts per million (ppm) relative to tetramethylsilane. ^{1}H NMR data are reported as follows: chemical shift δ (multiplicity, coupling constants J, integral). The following abbreviations were used for signal multiplicity: s (singlet), d (doublet), dd (double doublet), ddd (double double doublet), dt (double triplet), t (triplet), q (quartet), m (multiplet), br (broad). The coupling constants J are reported in Hertz (Hz). ^{13}C NMR-data are reported as chemical shift δ. NMR spectra were analysed by the Software MestReNova 15.0.0-34764 (Mestrelab Research S.L.). Melting points (mp) were measured on Büchi melting point B-540 apparatus, reported in °C and not corrected. Values for specific rotation $[\alpha]_D^{20}$ were measured at a wavelength of λ = 589 nm (Na-D-line) at 20 °C using a Perkin Elmer 241 Polarimeter instrument (layer thickness l = 10 cm). All samples were dissolved in MeOH and the concentration is stated in g/100 mL. HPLC analytical measurements for determination of the purities of the final products were carried out detecting at 210 nm and 254 nm, using the following Methods:

Method 1a: column Xbridge Phenyl, 3.5 µm (4.6 x 150mm), 10 µL injection volume, 35 °C, 0.8 mL/min flow rate, 30:70 → 70:30 MeOH/phosphate buffer pH 9.0 as eluent.

Method 1b: column Xbridge Phenyl, 3.5 µm (4.6 x 150mm), 5 µL injection volume, 35 °C, 0.8 mL/min flow rate, 20:80 → 90:10 MeOH/phosphate buffer pH 9.0 as eluent.

Method 1c: column Xbridge Phenyl, 3.5 µm (4.6 x 150mm), 10 µL injection volume, 35 °C, 0.8 mL/min flow rate, 70:30 MeOH/phosphate buffer pH 9.0 as eluent.

Method 2a: column Zorbax Eclipse Plus, C18 5 µm (4.6 x 150 mm), 10 µL injection volume, 50 °C, 1.5 mL/min flow rate, 70:30 MeOH/water as eluent.

Method 2b: column Zorbax Eclipse Plus, C18 5 µm (4.6 x 150 mm), 10 µL injection volume, 35 °C, 1.2 mL/min flow rate, 65:35 MeOH/water as eluent.

Method 3a: column Xbridge Phenyl, 3.5 µm (4.6 x 150mm), 2 µL injection volume, 35 °C, 0.6 mL/min flow rate, 30:70 acetonitrile/0.1% TFA in water as eluent.

Method 3b: column Xbridge Phenyl, 3.5 µm (4.6 x 150mm), 5 µL injection volume, 35 °C, 0.8 mL/min flow rate, 15:85 acetonitrile/0.1% TFA in water as eluent.

Method 3c: column Xbridge Phenyl, 3.5 µm (4.6 x 150mm), 2 µL injection volume, 35 °C, 0.6 mL/min flow rate, 20:80 → 80:20 MeOH/0.1% TFA in water as eluent.

Method 3d: column Xbridge Phenyl, 3.5 µm (4.6 x 150mm), 10 µL injection volume, 35 °C, 0.8 mL/min flow rate, 70:30 MeOH/0.1% TFA in water as eluent.

Method 3e: column Xbridge Phenyl, 3.5 µm (4.6 x 150mm), 3 µL injection volume, 35 °C, 0.6 mL/min flow rate, 65:35 MeOH/0.1% TFA in water as eluent.

Method 4: column Zorbax Eclipse Plus, C18 5 µm (4.6 x 150 mm), 10 µL injection volume, 50 °C, 1.5 mL/min flow rate, 5:95 → 0:100 acetonitrile/ion pair reagent pH 3.5 as eluent.

General procedures

General procedure A: Thiourea synthesis.
The respective amine (1.0 eq), methyl isothiocyanate (2.0 eq), and triethylamine (2.0 eq) were dissolved in toluene (2.5 mL/mmol amine) and the solution was stirred at 90 °C for 3 – 18 h. The mixture was cooled to room temperature. The formed precipitate was filtered off, washed with DCM and discarded. The filtrate was evaporated to dryness to give the respective products.

General procedure B: N-Methylurea synthesis.
Methylaminoformyl chloride (1.0 eq) was added portion wise at 5 °C to pyridine (0.3 mL/mmol methylaminoformyl chloride) under nitrogen atmosphere. After portion wise addition of the respective amine (1.0 eq), the mixture was stirred at 50 °C for 2 h. After cooling to room temperature, water was slowly added to the mixture, which was then diluted with DCM. The precipitate was collected by filtration with suction and washed with DCM and water. The solid was dried under reduced pressure to give the final product.

General procedure C: O-Demethylation of methoxybenzothiazoles.
Boron tribromide (11 eq) was added dropwise to a suspension of the corresponding methoxybenzothiazole (1.0 eq) in DCM (15 mL/mmol methoxybenzothiazole) at 0 °C. The mixture was allowed to warm up to room temperature and stirred for 16 h. The mixture was then cooled to 0 °C and quenched with methanol (2.5 mL/mmol benzothiazole). The mixture was neutralised through the addition of aq. sat. $NaHCO_3$ solution. The formed precipitate was filtered off and discarded. The aqueous and organic phase of the filtrate were separated. The aq. phase was extracted with EtOAc (3 x 50 mL). All organic layers were combined, dried over Na_2SO_4 and the solvent was removed under reduced pressure. The residue was treated with DCM and the formed precipitate was collected, washed with DCM and dried under reduced pressure. If required, the product was further purified by flash column chromatography.

General procedure D: N-Acetylation of 2-aminobenzothiazoles.
The corresponding 2-aminobenzothiazole (1.0 eq) was dissolved in DCM (9.0 mL/mmol 2-aminobenzothiazole) under nitrogen atmosphere. Subsequently, triethylamine (2.0 eq) and Ac_2O (1.5 eq) were added dropwise at 0 °C. The mixture was stirred at room temperature for 18 h. Water was added and the mixture was extracted with DCM (3 x). The organic layers were combined, dried over Na_2SO_4 and the solvent was removed under reduced pressure. The crude product was purified by recrystallization or flash column chromatography.

General procedure E: Amide synthesis from 2-aminobenzothiazoles.
The corresponding 2-aminobenzothiazole (1.0 eq) was dissolved in DCM (9.0 mL/mmol 2-aminobenzothiazole) or THF (4.5 mL/mmol 2-aminobenzothiazole) under nitrogen atmosphere. Subsequently, triethylamine (1.1 eq) and the corresponding acyl chloride (1.1 eq) were added dropwise at 0 °C. The mixture was stirred at room temperature for 1 – 72 h. The solvent was removed under reduced pressure and the residue taken up in water. The precipitate of the resulting suspension was filtered off, washed with water and the filtrate discarded. If the filtration from water was inconvenient, the aq. suspension was extracted with EtOAc (3 x). The organic layers were combined, dried over Na_2SO_4 and the solvent was removed under reduced pressure. The crude product was purified by crystallization from EtOH or flash column chromatography.

General procedure F: Nucleophilic substitution of alkyl chlorides with amines.
The corresponding alkyl chloride (1.0 eq) was dissolved in DMF (1.4 mL/mmol alkyl chloride). The corresponding amine (2.2 eq) or the corresponding amine (1.1 eq) and triethylamine (1.1 eq), with respect to the availability of the amines, were added to the solution at room temperature and the mixture was stirred for 1 – 24 h. The reaction was quenched by addition of water and the mixture was extracted with a suitable organic solvent (3 x). The organic layers were combined, dried over Na_2SO_4 and the solvent was removed under reduced pressure. The crude product was purified by filtration, crystallization, or flash column chromatography.

General procedure G: Reduction of nitroarenes to primary amines.
To a solution of the corresponding nitroarene (1.0 eq) in MeOH (50 mL/mmol nitroarene) was added palladium on charcoal (0.05 eq, 10 wt.%) and one drop of AcOH. The mixture was hydrogenated at atmospheric pressure for 1 – 3 h. Then, the catalyst was removed by filtration through a celite pad and the pad washed with methanol. The filtrate was evaporated under reduced pressure and the residue was further purified by flash column chromatography.

General procedure H: Boc deprotection.
The corresponding *tert*-butyl carbamate (1.0 eq) was dissolved in DCM (10 mL/mmol carbamate) and TFA (10 mL/mmol carbamate) was added at 0 °C. The mixture was stirred for 1 h and then concentrated under reduced pressure. The excess of TFA was neutralised with aq. sat. $NaHCO_3$ solution and the mixture was extracted with EtOAc (3 x), DCM (3 x) or $CHCl_3$/isopropanol (3:1, 3 x). The organic layers were combined, dried over Na_2SO_4 and the solvent was removed under reduced pressure. If required, the crude product was purified by flash column chromatography.

General procedure I: Reductive alkylation of primary amines with NaBH3CN.
Amine **A.36** or **A.37** (160 mg, 0.500 mmol, 1.0 eq) and 3-hydroxybenzaldehyde (123 mg, 1.00 mmol, 2.0 eq) were suspended in 2 mL MeOH and the suspension was stirred at room temperature under nitrogen atmosphere. After 1 h stirring, $NaBH_3CN$ (66 mg, 1.0 mml, 2.0 eq) was added at 0 °C and the mixture was stirred for 1 h. Aq. sat. $NaHCO_3$ solution (20 mL) was added and the mixture was extracted with $CHCl_3$/isopropanol (3:1, 3 x 20 mL). The organic layers were combined, dried over Na_2SO_4 and the solvent was removed under reduced pressure. The crude product was purified by flash column chromatography (96:3:1 DCM/MeOH/25% NH_3 aq. solution).

General procedure J: N-Alkylation of amino phenols with alkyl iodides.
The corresponding alkyl iodide (2.0 eq) was added to a suspension of 3-((2,2-diethoxyethyl)amino)phenol (1.0 eq) and Na_2CO_3 (2.0 eq) in ACN (10 mL/mmol aminophenol) and the mixture was stirred at 80 °C for 4 h. Water was added and the mixture was extracted with EtOAc (3 x). The organic layers were combined, dried over Na_2SO_4 and the solvent was removed under reduced pressure. The crude product was purified by flash column chromatography (1:4 EtOAc/isohexanes).

General procedure K: Nosyl protection of phenols.
The corresponding phenol (1.0 eq) was dissolved in ACN (2.3 mL/mmol phenol). 2-Nosyl chloride (1.5 eq) and triethylamine (2.0 eq) were added at 0 °C and the mixture was stirred for 1 h. The reaction was quenched with HCl (2 M aq. solution), the mixture was diluted with water and extracted with DCM (3 x). The organic layers were combined, dried over Na_2SO_4 and the solvent was removed under reduced pressure. The crude product was purified by flash column chromatography (1:3 EtOAc/isohexanes).

General procedure L: Acetal deprotection.
The corresponding acetal (1.0 eq) was dissolved in 1,4-dioxane (5.0 mL/mmol acetal) and HCl (3.0 eq, 37% aq. solution) was added. The mixture was stirred at 40 °C for 1 h. The mixture was cooled to 0 °C, neutralised with aq. sat. $NaHCO_3$ solution and the mixture was extracted with EtOAc (3 x). The organic layers were combined, dried over Na_2SO_4 and the solvent was removed under reduced pressure. The crude product was purified by flash column chromatography (2:1 EtOAc/isohexanes).

General procedure M: Reductive alkylation of primary amines with NaBH(OAc)3.

The corresponding aldehyde (1.0 eq), the corresponding amine (1.0 eq) and 3 Å molecular sieve (0.05 g/10 mL solvent) were suspended in anhydrous 1,2-dichloroethane (20 mL/mmol aldehyde) under nitrogen atmosphere. $NaBH(OAc)_3$ (1.4 eq) was added and the mixture was stirred at room temperature for 3 h. After filtration on celite and washing with $CHCl_3$/isopropanol (3:1), the filtrate was washed with NaOH (1 M aq. solution). The separated aq. phase was extracted with $CHCl_3$/isopropanol (3:1, 3 x). The organic layers were combined, dried over Na_2SO_4 and the solvent was removed under reduced pressure. The crude product was purified by flash column chromatography (97:2:1 DCM/MeOH/25% NH_3 aq. solution).

General procedure N: Nosyl deprotection.

The corresponding nosyl-phenol (1.0 eq) and Cs_2CO_3 (1.5 eq) were dissolved in DMF (10 mL/mmol nosyl-phenol) under nitrogen atmosphere and benzenethiol (2.5 eq) was added. The mixture was stirred at room temperature for 1 h. Water was added and the mixture was extracted with $CHCl_3$/isopropanol (3:1, 3 x). The organic layers were combined, dried over Na_2SO_4 and the solvent was removed under reduced pressure. The crude product was purified by flash column chromatography (96:3:1 DCM/MeOH/25% NH_3 aq. solution).

Precursor synthesis

1-(6-Methoxybenzo[*d*]thiazol-2-yl)-3-methylthiourea (1)

Thiourea **1** was prepared following General procedure A, using 2-amino-6-methoxybenzothiazole (251 mg, 1.35 mmol, 1.0 eq). The mixture was stirred for 18 h to give thiourea **1**(65 mg, 0.26 mmol, 19%) as an off-white solid. Mp: 213 °C (decomposition). ^1H NMR (400 MHz, DMSO) δ 11.76 (s, 1H), 9.93 (s, 1H), 7.57 (d, *J* = 8.5 Hz, 1H), 7.51 (d, *J* = 2.6 Hz, 1H), 7.00 (dd, *J* = 8.8, 2.6 Hz, 1H), 3.79 (s, 3H), 3.07 (d, *J* = 4.4 Hz, 3H). ^{13}C NMR (101 MHz, DMSO) δ 179.4, 156.1, 142.7, 131.0, 130.9, 120.0, 114.5, 105.3, 55.7, 31.5. HRMS (ESI): m/z = calculated for $C_{10}H_{10}N_3OS_2$ $[M-H]^-$: 252.0270; found: 252.0273. Purity (HPLC): 100% (λ = 210 nm), 100% (λ = 254 nm), Method 2a.

2-Chloro-*N*-(6-methoxybenzo[*d*]thiazol-2-yl)acetamide (2)

Chloroacetamide **2** was prepared as described in Sağlık *et al.* from 2-amino-6-methoxybenzothiazole (5.0 g, 27 mmol, 1.0 eq), using 2-chloroacetyl chloride (2.35 mL, 29.6 mmol, 1.1 eq) and triethylamine (4.13 mL, 29.6 mmol, 1.1 eq).[1] Light brown solid (6.08 g, 23.7 mmol, 88%). Mp: 172 – 173 °C [169 – 172 °C[2]]. ^1H NMR (400 MHz, DMSO) δ 12.58 (s, 1H), 7.66 (d, *J* = 8.8 Hz, 1H), 7.59 (d, *J* = 2.6 Hz, 1H), 7.04 (dd, *J* = 8.8, 2.6 Hz, 1H), 4.44 (s, 2H), 3.81 (s, 3H). ^{13}C NMR (101 MHz, DMSO) δ 166.1, 156.8, 155.9, 143.0, 133.3, 121.8, 115.6, 105.3, 56.1, 43.0. HRMS (ESI): m/z = calculated for $C_{10}H_{10}N_2O_2S$ $[M+H]^+$: 257.0147; found: 257.0148.

4,6-Dimethoxybenzo[*d*]thiazol-2-amine (3)

Aminobenzothiazole **3** was prepared as described in Hirose *et al.* from 2,4-dimethoxyaniline (2.0 g, 13 mmol, 1.0 eq), using ammonium thiocyanate (1.74 g, 22.8 mmol, 1.8 eq) and bromine (0.779 mL, 15.2 mmol, 1.2 eq).[3] Colourless solid (1.31 g, 6.22 mmol, 49%). Mp: 177 – 178 °C. ^1H NMR (400 MHz, $CDCl_3$) δ 6.71 (d, *J* = 2.3 Hz, 1H), 6.45 (d, *J* = 2.3 Hz, 1H), 5.16 (s, 2H), 3.93 (s, 3H), 3.81 (s, 3H). ^{13}C NMR (101 MHz, $CDCl_3$) δ 163.1, 156.7, 151.3, 135.9, 133.1, 97.5, 96.1, 56.0, 55.9. HRMS (ESI): m/z = calculated for $C_9H_{11}N_2O_2S$ $[M+H]^+$: 211.0536; found: 211.0534. Purity (HPLC): 100% (λ = 210 nm), 100% (λ = 254 nm), Method 1a.

N-(6-Bromobenzo[*d*]thiazol-2-yl)-2-chloroacetamide (4)

Chloroacetamide **4** was prepared from 2-amino-6-bromobenzothiazole (1.25 g, 5.46 mmol, 1.0 eq), using 2-chloroacetyl chloride (0.477 mL , 6.00 mmol, 1.1 eq) and triethylamine (0.837 mL, 6.00 mmol, 1.1 eq). Old pink solid (1.34 g, 4.40 mmol, 81%). Mp: 224 – 226 °C [210 – 211 °C[4]]. ^1H NMR (400 MHz, DMSO) δ 12.81 (s, 1H), 8.28 (d, *J* = 2.0 Hz, 1H), 7.71 (d, *J* = 8.6 Hz, 1H), 7.59 (dd, *J* = 8.6, 2.1 Hz, 1H), 4.47 (s, 2H). ^{13}C NMR (101 MHz, DMSO) δ 165.4, 157.6, 146.9, 132.9, 128.5, 123.6, 121.6, 115.0, 41.7. HRMS (ESI): m/z = calculated for $C_9H_7N_2O_2S^{79}BrCl$ $[M+H]^+$: 304.9146; found: 304.9146.

2-Chloro-*N*-(6-nitrobenzo[*d*]thiazol-2-yl)acetamide (5)

Chloroacetamide **5** was prepared as described in Sağlık *et al.* from 2-amino-6-nitrobenzothiazole (5.0 g, 26 mmol, 1.0 eq), using 2-chloroacetyl chloride (2.24 mL, 28.2 mmol, 1.1 eq) and triethylamine (3.93 mL, 28.2 mmol, 1.1 eq).[1] Beige solid (6.39 g, 23.5 mmol, 92%). Mp: 218 °C (decomposition) [188 – 189 °C[4]]. ^{1}H NMR (400 MHz, DMSO) δ 13.12 (s, 1H), 9.07 (d, *J* = 2.4 Hz, 1H), 8.28 (dd, *J* = 8.9, 2.4 Hz, 1H), 7.92 (d, *J* = 9.0 Hz, 1H), 4.52 (s, 2H). ^{13}C NMR (101 MHz, DMSO) δ 166.7, 163.1, 153.3, 143.2, 132.2, 121.9, 120.9, 119.2, 42.6. HRMS (ESI): m/z = calculated for $C_9H_5N_3O_2SCl$ $[M-H]^-$: 269.9745; found: 269.9746.

2-Chloro-*N*-(4,6-dimethoxybenzo[*d*]thiazol-2-yl)acetamide (6)

Chloroacetamide **6** was prepared following General procedure E, using 2-aminobenzothizole **3** (683 mg, 3.25 mmol, 1.0 eq) and 2-chloroacetyl chloride in THF for 16 h. Filtration and recrystallization gave product **6** (823 mg, 2.87 mmol, 89%) as an off-white solid. Mp: 218 – 219 °C. ^{1}H NMR (400 MHz, DMSO) δ 12.70 (s, 1H), 7.14 (d, *J* = 2.3 Hz, 1H), 6.60 (d, *J* = 2.3 Hz, 1H), 4.41 (s, 2H), 3.88 (s, 3H), 3.80 (s, 3H). ^{13}C NMR (101 MHz, DMSO) δ 165.4, 157.3, 152.2, 133.5, 132.7, 98.2, 95.8, 61.2, 55.9, 55.7, 42.4. HRMS (EI): m/z = calculated for $C_{11}H_{11}N_2O_3SCl$ $[M]^{\cdot+}$: 286.0174; found: 286.0171.

***N*-(Benzo[*d*]thiazol-2-ylmethyl)-2-chloroacetamide (7)**

Chloroacetamide **7** was prepared following General procedure E, using benzo[*d*]thiazol-2-ylmethanamine (100 mg, 0.578 mmol, 1.0 eq) and 2-chloroacetyl chloride in THF for 1 h. Filtration and recrystallization gave product **7** (127 mg, 0.526 mmol, 91%) as a light brown solid. Mp: 125 – 126 °C. ^{1}H NMR (500 MHz, $CDCl_3$) δ 8.02 (dd, *J* = 8.3, 1.4 Hz, 1H), 7.87 (dd, *J* = 8.0, 1.3 Hz, 1H), 7.55 (s, 1H), 7.50 (ddd, *J* = 8.3, 7.3, 1.3 Hz, 1H), 7.41 (ddd, *J* = 8.2, 7.2, 1.2 Hz, 1H), 4.93 (d, *J* = 5.7 Hz, 2H), 4.16 (s, 2H), 2.70 (s, 1H). ^{13}C NMR (126 MHz, $CDCl_3$) δ 167.1, 166.4, 152.4, 135.1, 126.6, 125.7, 123.1, 122.0, 42.6, 42.0. HRMS (EI): m/z = calculated for $C_{10}H_9ClN_2OS$ $[M]^{\cdot+}$: 240.0119; found: 240.0117.

2-Chloro-*N*-(6-methoxy-1*H*-benzo[*d*]imidazol-2-yl)acetamide (8)

Chloroacetamide **8** was prepared following General procedure E, using 5-methoxy-1*H*-benzimidazol-2-ylamine (490 mg, 3.00 mmol, 1.0 eq) and 2-chloroacetyl chloride in THF for 2 h. After extraction, the residue was purified by flash column chromatography (98:2 DCM/MeOH) to give product **8** (394 mg, 1.46 mmol, 49%) as a beige solid. Mp: 170 °C (decomposition), 224 – 225 °C (melting). ^{1}H NMR (500 MHz, DMSO) δ 11.96 (s, 2H), 7.32 (d, *J* = 8.6 Hz, 1H), 6.99 (d, *J* = 2.4 Hz, 1H), 6.74 (dd, *J* = 8.7, 2.5 Hz, 1H), 4.35 (s, 2H), 3.75 (s, 3H). ^{13}C NMR (126 MHz, DMSO) δ 167.0, 155.3, 146.7, 114.2, 110.1, 97.8, 55.4, 43.4. HRMS (ESI): m/z = calculated for $C_{10}H_{11}N_3O_2S$ $[M+H]^+$: 240.0535; found: 240.0534.

6-(Trifluoromethyl)benzo[*d*]thiazol-2-amine (9)

Aminobenzothiazole **9** was prepared as described in Potopnyk *et al.* from 4-(trifluoromethyl)aniline (200 mg, 1.24 mmol, 1.0 eq), using ammonium thiocyanate (189 mg, 2.48 mmol, 2.0 eq) and bromine (0.127 mL, 2.48 mmol, 2.0 eq).[5] Bright yellow solid (100 mg, 0.458 mmol, 37%). Mp: 117 – 119 °C [113.7 – 115.5 °C[5]]. ^{1}H NMR (400 MHz, $CDCl_3$) δ 7.86 (dt, *J* = 1.7, 0.7 Hz, 1H), 7.62 – 7.52 (m, 2H), 5.59 (s br, 2H). ^{13}C NMR (101 MHz, $CDCl_3$) δ 167.9, 154.7, 131.9, 125.9, 124.8, 124.5, 124.2, 123.5 (q, *J* = 3.8 Hz), 123.2, 119.2, 118.6 (q, *J* = 4.2 Hz). HRMS (ESI): m/z = calculated for $C_8H_9N_2S_2$ $[M+H]^+$: 197.0202; found: 197.0202.

6-(Methylthio)benzo[*d*]thiazol-2-amine (10)

Aminobenzothiazole **10** was prepared following the same procedure described for aminobenzothiazole **9**[5], from 4-(methylthio)aniline (200 mg, 1.44 mmol, 1.0 eq), using ammonium thiocyanate (219 mg, 2.87 mmol, 2.0 eq) and bromine (0.147 mL, 2.87 mmol, 2.0 eq). Grey solid (93 mg, 0.46 mmol, 33%). Mp: 141 – 143 °C [148 – 149 °C[6]]. ^{1}H NMR (500 MHz, $CDCl_3$) δ 7.52 (d, *J* = 1.9 Hz, 1H), 7.45 (d, *J* = 8.4 Hz, 1H), 7.28 (dd, *J* = 8.4, 1.9 Hz, 1H), 2.50 (s, 3H). ^{13}C NMR (126 MHz, $CDCl_3$) δ 165.78, 149.4, 132.2, 132.0, 126.9, 120.5, 119.3, 17.8. HRMS (ESI): m/z = calculated for $C_8H_9N_2S_2$ $[M+H]^+$: 197.0202; found: 197.0202.

2-Chloro-*N*-(6-(trifluoromethyl)benzo[*d*]thiazol-2-yl)acetamide (11)

Chloroacetamide **11** was prepared following General procedure E, using 2-aminobenzothiazole **9** (100 mg, 0.458 mmol, 1.0 eq) and 2-chloroacetyl chloride in THF for 3 h. Filtration and recrystallization gave product **11** (102 mg, 0.347 mmol, 76%) as a light yellow solid. ^{1}H and ^{13}C NMR data is in accordance with literature.[7] Mp: 180 – 181 °C [179 – 180 °C[7]]. ^{1}H NMR (400 MHz, $CDCl_3$) δ 8.13 (dt, *J* = 1.4, 0.8 Hz, 1H), 7.90 (dq, *J* = 8.6, 0.7 Hz, 1H), 7.75 – 7.68 (m, 1H), 4.35 (s, 2H). ^{13}C NMR (101 MHz, $CDCl_3$) δ 164.9, 159.1, 150.7, 132.5, 127.0, 126.7, 125.7, 123.8 (q, *J* = 3.6 Hz), 123.0, 121.8, 119.4 (q, *J* = 4.2 Hz), 42.2. HRMS (EI): *m/z* = calculated for $C_8H_6ClF_3N_2OS$ $[M]^{\cdot+}$: 293.9836; found: 293.9835.

2-Chloro-*N*-(6-(methylthio)benzo[*d*]thiazol-2-yl)acetamide (12)

Chloroacetamide **12** was prepared following General procedure E, using 2-aminobenzothizole **10** (88 mg, 0.45 mmol, 1.0 eq) and 2-chloroacetyl chloride in THF for 3 h. Filtration and recrystallization gave product **12** (112 mg, 0.441 mmol, 91%) as a yellow solid. Mp: 155 – 156 °C. ^{1}H NMR (400 MHz, $CDCl_3$) δ 7.71 (dd, *J* = 6.8, 0.5 Hz, 1H), 7.70 (s, 1H), 7.43 – 7.36 (m, 1H), 4.31 (s, 2H), 2.55 (s, 3H). ^{13}C NMR (101 MHz, $CDCl_3$) δ 164.6, 156.2, 146.3, 135.0, 133.4, 126.6, 121.6, 119.4, 42.2, 17.0. HRMS (EI): *m/z* = calculated for $C_{10}H_9ClN_2OS_2$ $[M]^{\cdot+}$: 271.9840; found: 271.9852.

2-Chloro-*N*-(6-(trifluoromethoxy)benzo[*d*]thiazol-2-yl)acetamide (13)

Chloroacetamide **13** was prepared following General procedure E, using 2-amino-6-(trifluoromethoxy)benzothiazole (100 mg, 0.427 mmol, 1.0 eq) and 2-chloroacetyl chloride in THF for 1 h. Filtration and recrystallization gave product **13** (95 mg, 0.30 mmol, 71%) as an off-white solid. Mp: 182 – 183 °C [188 – 189 °C[4]]. ^{1}H NMR (400 MHz, MeOD) δ 7.87 (dd, *J* = 2.3, 1.1 Hz, 1H), 7.81 (d, *J* = 8.9 Hz, 1H), 7.36 (dtd, *J* = 8.9, 1.9, 0.9 Hz, 1H), 4.36 (s, 2H). ^{13}C NMR (101 MHz, MeOD) δ 168.0, 148.9, 146.7, 134.5, 123.3, 123.0, 121.2, 120.8, 115.6, 43.0. HRMS (EI): *m/z* = calculated for $C_{10}H_6ClF_3N_2O_2S$ $[M]^{\cdot+}$: 309.9786; found: 309.9784.

Pyrimidine-5-carbaldehyde (14)

Aldehyde **14** was prepared as described in {Patent WO2020180624A1}, treating pyrimidine-5-methanol (899 mg, 8.00 mmol, 1.0 eq) with MnO_2 (6.95 g, 80.0 mmol, 10 eq). Yellow oil (35 mg, 2.8 mmol, 35%). ^{1}H NMR (400 MHz, $CDCl_3$) δ 10.17 (s, 1H), 9.44 (s, 1H), 9.19 (s, 2H). ^{13}C NMR (100 MHz, DMSO) δ 190.3, 163.1, 158.5, 129.2. HRMS (ESI): m/z = calculated for $C_5H_5N_2O$ $[M+H]^+$: 109.0397; found: 109.0399.

2-Chloro-*N*-(5-(3,4-dimethoxyphenyl)-1,3,4-thiadiazol-2-yl)acetamide (15)

Chloroacetamide **15** was prepared following General procedure E, using 5-(3,4-dimethoxyphenyl)-1,3,4-thiadiazol-2-amine (**xx**, 600 mg, 2.53 mmol, 1.0 eq) and 2-chloroacetyl chloride in THF for 1 h. After extraction, the residue was treated with DCM and the insoluble material was filtered off, washed with DCM and dried under reduced pressure to give **15** (435 mg, 1.39 mmol, 55%) as an off-white solid. Mp: 240 – 242 °C (decomposition and melting). ^{1}H NMR (500 MHz, DMSO) δ 12.99 (s, 1H), 7.51 (d, *J* = 2.1 Hz, 1H), 7.47 (dd, *J* = 8.3, 2.1 Hz, 1H), 7.09 (d, *J* = 8.4 Hz, 1H), 4.46 (s, 2H), 3.86 (s, 3H), 3.83 (s, 3H). ^{13}C NMR (126 MHz, DMSO) δ 165.3, 162.3, 157.5, 151.0, 149.1, 122.6, 120.5, 112.0, 109.3, 55.7, 55.6, 42.3. HRMS (ESI): *m/z* = calculated for $C_{12}H_{13}ClN_3O_3S$ $[M+H]^+$: 314.0361; found: 314.0388.

2-Chloro-*N*-(6-(methylsulfonyl)benzo[*d*]thiazol-2-yl)acetamide (16)

Chloroacetamide **16** was prepared following General procedure E, using 2-amino-6-(methylsulfonyl)benzothiazole (114 mg, 0.550 mmol, 1.0 eq) and 2-chloroacetyl chloride in THF for 1 h. After extraction, the residue was purified by flash column chromatography (1:2 EtOAc/isohexanes) to give product **16** (97 mg, 0.32 mmol, 64%) as a pale yellow solid. Mp: 170 – 171 °C [173 – 174 °C[4]]. ^{1}H NMR (400 MHz, DMSO) δ 13.02 (s, 1H), 8.67 (d, *J* = 1.2 Hz, 1H), 7.96 (d, *J* = 1.6 Hz, 2H), 4.51 (s, 2H), 3.25 (s, 3H). ^{13}C NMR (101 MHz, DMSO) δ 166.5, 161.6, 152.0, 135.7, 132.0, 125.0, 122.3, 121.1, 44.0, 42.6. HRMS (ESI): *m/z* = calculated for $C_{10}H_8N_2O_3S_2Cl$ $[M-H]^-$: 302.9670; found: 302.9670.

N-Methyl-1-(pyrimidin-5-yl)methanamine (17)

Aldehyde **14** (270 mg, 2.50 mmol, 1.0 eq) was dissolved in methylamine solution (4.05 mL, 33% in EtOH, 32.5 mmol, 13 eq). AcOH (0.05 mL) were added and the mixture was stirred at room temperature for 2 h. After cooling with an ice-bath, $NaBH_4$ (189 mg, 5.00 mmol, 2.0 eq) was added and the mixture was stirred for 1 h. Aq. sat. $NaHCO_3$ solution (20 mL) was added and the mixture was extracted with $CHCl_3$/isopropanol (3:1, 4 x 20 mL). The organic layers were combined, dried over Na_2SO_4 and the solvent was removed under reduced pressure to give amine **17** (216 mg, 1.75 mmol, 70%) as a yellow oil. The crude product was used without further purification for the next step. ^{1}H NMR (400 MHz, $CDCl_3$) δ 9.12 (s, 1H), 8.70 (d, J = 0.8 Hz, 2H), 3.78 – 3.75 (m, 2H), 2.46 (s, 4H). ^{13}C NMR (101 MHz, $CDCl_3$) δ 157.9, 156.9, 133.3, 51.1, 36.2. HRMS (EI): *m/z* = calculated for $C_6H_9N_3$ $[M]^{\cdot+}$: 123.0791; found: 123.0789.

***tert*-Butyl (*S*)-(1-(2-((5-(3,4-dimethoxyphenyl)-1,3,4-thiadiazol-2-yl)amino)-2-oxoethyl)piperidin-3-yl)carbamate (18)**

Piperidinecarbamate **18** was prepared following General procedure F, using alkyl chloride **15** (200 mg, 0.637 mmol, 1.0 eq), (*S*)-3-(*tert*-butoxycarbonylamino)piperidine (140 mg, 0.701 mmol, 1.1 eq) and triethylamine (98 µL, 0.701 mmol, 1.1 eq). The mixture was stirred for 18 h and the extraction was conducted with diethyl ether (3:1, 5 x 30 mL), yielding product **18** (281 mg, 0.588 mmol, 92%) as a beige solid. Mp: 187 – 188 °C (decomposition and melting). ^{1}H NMR (500 MHz, $CDCl_3$) δ 7.57 (d, J = 2.0 Hz, 1H), 7.41 (dd, J = 8.3, 2.0 Hz, 1H), 6.92 (d, J = 8.4 Hz, 1H), 5.16 (s, 1H), 3.97 (s, 3H), 3.94 (s, 3H), 3.83 (s, 1H), 3.36 (s, 2H), 2.95 (s br, 1H), 2.69 – 2.36 (m, 3H), 2.01 – 1.63 (m, 3H), 1.44 (s, 10H, $C(CH_3)_3$ and 4-H piperidine). ^{13}C NMR (126 MHz, $CDCl_3$) δ 163.9, 162.7, 157.4, 155.3, 151.4, 149.6, 123.3, 121.0, 111.3, 109.6, 79.8, 77.4, 61.2, 59.4, 56.3, 56.2, 54.3, 46.5, 28.5, 22.7. HRMS (ESI): *m/z* = calculated for $C_{22}H_{32}N_5O_5S$ $[M+H]^+$: 478.2119; found: 478.2133. Specific rotation: $[\alpha]_D^{20}$ = - 1.8 (c = 0.22).

***tert*-Butyl (*S*)-(1-(2-((5-(3,4-dimethoxyphenyl)-1,3,4-thiadiazol-2-yl)amino)-2-oxoethyl)piperidin-3-yl)carbamate (19)**

Piperidinecarbamate **19** was prepared following General procedure F, using alkyl chloride **15** (200 mg, 0.637 mmol, 1.0 eq), (*S*)-3-(*tert*-butoxycarbonylamino)piperidine (140 mg, 0.701 mmol, 1.1 eq) and triethylamine (98 µL, 0.70 mmol, 1.1 eq). The mixture was stirred for 18 h and the extraction was conducted with diethyl ether (3:1, 5 x 30 mL), yielding product **19** (284 mg, 0.595 mmol, 93%) as a beige solid. Mp: 189 – 190 °C (decomposition and melting). ^{1}H NMR (500 MHz, $CDCl_3$) δ 7.57 (d, J = 2.1 Hz, 1H), 7.41 (dd, J = 8.3, 2.1 Hz, 1H), 6.92 (d, J = 8.4 Hz, 1H), 5.18 (s, 1H) 3.97 (s, 3H), 3.94 (s, 3H), 3.88 (s, 1H), 3.40 (s, 2H), 2.95 (s br, 1H), 2.67 – 2.35 (m, 3H), 1.97 – 1.79 (m, 3H), 1.44 (s, 10H, $C(CH_3)_3$ and 4-H piperidine). ^{13}C NMR (126 MHz, $CDCl_3$) δ 163.9, 162.7, 157.5, 155.3, 151.4, 149.6, 123.3, 121.0, 111.3, 109.6, 79.8, 77.4, 61.2, 59.3, 56.3, 56.2, 54.3, 46.5, 28.5, 22.6. HRMS (ESI): *m/z* = calculated for $C_{22}H_{32}N_5O_5S$ $[M+H]^+$: 478.2119; found: 478.2125. Specific rotation: $[\alpha]_D^{20}$ = - 3.9 (c = 0.26).

2-Amino-5-methoxyphenol (20)

2-Amino-5-methoxyphenol (**20**) was prepared as described in {Patent US2011224269A1} from 5-methoxy-2-nitrophenol (338 mg, 2.00 mmol, 1.0 eq), using palladium on charcoal (85 mg, 10 wt.%, 0.080 mmol, 0.04 eq) under hydrogen atmosphere. Red solid (260 mg, 1.87 mmol, 93%). Due to its instability, product **20** was used immediately for the next step. Mp: 127 – 129 °C. ^{1}H NMR (400 MHz, MeOD): δ 6.67 (dd, J = 8.4, 0.8 Hz, 1H), 6.36 (d, J = 2.7 Hz, 1H), 6.26 (ddd, J = 8.4, 2.6, 0.7 Hz, 1H), 3.67 (d, J = 0.7 Hz, 3H). ^{13}C NMR (101 MHz, MeOD): δ 155.3, 147.8, 129.0, 118.3, 105.4, 103.0, 56.0. HRMS (ESI): *m/z* = calculated for $C_7H_8NO_2$ $[M-H]^-$: 138.0560; found: 138.0561.

1-(4-Methoxyphenyl)-3-methylthiourea (21)

Methyl isothiocyanate (226 mg, 3.00 mmol, 1.0 eq) was dissolved in 3 mL EtOH and p-anisidine (377 mg, 3.00 mmol, 1.0 eq) was added to the solution. The mixture was stirred at room temperature for 4.5 h. The formed precipitate was filtered and washed with EtOH to give methylthiourea **21** (432 mg, 2.20 mmol, 73%) as a colourless

solid. Mp: 152 – 153 °C. ^{1}H NMR (400 MHz, $CDCl_3$): δ = 7.17 – 7.10 (m, 2H), 6.96 – 6.90 (m, 2H), 3.81 (d, *J* = 0.9 Hz, 3H), 3.10 (d, *J* = 1.2 Hz, 3H). ^{13}C NMR (101 MHz, $CDCl_3$): δ = 182.3, 159.2, 128.5, 128.1, 115.5, 55.7, 32.2. HRMS (ESI): *m/z* = calculated for $C_{13}H_{15}N_2O_3S$ $[M-H]^-$: 195.0597; found: 195.0598.

6-Methoxy-*N*-methylbenzo[*d*]thiazol-2-amine (22)

Phenyl-3-methylthiourea **21** (343 mg, 1.75 mmol, 1.0 eq) was dissolved in 7 mL acetic acid and a solution of bromine (0.179 mL, 3.50 mmol, 2.0 eq) in 1 mL acetic acid was added dropwise at 10 °C. The mixture was stirred at room temperature for 3 h. The mixture was neutralised with conc. ammonia aq. solution and extracted with EtOAc (3 x 40 mL). The organic layers were combined, dried over Na_2SO_4 and the solvent was removed under reduced pressure. The crude product was further purified by flash column chromatography (97:2:1 DCM/MeOH/25% NH_3 aq. solution), yielding *N*-methylbenzothiazole-2-amine **22** (146 mg, 0.752 mmol, 43%) as a colourless solid. Mp: 165 – 166 °C. ^{1}H NMR (400 MHz, MeOD): δ 7.32 (dd, *J* = 8.7, 0.8 Hz, 1H), 7.18 (d, *J* = 2.6 Hz, 1H), 6.86 (ddd, *J* = 8.8, 2.6, 0.7 Hz, 1H), 3.78 (s, 3H), 3.00 (s, 3H). ^{13}C NMR (101 MHz, MeOD): δ 168.7, 156.7, 147.4, 132.2, 119.2, 114.4, 106.5, 56.3, 31.1. HRMS (ESI): *m/z* = calculated for $C_9H_{11}N_2OS$ $[M+H]^+$: 195.0587; found: 195.0586.

2-Chloro-*N*-(6-methoxybenzo[*d*]thiazol-2-yl)-*N*-methylacetamide (23)

Chloroacetamide **23** was prepared following General procedure E, using *N*-methylbenzothiazole-2-amine (**22**, 185 mg, 0.950 mmol, 1.0 eq) and 2-chloroacetyl chloride in THF for 2 h. After filtration, the residue was purified by flash column chromatography (1:4 EtOAc/isohexanes) to give product **23** (218 mg, 0.805 mmol, 85%) as colourless solid. Mp: 138 °C. ^{1}H NMR (500 MHz, $CDCl_3$): δ 7.74 (d, *J* = 8.9 Hz, 1H), 7.27 (d, *J* = 2.6 Hz, 1H), 7.05 (dd, *J* = 8.9, 2.5 Hz, 1H), 4.43 (s, 2H), 3.87 (s, 3H), 3.83 (s, 3H). ^{13}C NMR (126 MHz, $CDCl_3$): δ 166.4, 157.5, 157.2, 142.4, 134.8, 122.4, 115.6, 103.9, 56.0, 42.4, 35.6. HRMS (ESI): *m/z* = calculated for $C_{11}H_{12}ClN_2O_2S$ $[M+H]^+$: 271.0303; found: 271.0306.

6-Methoxybenzo[*d*]oxazol-2-amine (24)

Aminophenol **20** (209 mg, 1.50 mmol, 1.0 eq) and di(1*H*-imidazol-1-yl)methanimine (483 mg, 3.00 mmol, 2.0 eq) were dissolved in 3 mL ACN under nitrogen atmosphere. The solution was heated at 80 °C for 18 h. The solvents were removed under reduced pressure and the residue was purified by flash column chromatography (1:1 EtOAc/isohexanes) to give benzoxazole **24** (138 mg, 0.841 mmol, 56%) as an old pink solid. ^{1}H and ^{13}C NMR data is in accordance with {Patent JP5714745B2}. Mp: 172 – 173 °C. ^{1}H NMR (400 MHz, MeOD): δ 7.09 (d, *J* = 8.5 Hz, 1H), 6.92 (d, *J* = 2.4 Hz, 1H), 6.75 (dd, *J* = 8.5, 2.4 Hz, 1H), 3.78 (d, *J* = 0.7 Hz, 3H). ^{13}C NMR (101 MHz, MeOD): δ 164.3, 156.8, 150.1, 137.1, 116.0, 111.3, 96.8, 56.5. HRMS (ESI): *m/z* = calculated for $C_8H_9N_2O_2$ $[M+H]^+$: 165.0659; found: 165.0659.

2-Chloro-*N*-(6-methoxybenzo[*d*]oxazol-2-yl)acetamide (25)

Chloroacetamide **25** was prepared following General procedure E, using aminobenzoxazole **24** (197 mg, 1.20 mmol, 1.0 eq) and 2-chloroacetyl chloride in THF for 1 h. After filtration, the residue was purified by flash column chromatography (1:1 EtOAc/isohexanes) to give product **25** (213 mg, 0.885 mmol, 74%) as an off-white solid. Mp: 160 – 161 °C. ^{1}H NMR (400 MHz, MeOD): δ 7.43 (d, *J* = 8.7 Hz, 1H), 7.12 (d, *J* = 2.4 Hz, 1H), 6.93 (dd, *J* = 8.8, 2.4 Hz, 1H), 4.34 (s, 2H), 3.83 (s, 3H). ^{13}C NMR (101 MHz, MeOD): δ 166.3, 159.2, 157.4, 149.7, 135.0, 119.3, 113.5, 96.6, 56.5, 43.9. HRMS (ESI): *m/z* = calculated for $C_{10}H_8ClN_5O_3$ $[M-H]^-$: 239.0228; found: 239.0230.

2-Chloro-5-nitropyridine (26)

2-Chloro-5-nitropyridine (**26**) was prepared as described in McPhillie *et al.* from 2-hydroxy-5-nitropyridine (**xx**, 981 mg, 7.00 mmol, 1.0 eq), using phosphorus oxychloride (12.4 mL, 133 mmol, 19 eq).[5] Light yellow solid (985 mg, 6.21 mmol, 89%). Mp: 95 °C [110.2 – 111.8 °C[8]]. ^{1}H NMR (400 MHz, $CDCl_3$): δ 9.24 (dd, *J* = 2.8, 0.7 Hz, 3H), 8.49 – 8.41 (m, 3H), 7.55 (dd, *J* = 8.7, 0.7 Hz, 3H). ^{13}C NMR (101 MHz, $CDCl_3$): δ 157.3, 145.6, 143.5, 133.7, 125.0. HRMS (EI): *m/z* = calculated for $C_5H_3ClN_2O_2$ $[M]^{\cdot+}$: 157.9878; found: 157.9877.

2-Methoxy-5-nitropyridine (27)

2-Methoxy-5-nitropyridine (**27**) was prepared as described in Kokot *et al.* from 2-chloropyridine **26** (920 mg, 5.80 mmol, 1.0 eq), using potassium *tert*-butoxide (846 mg, 7.54 mmol, 1.3 eq).[9] Colourless solid (810 mg, 5.26 mmol, 91%). Mp: 100 – 101 °C. ^{1}H NMR (400 MHz, $CDCl_3$): δ 9.11 – 9.06 (m, 1H), 8.38 – 8.31 (m, 1H), 6.82 (dd, *J* = 9.2, 0.6 Hz, 1H), 4.05 (d, *J* = 0.7 Hz, 3H). ^{13}C NMR (101 MHz, $CDCl_3$): δ 167.5, 144.0, 139.6, 134.0, 111.4, 55.0. HRMS (EI): *m/z* = calculated for $C_6H_6N_2O_3$ $[M]^{\cdot+}$: 154.0373; found: 154.0371.

6-Methoxypyridin-3-amine (28)

Aminopyridine **28** was prepared following General procedure G from nitroyridine **27** (801 mg, 5.20 mmol, 1.0 eq). The mixture was stirred for 1.5 h. The crude product was purified by flash column chromatography (3:1 EtOAc/isohexanes), yielding product **28** (580 mg, 4.67 mmol, 90%) as a brown oil. ^{1}H and ^{13}C NMR data is in accordance with Kokot *et al.*[9] ^{1}H NMR (400 MHz, $CDCl_3$): δ 7.66 (dd, *J* = 3.0, 0.7 Hz, 2H), 7.03 (dd, *J* = 8.7, 3.0 Hz, 2H), 6.60 (dd, *J* = 8.7, 0.8 Hz, 2H), 3.86 (s, 6H). ^{13}C NMR (101 MHz, $CDCl_3$): δ 158.2, 136.8, 133.0, 127.8, 110.9, 53.5. HRMS (EI): *m/z* = calculated for $C_6H_8N_2O$ $[M]^{\cdot+}$: 124.0631; found: 124.0631.

5-Methoxythiazolo[5,4-*b*]pyridin-2-amine (29)

Thiazolopyridin-2-amine **29** was prepared as described in Bebernitz *et al.* from aminopyridine **28** (472 mg, 3.80 mmol, 1.0 eq), using potassium thiocyanate (1.11 g, 11.4 mmol, 3.0 eq) and bromine (0.234 mL, 4.56 mmol, 1.2 eq).[10] Yellow solid (594 mg, 3.28 mmol, 86%). Mp: 186 – 189 °C. ^{1}H NMR (400 MHz, MeOD): δ 7.57 (d, *J* = 8.6 Hz, 1H), 6.68 (d, *J* = 8.7 Hz, 1H), 3.89 (s, 3H). ^{13}C NMR (101 MHz, MeOD): δ 167.0, 161.6, 151.9, 141.7, 128.7, 109.1, 54.3. HRMS (ESI): *m/z* = calculated for $C_7H_8N_3OS$ $[M+H]^+$: 182.0383; found: 182.0384.

***tert*-Butyl (*S*)-(1-(2-((6-methoxybenzo[*d*]thiazol-2-yl)amino)-2-oxoethyl)piperidin-3-yl)carbamate (30)**

Piperidinecarbamate **30** was prepared following General procedure F, using alkyl chloride **2** (257 mg, 1.00 mmol, 1.0 eq), (*S*)-3-(*tert*-butoxycarbonylamino)piperidine (220 mg, 1.10 mmol, 1.1 eq) and triethylamine (0.153 mL, 1.10 mmol, 1.1 eq). The mixture was stirred for 1 h and the extraction was conducted with EtOAc (3:1, 5 x 30 mL). The crude product was purified by flash column chromatography (97:3 DCM/MeOH), yielding product **30** (380 mg, 0.904 mmol, 90%) as a pale yellow solid. Mp: 81 – 84 °C. ^{1}H NMR (400 MHz, $CDCl_3$): δ 10.28 (s, 1H), 7.70 (d, *J* = 8.8 Hz, 1H), 7.30 (d, *J* = 2.6 Hz, 1H), 7.06 (dd, *J* = 8.9, 2.6 Hz, 1H), 4.82 (s, 1H), 3.89 (s, 3H), 3.83 (s, 1H), 3.30 (s, 2H), 2.94 (s br, 1H), 2.65 (s, 1H), 2.45 (d br, 2H), 1.84 (s br, 3H), 1.45 (s, 10H, $C(CH_3)_3$ and 4-H piperidine). ^{13}C NMR (101 MHz, $CDCl_3$): δ 157.0, 155.2, 155.1, 142.8, 133.6, 121.8, 115.4, 104.4, 79.7, 61.5, 59.6, 56.0, 54.2, 46.8, 29.5, 28.5, 22.9. HRMS (ESI): *m/z* = calculated for $C_{20}H_{28}N_4O_4S$ $[M+H]^+$: 421.1905; found: 421.1910. Specific rotation: $[\alpha]_D^{20}$ = - 5.5 (c = 0.25).

***tert*-Butyl (*R*)-(1-(2-((6-methoxybenzo[*d*]thiazol-2-yl)amino)-2-oxoethyl)piperidin-3-yl)carbamate (31)**

Piperidinecarbamate **30** was prepared following General procedure F, using alkyl chloride **2** (257 mg, 1.00 mmol, 1.0 eq), (*R*)-3-(*tert*-butoxycarbonylamino)piperidine (220 mg, 1.10 mmol, 1.1 eq) and triethylamine (0.153 mL, 1.10 mmol, 1.1 eq). The mixture was stirred for 1 h and the extraction was conducted with EtOAc (3:1, 5 x 30 mL). The crude product was purified by flash column chromatography (97:3 DCM/MeOH), yielding product **31** (401 mg, 0.954 mmol, 95%) as a pale yellow solid. Mp: 75 – 76 °C. ^{1}H NMR (400 MHz, $CDCl_3$): δ 10.28 (s, 1H), 7.70 (d, *J* = 8.8 Hz, 1H), 7.30 (d, *J* = 2.6 Hz, 1H), 7.06 (dd, *J* = 8.9, 2.6 Hz, 1H), 4.82 (s, 1H), 3.89 (s, 3H), 3.83 (s, 1H), 3.30 (s, 2H), 2.94 (s br, 1H), 2.65 (s, 1H), 2.45 (s br, 2H), 1.84 (s br, 3H), 1.45 (s, 10H, $C(CH_3)_3$ and 4-H piperidine). ^{13}C NMR (101 MHz, $CDCl_3$): δ 157.0, 155.2, 155.1, 142.8, 133.6, 121.8, 115.4, 104.4, 79.7, 61.5, 59.6, 56.0, 54.2, 46.8, 29.5, 28.5, 22.9. HRMS (ESI): *m/z* = calculated for $C_{20}H_{28}N_4O_4S$ $[M+H]^+$: 421.1905; found: 421.1910. Specific rotation: $[\alpha]_D^{20}$ = + 4.5 (c = 0.20).

2-Chloro-*N*-(5-methoxythiazolo[5,4-*b*]pyridin-2-yl)acetamide (32)

Chloroacetamide **32** was prepared following General procedure E, using thiazolopyridin-2-amine **29** (598 mg, 3.30 mmol, 1.0 eq) and 2-chloroacetyl chloride in THF for 2 h. The extraction gave product **32** (847 mg, 3.29 mmol, quant.) as an orange solid. Mp: 166 – 169 °C. ^{1}H NMR (500 MHz, $CDCl_3$): δ 7.90 (d, *J* = 8.7 Hz, 1H), 6.85 (d, *J* =

8.8 Hz, 1H), 4.31 (s, 2H), 4.00 (s, 3H). ^{13}C NMR (126 MHz, $CDCl_3$): δ 164.5, 162.3, 154.3, 152.1, 136.0, 130.8, 110.6, 54.3, 42.2. HRMS (ESI): *m/z* = calculated for $C_9H_9ClN_3O_2S$ $[M+H]^+$: 258.0099; found: 258.0100.

2,4-Dichloropyrimidin-5-amine (33)

2,4-Dichloro-5-nitropyrimidine (1.16 g, 6.00 mmol, 1.0 eq) and $SnCl_2$ (6.78 g, 30.0 mmol, 5.0 eq) were suspended in 12 mL EtOH. The mixture was refluxed at 80 °C for 1 h. After cooling in an ice-bath, cold water (15 mL) was added, the mixture was neutralised with $NaHCO_3$ sat. aq. solution and extracted with EtOAc (3 x 20 mL). The organic layers were combined, dried over Na_2SO_4 and the solvent was removed under reduced pressure. The crude product was further purified by flash column chromatography (1:4 EtOAc/isohexanes), yielding pyrimidin-5-amine **33** (888 mg, 5.41 mmol, 90%) as a red solid. Mp: 108 – 111 °C. ^{1}H NMR (400 MHz, $CDCl_3$): δ = 8.08 (s, 1H), 3.82 (s br, 2H). ^{13}C NMR (101 MHz, $CDCl_3$): δ = 148.1, 146.2, 144.5, 137.2. HRMS (EI): *m/z* = calculated for $C_4H_3Cl_2N_3$ $[M]^{\cdot+}$: 162.96.99; found: 162.96.98.

5-Chlorothiazolo[5,4-*d*]pyrimidin-2-amine (34)

Aminopyrimidin **33** (868 mg, 5.29 mmol, 1.0. eq) and potassium thiocyanate (1.03 g, 10.6 mmol, 2.0 eq) were dissolved in 13 mL acetic acid and the solution was stirred at 95 °C for 3 h. After cooling in an ice-bath, the mixture was neutralised with conc. ammonia aq. solution and extracted with EtOAc (3 x 80 mL). The organic layers were combined, dried over Na_2SO_4 and the solvent was removed under reduced pressure. The crude product was further purified by flash column chromatography (2:1 EtOAc/ isohexanes), yielding thiazolopyrimidin-2-amine **34** (751 mg, 4.02 mmol, 76%) as a pale yellow solid. Mp: 250 °C (decomposition). ^{1}H NMR (400 MHz, DMSO-d_6): δ 8.53 (s, 1H), 8.29 (s, 2H). ^{13}C NMR (101 MHz, DMSO-d_6): δ 166.3, 166.1, 149.6, 144.9, 143.9. HRMS (ESI): *m/z* = calculated for $C_5H_2ClN_4S$ $[M-H]^-$: 184.9694; found: 184.9694.

2-Chloro-N-(5-chlorothiazolo[5,4-d]pyrimidin-2-yl)acetamide (35)

Chloroacetamide **35** was prepared following General procedure E, using thiazolopyrimidin-2-amine **34** (373 mg, 2.00 mmol, 1.0 eq) and 2-chloroacetyl chloride in THF for 2 h. After extraction, the residue was purified by flash column chromatography (1:2 EtOAc/ isohexanes) to give product **35** (394 mg, 1.50 mmol, 75%) as a beige solid. Mp: 142 – 144 °C. ^{1}H NMR (400 MHz, $CDCl_3$): δ 8.97 (s, 1H), 4.37 (s, 2H). ^{13}C NMR (101 MHz, $CDCl_3$): δ = 165.9, 165.3, 157.7, 155.3, 150.1, 139.4, 42.1. HRMS (ESI): *m/z* = calculated for $C_7H_3Cl_2N_4OS$ $[M-H]^-$: 260.9410; found: 260.9411.

Ethyl (5-chlorothiazolo[5,4-*d*]pyrimidin-2-yl)carbamate (36)

To a suspension of thiazolopyrimidin-2-amine **34** (700 mg, 3.75 mmol, 1.0 eq) in 8 mL 1,4-dioxane, ethyl chloroformate (0.43 mL, 4.5 mmol, 1.2 eq) and pyridine (0.36 mL, 4.5 mmol, 1.2 eq) were added at 0 °C. The mixture was stirred at the same temperature for 2 h. Water (80 mL) was added and the mixture was extracted with EtOAc (3 x 80 mL). The organic layers were combined, dried over Na_2SO_4 and the solvent was removed under reduced pressure. The crude product was further purified by flash column chromatography (2:1 EtOAc/isohexanes), yielding carbamate **36** (813 mg, 3.14 mmol, 84%) as a colourless solid. Mp: 152 °C (decomposition). ^{1}H NMR (400 MHz, DMSO-d_6): δ 12.66 (s, 1H), 9.04 (s, 1H), 4.29 (q, J = 7.1 Hz, 2H), 1.29 (t, J = 7.1 Hz, 3H). ^{13}C NMR (101 MHz, DMSO-d_6): δ 165.7, 160.3, 154.1, 152.4, 149.0, 140.8, 62.7, 14.2. HRMS (ESI): *m/z* = calculated for $C_8H_6ClN_4O_2S$ $[M-H]^-$: 256.9905; found: 256.9905.

5-Methoxythiazolo[5,4-*d*]pyrimidin-2-amine (37)

5-Methoxythiazolopyrimidin-2-amine **37** was prepared as described in {Patent JP5714745} from carbamate **36** (259 mg, 1.00 mmol, 1.0 eq), using sodium methoxide (10.2 mL, 25% in MeOH, 46.0 mmol, 46 eq). Colourless solid (155 mg, 0.851 mmol, 85%). Mp: 265 °C (decomposition). ^{1}H NMR (400 MHz, DMSO-d_6): δ 8.41 (s, 1H), 7.82 (s, 2H), 3.88 (s, 3H). ^{13}C NMR (101 MHz, DMSO-d_6): δ = 166.0, 163.3, 159.6, 143.7, 141.0, 54.7. HRMS (ESI): *m/z* = calculated for $C_6H_7N_4OS$ $[M+H]^+$: 183.0336; found: 183.0336.

2-Chloro-*N*-(5-methoxythiazolo[5,4-*d*]pyrimidin-2-yl)acetamide (38)

Chloroacetamide **38** was prepared following General procedure E, using thiazolopyrimidin-2-amine **37** (149 mg, 0.820 mmol, 1.0 eq) and 2-chloroacetyl chloride in THF for 2 h. After extraction, the residue was purified by flash column chromatography (1:1 EtOAc/isohexanes) to give product **38** (210 mg, 0.812 mmol, 99%) as a beige solid. Mp: 251 °C (decomposition). ^{1}H NMR (500 MHz, DMSO-d_6): δ 12.93 (s, 1H), 9.00 (s, 1H), 4.49 (s, 2H), 3.98 (s, 3H). ^{13}C NMR (126 MHz, DMSO-d_6): δ 166.5, 165.0, 161.5, 154.7, 149.9, 136.2, 55.2, 42.5. HRMS (EI): *m/z* = calculated for $C_8H_7N_4O_2S$ $[M]^{\cdot+}$: 257.9973; found: 257.9974.

3-((2,2-Diethoxyethyl)amino)phenol (39)

To a suspension of 3-aminophenol (1.64 g, 15.0 mmol, 1.0 eq) and $NaHCO_3$ (1.38 g, 16.5 mmol 1.1 eq) in 25 mL DMF, bromoacetaldehyde diethyl acetal (2.33 mL, 15.0 mmol, 1.0 eq) was added. The mixture was stirred at 120 °C for 16 h. Water (200 mL) was added and the mixture was extracted with diethyl ether (3 x 250 mL). The organic layers were combined, dried over Na_2SO_4 and the solvent was removed under reduced pressure. The crude product was purified by flash column chromatography (1:4 → 1:3, EtOAc/isohexanes), yielding product **39** (2.29 g, 10.2 mmol, 68%) as light yellow oil. ^{1}H NMR (400 MHz, $CDCl_3$): δ 7.02 (t, *J* = 8.0 Hz, 1H), 6.23 (ddd, *J* = 8.1, 2.2, 0.9 Hz, 1H), 6.20 (ddd, *J* = 8.0, 2.4, 0.8 Hz, 1H), 6.15 (t, *J* = 2.3 Hz, 1H), 4.68 (t, *J* = 5.5 Hz, 1H), 3.73 (dq, *J* = 9.5, 7.1 Hz, 2H), 3.57 (dq, *J* = 9.4, 7.0 Hz, 2H), 3.23 (d, *J* = 5.5 Hz, 2H), 1.24 (t, *J* = 7.1 Hz, 6H). ^{13}C NMR (101 MHz, $CDCl_3$): δ 157.0, 149.4, 130.4, 106.4, 105.1, 100.9, 100.3, 62.6, 46.6, 15.5. HRMS (ESI): *m/z* = calculated for $C_{12}H_{18}NO_3$ $[M-H]^-$: 224.1292; found: 224.1490.

3-((2,2-Diethoxyethyl)(methyl)amino)phenol (40)

N-Methylaminophenol **40** was prepared following General procedure J, using aminophenol **39** (901 mg, 4.00 mmol, 1.0 eq) and iodomethane. Pink oil (638 mg, 2.67 mmol, 67%). ^{1}H NMR (400 MHz, $CDCl_3$): δ 7.07 (t, *J* = 8.1 Hz, 1H), 6.32 (dd, *J* = 8.3, 2.4 Hz, 1H), 6.25 (s, 1H), 6.20 (dd, *J* = 7.9, 2.2 Hz, 1H), 5.03 (s, 1H), 4.65 (t, *J* = 5.3 Hz, 1H), 3.77 – 3.66 (m, 2H), 3.59 – 3.47 (m, 2H), 3.43 (d, *J* = 5.3 Hz, 2H), 2.99 (s, 3H), 1.20 (td, *J* = 7.1, 0.6 Hz, 6H). ^{13}C NMR (101 MHz, $CDCl_3$): δ 156.9, 150.8, 130.2, 104.9, 103.6, 101.4, 99.3, 63.5, 56.4, 39.8, 15.6. HRMS (ESI): *m/z* = calculated for $C_{13}H_{20}NO_3$ $[M-H]^-$: 238.1448; found: 238.1446.

3-((2,2-Diethoxyethyl)(methyl)amino)phenyl 2-nitrobenzenesulfonate (41)

Nosyl derivative **41** was prepared following General procedure K from *N*-methylaminophenol **40** (362 mg, 2.64 mmol, 1.0 eq). Bright orange oil (1.08 g, 2.54 mmol, 96%). ^{1}H NMR (400 MHz, $CDCl_3$): δ = 8.01 – 7.97 (m, 1H), 7.87 – 7.75 (m, 2H, 5-H), 7.67 (ddd, *J* = 7.9, 6.2, 2.7 Hz, 1H), 7.13 (t, *J* = 8.3 Hz, 1H), 6.69 – 6.64 (m, 1H), 6.57 (t, *J* = 2.4 Hz, 1H), 6.48 (ddd, *J* = 8.1, 2.3, 0.8 Hz, 1H), 4.58 (t, *J* = 5.2 Hz, 1H), 3.70 (dq, *J* = 9.3, 7.0 Hz, 2H), 3.49 (dq, *J* = 9.3, 7.0 Hz, 2H), 3.38 (d, *J* = 5.2 Hz, 2H), 2.95 (s, 3H), 1.18 (t, *J* = 7.0 Hz, 6H). ^{13}C NMR (101 MHz, $CDCl_3$): δ 150.5, 150.4, 148.9, 135.3, 132.3, 132.0, 130.2, 128.9, 124.9, 111.5, 109.7, 106.0, 101.0, 63.6, 56.3, 39.9, 15.6. HRMS (EI): m/z = calculated for $C_{19}H_{20}N_2O_7S$ $[M]^{\cdot+}$: 424.1299; found: 424.1302.

3-(Methyl(2-oxoethyl)amino)phenyl 2-nitrobenzenesulfonate (42)

Aldehyde **42** was prepared following General procedure L from diethyl acetal **41** (1.06 g, 2.50 mmol, 1.0 eq). Brown waxy solid (419 mg, 1.20 mmol, 48%). ^{1}H NMR (400 MHz, $CDCl_3$): δ 9.65 (t, J = 1.0 Hz, 1H), 7.97 – 7.92 (m, 1H), 7.82 – 7.80 (m, 2H), 7.70 – 7.64 (m, 1H), 7.14 (t, *J* = 8.3 Hz, 1H), 6.58 – 6.51 (m, 2H), 6.45 (t, *J* = 2.4 Hz, 1H), 4.03 (d, *J* = 1.0 Hz, 2H), 3.02 (s, 3H). ^{13}C NMR (101 MHz, $CDCl_3$): δ 200.2, 150.6, 150.4, 148.8, 135.4, 132.4, 132.1, 130.5, 128.6, 124.9, 111.4, 110.6, 106.1, 62.7, 39.9. HRMS (ESI): *m/z* = calculated for $C_{15}H_{15}N_2O_6S$ $[M+H]^+$: 351.9646; found: 351.0644.

3-((2,2-Diethoxyethyl)(ethyl)amino)phenol (43)

N-Ethylaminophenol **43** was prepared following General procedure J, using aminophenol **39** (951 mg, 4.22 mmol, 1.0 eq) and iodoethane. Pale pink oil (850 mg, 3.36 mmol, 80%). ^{1}H NMR (400 MHz, $CDCl_3$): δ 7.06 (t, *J* = 8.1 Hz, 1H), 6.30 (d, *J* = 8.4 Hz, 1H), 6.24 (s br, 1H), 6.18 (s br, 1H), 5.21 (s, 1H), 4.67 (d, *J* = 5.4 Hz, 1H), 3.73 (dq, *J* = 9.3, 7.0 Hz, 2H), 3.54 (dq, *J* = 9.3, 7.0 Hz, 2H), 3.45 (d, *J* = 7.1 Hz, 1H), 3.4 (d, *J* = 5.2 Hz, 2H), 1.21 (t, *J* =

7.1 Hz, 6H), 1.14 (t, *J* = 7.0 Hz, 3H). ^{13}C NMR (101 MHz, $CDCl_3$): δ 157.1, 149.2, 130.3, 104.7, 103.0, 101.6, 98.9, 63.5, 54.3, 45.9, 15.6, 11.8. HRMS (ESI): *m/z* = calculated for $C_{14}H_{22}NO_3$ [M-H]$^-$: 252.1605; found: 252.1602.

3-((2,2-Diethoxyethyl)(ethyl)amino)phenyl 2-nitrobenzenesulfonate (44)

Nosyl derivative **44** was prepared following General procedure K from *N*-ethylaminophenol **43** (798 mg, 3.15 mmol, 1.0 eq). Bright orange oil (1.24 g, 2.79 mmol, 89%). ^{1}H NMR (400 MHz, $CDCl_3$): δ 7.99 (ddd, *J* = 7.9, 1.2, 0.5 Hz, 1H), 7.84 – 7.76 (m, 2H, 5-H), 7.67 (ddd, *J* = 7.9, 6.5, 2.4 Hz, 1H), 7.10 (t, *J* = 8.3 Hz, 1H), 6.62 (dd, *J* = 8.4, 2.5 Hz, 1H), 6.52 (t, *J* = 2.4 Hz, 1H), 6.47 – 6.39 (m, 1H), 4.56 (t, *J* = 5.1 Hz, 1H), 3.70 (dq, *J* = 9.3, 7.1 Hz, 2H), 3.50 (dq, *J* = 9.3, 7.0 Hz, 2H), 3.38 (q, *J* = 7.1 Hz, 2H), 3.33 (d, *J* = 5.1 Hz, 2H), 1.19 (t, *J* = 7.0 Hz, 6H), 1.06 (t, *J* = 7.0 Hz, 3H). ^{13}C NMR (101 MHz, $CDCl_3$): δ 150.7, 149.3, 148.9, 135.2, 132.3, 132.0, 130.2, 129.0, 124.8, 111.1, 108.9, 105.6, 101.2, 63.6, 54.3, 46.1, 15.5, 11.5. HRMS (EI): m/z = calculated for $C_{20}H_{26}N_2O_7S$ [M]$^{\cdot+}$: 438.1456; found: 438.1454.

(*S*)-3-((2-((1-(2-((6-Methoxybenzo[d]thiazol-2-yl)amino)-2-oxoethyl)piperidin-3-yl)amino)ethyl)(methyl)amino)phenyl 2-nitrobenzenesulfonate (45)

Secondary amine **45** was prepared following General procedure M, using 3-aminopiperidine **A.36** (123 mg, 0.350 mmol, 1.0 eq) and aldehyde **42**. Light brown solid (112 mg, 0.171 mmol, 49%). Mp: 62 – 65 °C. ^{1}H NMR (500 MHz, DMSO) δ 8.18 (dd, *J* = 8.0, 1.1 Hz, 1H), 8.04 (td, *J* = 7.7, 1.4 Hz, 1H), 7.97 (dd, *J* = 8.1, 1.4 Hz, 1H), 7.86 (td, *J* = 7.8, 1.3 Hz, 1H), 7.62 (d, *J* = 8.8 Hz, 1H), 7.56 (d, *J* = 2.6 Hz, 1H), 7.12 (t, *J* = 8.2 Hz, 1H), 7.03 (dd, *J* = 8.8, 2.6 Hz, 1H), 6.63 (dd, *J* = 8.5, 2.4 Hz, 1H), 6.31 (t, *J* = 2.3 Hz, 1H), 6.29 (dd, *J* = 7.9, 2.2 Hz, 1H), 3.81 (s, 3H), 3.30 (s, 2H), 3.27 (d, *J* = 7.0 Hz, 2H), 2.81 (d, *J* = 8.2 Hz, 1H), 2.79 (s, 3H), 2.68 – 2.62 (m, 1H), 2.59 (t, *J* = 7.1 Hz, 2H), 2.55 (s, 1H), 2.27 – 2.20 (m, 1H), 2.03 (t, *J* = 9.6 Hz, 1H), 1.77 – 1.67 (m, 1H), 1.64 (t, *J* = 7.7 Hz, 1H), 1.52 – 1.40 (m, 1H), 1.04 (d, *J* = 9.4 Hz, 1H). ^{13}C NMR (126 MHz, DMSO-d_6) δ = 169.6, 156.2, 155.4, 150.4, 150.1, 148.0, 142.5, 136.8, 132.9, 132.8, 131.8, 130.2, 126.4, 125.2, 121.1, 114.9, 110.9, 107.6, 104.7, 104.3, 60.7, 59.0, 55.6, 53.7, 53.3, 52.3, 43.0, 38.2, 30.1, 23.2. HRMS (ESI): *m/z* = calculated for $C_{30}H_{35}N_6O_7S_2$ [M+H]$^+$: 655.2003; found: 655.1992. Specific rotation: $[\alpha]_D^{20}$ = + 4.1 (c = 0.25).

3-(Ethyl(2-oxoethyl)amino)phenyl 2-nitrobenzenesulfonate (46)

Aldehyde **46** was prepared following General procedure L from diethyl acetal **44** (570 mg, 1.30 mmol, 1.0 eq). Brown sticky oil (360 mg, 0.988 mmol, 76%). ^{1}H NMR (400 MHz, $CDCl_3$): δ 9.60 (t, *J* = 1.2 Hz, 1H), 7.97 – 7.94 (m, 1H), 7.83 – 7.80 (m, 2H,), 7.70 – 7.65 (m, 1H), 7.13 (t, *J* = 8.3 Hz, 1H), 6.53 (ddd, *J* = 8.1, 2.2, 0.8 Hz, 1H), 6.48 (ddd, *J* = 8.4, 2.6, 0.8 Hz, 1H), 6.42 (t, *J* = 2.4 Hz, 1H), 3.96 (d, *J* = 1.3 Hz, 2H), 3.42 (q, *J* = 7.1 Hz, 2H), 1.14 (t, J = 7.1 Hz, 3H). ^{13}C NMR (101 MHz, $CDCl_3$): δ 201.2, 150.7, 149.1, 148.8, 135.4, 132.3, 132.1, 130.6, 128.7, 124.9, 111.2, 110.3, 106.0, 60.6, 46.7, 12.3. HRMS (ESI): *m/z* = calculated for $C_{16}H_{17}N_2O_6S$ [M+H]$^+$: 365.0802; found: 365.0800.

(*S*)-3-(Ethyl(2-((1-(2-((6-methoxybenzo[d]thiazol-2-yl)amino)-2-oxoethyl)piperidin-3-yl)amino)ethyl)amino)phenyl 2-nitrobenzenesulfonate (47)

Secondary amine **47** was prepared following General procedure M, using 3-aminopiperidine **A.36** (182 mg, 0.500 mmol, 1.0 eq) and aldehyde **46**. Dark yellow solid (167 mg, 0.251 mmol, 50%). Mp: 57 – 60 °C. ^{1}H NMR (500 MHz, DMSO) δ 8.18 (dd, *J* = 8.0, 1.2 Hz, 1H), 8.04 (td, *J* = 7.8, 1.4 Hz, 1H), 7.98 (dd, *J* = 8.0, 1.4 Hz, 1H), 7.86 (td, *J* = 7.8, 1.2 Hz, 1H), 7.62 (d, *J* = 8.8 Hz, 1H), 7.56 (d, *J* = 2.6 Hz, 1H), 7.10 (t, *J* = 8.5 Hz, 1H), 7.03 (dd, *J* = 8.8, 2.6 Hz, 1H), 6.64 – 6.56 (m, 1H), 6.26 (dd, *J* = 7.7, 1.8 Hz, 2H), 3.81 (s, 3H), 3.21 (t, *J* = 7.2 Hz, 4H), 2.81 (d, *J* = 10.1 Hz, 1H), 2.65 (d, *J* = 7.0 Hz, 1H), 2.60 – 2.51 (m, 3H), 2.27 – 2.20 (m, 1H), 2.04 (t, *J* = 9.7 Hz, 1H), 1.72 (d, *J* = 9.6 Hz, 1H), 1.69 – 1.59 (m, 1H), 1.52 – 1.41 (m, 1H), 1.05 (d, *J* = 11.1 Hz, 1H), 0.91 (t, *J* = 7.0 Hz, 3H). ^{13}C NMR (126 MHz, DMSO) δ 169.56, 156.2, 155.5, 150.2, 149.0, 147.9, 142.5, 136.8, 132.9, 132.8, 131.7, 130.4, 126.5, 125.2, 121.1, 114.9, 110.6, 107.2, 104.7, 104.0, 60.7, 59.0, 55.6, 53.7, 53.4, 50.4, 44.5, 43.7, 30.17, 23.3, 11.6. HRMS (ESI): *m/z* = calculated for $C_{31}H_{37}N_6O_7S_2$ [M+H]$^+$: 669.2160; found: 669.2148. Specific rotation: $[\alpha]_D^{20}$ = + 2.6 (c = 0.23).

(*R*)-3-((2-((1-(2-((6-Methoxybenzo[d]thiazol-2-yl)amino)-2-oxoethyl)piperidin-3-yl)amino)ethyl)(methyl)amino)phenyl 2-nitrobenzenesulfonate (48)

Secondary amine **48** was prepared following General procedure M, using 3-aminopiperidine **A.37** (175 mg, 0.500 mmol, 1.0 eq) and aldehyde **42**. Light brown solid (164 mg, 0.250 mmol, 50%). Mp: 60 – 63 °C. ^{1}H NMR (500 MHz, DMSO-d_6): δ 8.18 (dd, *J* = 8.0, 1.1 Hz, 1H), 8.04 (td, *J* = 7.7, 1.4 Hz, 1H), 7.97 (dd, *J* = 8.1, 1.4 Hz, 1H), 7.86 (td, *J* = 7.8, 1.3 Hz, 1H), 7.62 (d, *J* = 8.8 Hz, 1H), 7.56 (d, *J* = 2.6 Hz, 1H), 7.12 (t, *J* = 8.2 Hz, 1H), 7.02 (dd, *J* = 8.8, 2.6 Hz, 1H), 6.62 (dd, *J* = 8.5, 2.4 Hz, 1H), 6.37 – 6.27 (m, 2H), 3.80 (s, 3H), 3.30 (s, 2H), 3.26 (d, *J* = 7.0 Hz, 2H), 2.79 (d, *J* = 8.2 Hz, 1H), 2.79 (s, 3H), 2.69 – 2.61 (m, 1H), 2.60 – 2.54 (m, 3H), 2.26 – 2.21 (m, 1H), 2.03 (t, *J* = 9.6 Hz, 1H), 1.77 – 1.59 (m, 2H), 1.54 – 1.38 (m, 1H), 1.05 – 1.02 (m, 1H). ^{13}C NMR (126 MHz, DMSO-d_6) δ 169.6, 156.2, 155.4, 150.4, 150.1, 148.0, 142.5, 136.8, 132.9, 132.8, 131.8, 130.2, 126.4, 125.2, 121.1, 114.9, 110.9, 107.6, 104.7, 104.3, 60.7, 59.0, 55.6, 53.7, 53.3, 52.3, 43.0, 38.2, 30.2, 23.3. HRMS (ESI): *m/z* = calculated for $C_{30}H_{35}N_6O_7S_2$ [M+H]$^+$: 655.2003; found: 655.1998. Specific rotation: $[\alpha]_D^{20}$ = - 3.4 (c = 0.21).

(*R*)-3-(Ethyl(2-((1-(2-((6-methoxybenzo[d]thiazol-2-yl)amino)-2-oxoethyl)piperidin-3-yl)amino)ethyl)amino)phenyl 2-nitrobenzenesulfonate (49)

Secondary amine **49** was prepared following General procedure M, using 3-aminopiperidine **A.37** (273 mg, 0.750 mmol, 1.0 eq) and aldehyde **46**. Dark yellow solid (265 mg, 0.396 mmol, 53%). Mp: 65 – 68 °C. ^{1}H NMR (500 MHz, DMSO) δ 8.18 (dd, *J* = 8.0, 1.2 Hz, 1H), 8.04 (td, *J* = 7.8, 1.4 Hz, 1H), 7.98 (dd, *J* = 8.0, 1.4 Hz, 1H), 7.86 (td, *J* = 7.8, 1.2 Hz, 1H), 7.62 (d, *J* = 8.8 Hz, 1H), 7.56 (d, *J* = 2.5 Hz, 1H), 7.10 (t, *J* = 8.5 Hz, 1H), 7.03 (dd, *J* = 8.8, 2.7 Hz, 1H), 6.63 – 6.57 (m, 1H), 6.26 (dd, *J* = 7.5, 1.9 Hz, 2H), 3.81 (s, 3H), 3.22 (q, *J* = 7.4 Hz, 4H), 2.84 – 2.78 (m, 1H), 2.65 (d, *J* = 11.2 Hz, 1H), 2.61 – 2.51 (m, 3H), 2.26 – 2.22 (m, 1H), 2.04 (t, *J* = 9.5 Hz, 1H), 1.72 (d, *J* = 11.8 Hz, 1H), 1.68 – 1.62 (m, 1H), 1.50 – 1.44 (m, 1H), 1.05 (d, *J* = 11.3 Hz, 1H), 0.91 (t, *J* = 6.9 Hz, 3H). ^{13}C NMR (126 MHz, DMSO) δ 169.6, 156.2, 155.5, 150.2, 149.0, 147.9, 142.5, 136.8, 132.9, 132.8, 131.7, 130.4, 126.5, 125.2, 121.1, 114.9, 110.6, 107.2, 104.7, 104.0, 60.7, 59.0, 55.6, 53.7, 53.35, 50.5, 44.5, 43.7, 30.2, 23.3, 11.6. HRMS (ESI): *m/z* = calculated for $C_{31}H_{37}N_6O_7S_2$ [M+H]$^+$: 669.2160; found: 669.2147. Specific rotation: $[\alpha]_D^{20}$ = - 1.9 (c = 0.21).

***tert*-Butyl (*S*)-(1-(2-((4,6-dimethoxybenzo[*d*]thiazol-2-yl)amino)-2-oxoethyl)piperidin-3-yl)carbamate (50)**

Piperidinecarbamate **50** was prepared following General procedure F, using alkyl chloride **6** (143 mg, 0.500 mmol, 1.0 eq), (*S*)-3-(*tert*-butoxycarbonylamino)piperidine (110 mg, 0.550 mmol, 1.1 eq) and triethylamine (78 µL, 0.55 mmol, 1.1 eq). The mixture was stirred for 2 h and the extraction was conducted with EtOAc (3:1, 5 x 30 mL). The crude product was purified by flash column chromatography (97:3 DCM/MeOH), yielding product **50** (191 mg, 0.424 mmol, 85%) as a pale yellow solid. Mp: 145 – 146 °C. ^{1}H NMR (400 MHz, $CDCl_3$) δ 10.20 (s, 1H), 6.86 (d, *J* = 2.2 Hz, 1H), 6.53 (d, *J* = 2.2 Hz, 1H), 4.73 (s, 1H), 3.99 (s, 3H), 3.86 (s, 3H), 3.76 (s, 1H), 3.23 (s, 2H), 2.89 (s br, 1H), 2.53 (s, 1H), 2.42 (s, 1H), 2.34 (s, 1H), 1.71 (s br, 3H), 1.43 (s, 10H, $C(CH_3)_3$ and 4-H piperidine). ^{13}C NMR (101 MHz, $CDCl_3$) δ 167.0, 158.1, 155.2, 153.9, 152.7, 134.4, 133.1, 97.9, 95.2, 79.6, 61.8, 59.8, 56.1, 56.0, 54.4, 46.7, 29.6, 28.5, 23.0. HRMS (ESI): *m/z* = calculated for $C_{21}H_{31}N_4O_5S$ [M+H]$^+$: 451.2010; found: 451.2004. Specific rotation: $[\alpha]_D^{20}$ = - 10.0 (c = 0.21).

***tert*-Butyl (*R*)-(1-(2-((4,6-dimethoxybenzo[*d*]thiazol-2-yl)amino)-2-oxoethyl)piperidin-3-yl)carbamate (51)**

Piperidinecarbamate **51** was prepared following General procedure F, using alkyl chloride **6** (100 mg, 0.350 mmol, 1.0 eq), (*R*)-3-(*tert*-butoxycarbonylamino)piperidine (77 mg, 0.35 mmol, 1.1 eq) and triethylamine (54 µL, 0.35 mmol, 1.1 eq). The mixture was stirred for 2 h and the extraction was conducted with EtOAc (3:1, 5 x 30 mL) and the crude product was purified by flash column chromatography (97:3 DCM/MeOH), yielding product **51** (131 mg, 0.291 mmol, 83%) as a pale yellow solid. Mp: 135 – 137 °C. ^{1}H NMR (500 MHz, $CDCl_3$) δ 10.29 (s, 1H), 6.84 (d, *J* = 2.2 Hz, 1H), 6.50 (d, *J* = 2.2 Hz, 1H), 4.85 (s, 1H), 3.97 (s, 3H), 3.85 (s, 3H), 3.81 (s, 1H), 3.31 (s, 2H), 2.89 (s br, 1H), 2.74 – 2.33 (m, 3H), 1.74 (s br, 3H), 1.43 (s, 10H, $C(CH_3)_3$ and 4-H piperidine). ^{13}C NMR (126 MHz, $CDCl_3$) δ 166.7, 158.1, 155.2, 154.0, 152.6, 134.3, 132.9, 97.9, 95.2, 79.7, 61.5, 59.5, 56.0, 55.9, 54.2, 46.5, 29.4, 28.5, 22.8. HRMS (ESI): *m/z* = calculated for $C_{21}H_{31}N_4O_5S$ [M+H]$^+$: 451.2010; found: 451.2004. Specific rotation: $[\alpha]_D^{20}$ = + 9.9 (c = 0.18).

Depicted product synthesis

N-(6-Methoxybenzo[*d*]thiazol-2-yl)acetamide (x.8)

Amide **x.8** was prepared following General procedure D, using 2-amino-6-methoxybenzothiazole (250 mg, 1.66 mmol, 1.0 eq). Purification by flash column chromatography (9.5:0.5 DCM/MeOH) gave the final product that was recrystallised from DCM to give **x.8** (236 mg, 1.06 mmol, 79%) as a colourless solid. Mp: 230 – 231 °C [226 – 228 °C[11]]. ^{1}H NMR (400 MHz, DMSO-d_6) δ 12.19 (s, 1H), 7.62 (d, *J* = 8.8 Hz, 1H), 7.54 (d, *J* = 2.6 Hz, 1H), 7.01 (dd, *J* = 8.8, 2.6 Hz, 1H), 3.80 (s, 3H), 2.17 (s, 3H). ^{13}C NMR (101 MHz, DMSO-d_6) δ 169.1, 156.1, 155.9, 142.6, 132.7, 121.1, 114.8, 104.7, 55.6, 22.7. HRMS (ESI): m/z = calculated for $C_{10}H_9N_2O_2S$ [M-H]$^-$: 221.0390; found: 221.0391. Purity (HPLC): 100% (λ = 210 nm), 100% (λ = 254 nm), Method 2a.

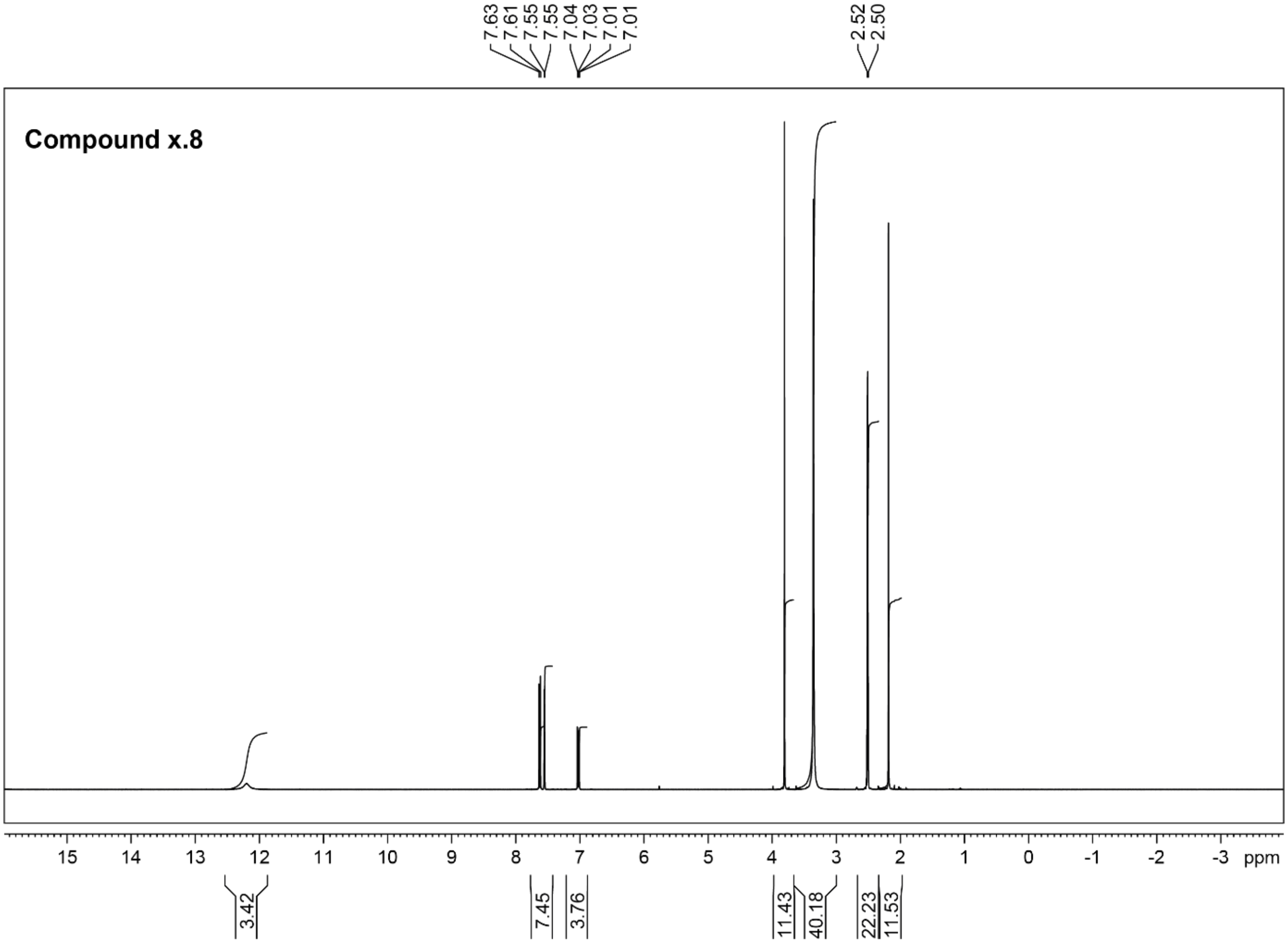

Synthesis Suppl. Figure 1. ^{1}H NMR of compound x.8.

Benzo[*d*]thiazole-2,6-diamine (x.15)

2-Amino-6-nitrobenzothiazole (2.0 g, 10 mmol, 1.0 eq) was dissolved in 8 mL 37% HCl 3 x and 8 mL DMF. $SnCl_2$ (9.7 g, 51 mmol, 5.0 eq) was added to the mixture, that was stirred at room temperature for 2 h. Water (80 mL) was added and the mixture was basified (pH = 9) with 2 M NaOH aq. solution. The precipitate of the resulting suspension was filtered off, washed with water and diethyl ether and discarded. The filtrate was extracted with diethyl ether (3 x 100 mL). The organic layers were combined, dried over Na_2SO_4 and the solvent was removed under reduced pressure. The crude product was treated with diethyl ether (20 mL). The precipitate was filtered, washed with diethyl ether and dried under reduced pressure to give benzothiazole-2,6-diamine (**x.15**, 996 mg, 6.03 mmol, 59%) as beige solid. ^1H and ^{13}C NMR data is in accordance with Jaryal *et al.*[12] Mp: 190 °C (decomposition), 204 °C (melting) [156 – 158 °C[13]]. ^1H NMR (400 MHz, DMSO-d_6) δ 7.03 (d, *J* = 8.4 Hz, 1H), 6.92 (s br, 2H), 6.81 (d, *J* = 2.3 Hz, 1H), 6.49 (dd, *J* = 8.4, 2.3 Hz, 1H), 4.81 (s, 2H). ^{13}C NMR (101 MHz, DMSO) δ 162.4, 143.9, 143.4, 133.0, 118.1, 113.0, 105.4. HRMS (ESI): m/z = calculated for $C_7H_8N_3S$ $[M+H]^+$: 166.0434; found: 166.0434. Purity (HPLC): > 87% (λ = 210 nm), > 86% (λ = 254 nm), Method 1b.

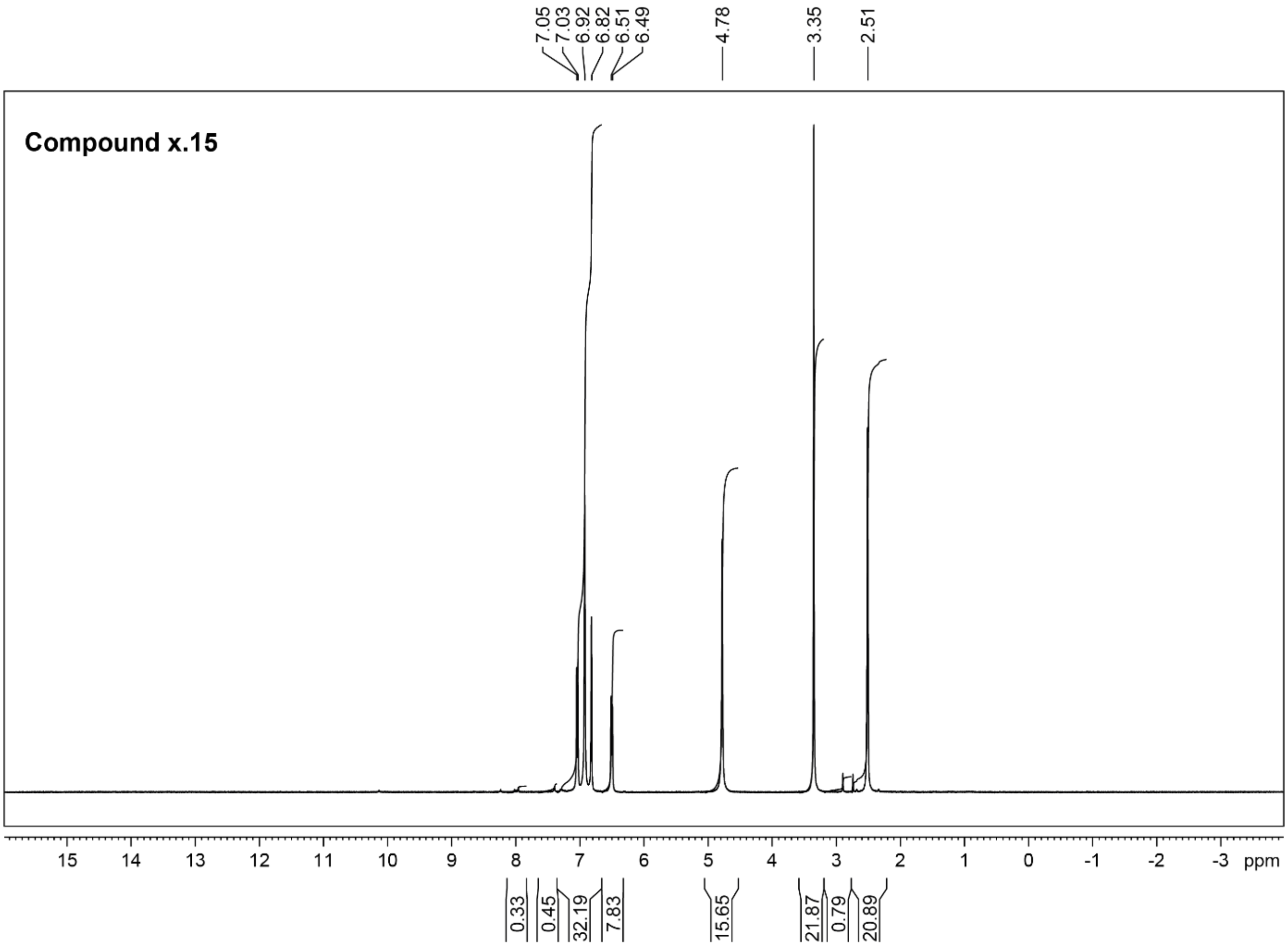

Synthesis Suppl. Figure 2. ^1H NMR of compound x.15.

1-(Benzo[*d*]thiazol-2-ylmethyl)-3-methylurea (x.16)

Methylurea **x.16** was prepared following General procedure B, using 1,3-benzothiazol-2-ylmethylamine (250 mg, 1.45 mmol, 1.0 eq). Off-white solid (120 mg, 0.542 mmol, 37%). Mp: 186 – 187 °C. ^{1}H NMR (400 MHz, DMSO-d_6) δ 8.04 (d, *J* = 8.0 Hz, 1H), 7.91 (d, *J* = 8.1 Hz, 1H), 7.48 (t, *J* = 7.6 Hz, 1H), 7.39 (t, *J* = 7.7 Hz, 1H), 6.88 (s, 1H), 6.14 (s, 1H), 4.58 (d, *J* = 6.1 Hz, 3H), 2.59 (d, *J* = 4.6 Hz, 4H). ^{13}C NMR (101 MHz, DMSO-d_6) δ 174.1, 158.4, 153.0, 134.5, 126.0, 12434.7, 122.2, 42.1, 26.5. HRMS (EI): *m/z* = calculated for $C_{10}H_{11}N_3OS$ $[M]^{\cdot+}$: 221.0618; found: 221.0624. Purity (HPLC): 100% (λ = 210 nm), 100% (λ = 254 nm), Method 1a.

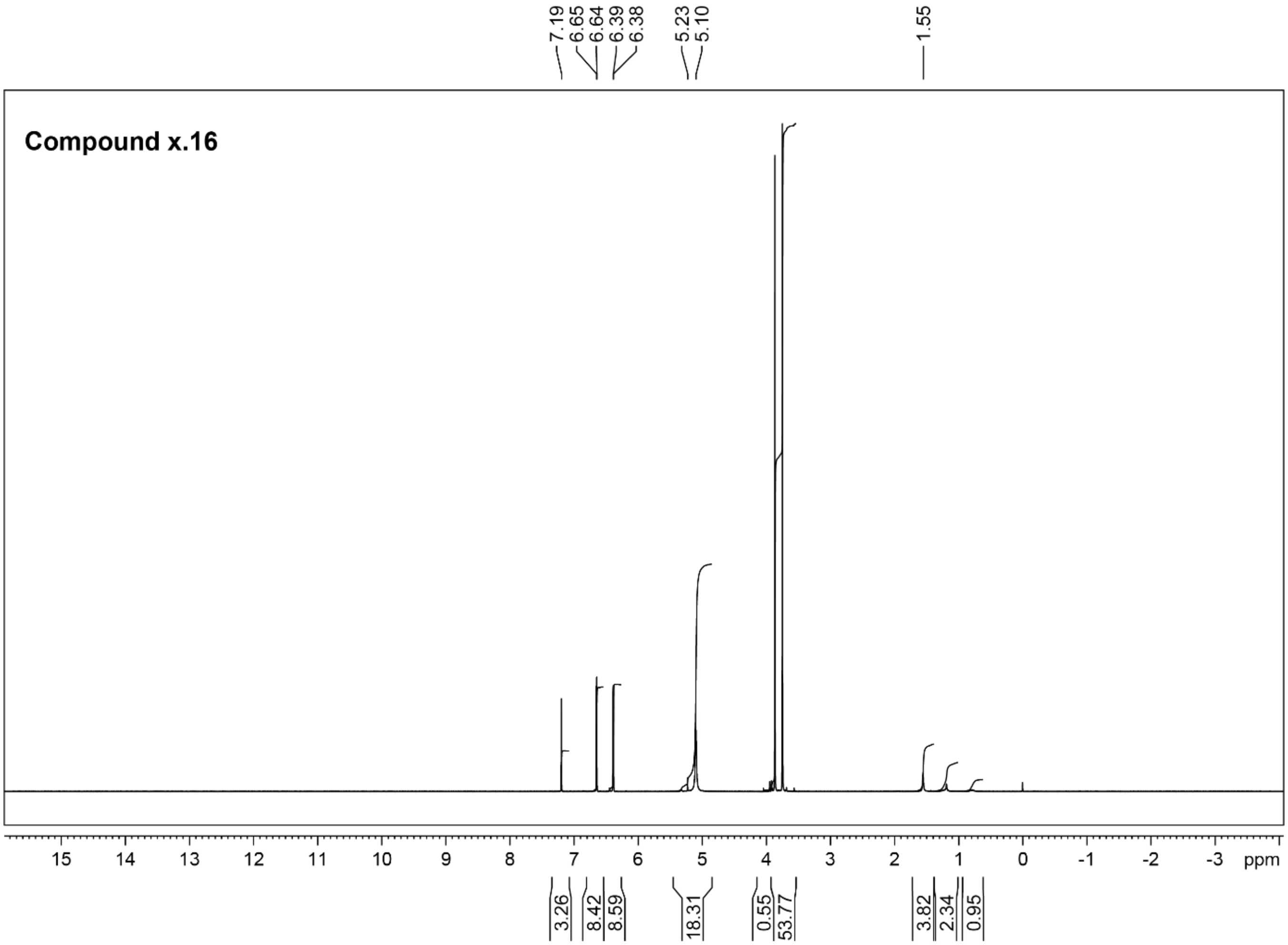

Synthesis Suppl. Figure 3. ^{1}H NMR of compound x.16.

1-(Benzo[*d*]thiazol-2-ylmethyl)-3-methylthiourea (x.17)

Thiourea **x.17** was prepared following General procedure A, using 1,3-benzothiazol-2-ylmethylamine (200 mg, 1.16 mmol, 1.0 eq). The mixture was stirred for 3 h to give thiourea **x.17** (208 mg, 0.876 mmol, 76%) as a beige red solid. Mp: 140 °C. ^{1}H NMR (400 MHz, DMSO-d_6) δ 8.27 (s, 1H), 8.08 – 8.01 (m, 1H), 7.97 – 7.90 (m, 1H), 7.80 (s, 1H), 7.49 (ddd, *J* = 8.2, 7.2, 1.3 Hz, 1H), 7.40 (ddd, *J* = 8.4, 7.2, 1.2 Hz, 1H), 5.09 (s, 2H), 2.88 (s, 3H). ^{13}C NMR (101 MHz, DMSO-d_6) δ 172.0, 152.7, 134.6, 126.0, 124.9, 122.2, 122.2, 40.2, 40.0. HRMS (ESI): m/z = calculated for $C_{10}H_{12}N_3S_2$ [M+H]$^+$: 238.0468; found: 238.0466. Purity (HPLC): 100% (λ = 210 nm), 100% (λ = 254 nm), Method 1a.

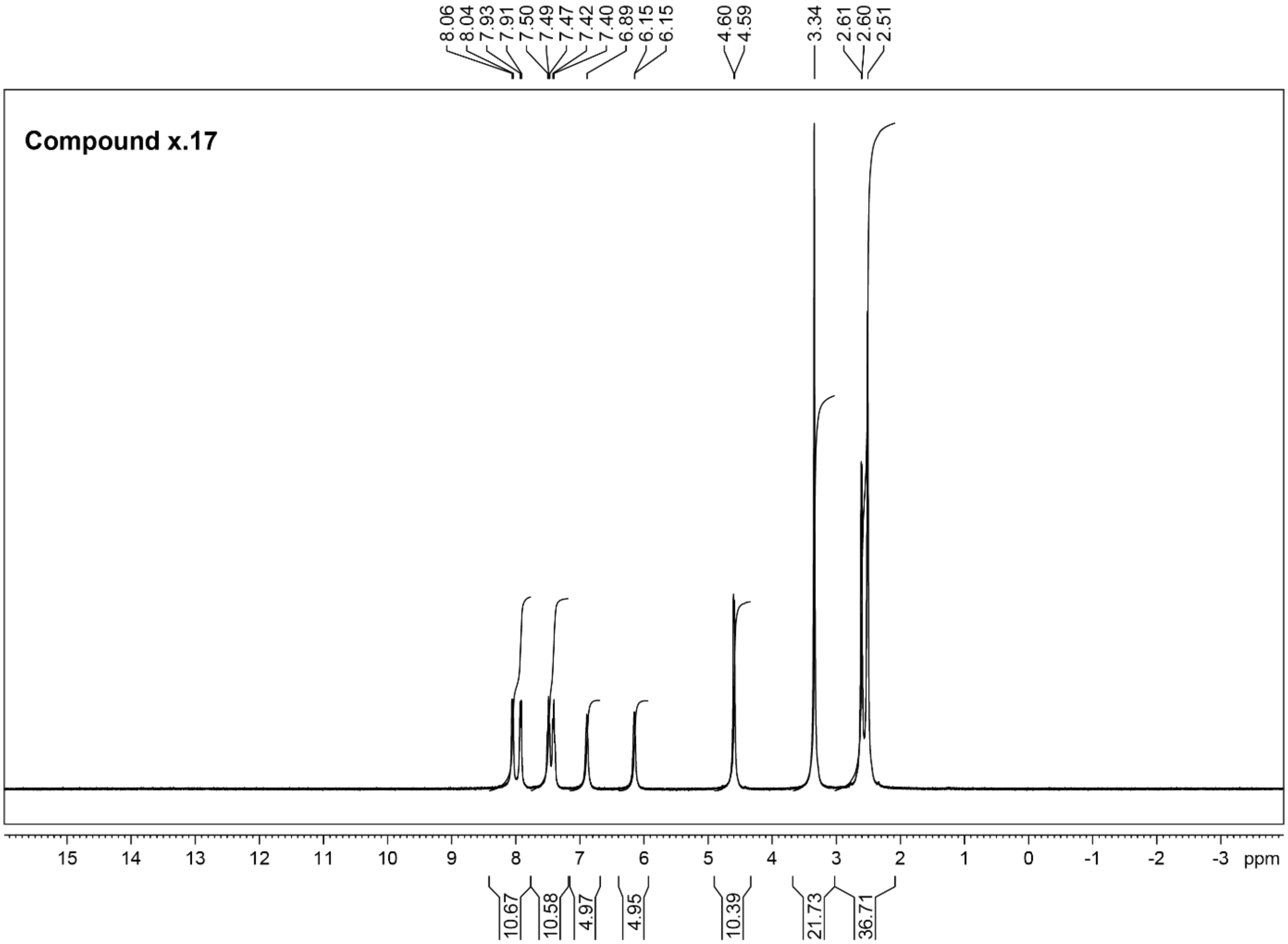

Synthesis Suppl. Figure 4. ^{1}H NMR of compound x.17.

1-(6-Hydroxybenzo[*d*]thiazol-2-yl)-3-methylguanidine (x.18)

6-Hydroxybenzothiazole **x.18** was prepared following General procedure C from 6-methoxybenzothiazole **A.4** (200 mg, 0.846 mmol, 1.0 eq). The crude product was treated with DCM/diethyl ether (2:1, 20 mL) and the insoluble solid was filtered off, washed with diethyl ether and dried under reduced pressure to give **x.18** (148 mg, 0.666 mmol, 79%) as an off-white solid. Mp: 232 °C (decomposition). ^{1}H NMR (400 MHz, DMSO-d_6) δ 9.18 (s, 1H), 7.56 (s, 2H), 7.25 (d, *J* = 8.6 Hz, 1H), 7.02 (d, *J* = 2.5 Hz, 1H), 6.69 (dd, *J* = 8.6, 2.5 Hz, 1H), 6.52 (s, 1H), 2.75 (d, *J* = 4.8 Hz, 3H). ^{13}C NMR (101 MHz, DMSO-d_6) δ 171.2, 157.2, 152.8, 144.9, 131.3, 118.8, 113.7, 106.5, 27.6. HRMS (ESI): m/z = calculated for $C_9H_9N_4OS$ [M-H]$^-$: 221.0502; found: 221.0502. Purity (HPLC): > 99% (λ = 210 nm), > 99% (λ = 254 nm), Method 3b.

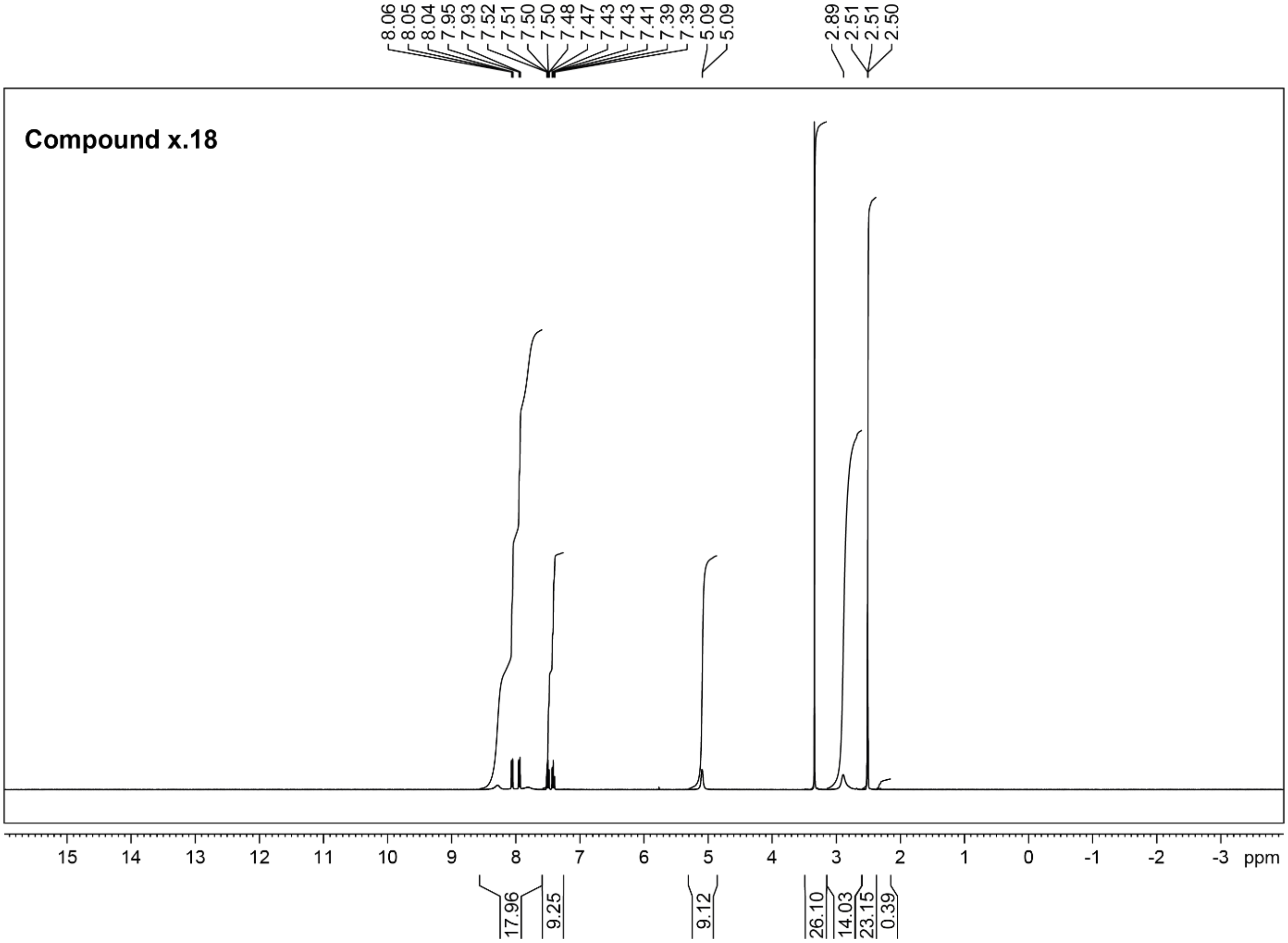

Synthesis Suppl. Figure 5. ^{1}H NMR of compound x.18.

1-(6-Methoxybenzo[*d*]thiazol-2-yl)-3-methylurea (A)

Methylurea **A** was prepared following General procedure B, using 2-amino-6-methoxybenzothiazole (4.0 g, 21 mmol, 1.0 eq). Off-white solid (3.7 g, 16 mmol, 72%). Mp: 320 °C (decomposition). ^{1}H NMR (400 MHz, DMSO-d_6) δ 10.62 (s, 1H), 7.55 – 7.41 (m, 2H), 6.94 (dd, *J* = 8.8, 2.6 Hz, 1H), 6.60 (d, *J* = 5.0 Hz, 1H), 3.78 (s, 3H), 2.71 (d, *J* = 4.7 Hz, 3H). ^{13}C NMR (101 MHz, DMSO-d_6) δ 155.5, 154.4, 143.2, 132.6, 124.3, 120.1, 114.1, 104.8, 55.6, 26.3. HRMS (ESI): m/z = calculated for $C_{10}H_{10}N_3O_2S$ [M-H]$^-$: 236.0499; found: 236.0499. Purity (HPLC): > 98% (λ = 210 nm), > 98% (λ = 254 nm), Method 3a.

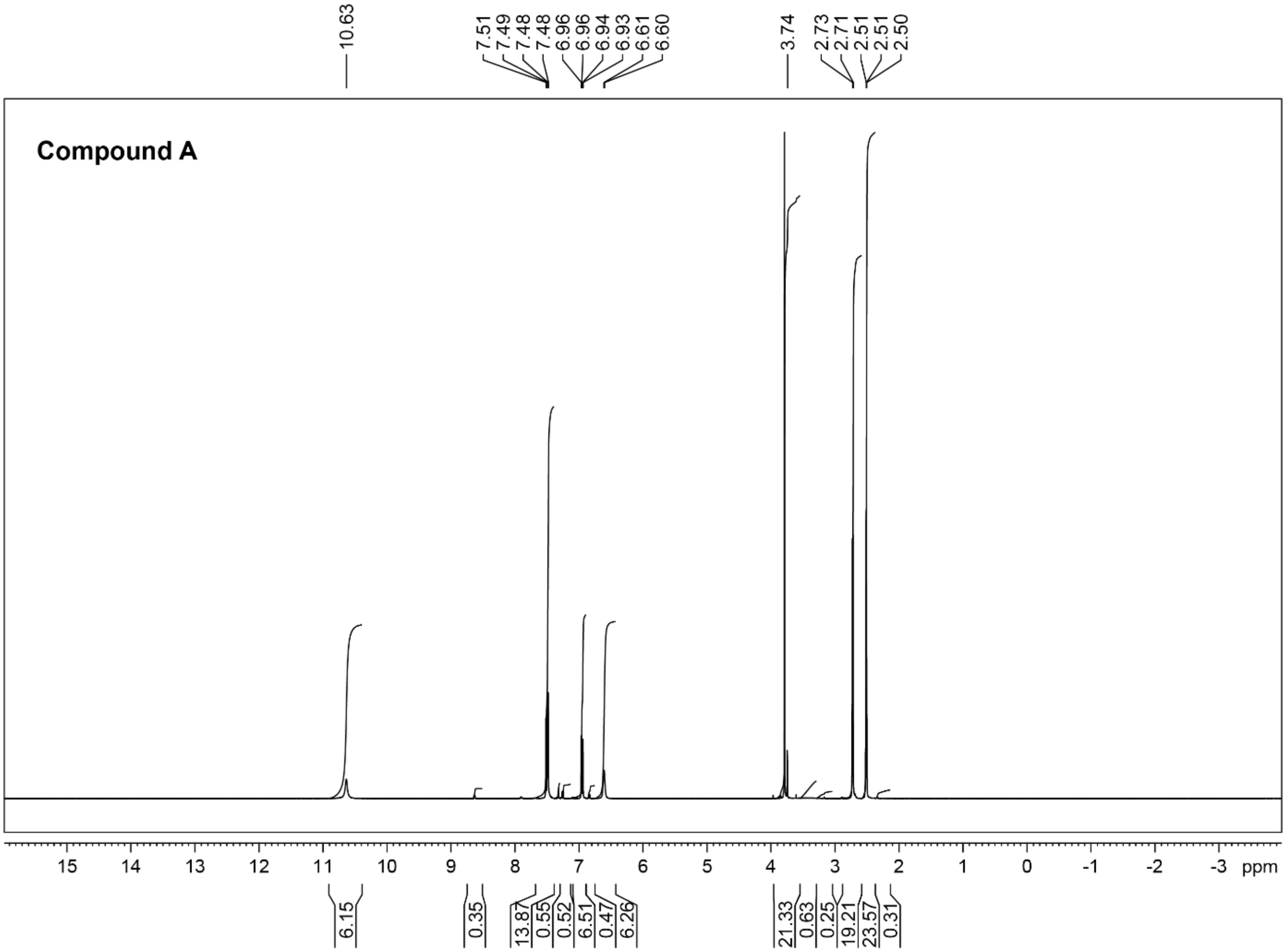

Synthesis Suppl. Figure 6. ^{1}H NMR of compound **A**.

1-(Benzo[*d*]thiazol-2-yl)-3-ethylurea (A.1)

2-Aminobenzothiazole (250 mg, 1.66 mmol, 1.0 eq), triethylamine (0.464 mL, 3.33 mmol, 2.0 eq), and ethyl isocyanate (237 mg, 0.263 mmol, 2.0 eq) were dissolved in 3 mL toluene. The solution was heated to 90 °C with stirring for 1 h. After cooling to room temperature, the precipitate formed was filtered off, washed with diethyl ether and dried under reduced pressure to give urea **A.1** (306 mg, 1.38 mmol, 83%) as a colourless solid. ^{1}H and ^{13}C NMR data is in accordance with Panchaud *et al.*[14] Mp: 199 °C (decomposition) [197 °C[15], decomposition]. ^{1}H NMR (400 MHz, DMSO-d_6) δ 10.66 (s, 1H), 7.89 – 7.83 (m, 1H), 7.60 (d, *J* = 8.0 Hz, 1H), 7.35 (ddd, *J* = 8.3, 7.3, 1.3 Hz, 1H), 7.20 (td, *J* = 7.6, 1.1 Hz, 1H), 6.73 (s, 1H), 3.18 (qd, *J* = 7.1, 5.6 Hz, 2H), 1.09 (t, *J* = 7.2 Hz, 3H). ^{13}C NMR (101 MHz, DMSO-d_6) δ 191.8, 159.9, 153.7, 149.2, 131.4, 125.8, 122.6, 121.3, 119.7, 34.3, 15.2. HRMS (ESI): m/z = calculated for $C_{10}H_{12}N_3OS$ $[M+H]^+$: 222.0696; found: 222.0697. Purity (HPLC): 100% (λ = 210 nm), 100% (λ = 254 nm), Method 2a.

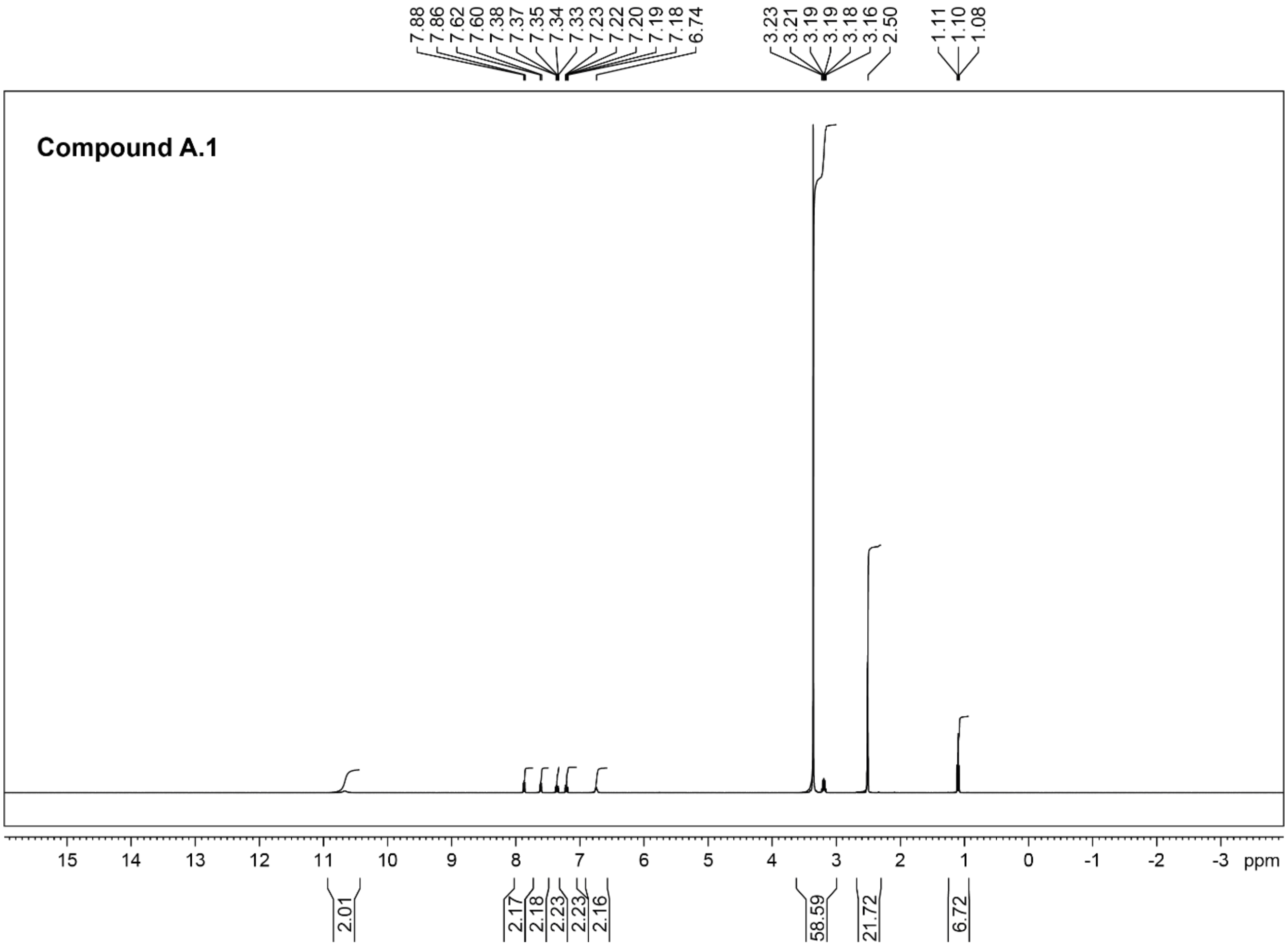

Synthesis Suppl. Figure 7. ^{1}H NMR of compound A.1.

2-(Dimethylamino)-*N*-(6-methoxybenzo[*d]*thiazol-2-yl)acetamide (A.2)

2-(Dimethylamino)acetamide **A.2** was prepared following General procedure F, using chloroacetamide **2** (513 mg, 2.00 mmol, 1.0 eq) and dimethylamine (2.2 mL, 2 M in THF, 4.4 mmol, 2.2 eq). The mixture was stirred for 1 h and the extraction was conducted with diethyl ether. The residue was suspended in DCM, the solid collected by filtration and washed with DCM to give product **A.2** (108 mg, 0.407 mmol, 42%), as a light yellow solid (108 mg, 0.407 mmol, 42%). Mp: 114 – 115 °C. ^{1}H NMR (400 MHz, DMSO-d_6) δ 11.90 (s, 1H), 7.63 (d, *J* = 8.8 Hz, 1H), 7.56 (d, *J* = 2.6 Hz, 1H), 7.02 (dd, *J* = 8.8, 2.6 Hz, 1H), 3.80 (s, 3H), 3.27 (s, 2H), 2.29 (s, 6H). ^{13}C NMR (101 MHz, DMSO-d_6) δ 169.4, 156.1, 155.5, 142.6, 132.8, 121.1, 114.9, 104.7, 61.3, 55.6, 45.1. HRMS (ESI): m/z = calculated for $C_{12}H_{16}N_3O_2S$ [M+H]$^+$: 266.0958; found: 266.0958. Purity (HPLC): > 99% (λ = 210 nm), > 98% (λ = 254 nm), Method 2a.

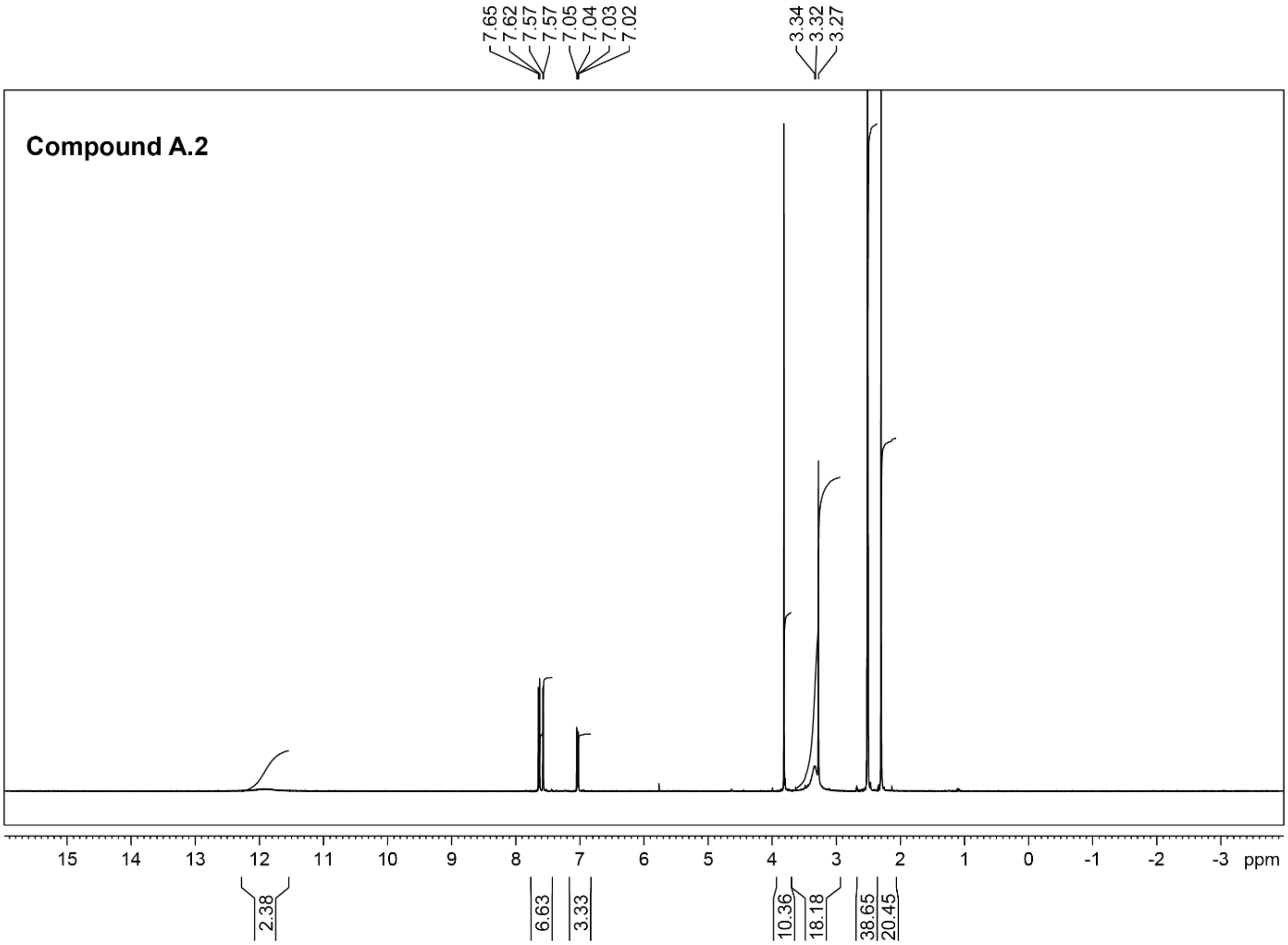

Synthesis Suppl. Figure 8. ^{1}H NMR of compound A.2.

***N*-(6-Methoxybenzo[*d*]thiazol-2-yl)-2-morpholinoacetamide (A.3)**

Amide **A.3** was prepared as described in Srivastava *et al.* from **2** (250 mg, 0.974 mmol, 1.0 eq) and morpholine (0.187 mL, 2.14 mmol, 2.2 eq).[16] Bright beige solid (200 mg, 0.650 mmol, 67%). Mp: 132 – 133 °C. ^{1}H NMR (400 MHz, DMSO-d_6) δ 11.96 (s, 1H), 7.63 (d, J = 8.8 Hz, 1H), 7.57 (d, J = 2.5 Hz, 1H), 7.03 (dd, J = 8.8, 2.6 Hz, 1H), 3.80 (s, 3H), 3.66 – 3.56 (m, 4H), 3.32 (s, 3H), 2.58 – 2.52 (m, 4H). ^{13}C NMR (101 MHz, DMSO-d_6) δ 169.0, 156.2, 155.4, 142.5, 132.8, 121.1, 115.0, 104.8, 66.1, 60.5, 55.6, 53.0. HRMS (ESI): m/z = calculated for $C_{14}H_{18}N_3O_3S$ [M+H]$^+$: 308.1064; found: 308.1064. Purity (HPLC): 100% (λ = 210 nm), 100% (λ = 254 nm), Method 2a.

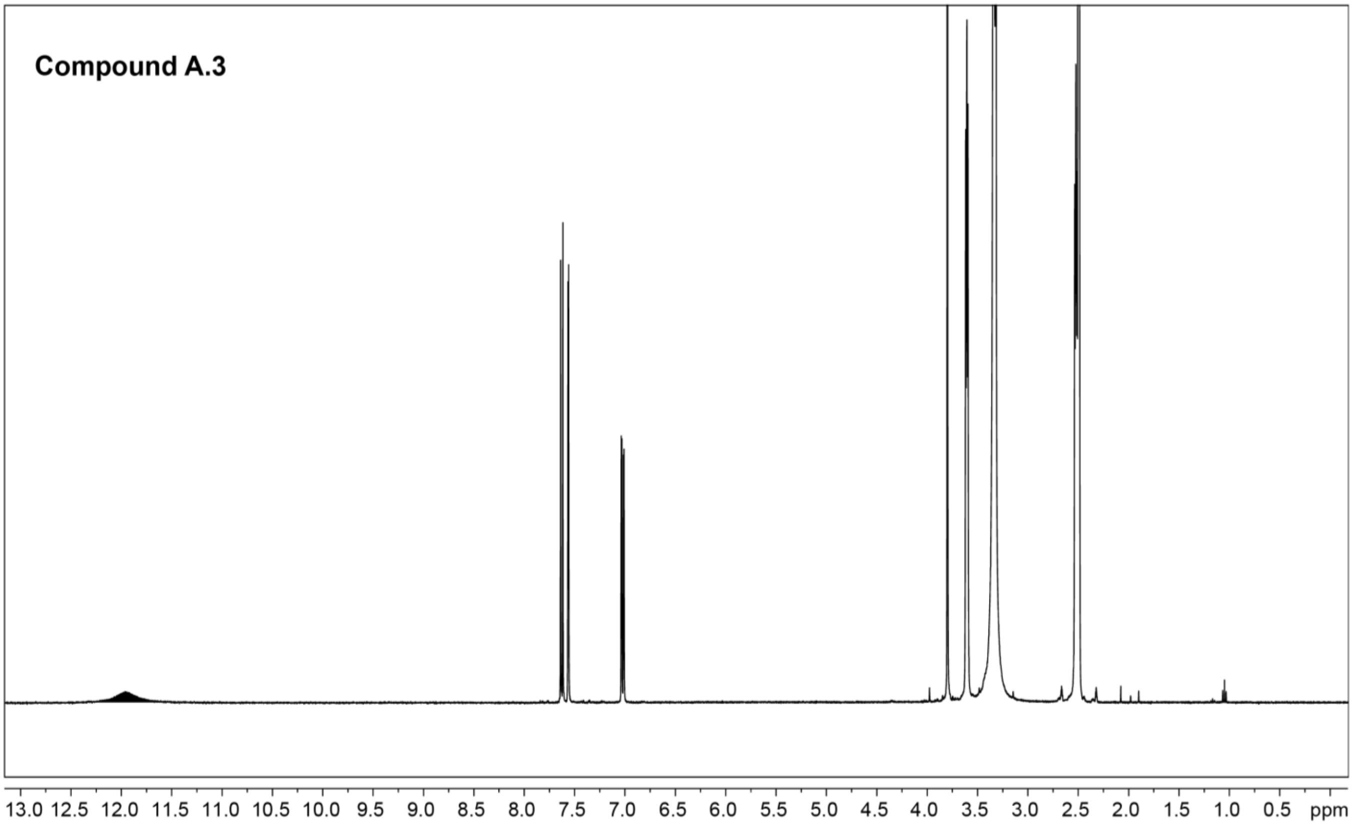

Synthesis Suppl. Figure 9. ^{1}H NMR of compound A.3.

1-(6-Methoxybenzo[*d*]thiazol-2-yl)-3-methylguanidine (A.4)

In a closed vial were added thiourea **1**(200 mg, 0.789 mmol, 1.0 eq), PbO (405 mg, 1.82 mmol) and NH_3 (1.6 mL, 7 N in MeOH, 14 eq). The mixture was heated to 100 °C for 4 h. After cooling to room temperature, 2 mL EtOH were added, the mixture was heated to 60 °C and the precipitate was collected by filtration from the warm mixture. The precipitate was then washed with diethyl ether to give guanidine derivative **A.4** (65 mg, 0.28 mmol, 35%) as colourless solid. Mp: 197 – 198 °C. ^{1}H NMR (400 MHz, DMSO-d_6) δ 7.58 (s, 3H), 7.35 (d, *J* = 8.8 Hz, 1H), 7.28 (d, *J* = 2.6 Hz, 1H), 6.84 (dd, *J* = 8.8, 2.7 Hz, 1H), 3.74 (s, 3H), 2.76 (d, *J* = 4.8 Hz, 3H). ^{13}C NMR (101 MHz, DMSO-d_6) δ 172.2, 154.8, 146.0, 131.3, 118.8, 113.0, 105.0, 55.5, 27.6. HRMS (ESI): m/z = calculated for $C_{10}H_9N_2O_2S$ [M-H]$^-$: 235.0659; found: 235.0659. Purity (HPLC): 100% (λ = 210 nm), 100% (λ = 254 nm), Method 1a.

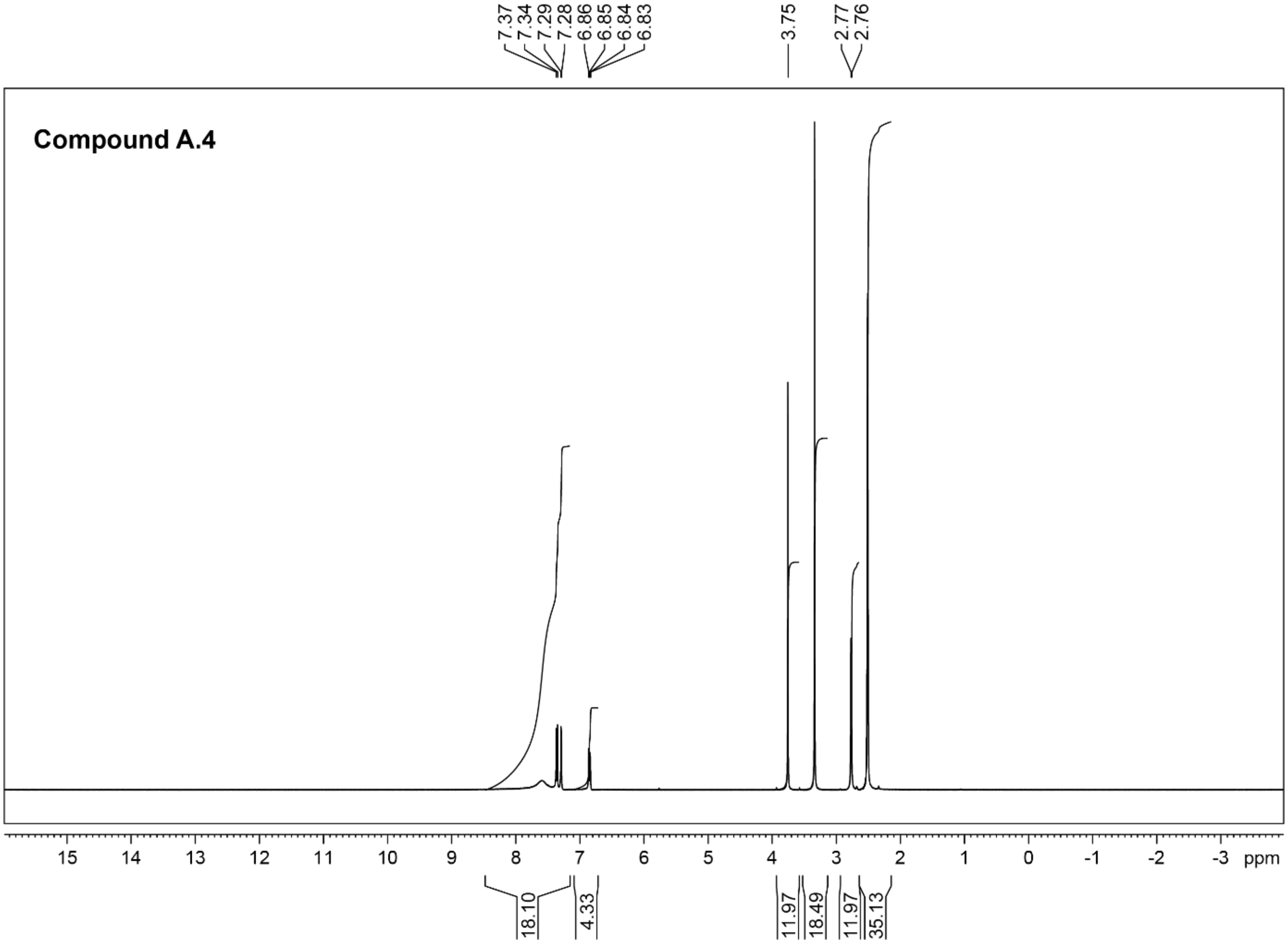

Synthesis Suppl. Figure 10. ^{1}H NMR of compound A.4.

Methyl 3-(benzo[*d*]thiazol-2-ylamino)-3-oxopropanoate (A.5)

Amide **A.5** was prepared following General procedure E, using 2-aminobenzothiazole (**xx**, 250 mg, 1.66 mmol, 1.0 eq) and methyl malonyl chloride in DCM for 18 h. After extraction, the residue was treated with DCM and the insoluble material was filtered off, washed with DCM and dried under reduced pressure to give **A.5** (162 mg, 0.647 mmol, 39%) as a colourless solid. Mp: 171 °C. ^{1}H NMR (400 MHz, DMSO-d_6) δ 12.56 (s, 1H), 7.99 (ddd, *J* = 7.9, 1.3, 0.6 Hz, 1H), 7.76 (dt, *J* = 8.0, 1.0 Hz, 1H), 7.45 (ddd, *J* = 8.3, 7.2, 1.3 Hz, 1H), 7.32 (ddd, *J* = 8.2, 7.2, 1.2 Hz, 1H), 3.68 (s, 2H), 3.67 (s, 3H). ^{13}C NMR (101 MHz, DMSO-d_6) δ 167.4, 165.3, 157.6, 148.5, 131.5, 126.2, 123.8, 121.8, 120.7, 52.2, 42.2. HRMS (ESI): m/z = calculated for $C_{11}H_{11}N_2O_3S$ $[M+H]^+$: 251.0485; found: 251.0486. Purity (HPLC): > 99% (λ = 210 nm), > 98% (λ = 254 nm), Method 2b.

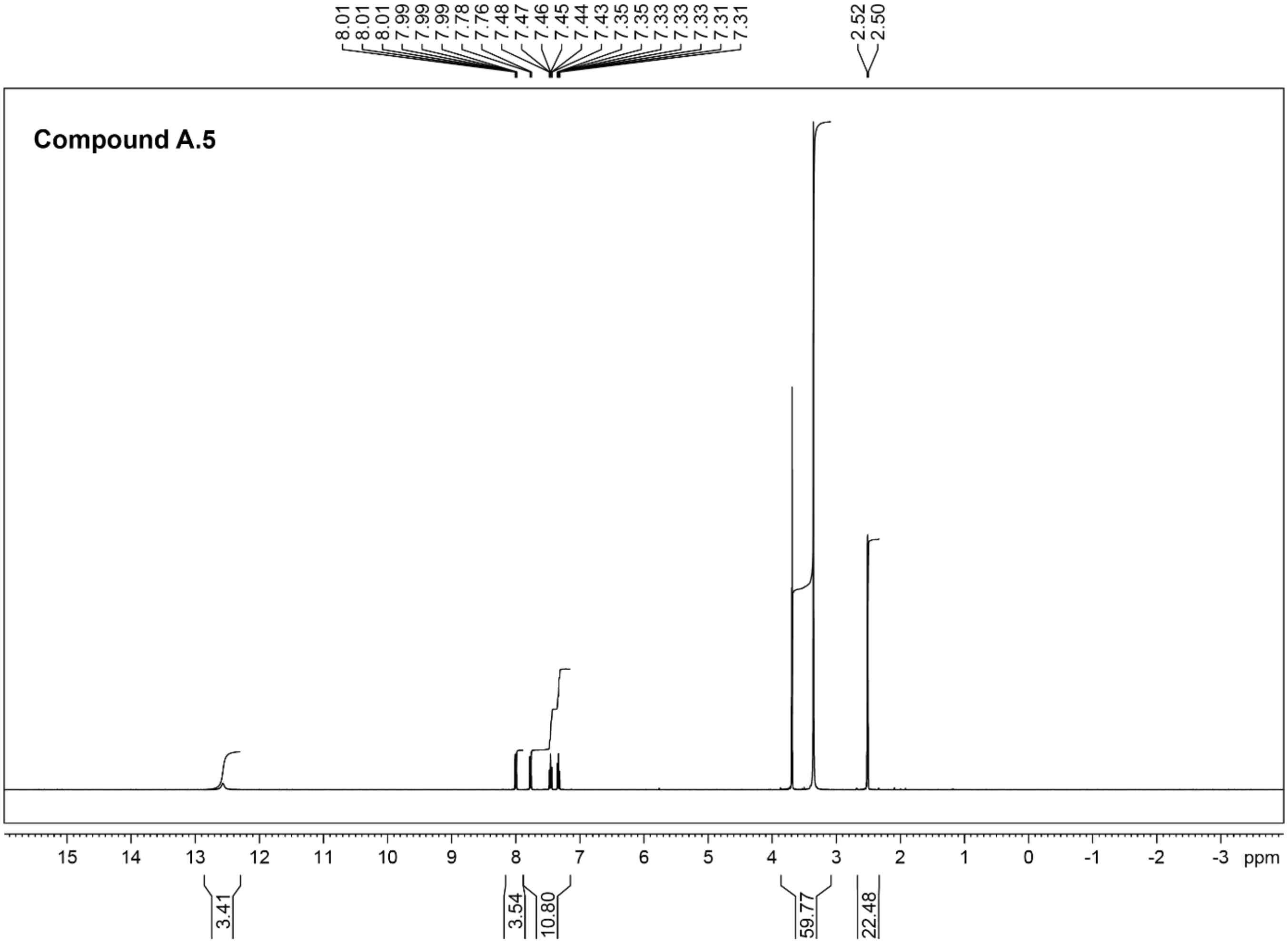

Synthesis Suppl. Figure 11. ^{1}H NMR of compound A.5.

_N_-(Benzo[_d_]thiazol-2-yl)acetamide (A.6)

Amide **A.6** was prepared following General procedure D, using 2-aminobenzothiazole (250 mg, 1.66 mmol, 1.0 eq). Purification by recrystallization from DCM gave amide **A.6** (110 mg, 0.572 mmol, 34%) as a colourless solid. ^{1}H and ^{13}C NMR data is in accordance with Al-Janabi *et al.*[17] Mp: 182 – 183 °C [184 – 187 °C[17]]. ^{1}H NMR (400 MHz, DMSO-d_6) δ 12.32 (s, 1H), 7.99 – 7.92 (m, 1H), 7.73 (dt, *J* = 8.1, 0.8 Hz, 1H), 7.42 (ddd, *J* = 8.3, 7.2, 1.3 Hz, 1H), 7.33 – 7.25 (m, 1H), 2.20 (s, 3H). ^{13}C NMR (101 MHz, DMSO-d_6) δ 169.4, 158.0, 148.5, 131.4, 126.1, 123.5, 121.7, 120.5, 22.8. HRMS (ESI): m/z = calculated for $C_9H_7N_2OS$ $[M-H]^-$: 191.0284; found: 191.0284. Purity (HPLC): 100% (λ = 210 nm), 100% (λ = 254 nm), Method 2b.

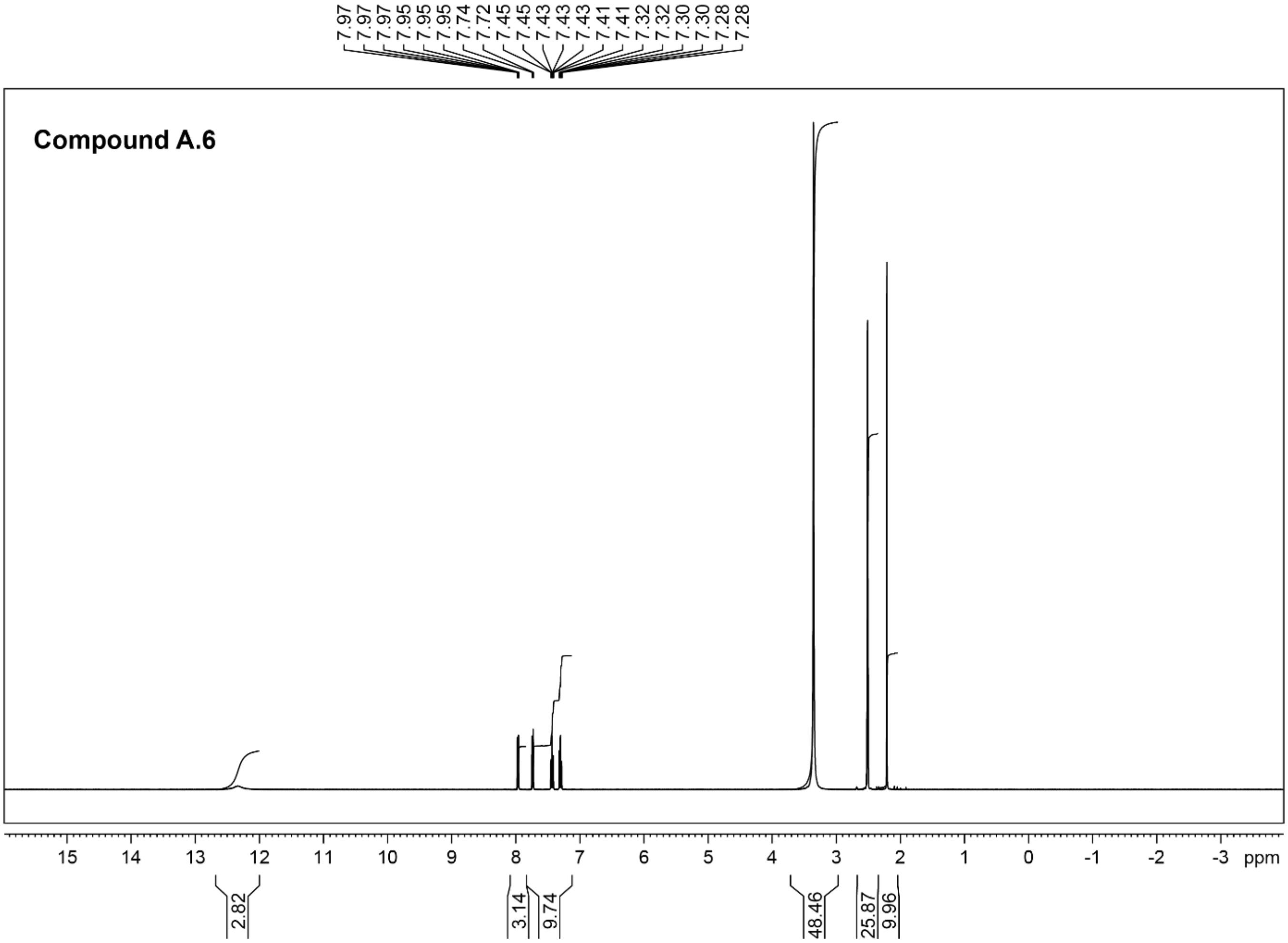

Synthesis Suppl. Figure 12. ^{1}H NMR of compound A.6.

***N*-(Benzo[*d*]thiazol-2-yl)pivalamide (A.7)**

Amide **A.7** was prepared following General procedure E, using 2-aminobenzothiazole (250 mg, 1.66 mmol, 1.0 eq) and pivaloyl chloride in DCM for 72 h. After extraction, the residue was purified by flash column chromatography (9.75:0.25 DCM/EtOH) to give pivalamide **A.7** (144 mg, 0.616 mmol, 37%) as a low-melting beige solid. ^{1}H NMR (400 MHz, DMSO-d_6) δ 12.04 (s, 1H), 7.97 (ddd, *J* = 7.9, 1.3, 0.6 Hz, 1H), 7.73 (dt, *J* = 8.1, 0.9 Hz, 1H), 7.43 (ddd, *J* = 8.2, 7.2, 1.3 Hz, 1H), 7.30 (ddd, *J* = 8.2, 7.2, 1.1 Hz, 1H), 1.27 (s, 9H). ^{13}C NMR (101 MHz, DMSO-d_6) δ 177.5, 158.8, 148.5, 131.5, 126.1, 123.4, 121.6, 120.3, 39.1, 26.5. HRMS (ESI): m/z = calculated for $C_{12}H_{15}N_2OS$ $[M+H]^+$: 235.0900; found: 235.0902. Purity (HPLC): 100% (λ = 210 nm), 100% (λ = 254 nm), Method 2b.

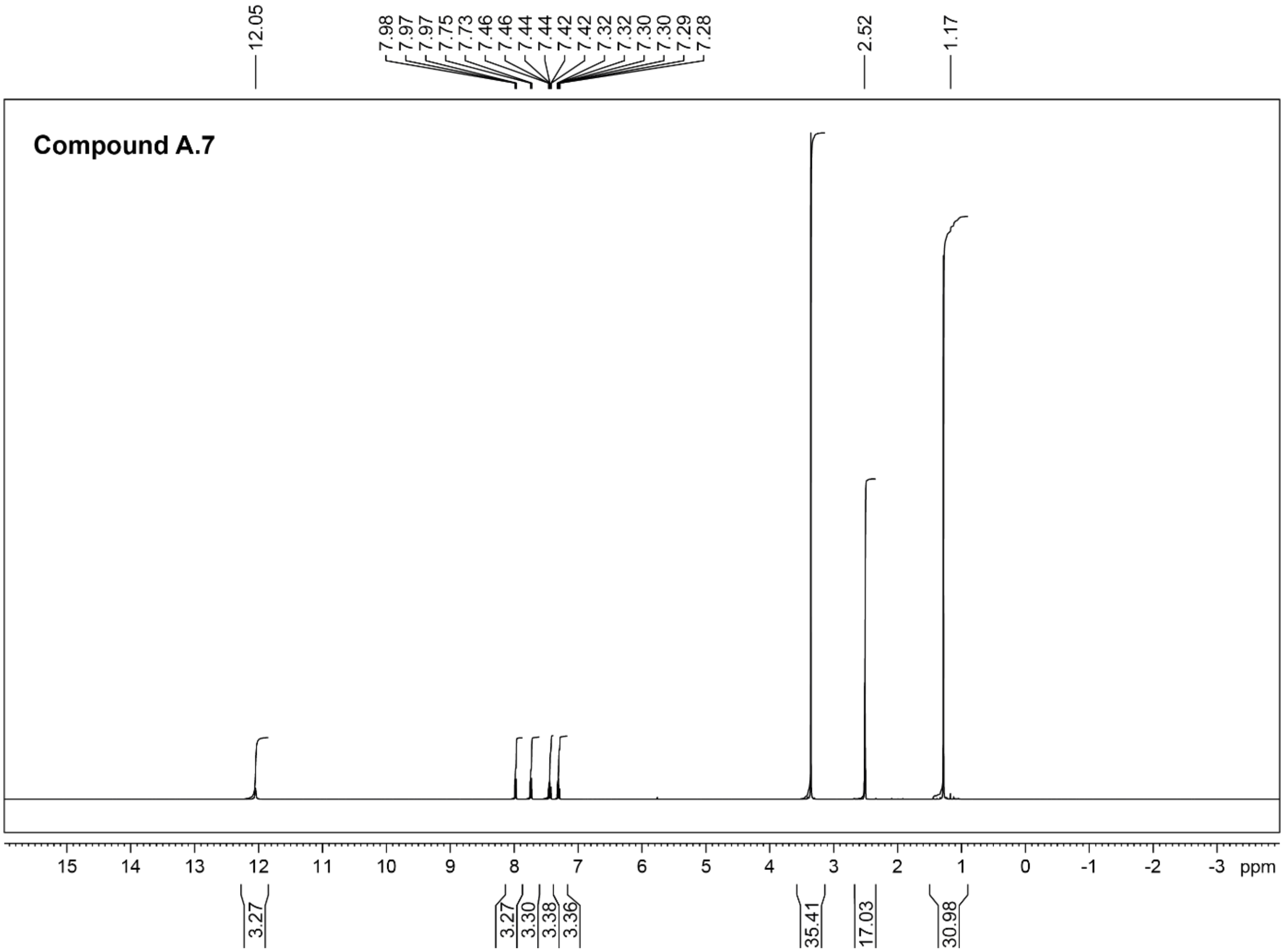

Synthesis Suppl. Figure 13. ^{1}H NMR of compound A.7.

N-(6-Bromobenzo[d]thiazol-2-yl)pivalamide (A.8)

Amide **A.8** was prepared following General procedure E, using 2-amino-6-bromobenzothiazole (250 mg, 1.09 mmol, 1.0 eq) and pivaloyl chloride in DCM for 72 h. After extraction, the residue was purified by flash column chromatography (9.75:0.25 DCM/EtOH) to give pivalamide **A.8** (62 mg, 0.20 mmol, 18%) as light beige solid. Mp: 129 – 130 °C. ^{1}H NMR (400 MHz, DMSO-d_6) δ 12.15 (s, 1H), 8.24 (d, J = 2.0 Hz, 1H), 7.66 (d, J = 8.6 Hz, 1H), 7.57 (dd, J = 8.6, 2.0 Hz, 1H), 1.27 (s, 9H). ^{13}C NMR (101 MHz, DMSO-d_6) δ 177.8, 159.6, 147.7, 133.7, 129.1, 124.2, 121.9, 115.3, 39.1, 26.5. HRMS (ESI): m/z = calculated for $C_{12}H_{14}N_2OS^{79}Br$ [M+H]$^+$: 313.0005; found: 313.0005. Purity (HPLC): 100% (λ = 210 nm), 100% (λ = 254 nm), Method 2b.

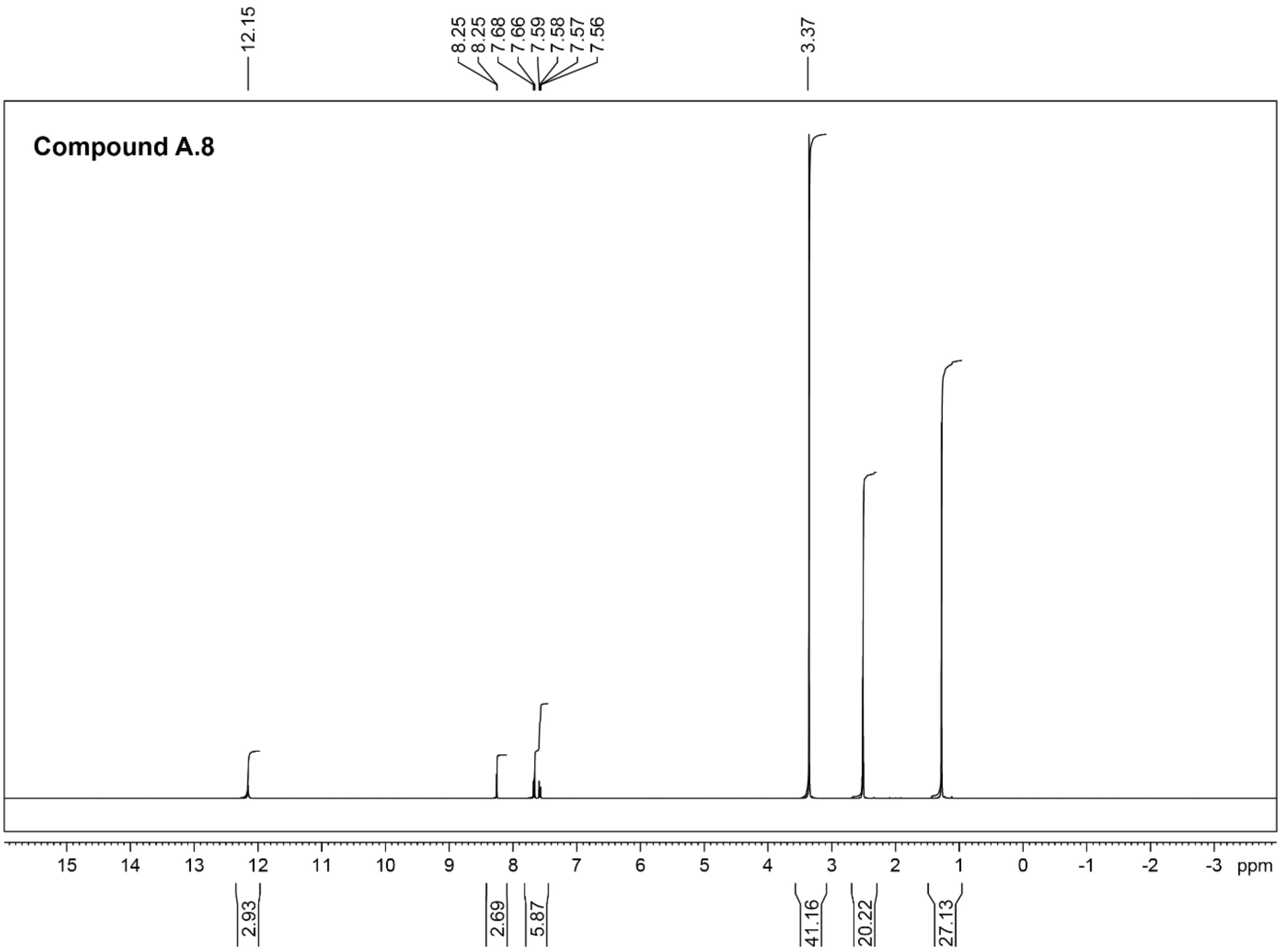

Synthesis Suppl. Figure 14. ^{1}H NMR of compound A.8.

1-(6-Hydroxybenzo[*d*]thiazol-2-yl)-3-methylurea (A.9)

6-Hydroxybenzothiazole **A.9** was prepared following General procedure C from 6-methoxybenzothiazole **A** (500 mg, 2.11 mmol, 1.0 eq). Grey solid (292 mg, 1.31 mmol, 62%). Mp: 347 °C (decomposition), 281 – 283 °C (melting). ^{1}H NMR (400 MHz, DMSO-d_6) δ 10.54 (s, 1H), 9.38 (s, 1H), 7.39 (d, *J* = 8.6 Hz, 1H), 7.18 (d, *J* = 2.5 Hz, 1H), 6.79 (dd, *J* = 8.6, 2.5 Hz, 1H), 6.67 (d, *J* = 5.0 Hz, 1H), 2.70 (d, *J* = 4.6 Hz, 3H). ^{13}C NMR (101 MHz, DMSO-d_6) δ 157.2, 154.5, 153.4, 142.2, 132.5, 120.1, 114.5, 106.5, 26.3. HRMS (ESI): m/z = calculated for $C_9H_8N_3O_2S$ [M-H]$^-$: 222.0342; found: 222.0342. Purity (HPLC): 100% (λ = 210 nm), 100% (λ = 254 nm), Method 4.

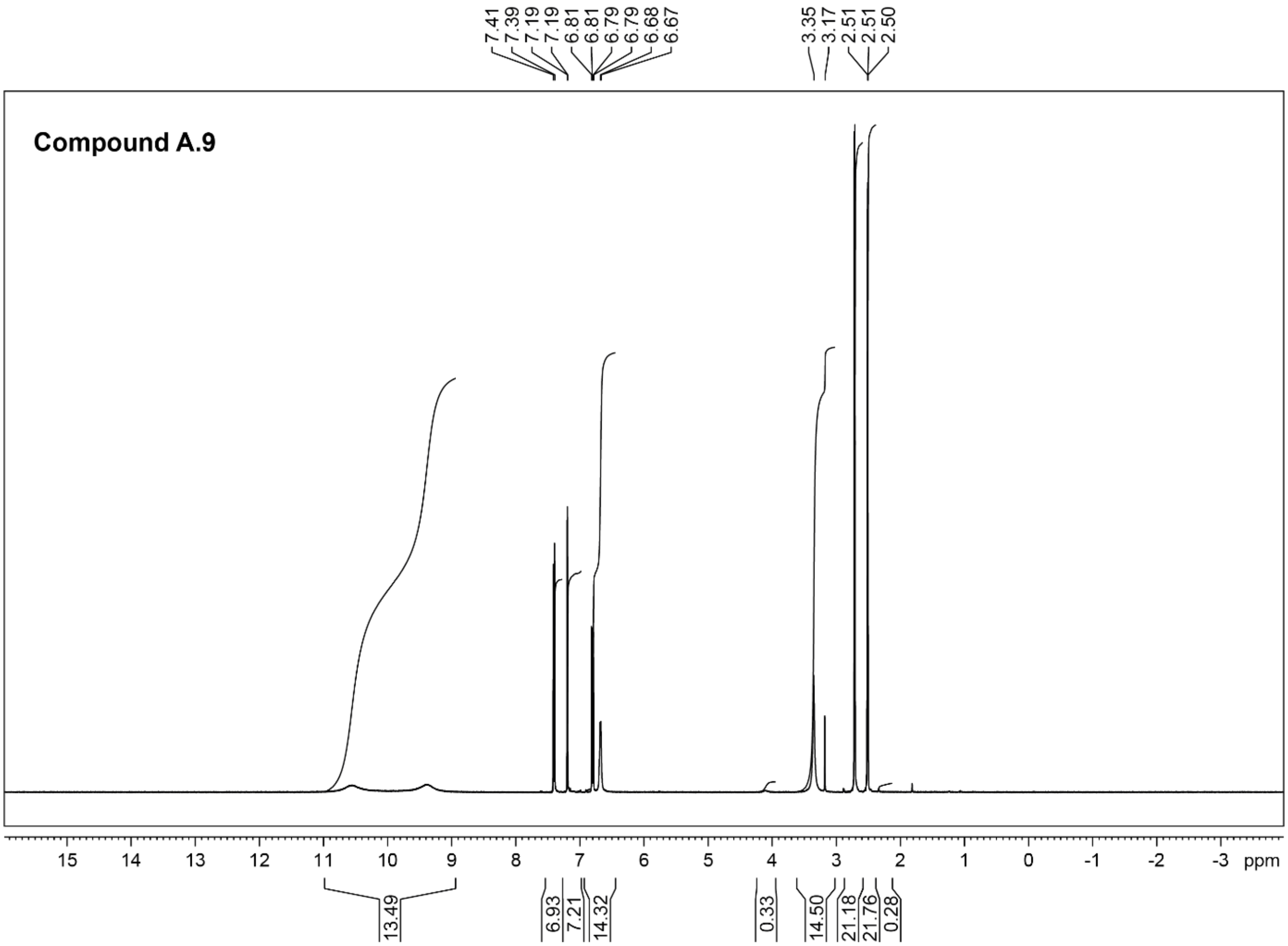

Synthesis Suppl. Figure 15. ^{1}H NMR of compound A.9.

1-(4,6-Dimethoxybenzo[*d*]thiazol-2-yl)-3-methylurea (A.11)

Methylurea **A.11** was prepared following General procedure B, using 2-benzothiazole **3** (190 mg, 0.904 mmol, 1.0 eq). Colourless solid (46 mg, 0.17 mmol, 19%). Mp: 208 – 209 °C. ^{1}H NMR (400 MHz, $CDCl_3$) δ 11.34 (s, 1H), 6.89 (d, *J* = 2.2 Hz, 1H), 6.54 (d, *J* = 2.2 Hz, 1H), 6.29 (s, 1H), 3.96 (s, 3H), 3.87 (s, 3H), 2.87 (d, *J* = 4.6 Hz, 3H), 1.61 (s, 1H). ^{13}C NMR (101 MHz, $CDCl_3$) δ 160.3, 157.3, 155.1, 150.7, 133.9, 132.8, 98.2, 96.2, 56.1, 56.0, 27.0. HRMS (EI): *m/z* = calculated for $C_{11}H_{13}N_3O_3S$ $[M]^{\cdot+}$: 267.0673; found: 267.0669. Purity (HPLC): > 99% (λ = 210 nm), > 98% (λ = 254 nm), Method 1a.

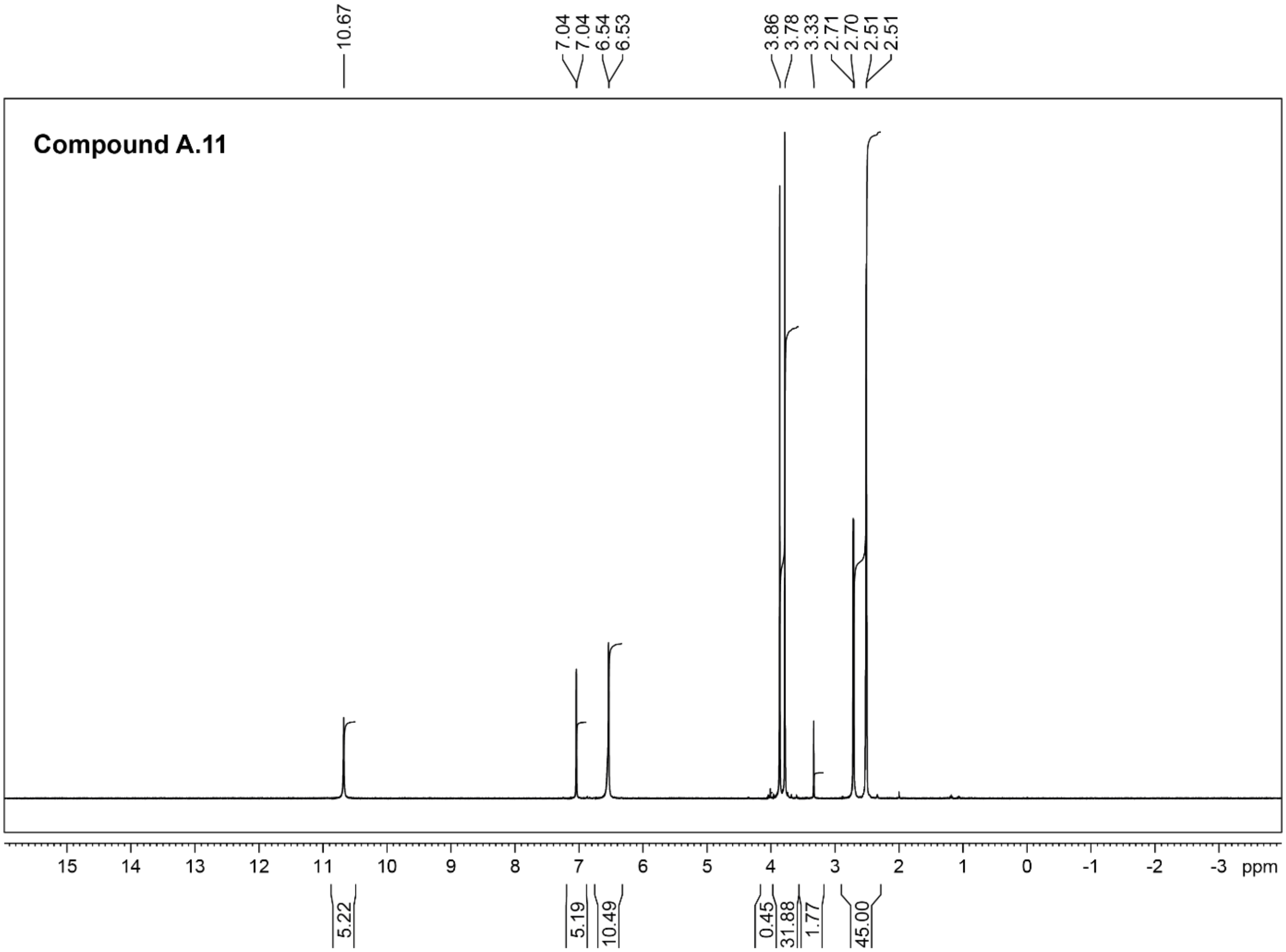

Synthesis Suppl. Figure 16. ^{1}H NMR of compound A.11.

2-(Dimethylamino)-*N*-(6-hydroxybenzo[*d*]thiazol-2-yl)acetamide (A.12)

4,6-Dihydroxybenzothiazole **A.12** was prepared following General procedure C from 4,6-dimethoxybenzothiazole **A.2** (120 mg, 0.452 mmol, 1.0 eq). Off-white solid (70 mg, 0.28 mmol, 62%). Mp: 199 – 200 °C. ^{1}H NMR (400 MHzDMSO-d_6) δ 7.53 (d, *J* = 8.7 Hz, 1H), 7.27 (d, *J* = 2.4 Hz, 1H), 6.88 (dd, *J* = 8.7, 2.5 Hz, 1H), 3.26 (s, 2H), 2.29 (s, 6H), 1.99 (s, 1H). ^{13}C NMR (101 MHz, DMSO-d_6) δ 169.2, 154.5, 154.2, 141.5, 132.8, 121.1, 115.2, 106.5, 61.3, 45.1. HRMS (ESI): m/z = calculated for $C_{11}H_{14}N_3O_2S$ [M+H]$^+$: 252.0802; found: 252.0800. Purity (HPLC): > 80% (λ = 210 nm), > 80% (λ = 254 nm), Method 3c.

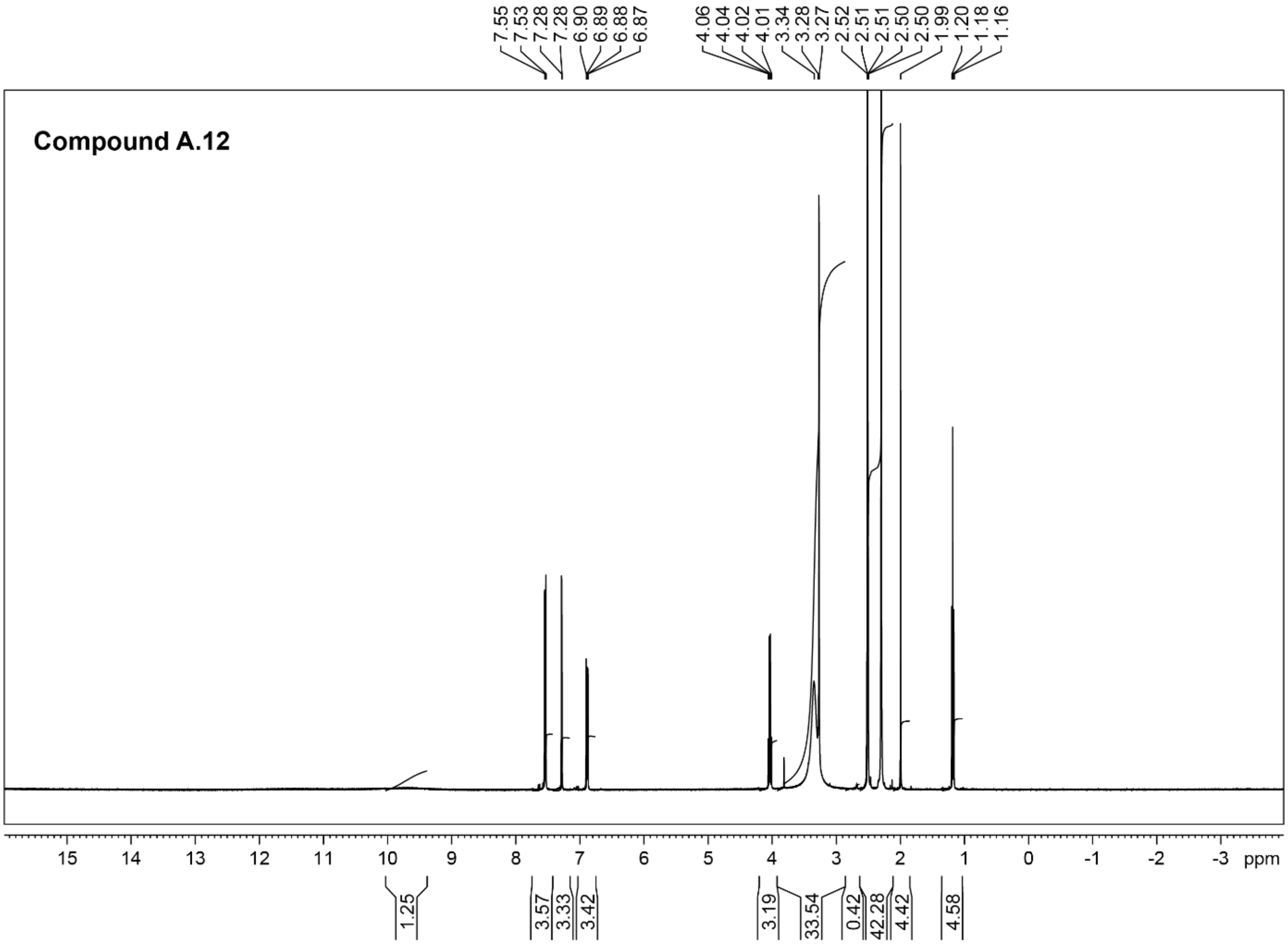

Synthesis Suppl. Figure 17. ^{1}H NMR of compound A.12.

1-(4,6-Dihydroxybenzo[*d*]thiazol-2-yl)-3-methylurea (A.13)

4,6-Dihydroxybenzothiazole **A.13** was prepared following General procedure C from 4,6-dimethoxybenzothiazole **A.11** (64 mg, 0.24 mmol, 1.0 eq). The crude product was purified by flash column chromatography (DCM → 9:1 DCM/EtOH) to give **A.13** (25 mg, 0.084 mmol, 35%) as an light yellow solid. Mp: 234 – 236 °C. ^{1}H NMR (400 MHz, DMSO-d_6) δ 10.37 (s, 1H), 9.50 (s, 1H), 9.19 (s, 1H), 7.07 (s, 1H), 6.61 (d, J = 2.3 Hz, 1H), 6.28 (d, J = 2.2 Hz, 1H), 2.72 (d, J = 4.6 Hz, 3H). ^{13}C NMR (101 MHz, DMSO-d_6) δ 154.9, 154.4, 149.3, 133.1, 131.6, 130.4, 100.8, 97.2, 26.3. HRMS (ESI): m/z = calculated for $C_9H_8N_3O_3S$ [M-H]$^-$: 238.0291; found: 238.0292. Purity (HPLC): 100% (λ = 210 nm), > 99% (λ = 254 nm), Method 3b.

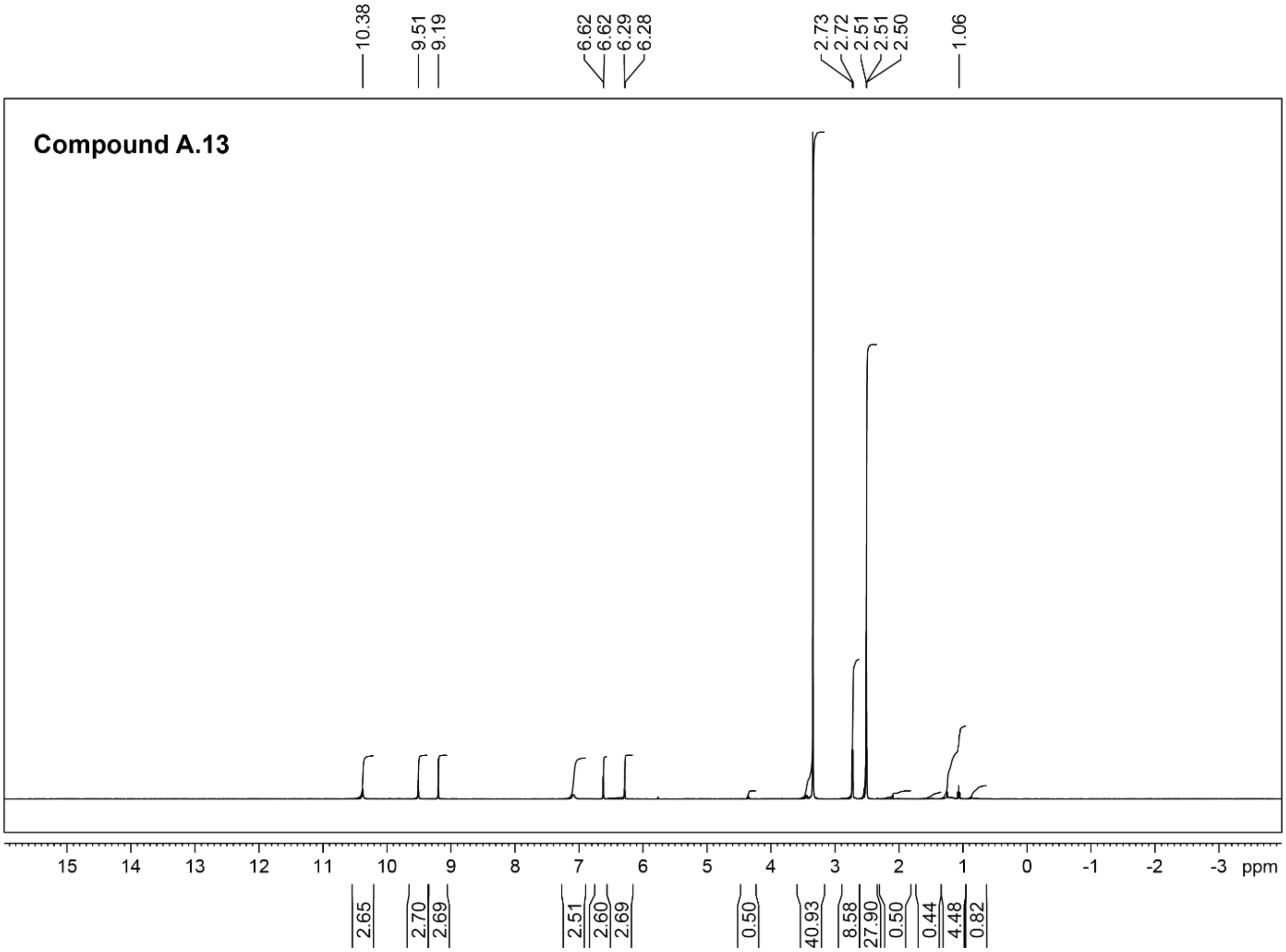

Synthesis Suppl. Figure 18. ^{1}H NMR of compound A.13.

2-(Dimethylamino)-*N*-(6-nitrobenzo[*d*]thiazol-2-yl)acetamide (A.14)

2-(Dimethylamino)acetamide **A.14** was prepared following General procedure F, using chloroacetamide **5** (300 mg, 1.10 mmol, 1.0 eq) and dimethylamine (1.21 mL, 2 M in THF, 2.43 mmol, 2.2 eq). The mixture was stirred for 18 h, the formed precipitate was filtered and the solid washed with DCM to give product **A.14** (268 mg, 0.956 mmol, 87%) as a yellow solid. Mp: 209 – 210 °C [204 – 206 °C[13]]. ^{1}H NMR (400 MHz, DMSO-d_6) δ 8.99 (d, *J* = 2.4 Hz, 1H), 8.25 (dd, *J* = 8.9, 2.5 Hz, 1H), 7.84 (d, *J* = 9.0 Hz, 1H), 3.45 (s, 2H), 2.39 (s, 6H). ^{13}C NMR (101 MHz, DMSO-d_6) δ 170.1, 164.4, 153.8, 142.7, 132.3, 121.7, 120.3, 118.9, 60.8, 44.7. HRMS (ESI): *m/z* = calculated for $C_{11}H_{11}N_4O_3S$ [M-H]$^-$: 279.0557; found: 279.0557. Purity (HPLC): > 88% (λ = 210 nm), > 92% (λ = 254 nm), Method 3a.

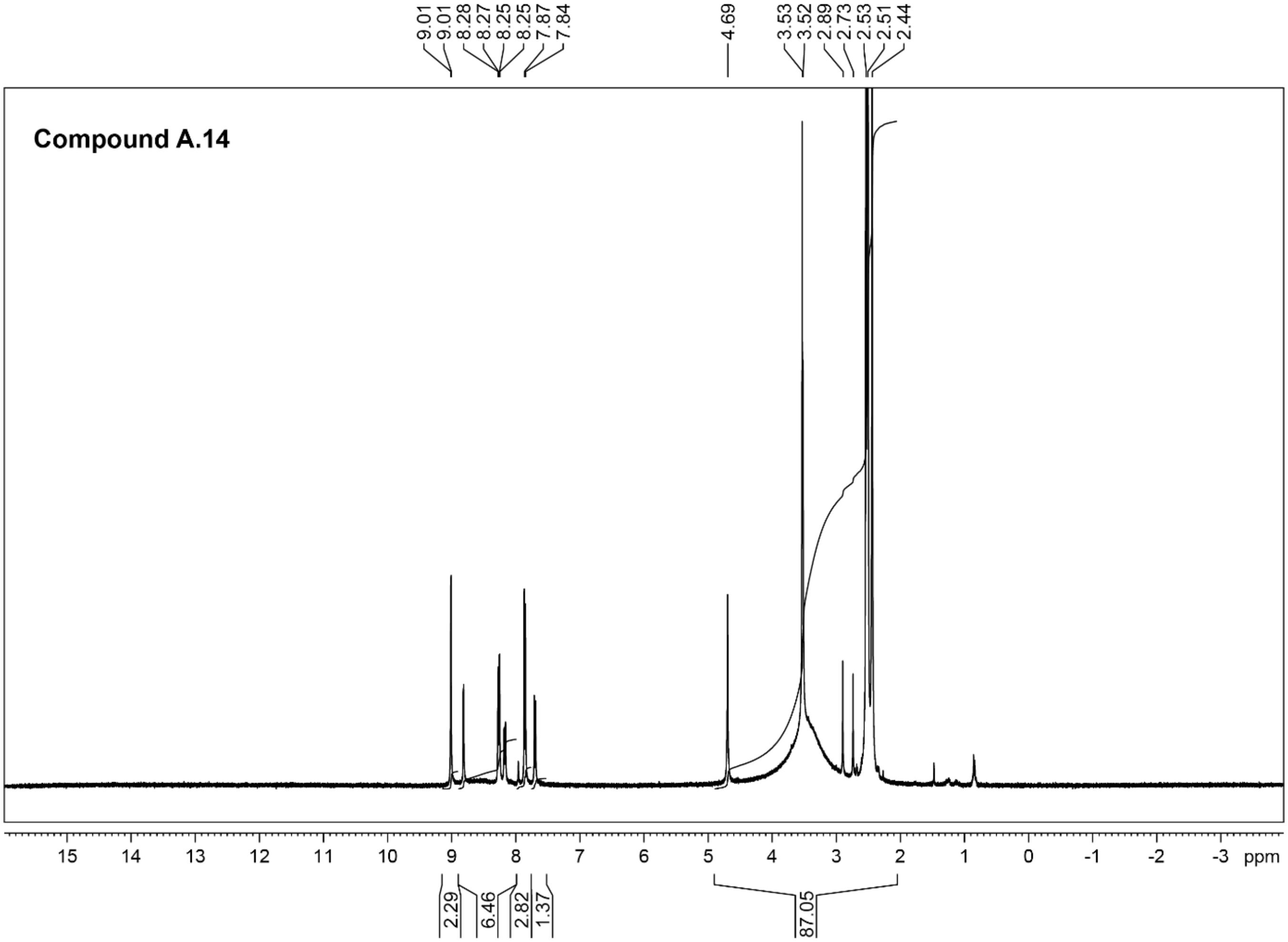

Synthesis Suppl. Figure 19. ^{1}H NMR of compound A.14.

N-(4,6-Dimethoxybenzo[d]thiazol-2-yl)-2-(dimethylamino)acetamide (A.15)

2-(Dimethylamino)acetamide **A.15** was prepared following General procedure F, using chloroacetamide **6** (200 mg, 0.697 mmol, 1.0 eq) and dimethylamine (0.767 mL, 2 M in THF, 1.53 mmol, 2.2 eq). The mixture was stirred for 18 h and the extraction was conducted with diethyl ether. The residue was purified by flash column chromatography (99.5:0.5 DCM/MeOH), yielding product **A.15** (142 mg, 0.481 mmol, 69%) as an off-white solid. Mp: 133 – 134 °C. ^{1}H NMR (400 MHz, DMSO-d_6) δ 12.00 (s, 1H), 7.11 (d, J = 2.3 Hz, 1H), 6.59 (d, J = 2.3 Hz, 1H), 3.88 (s, 3H), 3.80 (s, 3H), 3.24 (s, 2H), 2.28 (s, 6H). ^{13}C NMR (101 MHz, DMSO-d_6) δ 169.2, 157.3, 153.8, 152.2, 133.5, 132.7, 98.0, 95.7, 61.2, 55.8, 55.7, 45.1. HRMS (ESI): m/z = calculated for $C_{13}H_{18}N_3O_3S$ [M+H]$^+$: 296.1062; found: 296.1064. Purity (HPLC): > 99% (λ = 210 nm), 100% (λ = 254 nm), Method 1a.

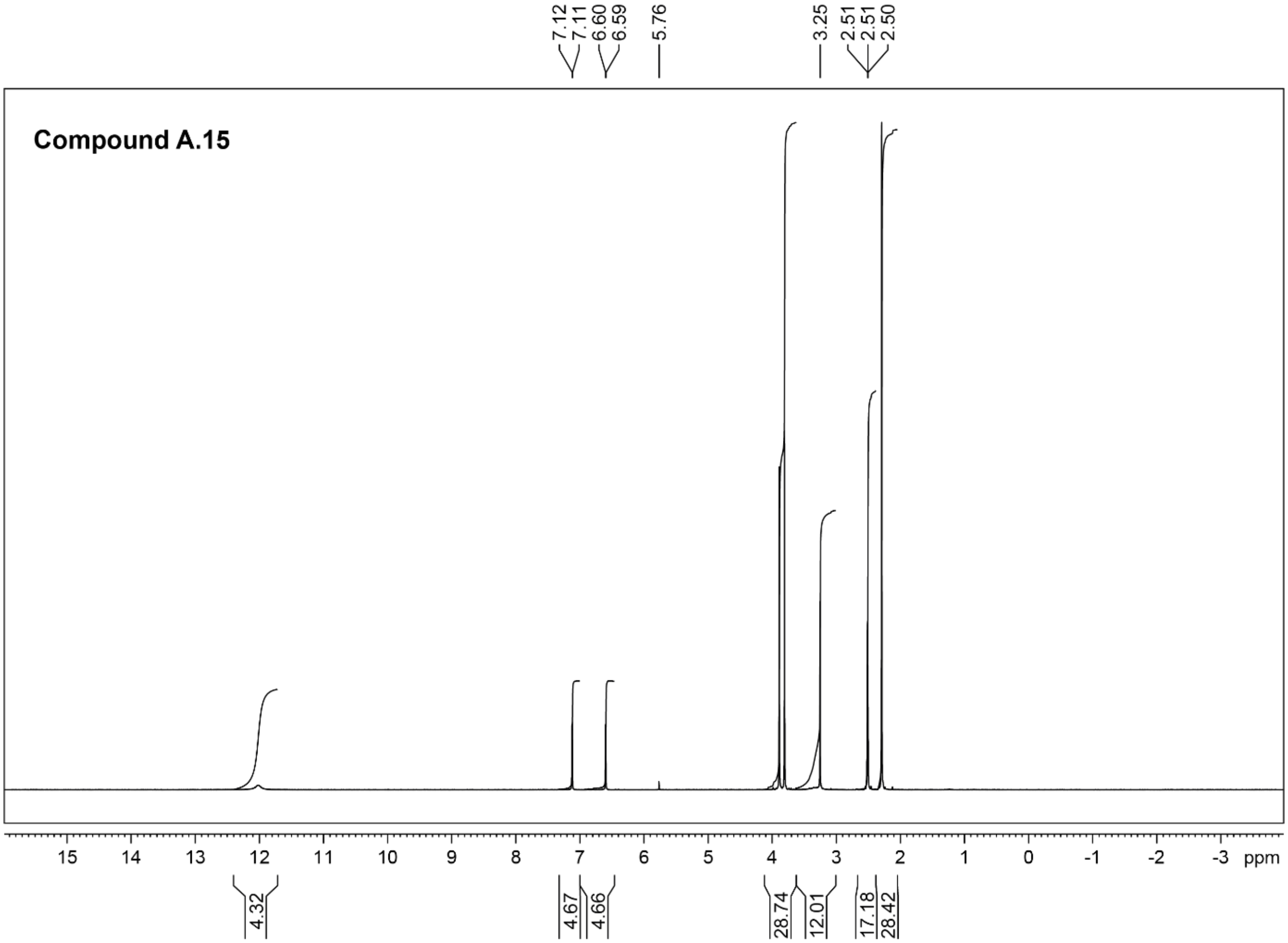

Synthesis Suppl. Figure 20. ^{1}H NMR of compound A.15.

***N*-(6-Bromobenzo[*d*]thiazol-2-yl)-2-(dimethylamino)acetamide (A.16)**

2-(Dimethylamino)acetamide **A.16** was prepared following General procedure F, using chloroacetamide **4** (100 mg, 0.327 mmol, 1.0 eq) and dimethylamine (0.36 mL, 2 M in THF, 0.72 mmol, 2.2 eq). The mixture was stirred for 18 h and the extraction was conducted with diethyl ether. The residue was recrystallised from isohexanes to give product **A.16** (39 mg, 0.12 mmol, 37%) as a beige solid. Mp: 97 – 98 °C. ^{1}H NMR (400 MHz, DMSO-d_6) δ 8.24 (d, *J* = 1.9 Hz, 1H), 7.67 (d, *J* = 8.6 Hz, 1H), 7.56 (dd, *J* = 8.6, 2.1 Hz, 1H), 3.30 (s, 2H), 2.29 (s, 6H). ^{13}C NMR (101 MHz, DMSO-d_6) δ 170.0, 158.5, 147.7, 133.7, 129.1, 124.2, 122.1, 115.4, 61.3, 45.0. HRMS (EI): m/z = calculated for $C_{11}H_{12}N_3OS^{79}BrCl$ $[M]^{\cdot+}$: 312.9878; found: 312.9879. Purity (HPLC): > 94% (λ = 210 nm), > 98% (λ = 254 nm), Method 1a.

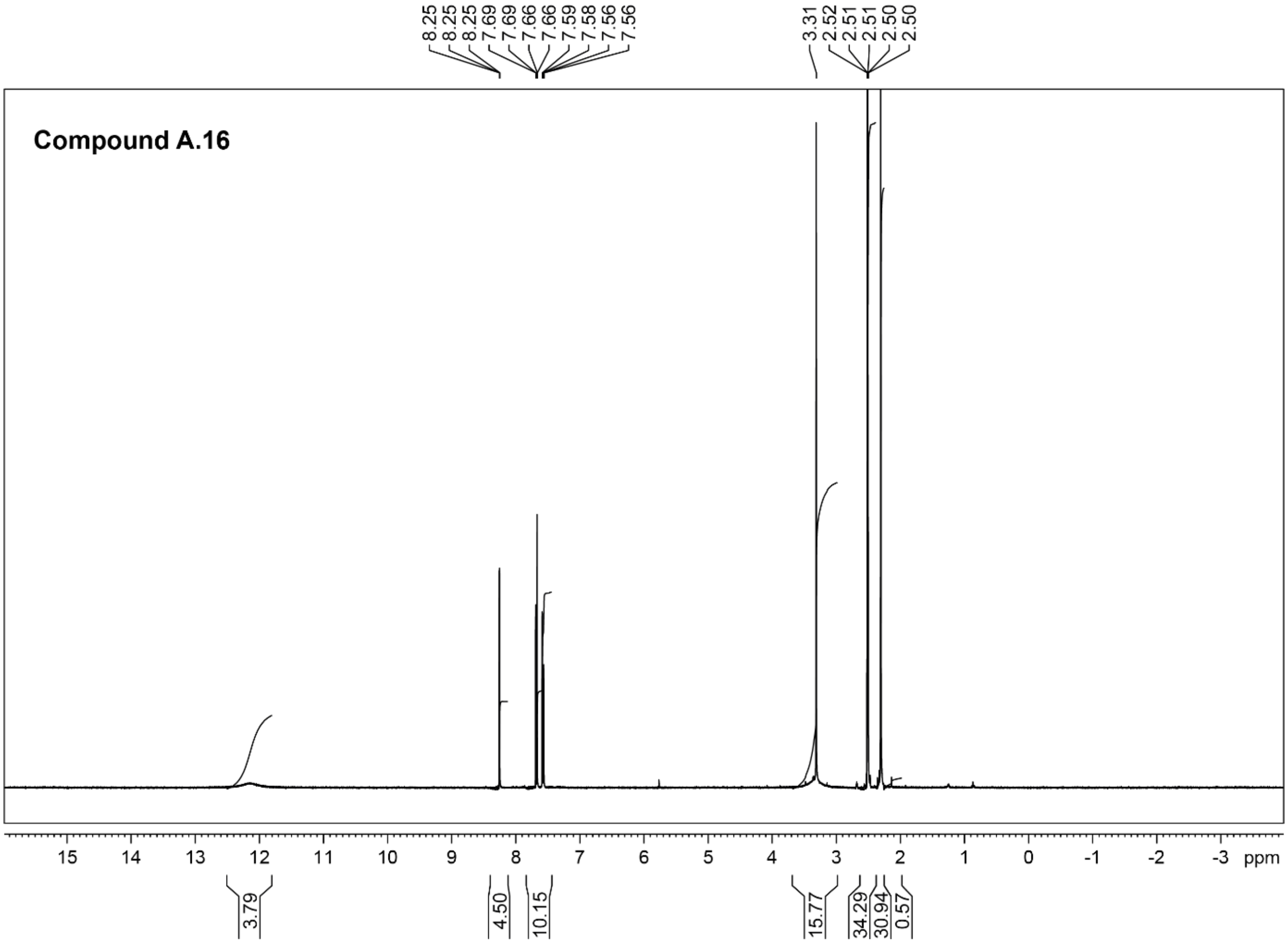

Synthesis Suppl. Figure 21. ^{1}H NMR of compound A.16.

N-(Benzo[d]thiazol-2-ylmethyl)-2-(dimethylamino)acetamide (A.17)

2-(Dimethylamino)acetamide **A.17** was prepared following General procedure F, using chloroacetamide **7** (60 mg, 0.25 mmol, 1.0 eq) and dimethylamine (0.274 mL, 2 M in THF, 0.548 mmol, 2.2 eq). The mixture was stirred for 18 h and the extraction was conducted with diethyl ether. The residue was solubilised in DCM, treated with isohexanes and the formed suspension was cooled in the refrigerator at 5 °C for 2 h. The formed precipitate collected by filtration and washed with isohexanes to give product **A.17** (34 mg, 0.13 mmol, 54%) as a dark yellow solid. Mp: 117 – 118 °C. ^{1}H NMR (400 MHz, DMSO-d_6) δ 8.77 (t, J = 6.2 Hz, 1H), 8.06 (ddd, J = 7.9, 1.3, 0.6 Hz, 1H), 7.99 – 7.89 (m, 1H), 7.49 (ddd, J = 8.2, 7.2, 1.3 Hz, 1H), 7.41 (ddd, J = 8.3, 7.2, 1.3 Hz, 1H), 4.68 (d, J = 6.2 Hz, 2H), 2.98 (s, 2H), 2.27 (s, 6H). ^{13}C NMR (101 MHz, DMSO-d_6) δ 171.5, 170.5, 152.7, 134.5, 126.1, 122.3, 62.7, 45.6, 41.0. HRMS (EI): m/z = calculated for $C_{12}H_{15}N_3OS$ $[M]^{\cdot+}$: 249.0931; found: 249.0930. Purity (HPLC): 100% (λ = 210 nm), 100% (λ = 254 nm), Method 1a.

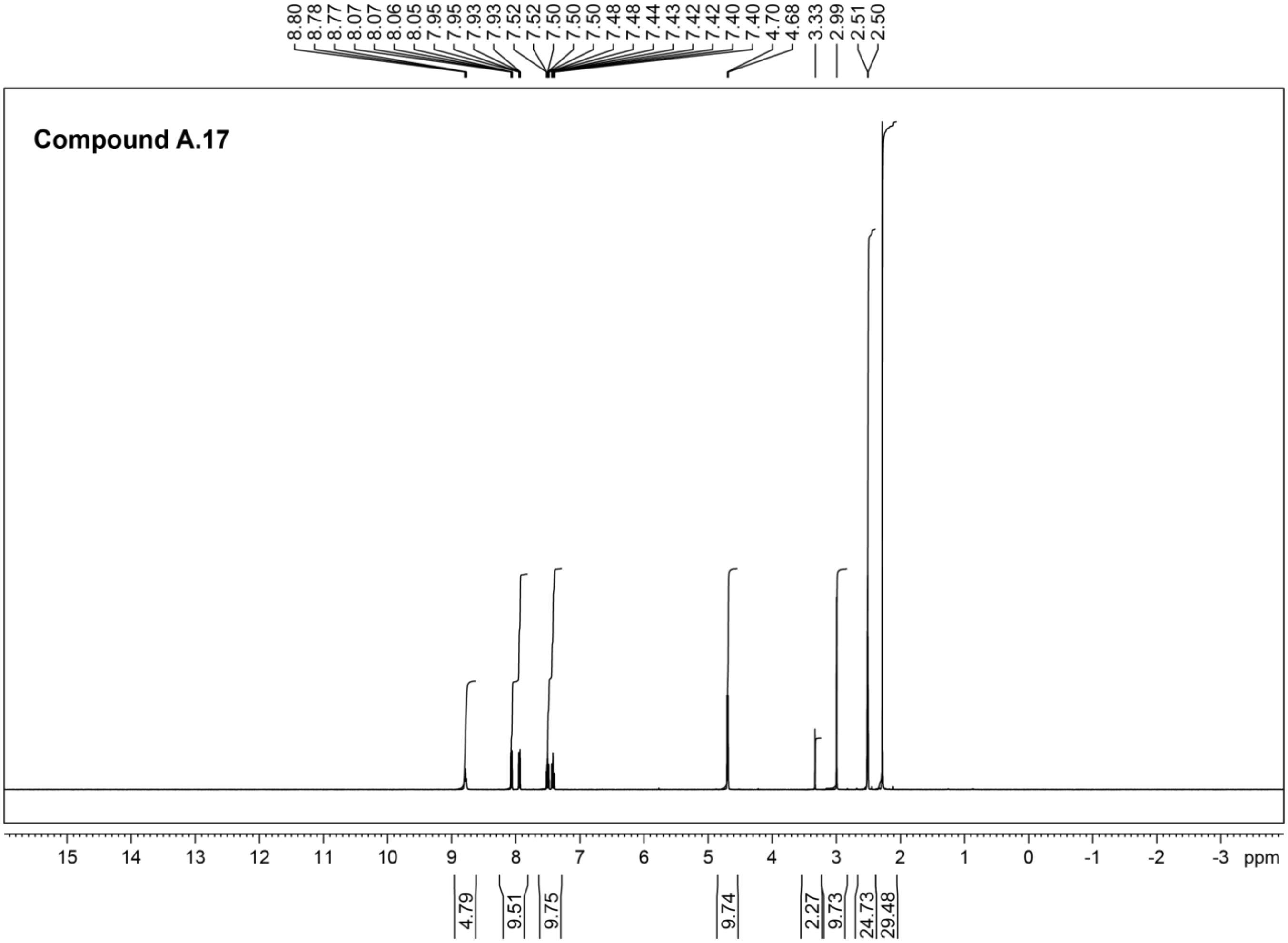

Synthesis Suppl. Figure 22. ^{1}H NMR of compound A.17.

***N*-(6-Methoxybenzo[*d*]thiazol-2-yl)-2-(4-methylpiperazin-1-yl)acetamide (A.18)**

Amine **A.18** was prepared following General procedure F, using chloroacetamide **2** (100 mg, 0.390 mmol, 1.0 eq) and 1-methylpiperazine (95 µL, 0.86 mmol, 2.2 eq). The mixture was stirred for 18 h and the extraction was conducted with EtOAc. The residue was recrystallised from DCM to give **A.18** (98 mg, 0.31 mmol, 79%) as a dark yellow solid. Mp: 104 – 105 °C. ^{1}H NMR (400 MHz, $CDCl_3$) δ 10.31 (s, 1H), 7.68 (d, *J* = 9.0 Hz, 1H), 7.29 (d, *J* = 2.5 Hz, 1H), 7.07 – 7.01 (m, 1H), 3.87 (s, 3H), 3.29 (s, 2H), 2.71 (d, *J* = 5.0 Hz, 4H), 2.37 (s, 3H). ^{13}C NMR (101 MHz, $CDCl_3$) δ 168.9, 157.1, 155.2, 142.8, 133.6, 121.7, 115.5, 104.4, 61.1, 56.0, 54.9, 53.4, 45.9. HRMS (EI): *m/z* = calculated for $C_{15}H_{20}N_4O_2S$ $[M]^{\cdot+}$: 420.1302; found: 420.1299. Purity (HPLC): > 97% (λ = 210 nm), > 98% (λ = 254 nm), Method 1a.

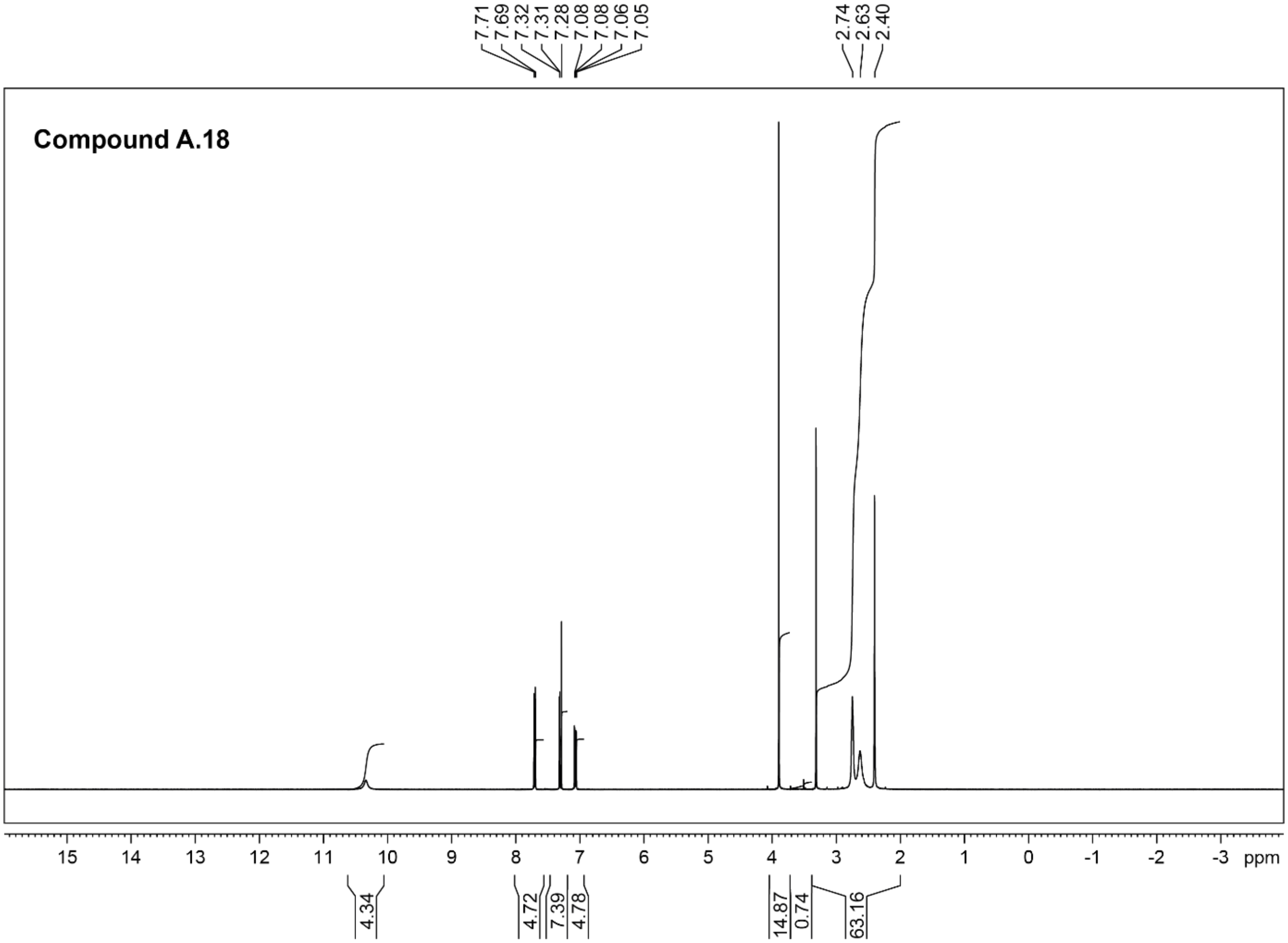

Synthesis Suppl. Figure 23. ^{1}H NMR of compound A.18.

2-(Dimethylamino)-*N*-(6-(methylthio)benzo[*d*]thiazol-2-yl)acetamide (A.19)

2-(Dimethylamino)acetamide **A.19** was prepared following General procedure F, using chloroacetamide **12** (106 mg, 0.389 mmol, 1.0 eq) and dimethylamine (0.427 mL, 2 M in THF, 0.855 mmol, 2.2 eq). The mixture was stirred for 18 h and the extraction was conducted with diethyl ether. The residue was purified by flash column chromatography (99:1 DCM/MeOH), yielding product **A.19** (75 mg, 0.27 mmol, 69%) as a brown solid. Mp: 109 – 110 °C. ^{1}H NMR (400 MHz, DMSO-d_6) δ 7.91 (d, *J* = 1.9 Hz, 1H), 7.65 (d, *J* = 8.5 Hz, 1H), 7.34 (dd, *J* = 8.5, 2.0 Hz, 1H), 2.53 (s, 3H), 2.29 (s, 6H). ^{13}C NMR (101 MHz, DMSO-d_6) δ 169.7, 157.1, 146.4, 133.0, 132.7, 125.4, 120.7, 119.1, 61.3, 45.1, 15.8. HRMS (ESI): *m/z* = calculated for $C_{12}H_{16}N_3O_2S_2$ [M+H]$^+$: 282.0730; found: 282.0730. Purity (HPLC): > 96% (λ = 210 nm), 100% (λ = 254 nm), Method 1a.

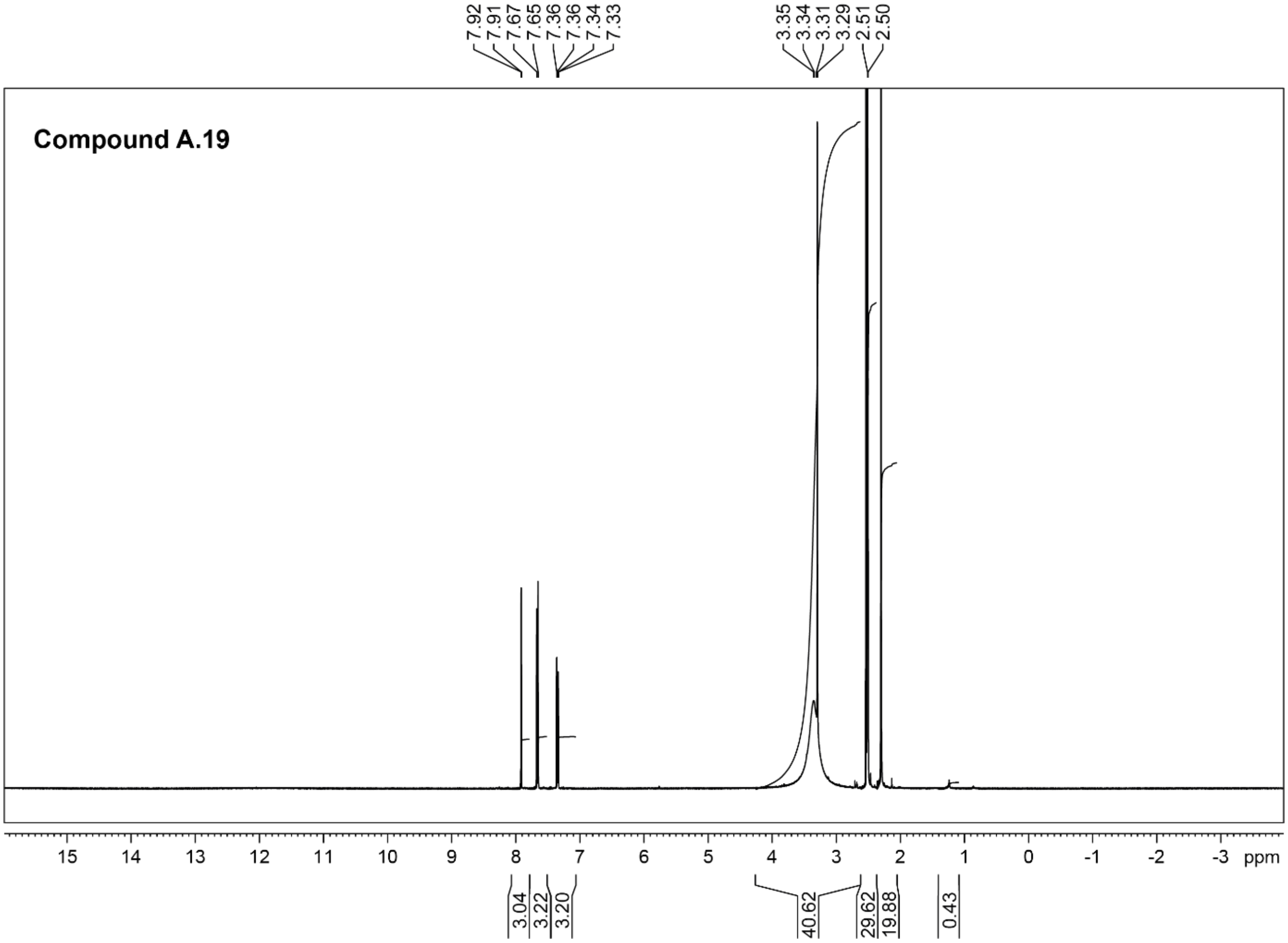

Synthesis Suppl. Figure 24. ^{1}H NMR of compound A.19.

2-(Dimethylamino)-*N*-(6-methoxy-1*H*-benzo[*d*]imidazol-2-yl)acetamide (A.20)

2-(Dimethylamino)acetamide **A.20** was prepared following General procedure F, using chloroacetamide **8** (21 mg, 0.088 mmol, 1.0 eq) and dimethylamine (96 µL, 2 M in THF, 0.19 mmol, 2.2 eq). The mixture was stirred for 18 h and the extraction was conducted with EtOAc. The crude product was purified by flash column chromatography (9:1 DCM/MeOH), yielding product **A.20** (13 mg, 0.051 mmol, 58%) as a light brown solid. Mp: 178 – 180 °C. ^{1}H NMR (400 MHz, DMSO-*d*$_6$) δ 11.48 (s br, 1H), 7.31 (d, *J* = 8.6 Hz, 1H), 6.99 (s, 1H), 6.71 (dd, *J* = 8.6, 2.4 Hz, 1H), 3.74 (s, 3H), 3.22 (s, 2H), 2.31 (s, 6H). ^{13}C NMR (101 MHz, DMSO-*d*$_6$) δ 169.6, 155.0, 109.7, 61.6, 55.4, 45.1. HRMS (EI): *m/z* = calculated for $C_{12}H_{16}N_4O_2$ $[M]^{\cdot+}$: 248.1268; found: 248.1268. Purity (HPLC): > 98% (λ = 210 nm), 100% (λ = 254 nm), Method 1a.

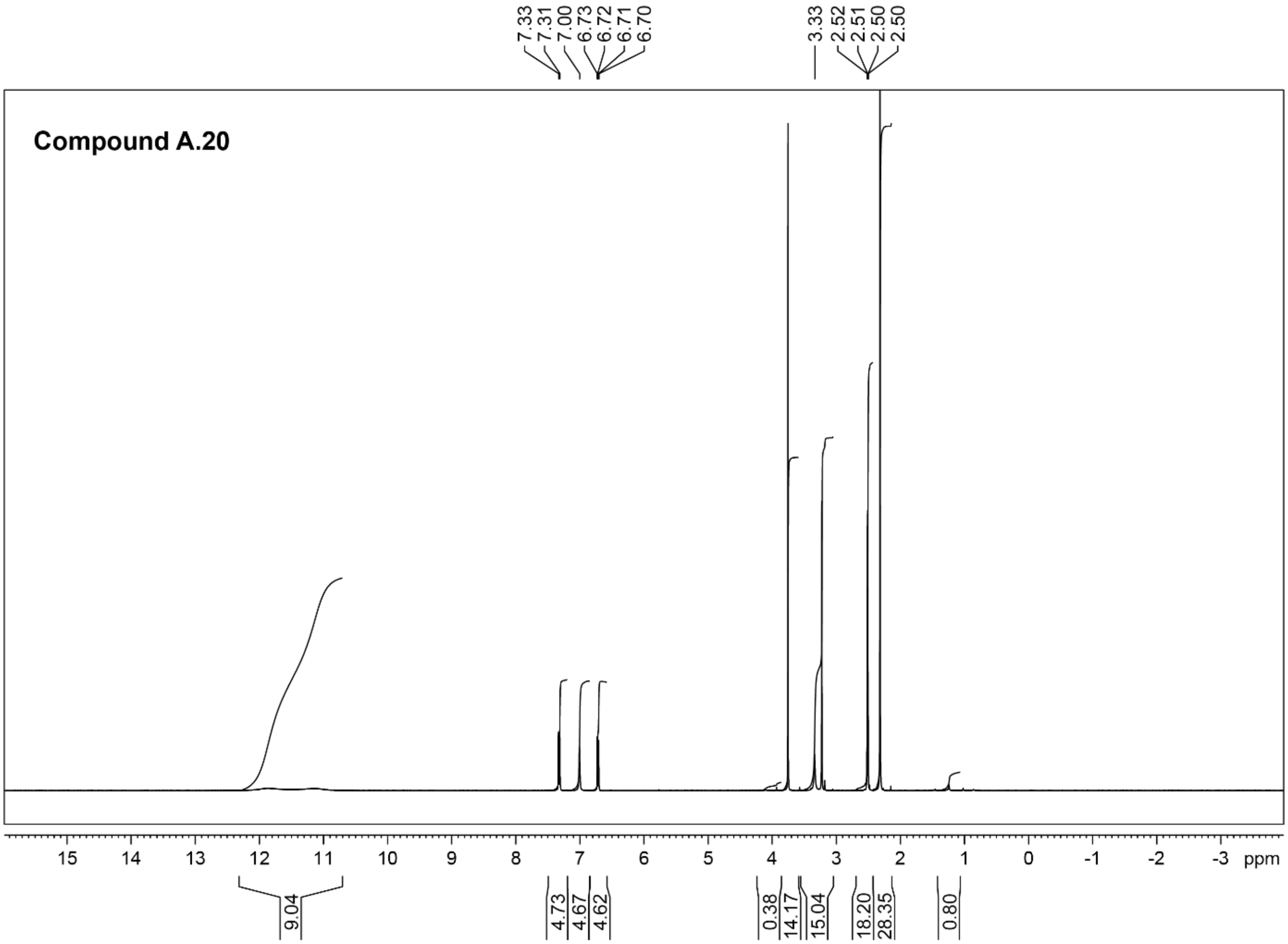

Synthesis Suppl. Figure 25. ^{1}H NMR of compound A.20.

2-(Dimethylamino)-*N*-(5-(4-methoxyphenyl)-1,3,4-thiadiazol-2-yl)acetamide (A.21)

2-(Dimethylamino)acetamide **A.21** was prepared following General procedure E and F, without isolating the chloroacetamide intermediate. 5-(4-Methoxyphenyl)-1,3,4-thiadiazol-2-amine (116 mg, 0.409 mmol, 1.0 eq) was suspended in THF and 2-chloroacetyl chloride (36 µL, 0.45 mmol, 1.1 eq) and triethylamine (63 µL, 0.45 mmol, 1.1 eq) were added to the solution. After stirring at room temperature for 24 h, dimethylamine (0.41 mL, 2 M in THF, 0.82 mmol, 2.0 eq) was added and the mixture was stirred for additional 24 h. The extraction was conducted with EtOAc and the residue was purified by flash column chromatography (99:1 DCM/MeOH), yielding product **A.21** (87 mg, 0.30 mmol, 73%) as a colourless solid. Mp: 185 – 187 °C. ^{1}H NMR (400 MHz, DMSO-d_6) δ 12.20 (s, 1H), 7.92 – 7.83 (m, 2H), 7.11 – 7.03 (m, 2H), 3.83 (s, 3H), 3.33 (s, 2H), 2.31 (s, 6H). ^{13}C NMR (101 MHz, DMSO-d_6) δ 168.9, 161.5, 161.0, 157.8, 128.4, 122.8, 114.7, 61.1, 55.4, 44.9. HRMS (EI): *m/z* = calculated for $C_{13}H_{16}N_4O_2S$ $[M]^{\cdot+}$: 292.0989; found: 292.0990. Purity (HPLC): > 95% (λ = 210 nm), > 99% (λ = 254 nm), Method 1a.

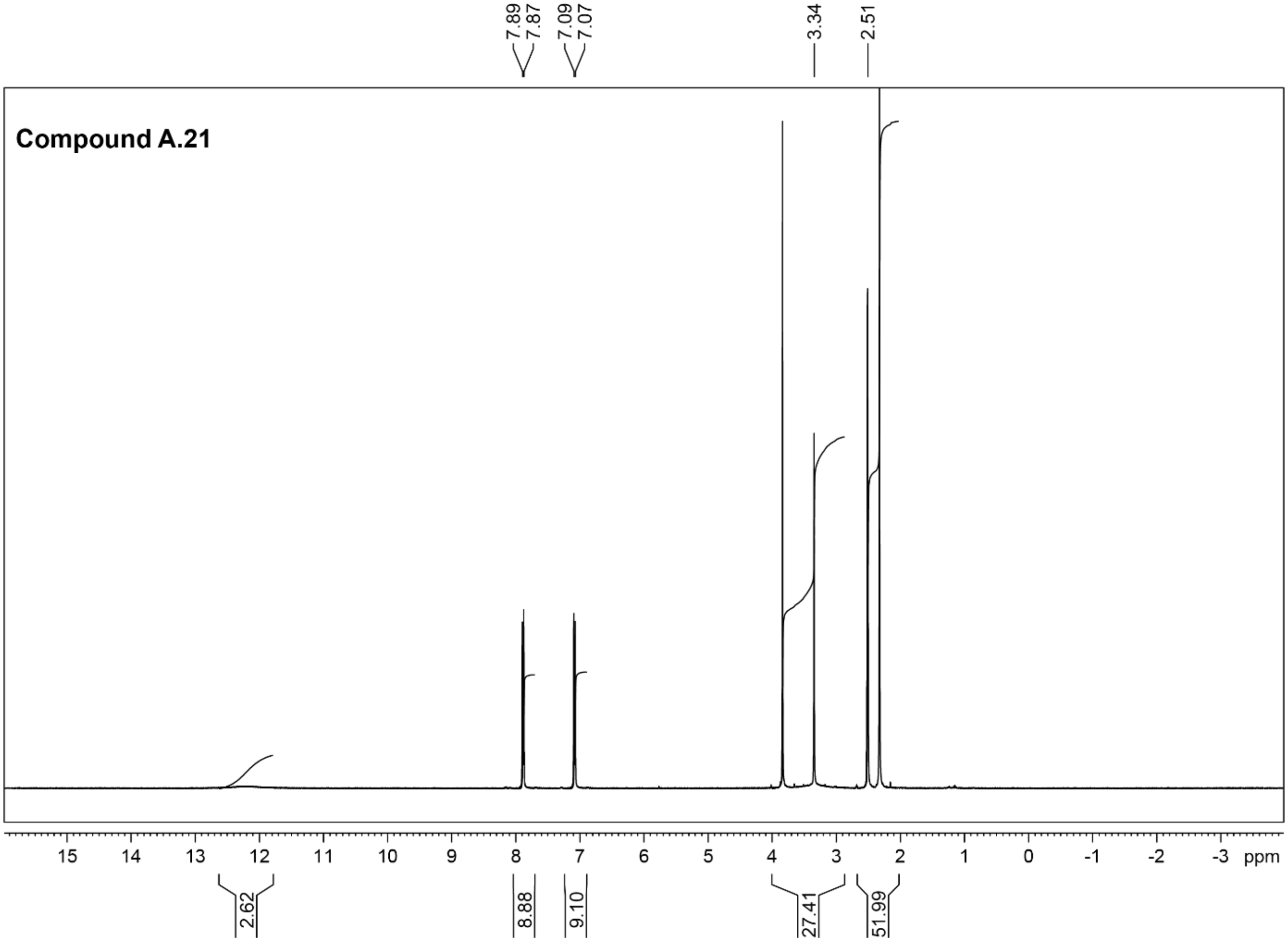

Synthesis Suppl. Figure 26. ^{1}H NMR of compound A.21.

N-(6-Aminobenzo[d]thiazol-2-yl)-2-(dimethylamino)acetamide (A.24)

6-Aminobenzothiazole **A.24** was prepared following General procedure G from 6-nitrobenzothiazole **A.14** (100 mg, 0.357 mmol, 1.0 eq). The mixture was stirred for 3 h. The crude product was purified by flash column chromatography (98:2 DCM/MeOH), yielding product **A.24** (55 mg, 0.22 mmol, 62%) as an old pink sold. Mp: 151 – 152 °C. ^{1}H NMR (400 MHz, DMSO-d_6) δ 11.66 (s, 1H), 7.40 (d, J = 8.6 Hz, 1H), 7.00 (d, J = 2.2 Hz, 1H), 6.70 (dd, J = 8.6, 2.2 Hz, 1H), 5.16 (s, 2H), 3.22 (s, 2H), 2.28 (s, 6H). ^{13}C NMR (101 MHz, DMSO-d_6) δ 168.9, 152.5, 145.8, 139.5, 133.0, 120.9, 114.4, 104.1, 61.3, 45.1. HRMS (ESI): m/z = calculated for $C_{11}H_{15}N_4OS$ $[M+H]^+$: 251.0962; found: 251.0961. Purity (HPLC): > 99% (λ = 210 nm), > 98% (λ = 254 nm), Method 1a.

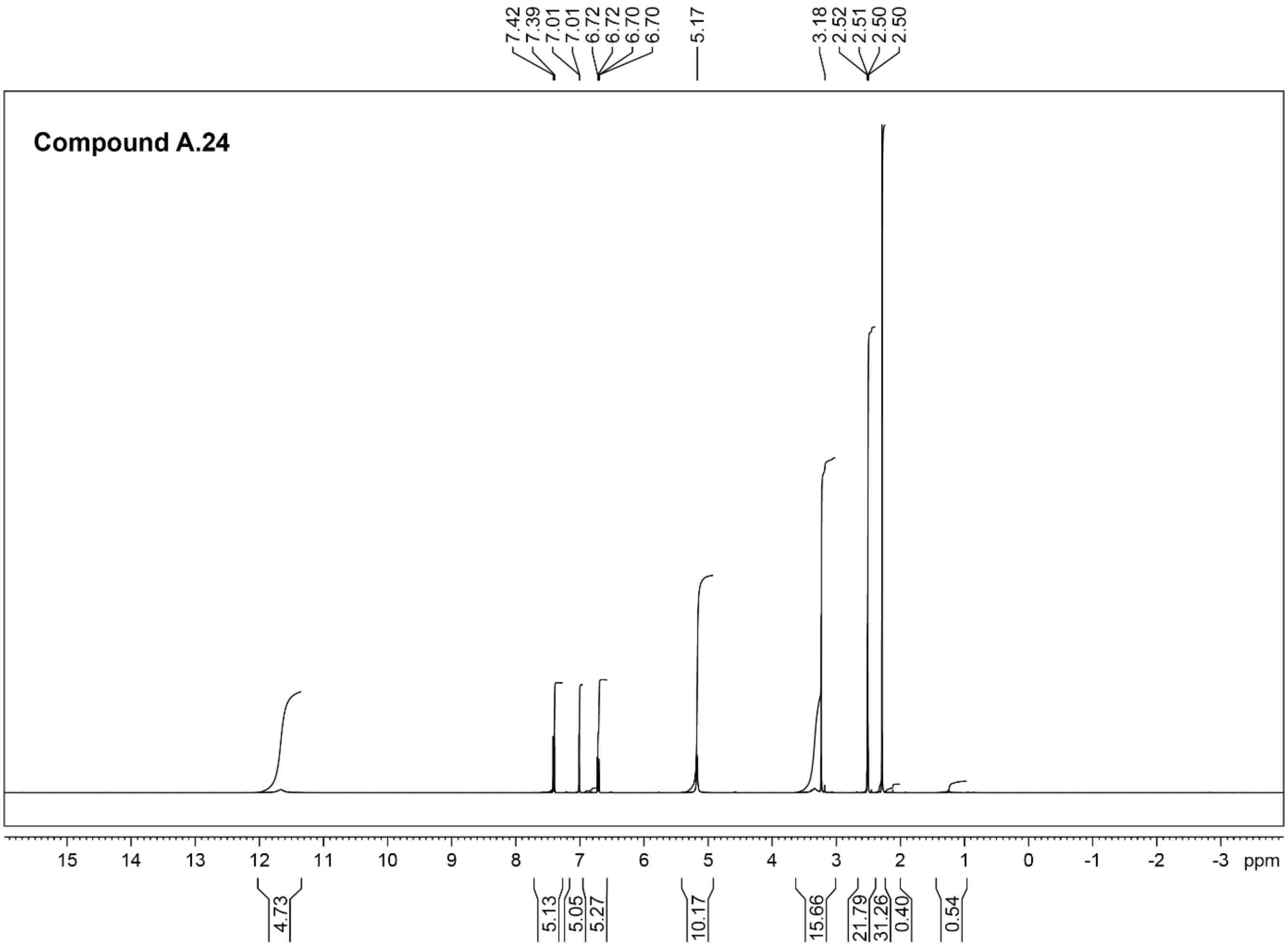

Synthesis Suppl. Figure 27. ^{1}H NMR of compound A.24.

2-(Benzyl(methyl)amino)-*N*-(4,6-dimethoxybenzo[*d*]thiazol-2-yl)acetamide (A.25)

Amine **A.25** was prepared following General procedure F, using chloroacetamide **6** (400 mg, 1.39 mmol, 1.0 eq) and *N*-benzylmethylamine (0.275 mL, 2.09 mmol, 2.2 eq). The mixture was stirred for 24 h and the extraction was conducted with EtOAc. The residue was purified by flash column chromatography (99:1 DCM/MeOH), yielding product **A.25** (481 mg, 1.29 mmol, 93%) as a colourless solid. Mp: 162 – 164 °C. ^{1}H NMR (400 MHz, DMSO-d_6) δ 10.76 (s, 1H), 7.62 – 7.54 (m, 2H), 7.47 (dd, *J* = 4.8, 1.8 Hz, 3H), 7.16 (d, *J* = 2.2 Hz, 1H), 6.62 (d, *J* = 2.3 Hz, 1H), 4.49 – 4.41 (m, 2H), 4.30 – 4.24 (m, 2H), 3.89 (s, 3H), 3.81 (s, 3H), 2.86 (s, 3H). ^{13}C NMR (101 MHz, DMSO-d_6) δ 157.6, 152.3, 131.5, 129.8, 129.6, 128.9, 98.2, 95.8, 66.4, 51.9, 55.9, 55.7, 55.0, 40.7. HRMS (ESI): *m/z* = calculated for $C_{19}H_{22}N_3O_3S$ [M+H]$^+$: 371.1304; found: 371.1301. Purity (HPLC): 100% (λ = 210 nm), 100% (λ = 254 nm), Method 3d.

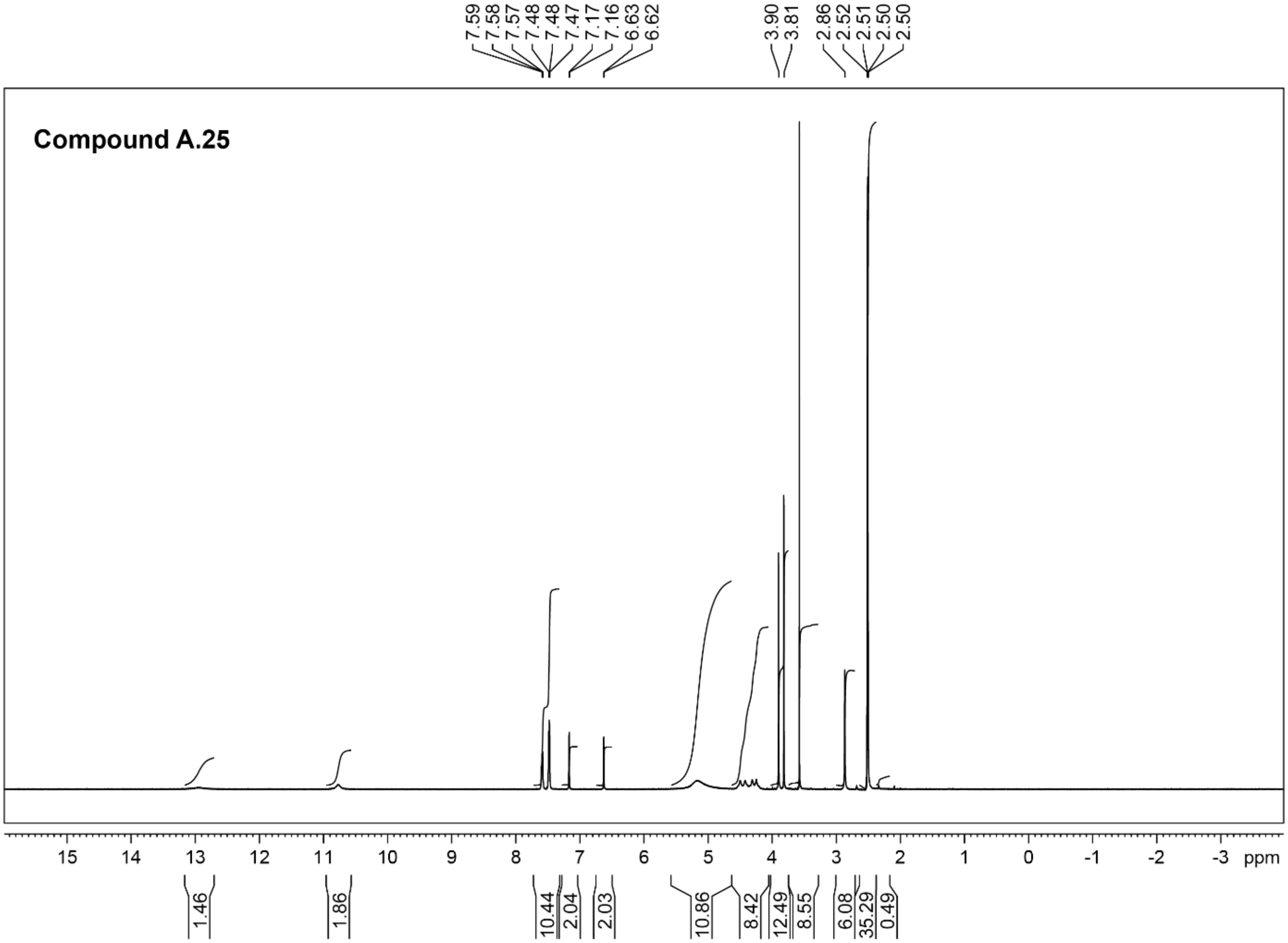

Synthesis Suppl. Figure 28. ^{1}H NMR of compound A.25.

2-(Dimethylamino)-*N*-(6-(trifluoromethyl)benzo[*d*]thiazol-2-yl)acetamide (A.27)

2-(Dimethylamino)acetamide **A.27** was prepared following General procedure F, using chloroacetamide **11** (102 mg, 0.346 mmol, 1.0 eq) and dimethylamine (0.381 mL, 2 M in THF, 0.761 mmol, 2.2 eq). The mixture was stirred for 18 h and the extraction was conducted with diethyl ether. The residue was purified by flash column chromatography (99:1 DCM/MeOH), yielding product **A.27** (28 mg, 0.093 mmol, 27%) as a colourless solid. Mp: 238 – 239 °C. ^{1}H NMR (400 MHz, DMSO-*d*$_6$) δ 12.25 (s, 1H), 8.48 (dt, *J* = 2.0, 0.8 Hz, 1H), 7.89 (dt, *J* = 8.4, 0.8 Hz, 1H), 7.73 (dd, *J* = 8.8, 1.9 Hz, 1H), 3.37 (s, 2H), 2.33 (s, 6H). ^{13}C NMR (101 MHz, DMSO-*d*$_6$) δ 170.1, 161.1, 151.3, 132.1, 126.0, 123.8, 123.5, 123.3, 122.9, 120.9, 119.8, 61.2, 45.0. HRMS (EI): *m/z* = calculated for $C_{12}H_{12}N_3O_2F_3$ $[M]^{\cdot+}$: 303.0648; found: 303.0646. Purity (HPLC): > 91% (λ = 210 nm), > 87% (λ = 254 nm), Method 1a.

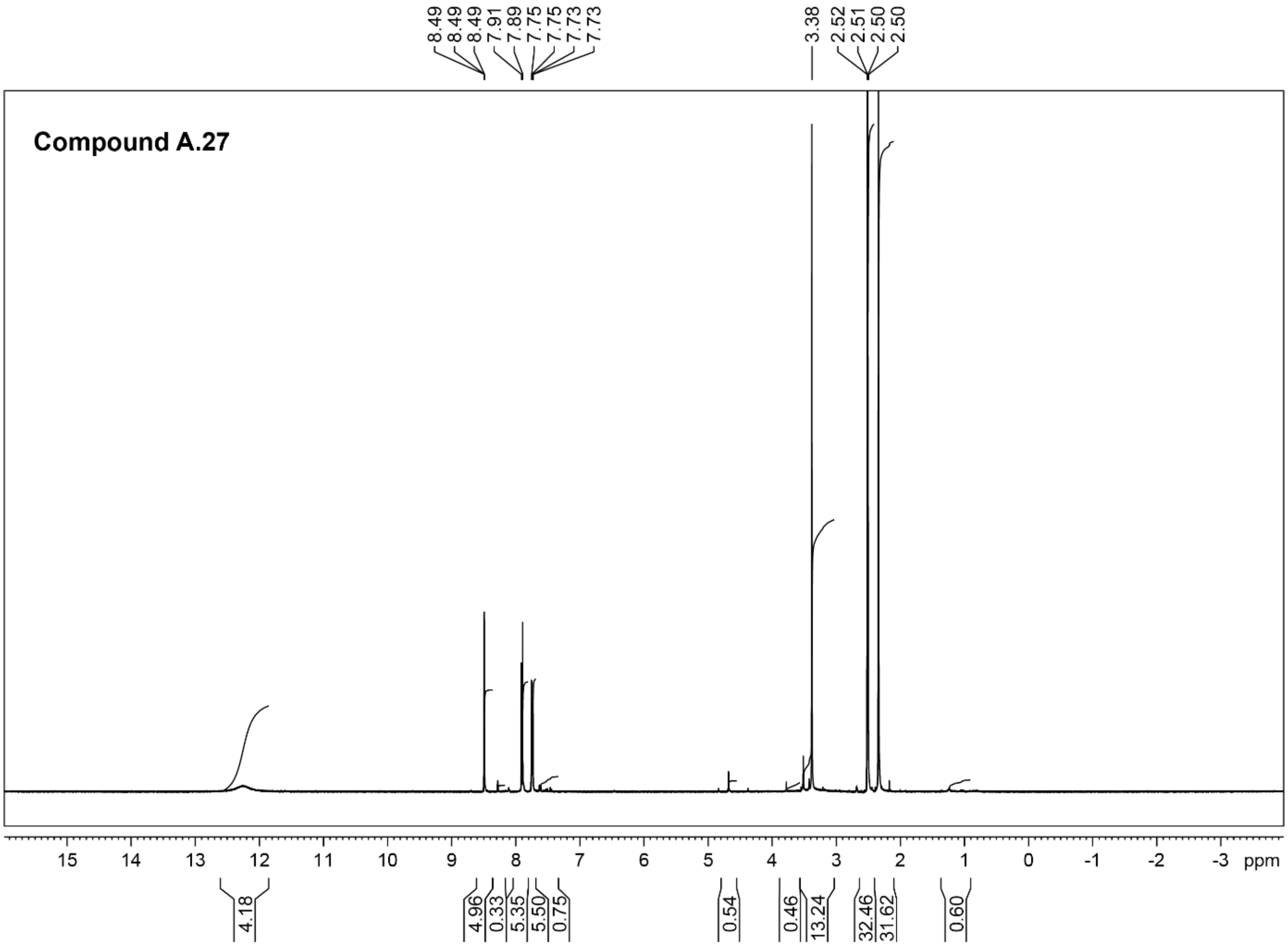

Synthesis Suppl. Figure 29. ^{1}H NMR of compound A.27.

2-(Dimethylamino)-*N*-(6-(trifluoromethoxy)benzo[*d*]thiazol-2-yl)acetamide (A.28)

2-(Dimethylamino)acetamide **A.28** was prepared following General procedure F, using chloroacetamide **13** (86 mg, 0.28 mmol, 1.0 eq) and dimethylamine (0.306 mL, 2 M in THF, 0.612 mmol, 2.2 eq). The mixture was stirred for 18 h and the extraction was conducted with diethyl ether. The residue was purified by flash column chromatography (95:5 DCM/MeOH), yielding product **A.28** (28 mg, 0.15 mmol, 53%) as an off-white solid. Mp: 120 – 122 °C. ^{1}H NMR (400 MHz, DMSO-d_6) δ 8.11 (dd, J = 2.6, 1.1 Hz, 1H), 7.81 (d, J = 8.8 Hz, 1H), 7.41 (ddd, J = 9.5, 2.9, 1.3 Hz, 1H), 3.31 (s, 2H), 2.30 (s, 6H). ^{13}C NMR (101 MHz, DMSO-d_6) δ 170.0, 159.3, 147.6, 144.0, 144.0, 132.7, 121.5, 119.8, 115.0, 61.3, 45.0. HRMS (EI): m/z = calculated for $C_{12}H_{12}N_3O_2F_3$ $[M]^{\cdot+}$: 319.0597; found: 319.0597. Purity (HPLC): > 97% (λ = 210 nm), > 93% (λ = 254 nm), Method 1a.

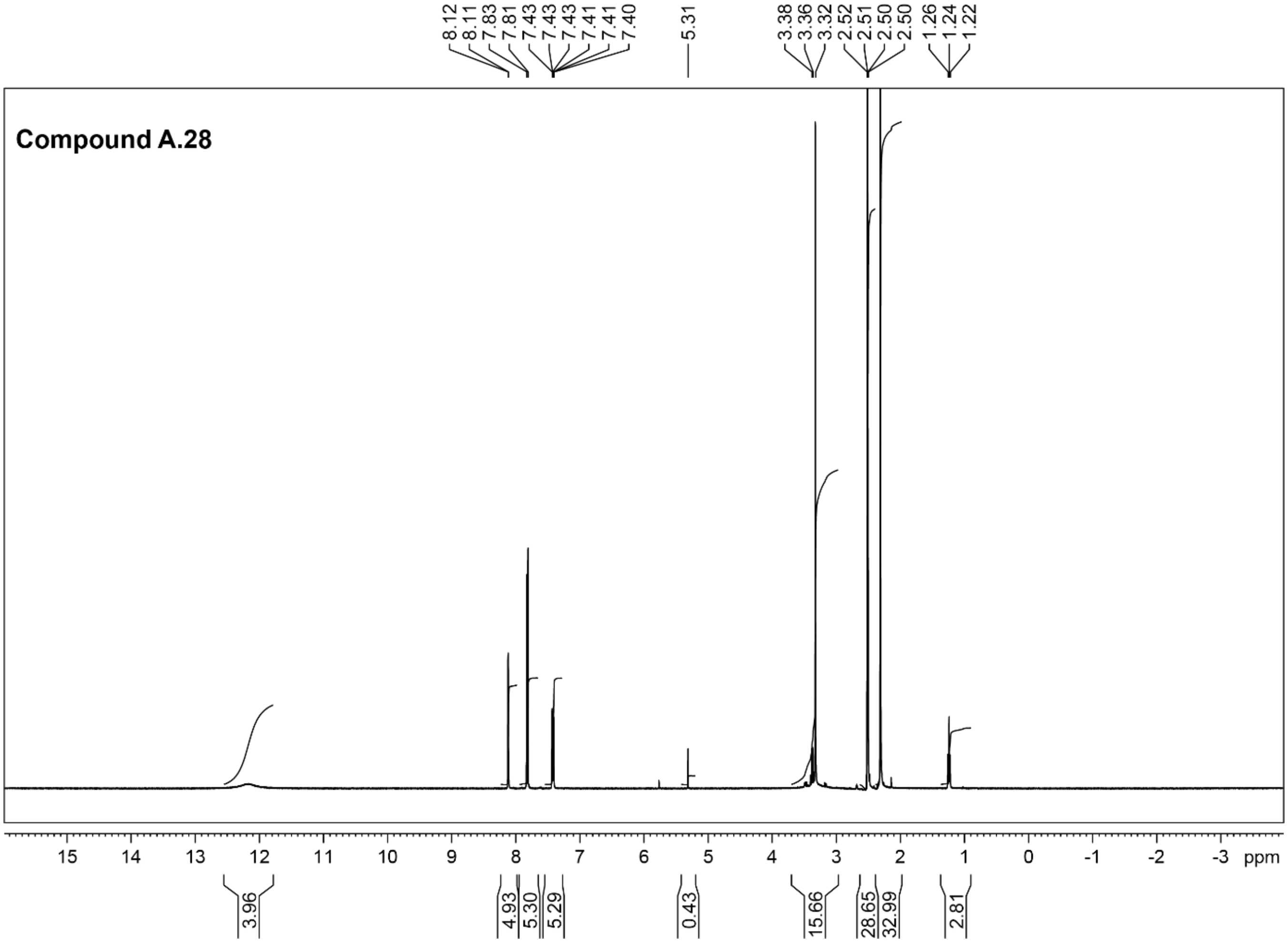

Synthesis Suppl. Figure 30. ^{1}H NMR of compound A.28.

***N*-(6-Methoxybenzo[*d*]thiazol-2-yl)-2-((2-methoxyethyl)(methyl)amino)acetamide (A.30)**

Amine **A.30** was prepared following General procedure F, using chloroacetamide **2** (100 mg, 0.390 mmol, 1.0 eq) and (2-methoxyethyl)methylamine (92 µL, 0.86 mmol, 2.2 eq). The mixture was stirred for 18 h and the extraction was conducted with EtOAc. The residue was purified by flash column chromatography (99:1 DCM/MeOH), yielding product **A.30** (109 mg, 0.354 mmol, 91%) as a beige waxy solid. ^{1}H NMR (400 MHz, DMSO-d_6) δ 11.71 (s, 1H), 7.63 (d, *J* = 8.8 Hz, 1H), 7.57 (d, *J* = 2.6 Hz, 1H), 7.03 (dd, *J* = 8.8, 2.6 Hz, 1H), 3.80 (s, 3H), 3.45 (t, *J* = 5.5 Hz, 2H), 3.39 (s, 2H), 2.69 (t, *J* = 5.5 Hz, 2H), 2.37 (s, 3H). ^{13}C NMR (101 MHz,DMSO-d_6) δ 170.1, 156.2, 155.3, 142.6, 132.8, 121.2, 114.9, 104.8, 70.0, 59.8, 58.0, 56.0, 55.6, 42.8. HRMS (ESI): *m/z* = calculated for $C_{20}H_{31}N_4O_2S$ [M-H]$^-$: 308.1074; found: 308.1075. Purity (HPLC): 100% (λ = 210 nm), 100% (λ = 254 nm), Method 1a.

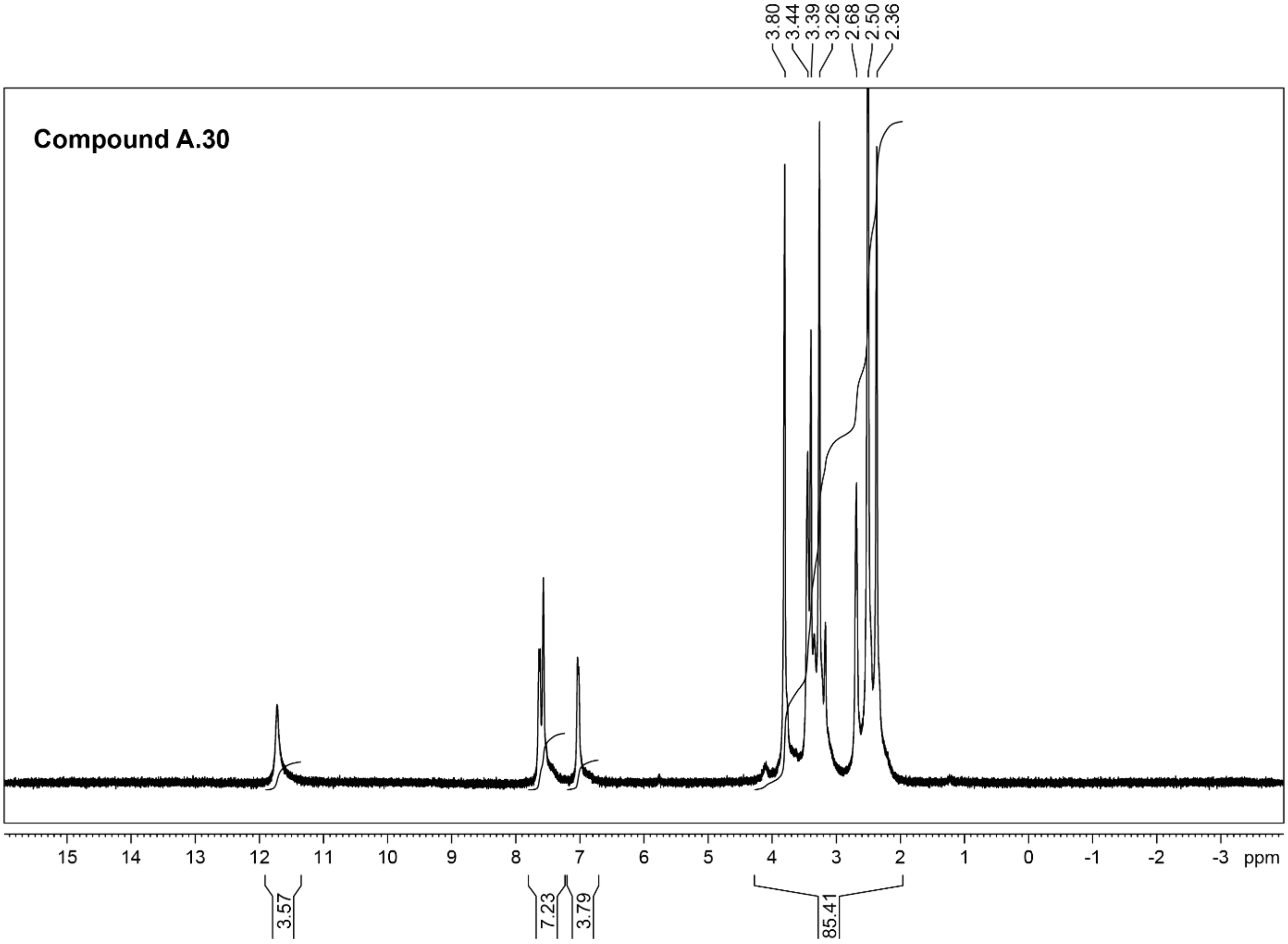

Synthesis Suppl. Figure 31. ^{1}H NMR of compound A.30.

***N*-(5-(3,4-Dimethoxyphenyl)-1,3,4-thiadiazol-2-yl)-2-(dimethylamino)acetamide (A.31)**

2-(Dimethylamino)acetamide **A.31** was prepared following General procedure F, using chloroacetamide **15** (36 mg, 0.12 mmol, 1.0 eq) and dimethylamine (0.126 mL, 2 M in THF, 0.252 mmol, 2.2 eq). The mixture was stirred for 18 h and the extraction was conducted with DCM. The residue was purified by flash column chromatography (DCM → 95:5 DCM/MeOH), yielding product **A.31** (27 mg, 0.084 mmol, 73%) as an off-white solid. Mp: 188 – 190 °C (decomposition and melting). ^{1}H NMR (500 MHz, DMSO-d_6) δ 12.18 (s, 2H), 7.50 (d, J = 2.1 Hz, 1H), 7.45 (dd, J = 8.3, 2.1 Hz, 1H), 7.08 (d, J = 8.3 Hz, 1H), 3.85 (s, 3H), 3.82 (s, 3H), 3.34 (s, 3H), 2.32 (s, 6H). ^{13}C NMR (126 MHz, DMSO-d_6) δ 168.9, 161.7, 157.9, 150.8, 149.1, 122.9, 120.3, 115.8, 112.0, 109.3, 61.0, 55.7, 55.6, 44.9. HRMS (ESI): m/z = calculated for $C_{14}H_{19}N_4O_3S$ $[M+H]^+$: 323.1173; found: 323.1177. Purity (HPLC): > 95% (λ = 210 nm), > 96% (λ = 254 nm), Method 1a.

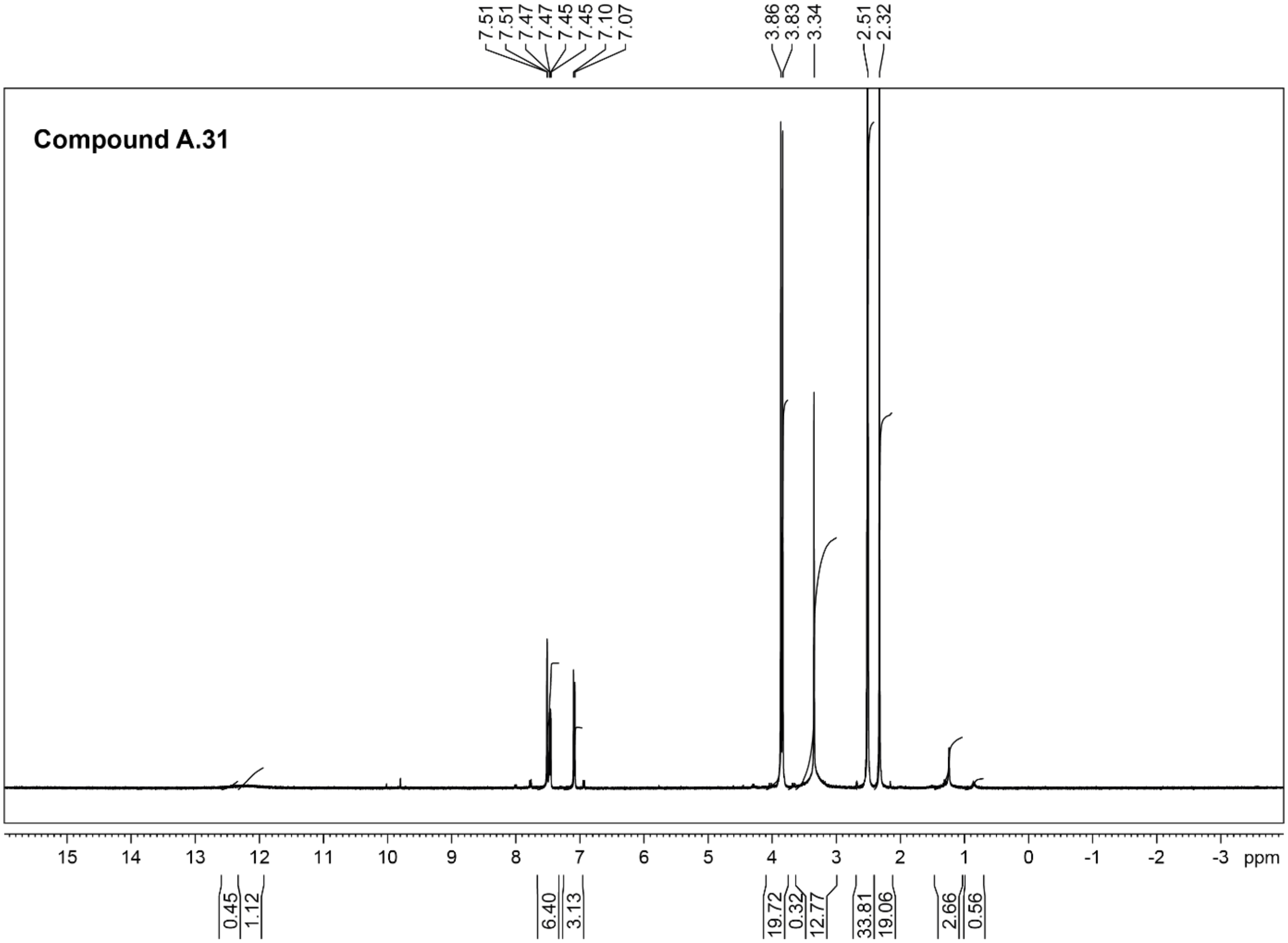

Synthesis Suppl. Figure 32. ^{1}H NMR of compound A.31.

2-(Dimethylamino)-*N*-(6-(methylsulfonyl)benzo[*d*]thiazol-2-yl)acetamide (A.32)

2-(Dimethylamino)acetamide **A.32** was prepared following General procedure F, using alkyl chloride **16** (67 mg, 0.22 mmol, 1.0 eq) and dimethylamine (0.242 mL, 2 M in THF, 0.484 mmol, 2.2 eq). The mixture was stirred for 2 h and the extraction was conducted with EtOAc. The crude product was purified by flash column chromatography (97:3 DCM/MeOH), yielding product **A.32** (44 mg, 0.14 mmol, 64%) as a pale yellow solid. Mp: 232 – 233 °C. ^{1}H NMR (400 MHz, DMSO-d_6) δ 8.61 (dd, J = 1.7, 0.8 Hz, 1H), 7.95 – 7.88 (m, 2H), 3.37 (s, 3H), 3.24 (s, 3H), 2.33 (s, 7H). ^{13}C NMR (101 MHz, DMSO-d_6) δ 170.3, 162.2, 152.2, 135.2, 132.1, 124.8, 122.0, 120.7, 61.2, 44.9, 44.1. HRMS (EI): m/z = calculated for $C_{12}H_{15}N_3O_3S_2$ $[M]^{\cdot+}$: 313.0550; found: 313.0550. Purity (HPLC): > 99% (λ = 210 nm), > 98% (λ = 254 nm), Method 3b.

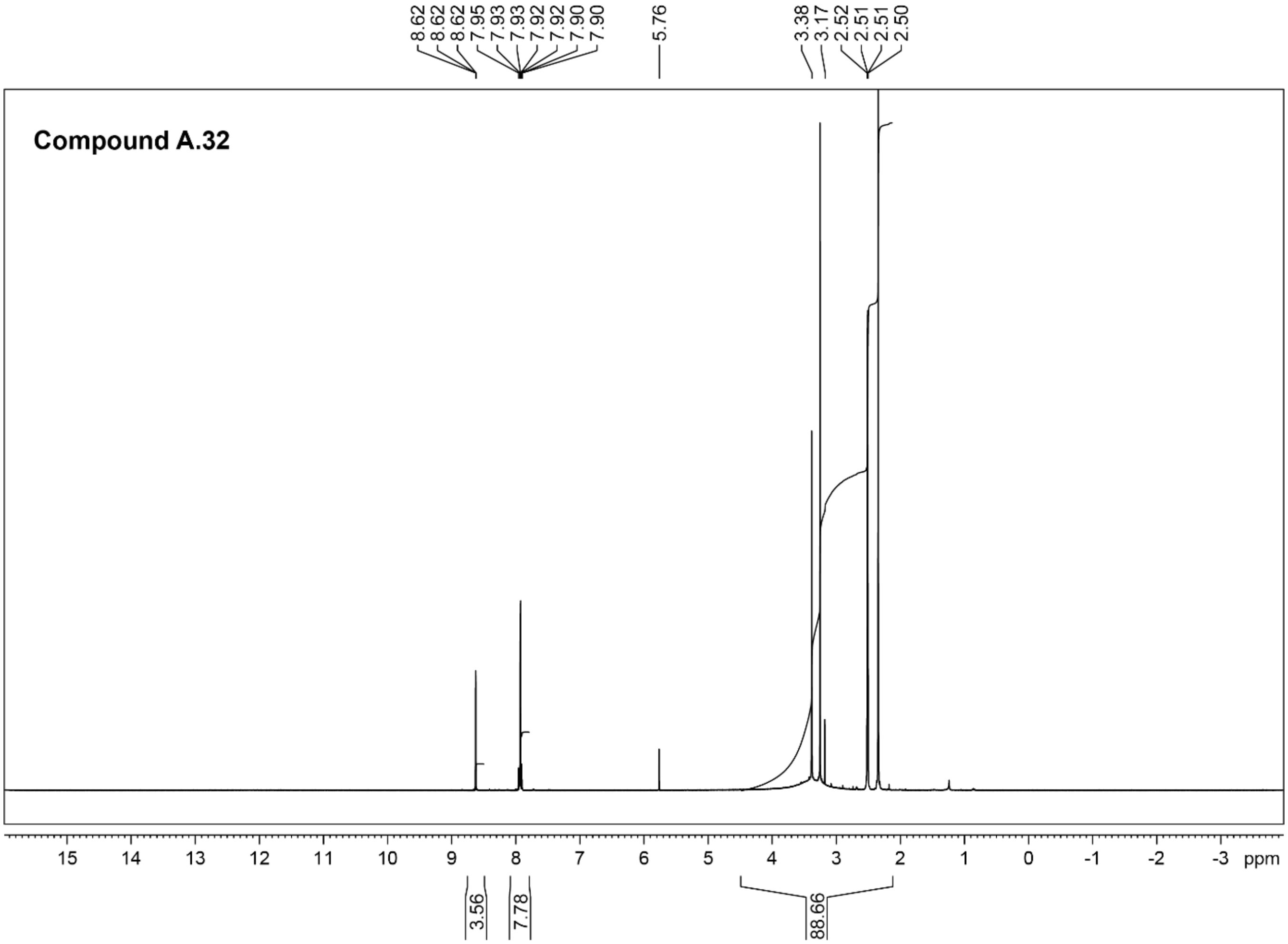

Synthesis Suppl. Figure 33. ^{1}H NMR of compound A.32.

N-(6-Methoxybenzo[d]thiazol-2-yl)-2-(methyl(pyrimidin-5-ylmethyl)amino)acetamide (A.33)

Amine **A.33** was prepared following General procedure F, using chloroacetamide **2** (282 mg, 1.10 mmol, 1.0 eq), amine **17** (149 mg, 1.21 mmol, 1.1 eq) and triethylamine (0.169 mL, 1.21 mmol, 1.1 eq). The mixture was stirred for 1 h and the extraction was conducted with EtOAc. The residue was purified by flash column chromatography (97:3 DCM/MeOH), yielding product **A.33** (134 mg, 0.390 mmol, 36%) as an off-white solid. Mp: 155 – 156 °C. ^{1}H NMR (400 MHz, DMSO-d_6) δ 12.06 (s, 1H), 9.10 (s, 1H), 8.81 (s, 2H), 7.64 (d, J = 8.8 Hz, 1H), 7.57 (d, J = 2.6 Hz, 1H), 7.03 (dd, J = 8.8, 2.6 Hz, 1H), 3.81 (s, 3H), 3.75 (s, 2H), 3.47 (s, 2H), 2.29 (s, 3H). ^{13}C NMR (101 MHz, DMSO-d_6) δ 169.6, 157.4, 157.3, 156.1, 155.5, 142.6, 132.8, 131.9, 121.1, 114.9, 104.7, 59.1, 55.6, 55.4, 41.7. HRMS (ESI): m/z = calculated for $C_{16}H_{16}N_5O_2S$ [M-H]$^-$: 342.1030; found: 342.1030. Purity (HPLC): 100% (λ = 210 nm), 100% (λ = 254 nm), Method 1a.

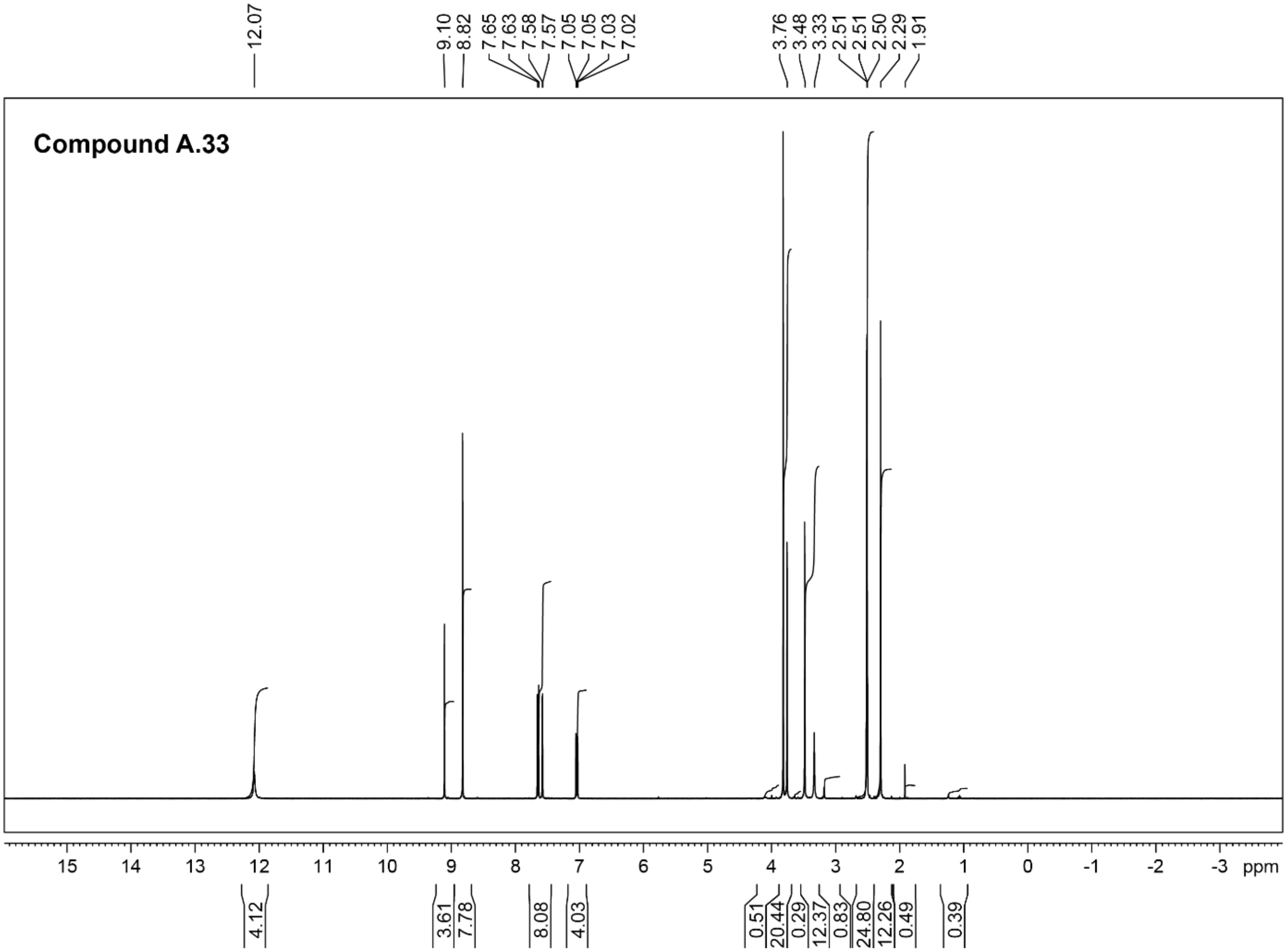

Synthesis Suppl. Figure 34. ^{1}H NMR of compound A.33.

2-(Dimethylamino)-*N*-(6-methoxybenzo[*d*]thiazol-2-yl)-*N*-methylacetamide (A.34)

2-(Dimethylamino)acetamide **A.34** was prepared following General procedure F, using alkyl chloride **23** (81 mg, 0.30 mmol, 1.0 eq) and dimethylamine (0.33 mL, 2 M in THF, 0.66 mmol, 2.2 eq). The mixture was stirred for 2 h and the extraction was conducted with EtOAc (3 x 5 mL). The crude product was purified by flash column chromatography (99:1 DCM/MeOH), yielding product **A.34** (65 mg, 0.23 mmol, 78%) as a beige solid. Mp: 120 – 121 °C. ^{1}H NMR (400 MHz, DMSO-d_6): δ 7.70 (d, J = 8.8 Hz, 1H), 7.54 (d, J = 2.5 Hz, 1H), 7.03 (dd, J = 8.8, 2.6 Hz, 1H), 3.81 (s, 3H), 3.72 (s, 3H), 3.52 (s, 2H), 2.30 (s, 6H). ^{13}C NMR (101 MHz, DMSO-d_6): δ 170.6, 157.5, 156.2, 141.8, 133.9, 121.5, 114.9, 104.3, 61.6, 55.6, 45.0, 34.5. HRMS (ESI): *m/z* = calculated for $C_{13}H_{18}N_3O_2S$ [M+H]$^+$: 280.1115; found: 280.1113. Purity (HPLC): 98 % (λ = 210 nm), 97 % (λ = 254 nm), Method 1a.

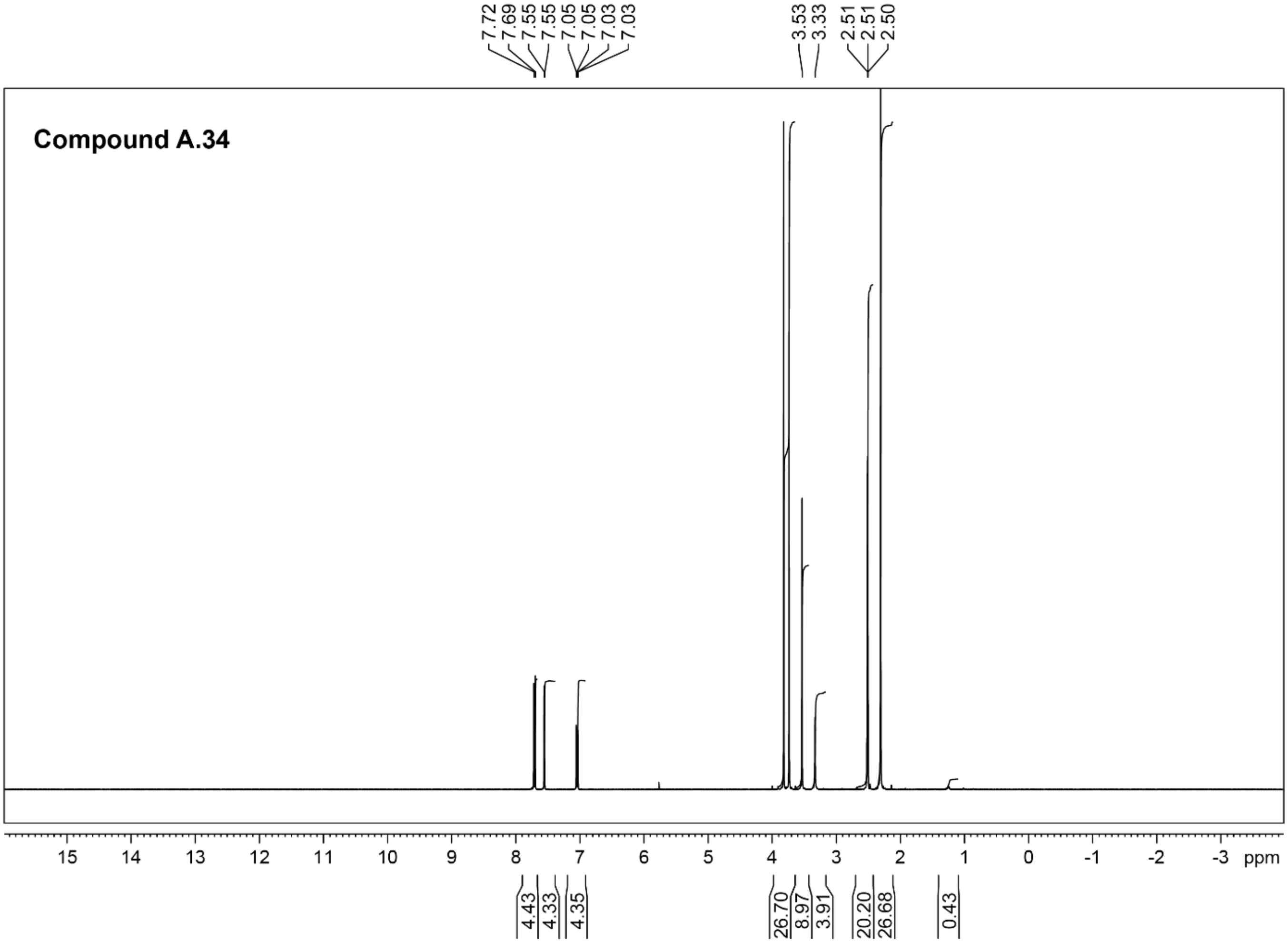

Synthesis Suppl. Figure 35. ^{1}H NMR of compound A.34.

2-(Dimethylamino)-*N*-(6-methoxybenzo[*d*]oxazol-2-yl)acetamide (A.35)

2-(Dimethylamino)acetamide **A.35** was prepared following General procedure F, using alkyl chloride **25** (229 mg, 0.950 mmol, 1.0 eq) and dimethylamine (1.0 mL, 2 M in THF, 2.1 mmol, 2.2 eq). The mixture was stirred for 4 h and the extraction was conducted with EtOAc (3 x 5 mL). The crude product was purified by flash column chromatography (96:3:1 DCM/MeOH/triethylamine), yielding product **A.35** (156 mg, 0.626 mmol, 67%) as an off-white solid Mp: 117 – 119 °C. ^{1}H NMR (400 MHz, DMSO-d_6): δ 11.21 (s, 1H), 7.46 (d, J = 8.7 Hz, 1H), 7.28 (d, J = 2.4 Hz, 1H), 6.90 (dd, J = 8.7, 2.4 Hz, 1H), 3.80 (s, 3H), 3.25 (s, 2H), 2.30 (s, 6H). ^{13}C NMR (101 MHz, DMSO-d_6): δ 168.4, 156.7, 154.1, 148.6, 134.0, 118.3, 111.9, 95.8, 62.1, 55.9, 45.0. HRMS (ESI): m/z = calculated for $C_{12}H_{16}N_5O_3$ $[M+H]^+$: 250.1187; found: 250.1187. Purity (HPLC): 100% (λ = 210 nm), 100% (λ = 254 nm), Method 1a.

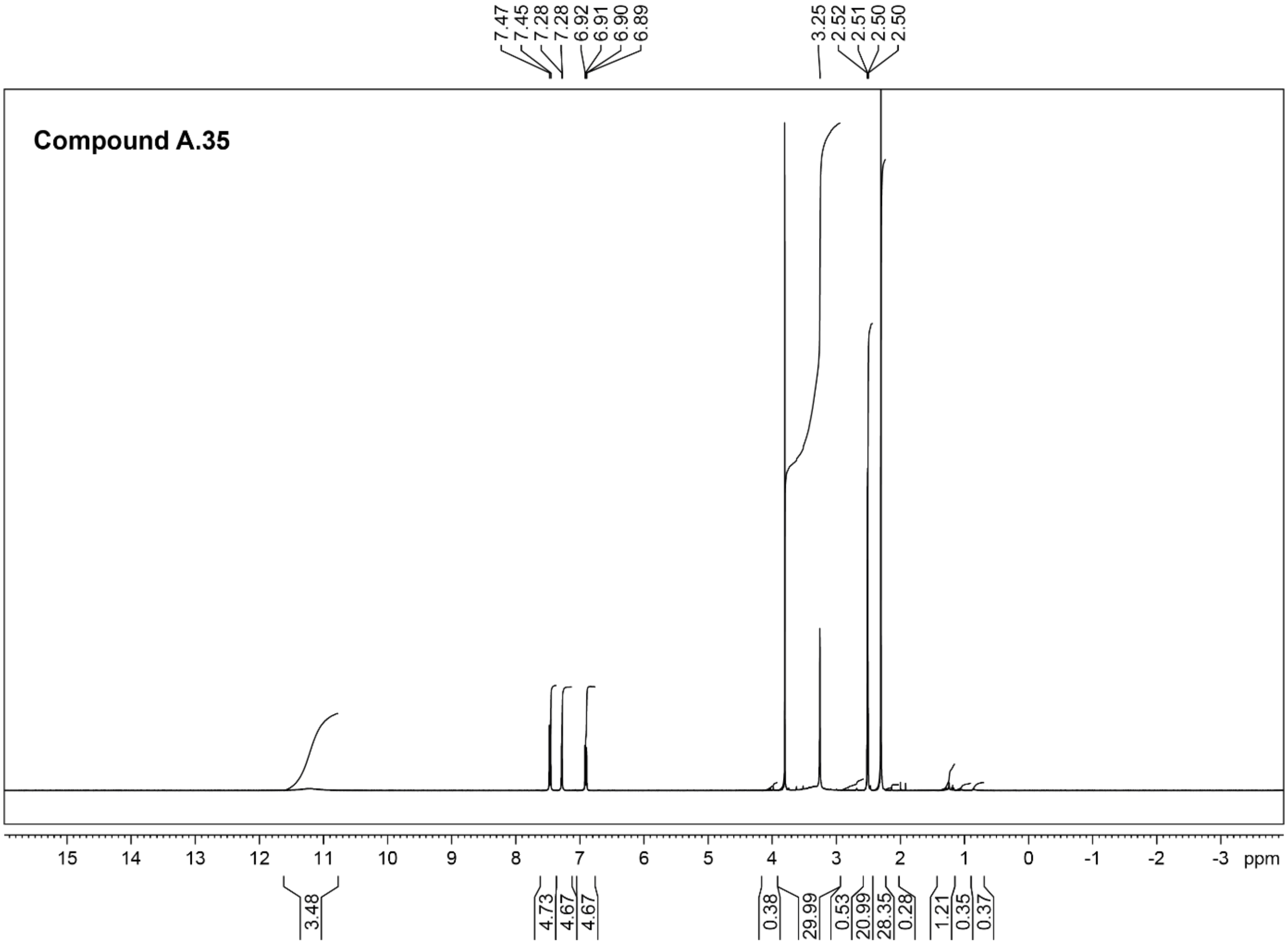

Synthesis Suppl. Figure 36. ^{1}H NMR of compound A.35.

(*S*)-2-(3-Aminopiperidin-1-yl)-*N*-(6-methoxybenzo[*d*]thiazol-2-yl)acetamide (A.36)

3-Aminopiperidine **A.36** was prepared following General procedure H from Boc-derivative **30** (336 mg, 0.800 mmol, 1.0 eq). The mixture was extracted with $CHCl_3$/isopropanol (3:1, 5 x 10 mL) and the residue purified by flash column chromatography (94:5:1 DCM/MeOH/triethylamine), yielding product **A.36** (205 mg, 0.640 mmol, 80%) as an off-white solid. Mp: 128 – 130 °C. ^{1}H NMR (400 MHz, DMSO-d_6): δ 7.62 (d, *J* = 8.8 Hz, 1H), 7.55 (d, *J* = 2.6 Hz, 1H), 7.02 (dd, *J* = 8.8, 2.6 Hz, 1H), 4.71 (s, 2H), 3.80 (s, 3H), 3.34 – 3.21 (m, 2H), 2.80 – 2.69 (m, 2H), 2.69 – 2.60 (m, 1H), 2.18 (dd, *J* = 11.7, 8.9 Hz, 1H), 1.96 (t, *J* = 9.9 Hz, 1H), 1.74 – 1.60 (m, 2H), 1.48 (td, *J* = 10.0, 5.1 Hz, 1H), 1.02 (q, *J* = 10.1 Hz, 1H). ^{13}C NMR (101 MHz, DMSO-d_6): δ 169.7, 156.1, 155.9, 142.6, 132.8, 121.0, 114.8, 104.7, 61.9, 60.8, 55.6, 53.1, 47.6, 32.8, 23.4. HRMS (ESI): *m/z* = calculated for $C_{15}H_{21}N_4O_2S$ $[M+H]^+$: 321.1380; found: 321.1384. Specific rotation: $[\alpha]_D^{20}$ = + 14.1 (c = 0.26). Purity (HPLC): > 95% (λ = 210 nm), > 92% (λ = 254 nm), Method 1a.

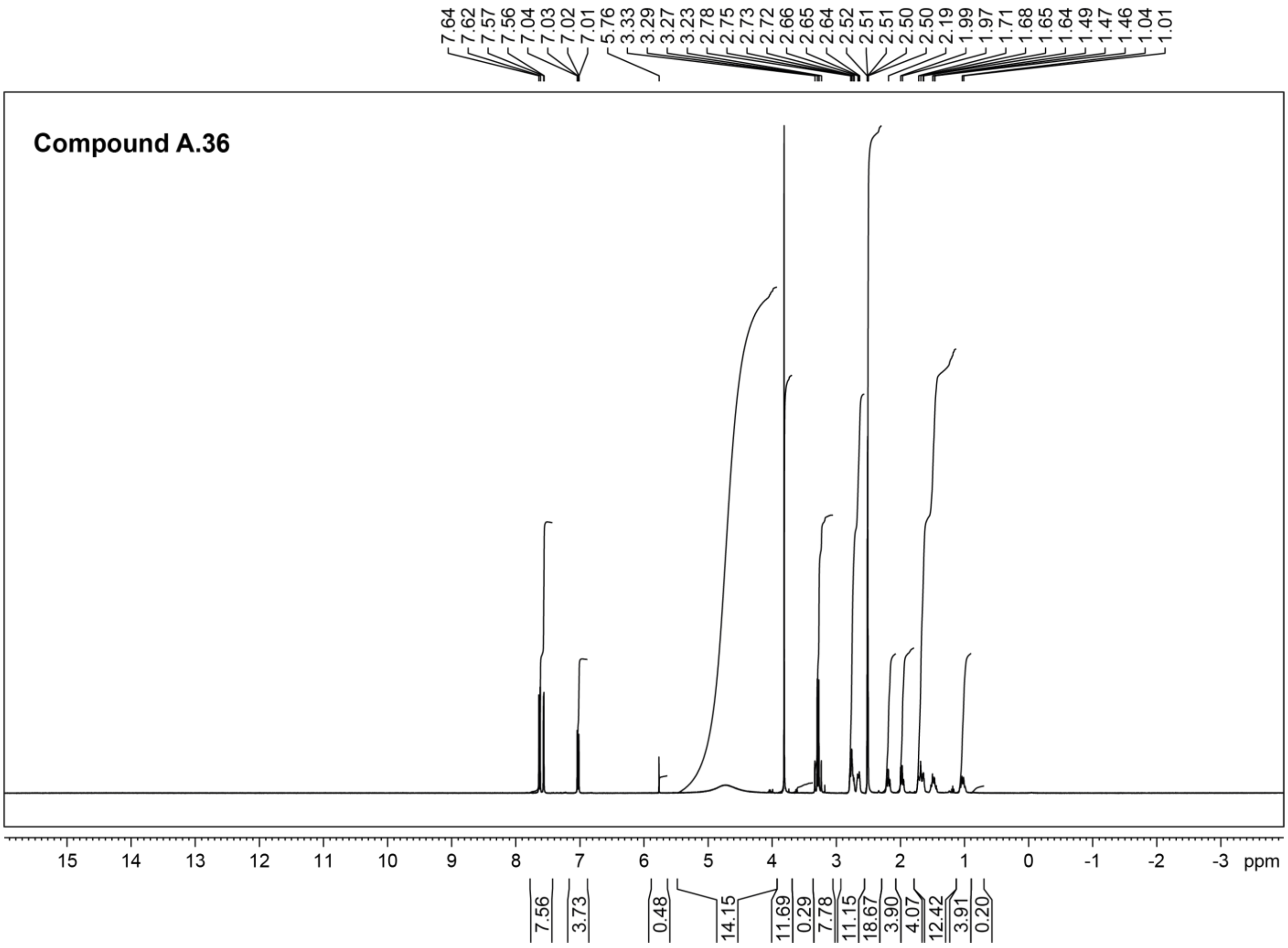

Synthesis Suppl. Figure 37. ^{1}H NMR of compound A.36.

(*R*)-2-(3-Aminopiperidin-1-yl)-*N*-(6-methoxybenzo[*d*]thiazol-2-yl)acetamide (A.37)

3-Aminopiperidine **A.37** was prepared following General procedure H from Boc-derivative **31** (421 mg, 1.00 mmol, 1.0 eq). The mixture was extracted with $CHCl_3$/isopropanol (3:1, 5 x 10 mL) and the residue purified by flash column chromatography (94:5:1 DCM/MeOH/triethylamine), yielding product **A.37** (277 mg, 0.865 mmol, 87%) as an off-white solid. Mp: 125 – 127 °C. ^{1}H NMR (500 MHz, DMSO-d_6): δ 7.62 (d, *J* = 8.8 Hz, 1H), 7.55 (d, *J* = 2.6 Hz, 1H), 7.02 (dd, *J* = 8.8, 2.6 Hz, 1H), 3.80 (s, 3H), 3.33 – 3.21 (m, 2H), 2.75 (s br, 2H), 2.68 – 2.61 (m, 1H), 2.22 – 2.14 (m, 1H), 1.99 – 1.92 (m, 1H), 1.73 – 1.60 (m, 2H), 1.52 – 1.40 (m, 1H), 1.02 (q, *J* = 11.0, 9.7 Hz, 1H). ^{13}C NMR (126 MHz, DMSO-d_6): δ 69.7, 156.1, 155.9, 142.6, 132.8, 121.0, 114.8, 104.7, 61.9, 60.8, 55.6, 53.1, 47.6, 32.9, 23.4. HRMS (ESI): *m/z* = calculated for $C_{15}H_{21}N_4O_2S$ $[M+H]^+$: 321.1380; found: 321.1383. Specific rotation: $[\alpha]_D^{20}$ = - 13.3 (c = 0.23). Purity (HPLC): > 95% (λ = 210 nm), > 91% (λ = 254 nm), Method 1a.

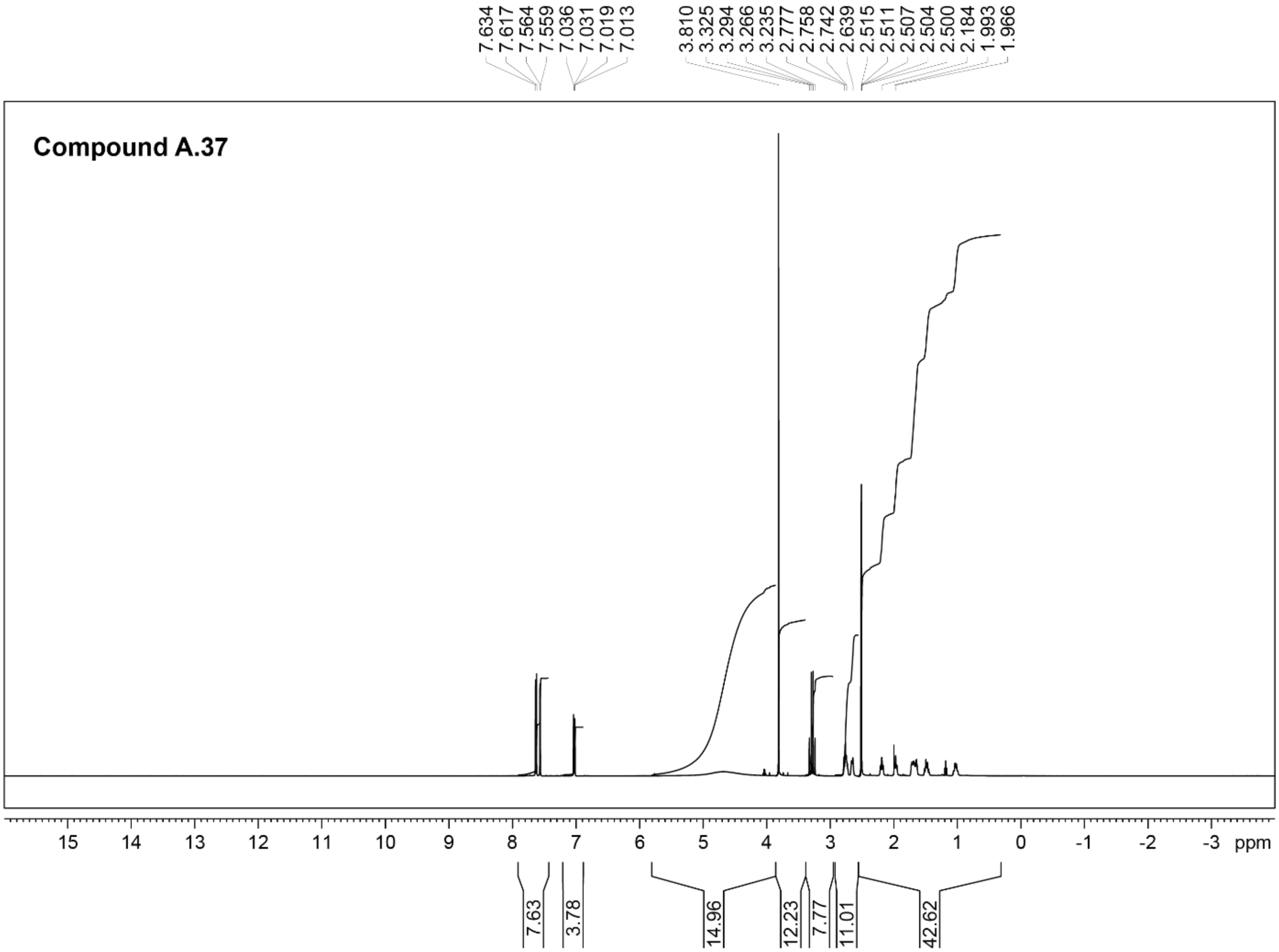

Synthesis Suppl. Figure 38. ^{1}H NMR of compound A.37.

2-(Dimethylamino)-*N*-(5-methoxythiazolo[5,4-*b*]pyridin-2-yl)acetamide (A.38)

2-(Dimethylamino)acetamide **A.38** was prepared following General procedure F, using crude alkyl chloride **32** (438 mg, 1.70 mmol, 1.0 eq) and dimethylamine (1.87 mL, 2 M in THF, 3.74 mmol, 2.2 eq). The mixture was stirred for 2 h and the extraction was conducted with EtOAc (3 x 20 mL). The crude product was purified by flash column chromatography (96:3:1 DCM/MeOH/triethylamine), yielding product **A.38** (292 mg, 1.10 mmol, 65%) as a beige solid. Mp: 115 – 117 °C. ^{1}H NMR (400 MHz, DMSO-*d*$_6$): δ 11.99 (s, 1H), 8.02 (d, *J* = 8.7 Hz, 1H), 6.90 (d, *J* = 8.8 Hz, 1H), 3.91 (s, 3H), 3.29 (s, 2H), 2.30 (s, 6H). ^{13}C NMR (101 MHz, DMSO-*d*$_6$): δ 169.6, 161.1, 154.1, 151.3, 136.8, 131.0, 109.6, 61.2, 53.8, 45.0. HRMS (ESI): *m/z* = calculated for $C_{11}H_{14}ClN_4O_2S$ [M+H]$^+$: 267.0911; found: 267.0913. Purity (HPLC): > 96% (λ = 210 nm), > 92% (λ = 254 nm), Method 1a.

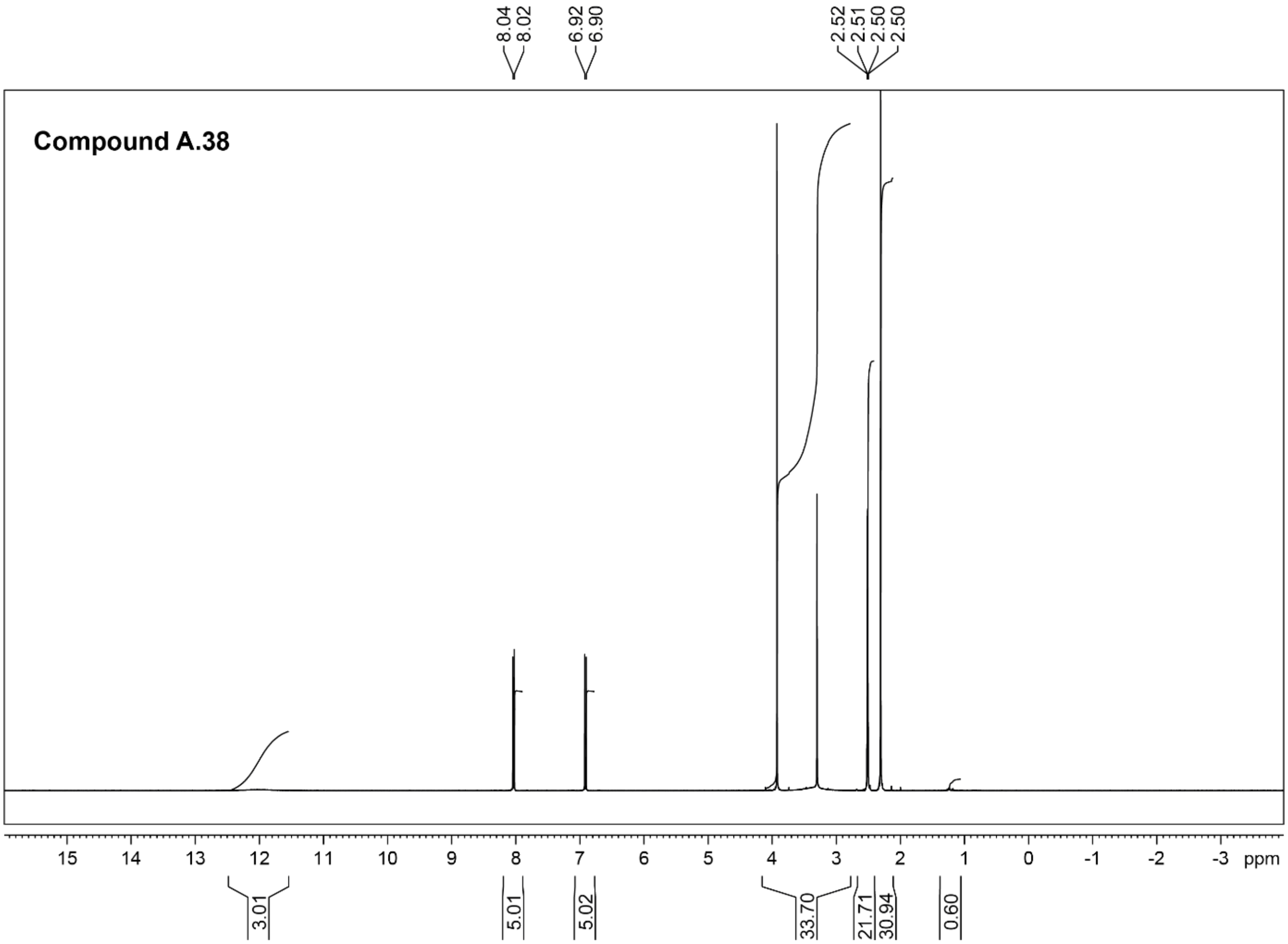

Synthesis Suppl. Figure 39. ^{1}H NMR of compound A.38.

N-(6-Methoxybenzo[d]thiazol-2-yl)-2-((2,2,6,6-tetramethylpiperidin-4-yl)amino)acetamide (A.39)

Amine **A.39** was prepared following General procedure F, using chloroacetamide **2** (257 mg, 1.00 mmol, 1.0 eq), 4-amino-2,2,6,6-tetramethylpiperidine (172 mg, 1.10 mmol, 1.1 eq), and triethylamine (0.153 mL, 1.10 mmol, 1.1 eq). The mixture was stirred for 2 h and the extraction was conducted with $CHCl_3$/isopropanol. The residue was purified by flash column chromatography (93:6:1 DCM/MeOH/triethylamine), yielding product **A.39** (176 mg, 0.467 mmol, 47%) as a colourless solid. Mp: 170 – 171 °C. ^{1}H NMR (500 MHz, DMSO-d_6): δ = 7.61 (d, J = 8.8 Hz, 1H), 7.55 (d, J = 2.6 Hz, 1H), 7.01 (dd, J = 8.8, 2.6 Hz, 1H), 3.80 (s, 3H), 3.49 (s, 2H), 2.87 (ddt, J = 11.6, 7.1, 3.5 Hz, 1H), 1.72 (dd, J = 12.4, 3.5 Hz, 2H), 1.09 (s, 6H), 1.02 (s, 6H), 0.84 (t, J = 11.9 Hz, 2H). ^{13}C NMR (126 MHz, DMSO-d_6): δ = 171.7, 156.1, 156.0, 142.7, 132.9, 121.0, 114.8, 104.8, 55.6, 50.6, 49.4, 49.1, 45.0, 34.6, 28.8. HRMS (ESI): m/z = calculated for $C_{19}H_{29}N_4O_2S$ $[M+H]^+$: 377.2006; found: 377.2008. Purity (HPLC): > 97% (λ = 210 nm), > 95% (λ = 254 nm), Method 1a.

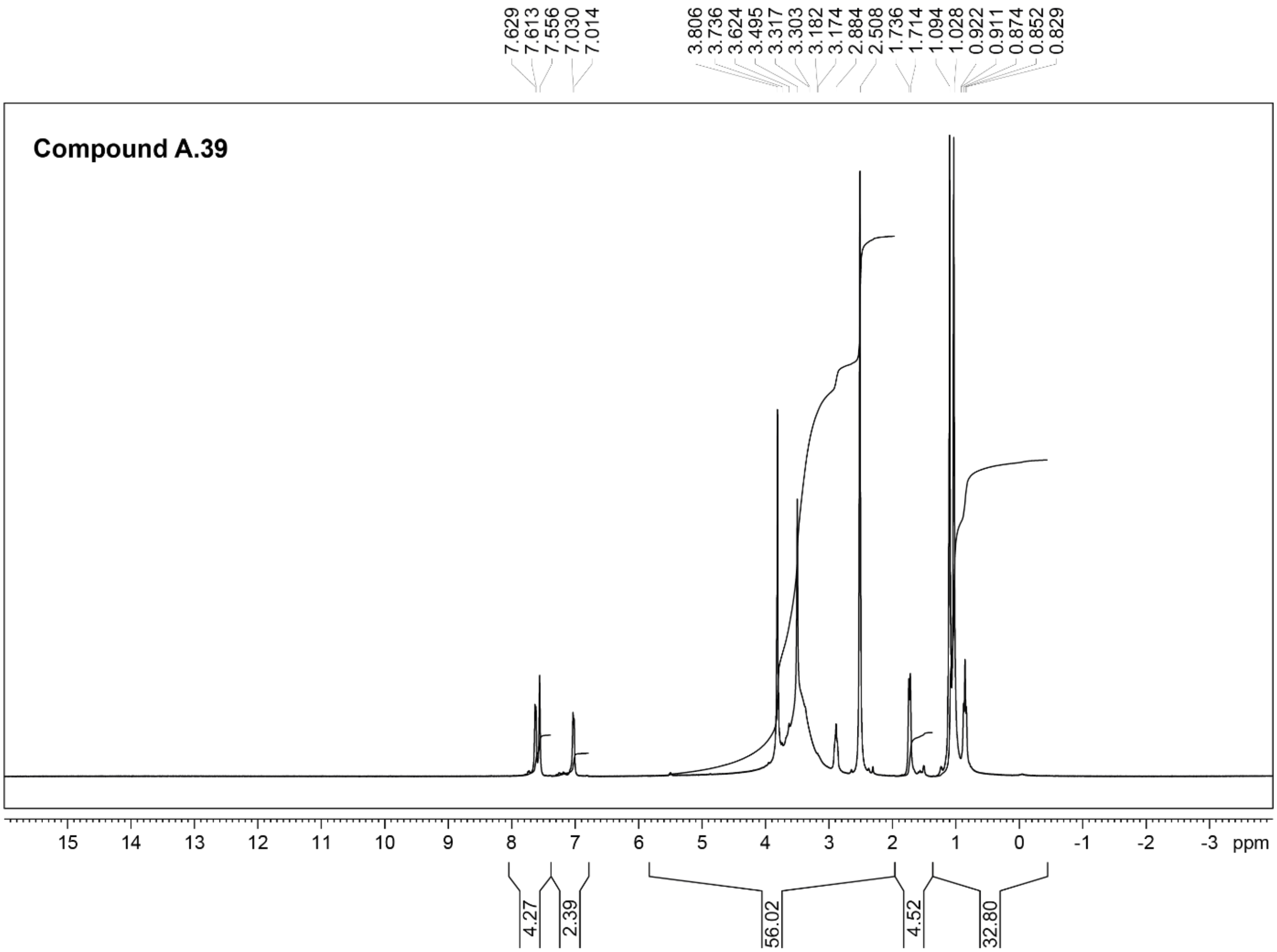

Synthesis Suppl. Figure 40. ^{1}H NMR of compound A.39.

***N*-(6-Methoxy-1*H*-benzo[*d*]imidazol-2-yl)-2-(4-methylpiperazin-1-yl)acetamide (A.40)**

Amine **A.40** was prepared following General procedure F, using chloroacetamide **8** (204 mg, 1.40 mmol, 1.0 eq) and 1-methylpiperazine (0.342, 3.08 mmol, 2.2 eq). The mixture was stirred for 1 h and the extraction was conducted with $CHCl_3$/isopropanol. The residue was purified by flash column chromatography (88:10:2 DCM/MeOH/triethylamine), yielding product **A.40** (204 mg, 0.672 mmol, 48%) as a colourless solid. Mp: 115 – 117 °C. ^{1}H NMR (400 MHz, DMSO-d_6): δ = 11.48 (s, 1H), 7.31 (d, *J* = 8.7 Hz, 1H), 6.99 (d, *J* = 2.4 Hz, 1H), 6.71 (dd, *J* = 8.7, 2.5 Hz, 1H), 3.74 (s, 3H), 3.25 (s, 2H), 2.55 (s br, 4H), 2.35 (s br, 4H), 2.16 (s, 3H). ^{13}C NMR (101 MHz, DMSO-d_6): δ = 169.3, 155.0, 109.7, 60.5, 55.4, 54.7, 52.5, 45.8. HRMS (ESI): *m/z* = calculated for $C_{15}H_{22}N_5O_2$ $[M+H]^+$: 304.1768; found: 304.1775. Purity (HPLC): 100% (λ = 210 nm), 100% (λ = 254 nm), Method 1a.

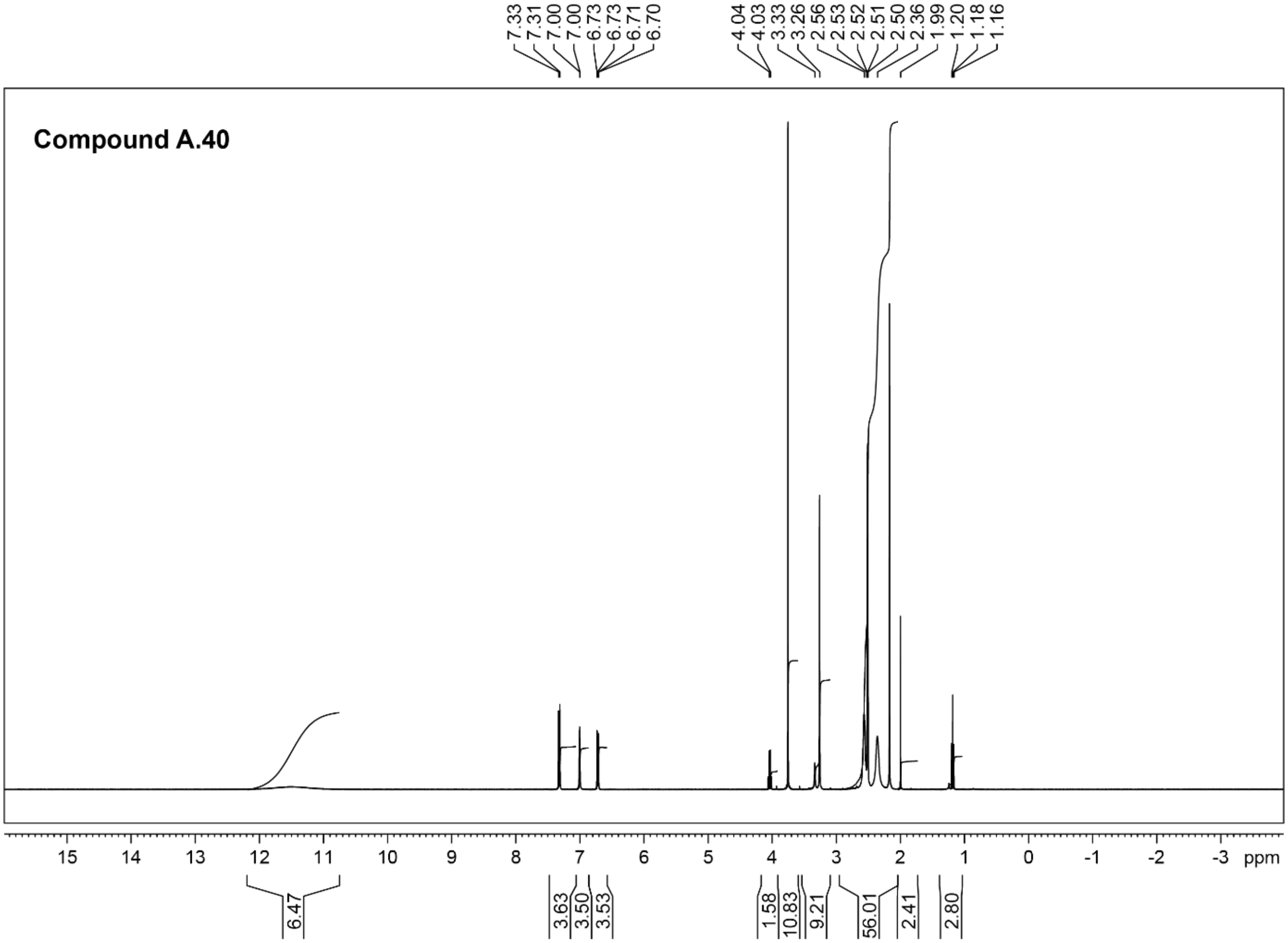

Synthesis Suppl. Figure 41. ^{1}H NMR of compound A.40.

2-(Dimethylamino)-*N*-(5-(3,5-dinitrophenyl)-1,3,4-thiadiazol-2-yl)acetamide (A.41)

2-(Dimethylamino)acetamide **A.41** was prepared following General procedure E and F, without isolating the chloroacetamide intermediate. 5-(3,5-Dinitrophenyl)-1,3,4-thiadiazol-2-amine (100 mg, 0.374 mmol, 1.0 eq) was suspended in THF and 2-chloroacetyl chloride (33 µL, 0.41 mmol, 1.1 eq) and triethylamine (58 µL, 0.41 mmol, 1.1 eq) were added to the solution. After stirring at room temperature for 24 h, dimethylamine (0.374 mL, 2 M in THF, 0.748 mmol, 2.0 eq) was added and the mixture was stirred for additional 18 h. The extraction was conducted with DCM and the residue was purified by flash column chromatography (DCM → 9:1 DCM/MeOH), yielding product **A.41** (48 mg, 0.17 mmol, 36%) as a yellow solid. Mp: 220 °C (decomposition), 230 – 232 °C (melting). ^{1}H NMR (400 MHz, DMSO-d_6) δ 8.94 (d, *J* = 2.0 Hz, 2H), 8.84 (t, *J* = 2.1 Hz, 1H), 3.62 (s, 2H), 2.56 (s, 6H). ^{13}C NMR (126 MHz, DMSO-d_6) δ 169.3, 164.5, 156.3, 148.7, 134.1, 125.9, 118.6, 61.0, 44.2. HRMS (ESI): *m/z* = calculated for $C_{12}H_{13}N_6O_5S$ $[M+H]^+$: 353.0663; found: 353.0671. Purity (HPLC): 100% (λ = 210 nm), 100% (λ = 254 nm), Method 3a.

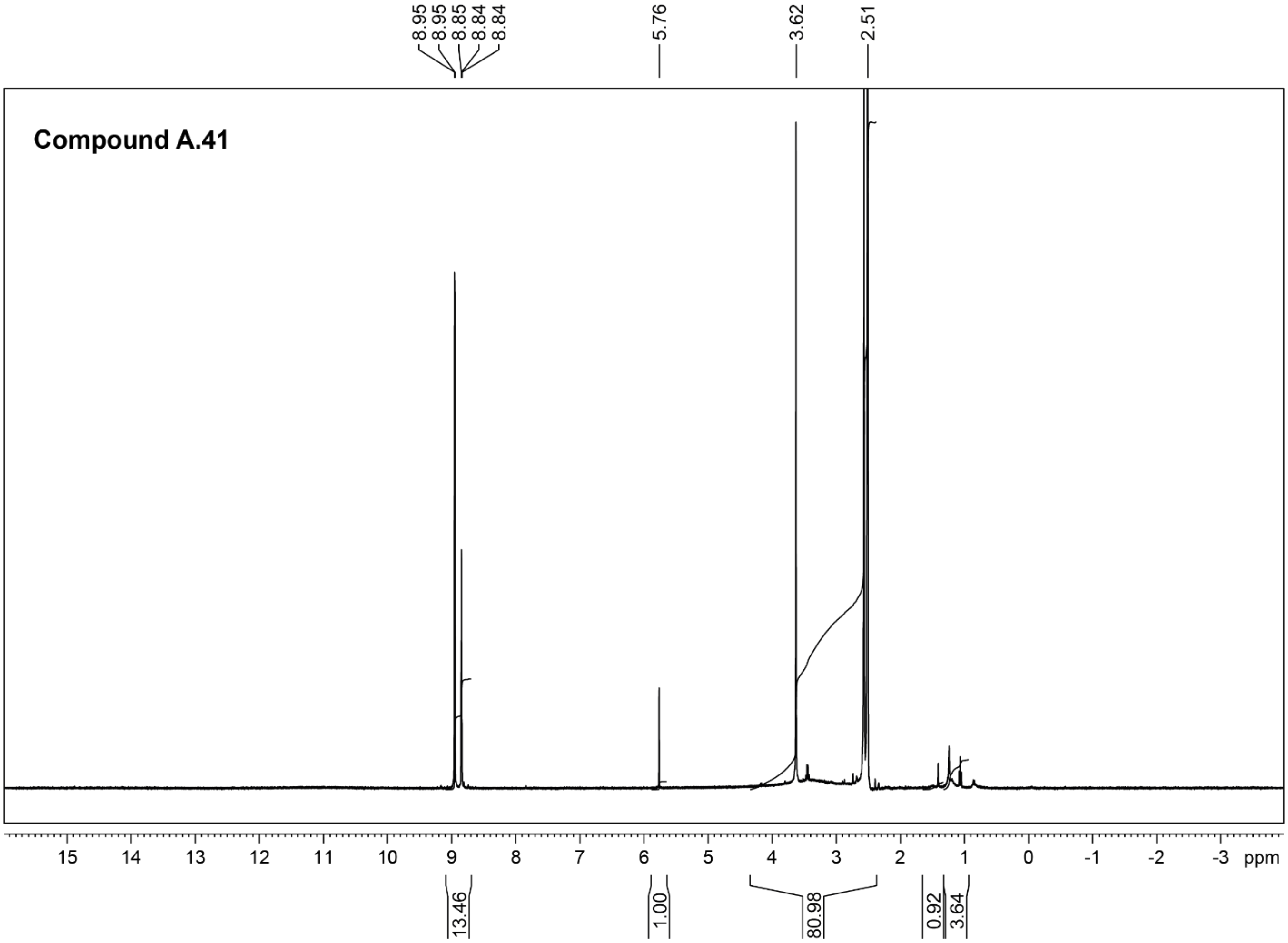

Synthesis Suppl. Figure 42. ^{1}H NMR of compound A.41.

2-(dimethylamino)-N-(5-(4-nitrophenyl)-1,3,4-thiadiazol-2-yl)acetamide (A.42)

2-(Dimethylamino)acetamide **A.42** was prepared following General procedure E and F, without isolating the chloroacetamide intermediate. 5-(4-Nitrophenyl)-1,3,4-thiadiazol-2-amine (100 mg, 0.450 mmol, 1.0 eq) was suspended in THF and 2-chloroacetyl chloride (39 µL, 0.50 mmol, 1.1 eq) and triethylamine (70 µL, 0.50 mmol, 1.1 eq) were added to the solution. After stirring at room temperature for 24 h, dimethylamine (0.45 mL, 2 M in THF, 0.90 mmol, 2.0 eq) was added and the mixture was stirred for additional 18 h. The extraction was conducted with DCM and the residue was purified by flash column chromatography (DCM → 9:1 DCM/MeOH), yielding product **A.41** (23 mg, 0.075 mmol, 17%) as a yellow solid. Mp: 204 °C (decomposition), 224 – 226 °C (melting). ^{1}H NMR (400 MHz, DMSO-*d*$_6$) δ 8.37 – 8.31 (m, 2H), 8.24 – 8.16 (m, 2H), 3.52 (s, 2H), 2.45 (s, 6H). ^{13}C NMR (126 MHz, DMSO-*d*$_6$) δ 169.2, 162.3, 158.7, 147.9, 136.7, 127.7, 124.5, 61.1, 44.5. HRMS (EI): *m/z* = calculated for $C_{12}H_{13}N_5O_3S$ $[M]^{\cdot+}$: 307.0734; found: 307.0721. Purity (HPLC): > 97% (λ = 210 nm), > 96% (λ = 254 nm), Method 3a.

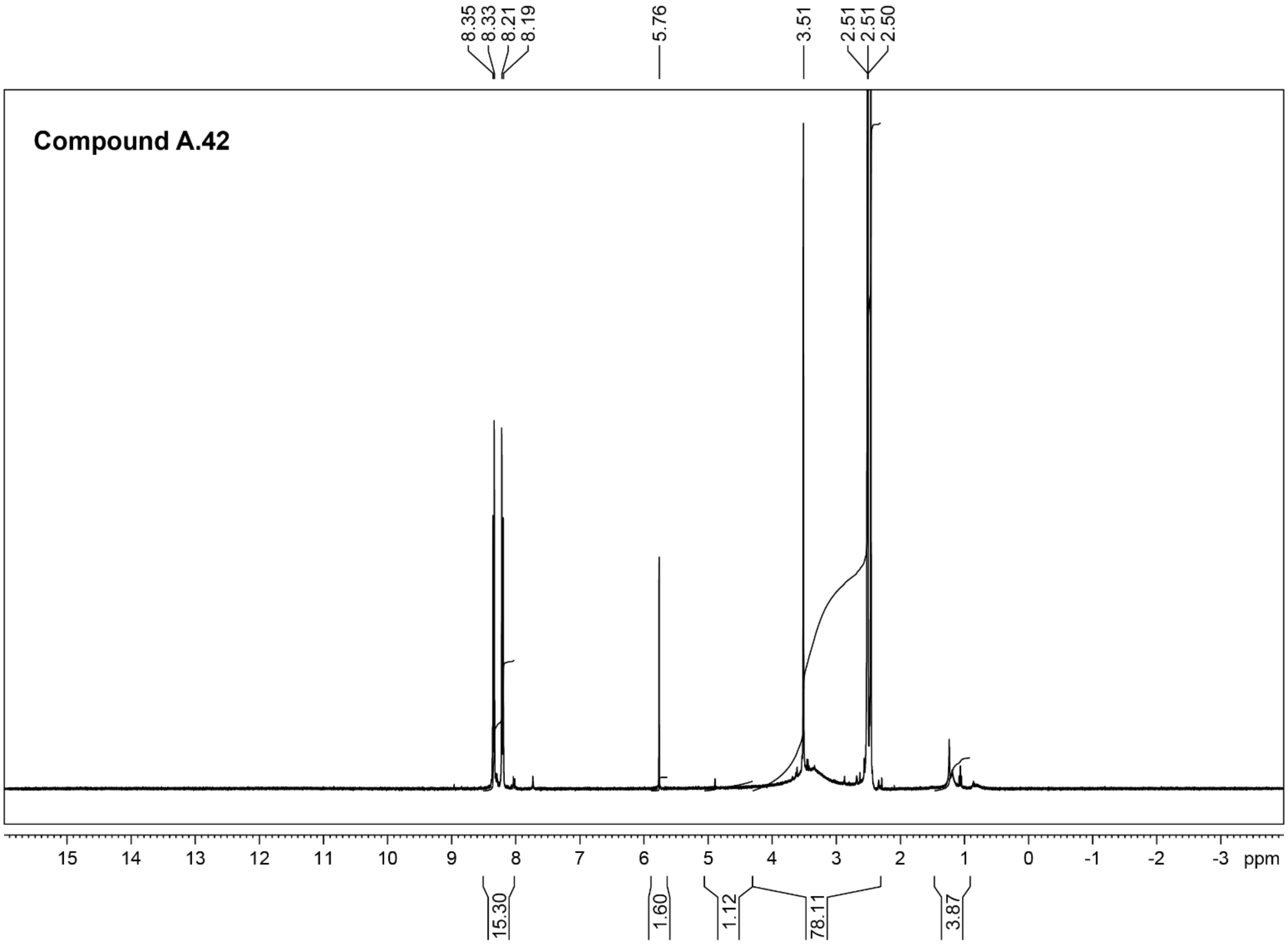

Synthesis Suppl. Figure 43. ^{1}H NMR of compound A.42.

_N_-(5-(2,4-Dichloro-5-fluoro-3-nitrophenyl)-1,3,4-thiadiazol-2-yl)-2-(dimethylamino)acetamide (A.43)

2-(Dimethylamino)acetamide **A.43** was prepared following General procedure E and F, without isolating the chloroacetamide intermediate. 5-(2,4-Dichloro-5-fluoro-3-nitrophenyl)-1,3,4-thiadiazol-2-amine (100 mg, 0.324 mmol, 1.0 eq) was suspended in THF and 2-chloroacetyl chloride (28 µL, 0.36 mmol, 1.1 eq) and triethylamine (50 µL, 0.36 mmol, 1.1 eq) were added to the solution. After stirring at room temperature for 24 h, dimethylamine (0.324 mL, 2 M in THF, 0.647 mmol, 2.0 eq) was added and the mixture was stirred for additional 18 h. The extraction was conducted with DCM and the residue was purified by flash column chromatography (DCM → 9:1 DCM/MeOH), yielding product **A.43** (74 mg, 0.19 mmol, 58%) as a yellow solid. Mp: 197 – 199 °C. ^{1}H NMR (400 MHz, DMSO-d_6) δ 8.42 (d, J = 9.8 Hz, 1H), 3.58 (s, 2H), 2.52 (s, 6H). ^{13}C NMR (126 MHz, DMSO-d_6) δ 169.2, 164.9, 157.2, 155.2, 153.5, 153.5, 148.9, 132.4, 132.3, 119.4, 119.2, 118.3, 118.3, 114.8, 114.6, 60.9, 44.3. HRMS (ESI): _m/z_ = calculated for $C_{12}H_{11}Cl_2FN_5O_3S$ [M+H]$^+$: 393.9939; found: 393.9945. Purity (HPLC): > 97% (λ = 210 nm), > 96% (λ = 254 nm), Method 3e.

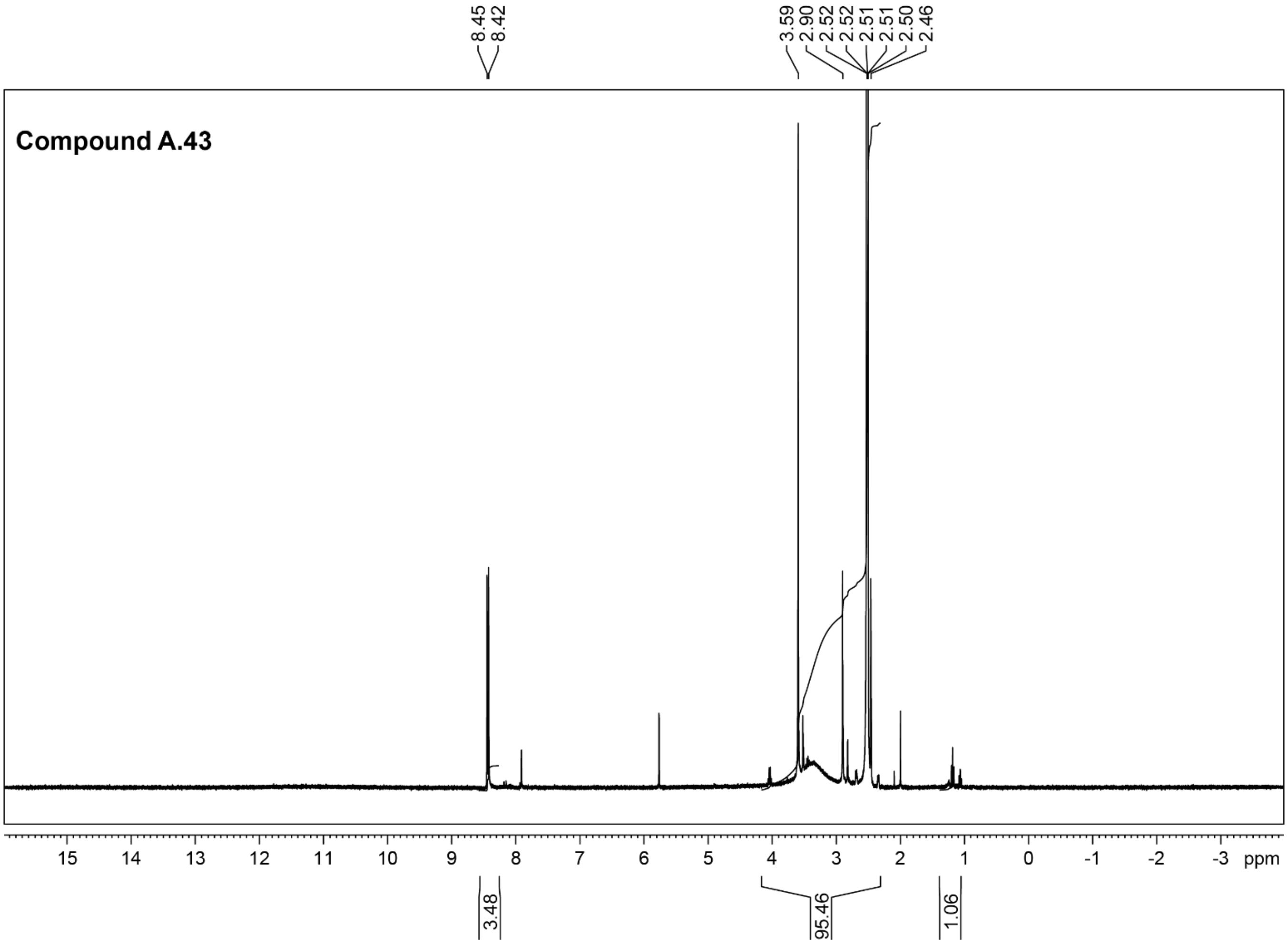

Synthesis Suppl. Figure 44. ^{1}H NMR of compound A.43.

***N*-(6-Methoxybenzo[*d*]thiazol-2-yl)-2-(methyl(2,2,6,6-tetramethylpiperidin-4-yl)amino)acetamide (A.44)**

Amine **A.44** was prepared following General procedure F, using chloroacetamide **2** (257 mg, 1.00 mmol, 1.0 eq), *N*,2,2,6,6-pentamethylpiperidin-4-amine (0.213 mL, 1.10 mmol, 1.1 eq), and triethylamine (0.153 mL, 1.10 mmol, 1.1 eq). The mixture was stirred for 4 h and the extraction was conducted with $CHCl_3$/isopropanol. The residue was purified by flash column chromatography (96:3:1 DCM/MeOH/triethylamine), yielding product **A.44** (187 mg, 0.479 mmol, 48%) as a pale yellow solid. Mp: 47 – 48 °C. ^{1}H NMR (400 MHz, DMSO-d_6): δ = 7.62 (d, J = 8.8 Hz, 1H), 7.56 (d, J = 2.6 Hz, 1H), 7.03 (dd, J = 8.8, 2.6 Hz, 1H), 3.80 (s, 3H), 3.36 (s, 2H), 3.04 – 2.93 (m, 1H), 2.30 (s, 3H), 1.62 (dd, J = 12.3, 3.1 Hz, 2H), 1.11 (s, 6H), 1.06 – 0.96 (m, 8H). ^{13}C NMR (101 MHz, DMSO-d_6): δ = 170.7, 156.1, 155.5, 142.6, 132.8, 121.1, 114.9, 104.8, 56.6, 55.6, 55.0, 50.7, 40.3, 38.2, 34.8, 28.7. HRMS (ESI): m/z = calculated for $C_{20}H_{31}N_4O_2S$ [M+H]$^+$: 391.2163; found: 391.2177. Purity (HPLC): 100% (λ = 210 nm), 100% (λ = 254 nm), Method 1a.

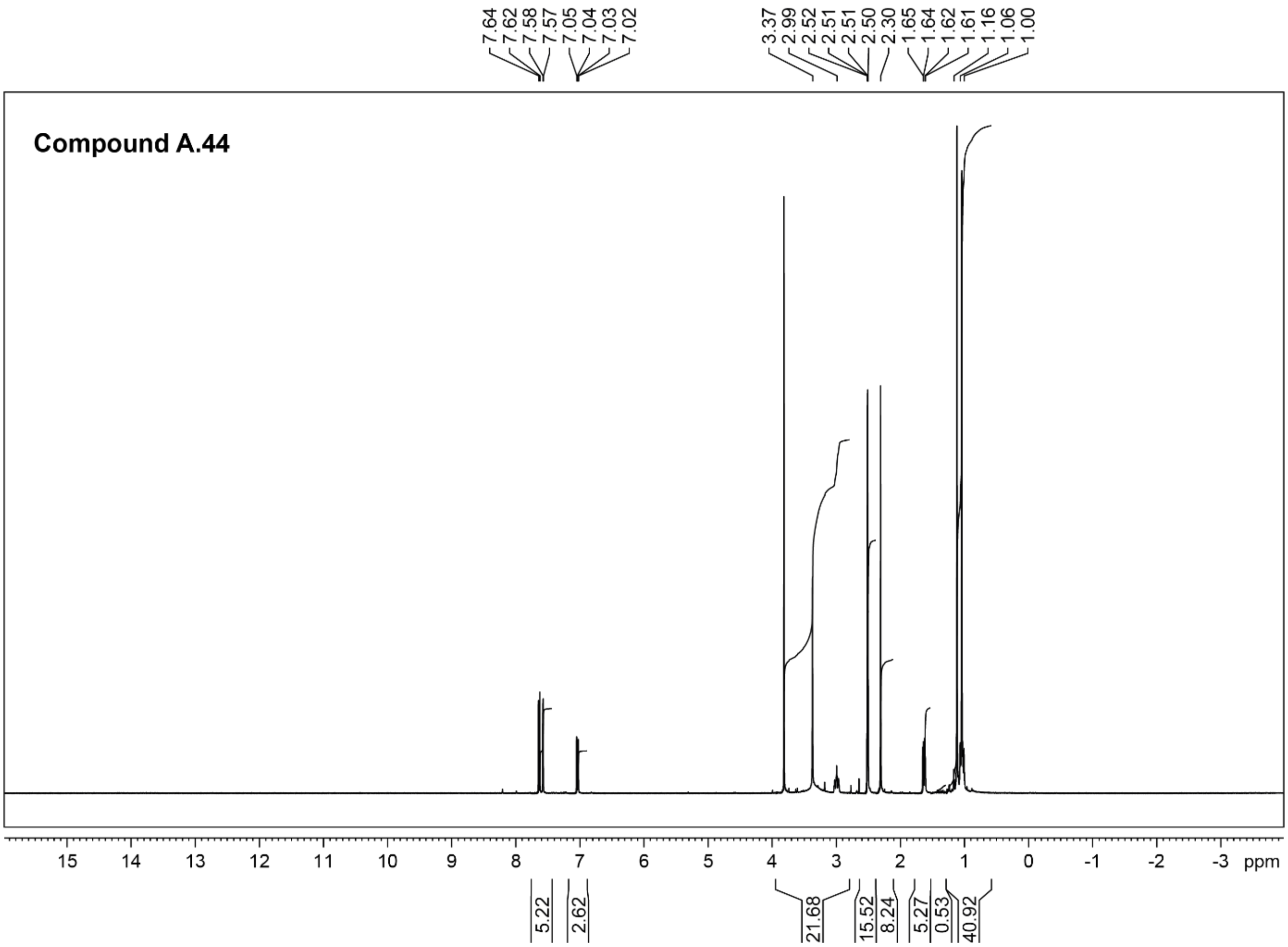

Synthesis Suppl. Figure 45. ^{1}H NMR of compound A.44.

N-(6-Methoxybenzo[d]thiazol-2-yl)-2-(piperazin-1-yl)acetamide (A.45)

Amine **A.45** was prepared following General procedure F, using chloroacetamide **2** (257 mg, 1.00 mmol, 1.0 eq), piperazine (96 mg, 1.1 mmol, 1.1 eq) and triethylamine (0.153 mL, 1.10 mmol, 1.1 eq). The mixture was stirred for 2 h and the extraction was conducted with $CHCl_3$/isopropanol. The residue was purified by flash column chromatography (88:10:2 DCM/MeOH/triethylamine), yielding product **A.45** (70 mg, 0.23 mmol, 23%) as a colourless solid. Mp: 243 – 245 °C (decomposition and melting). ^{1}H NMR (400 MHz, DMSO-d_6) δ 7.63 (d, J = 8.8 Hz, 1H), 7.57 (d, J = 2.6 Hz, 1H), 7.03 (dd, J = 8.8, 2.6 Hz, 1H), 3.80 (s, 3H), 3.27 (s, 2H), 2.72 (t, J = 4.8 Hz, 4H), 2.45 (t, J = 4.5 Hz, 4H). ^{13}C NMR (101 MHz, DMSO-d_6) δ 169.2, 156.1, 155.5, 142.5, 132.8, 121.1, 114.9, 104.7, 61.0, 55.61, 53.8, 45.5. HRMS (ESI): m/z = calculated for $C_{14}H_{19}N_4O_2S$ $[M+H]^+$: 307.1224; found: 307.1229. Purity (HPLC): 100% (λ = 210 nm), 100% (λ = 254 nm), Method 1a.

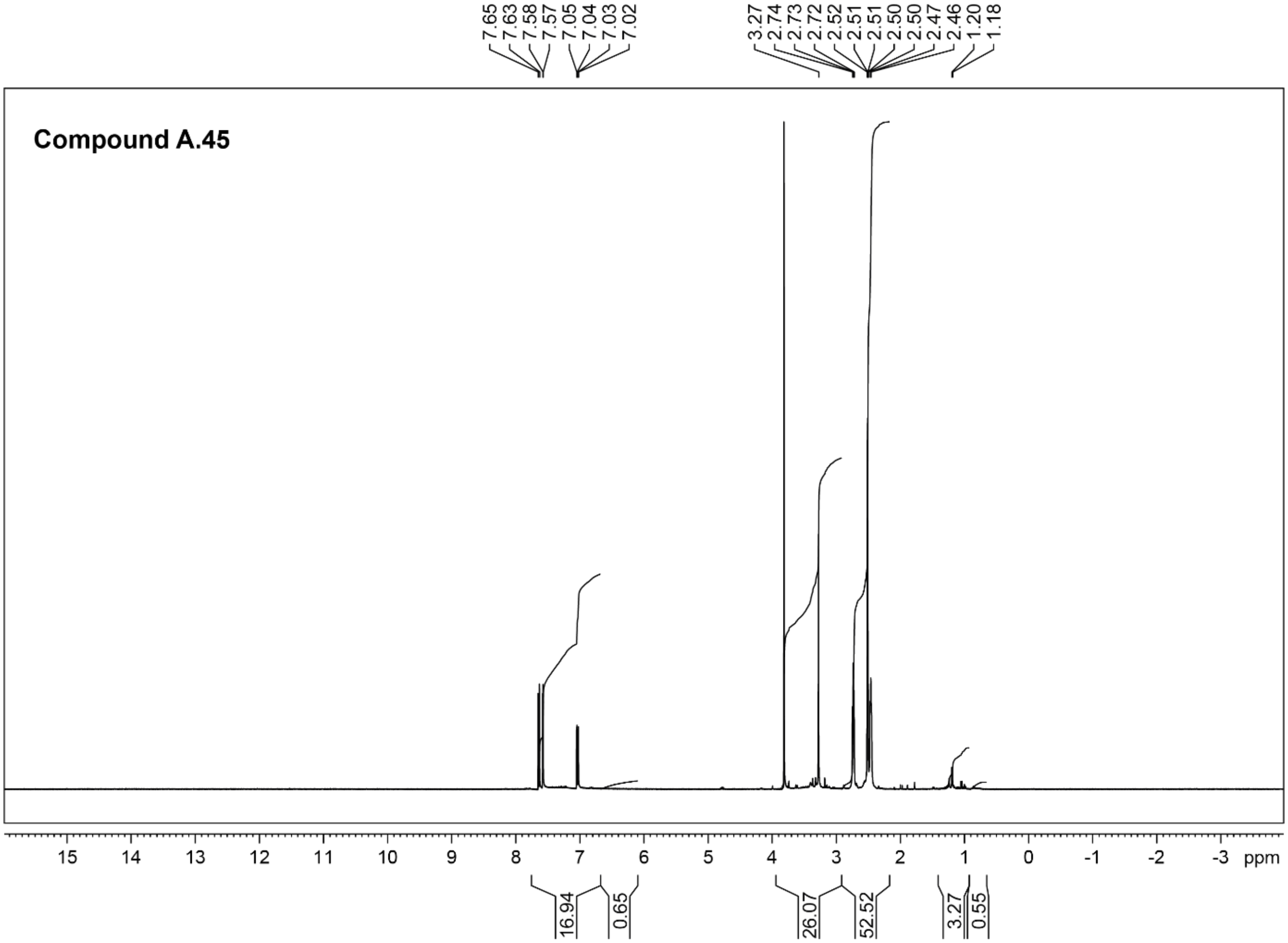

Synthesis Suppl. Figure 46. ^{1}H NMR of compound A.45.

N-(5-Chlorothiazolo[5,4-d]pyrimidin-2-yl)-2-(dimethylamino)acetamide (A.46)

2-(Dimethylamino)acetamide **A.46** was prepared following General procedure F, using alkyl chloride **35** (342 mg, 1.30 mmol, 1.0 eq) and dimethylamine (1.43 mL, 2 M in THF, 2.86 mmol, 2.2 eq). The mixture was stirred for 2 h and the extraction was conducted with EtOAc (3 x 20 mL). The crude product was purified by flash column chromatography (96:3:1 DCM/MeOH/triethylamine), yielding product **A.46** (185 mg, 0.681 mmol, 52%) as an off-white solid. Mp: 180 – 181 (decomposition), 248 – 250 °C (melting). ^{1}H NMR (400 MHz, DMSO-d_6): δ 8.84 (s, 1H), 3.69 (s, 2H), 2.61 (s, 6H). ^{13}C NMR (101 MHz, DMSO-d_6): δ 170.9, 166.4, 163.1, 151.4, 147.2, 141.0, 61.0, 44.1. HRMS (ESI): *m/z* = calculated for $C_9H_{11}ClN_5OS$ [M+H]$^+$: 272.0368; found: 272.0373. Purity (HPLC): > 97% (λ = 210 nm), > 97% (λ = 254 nm), Method 3c.

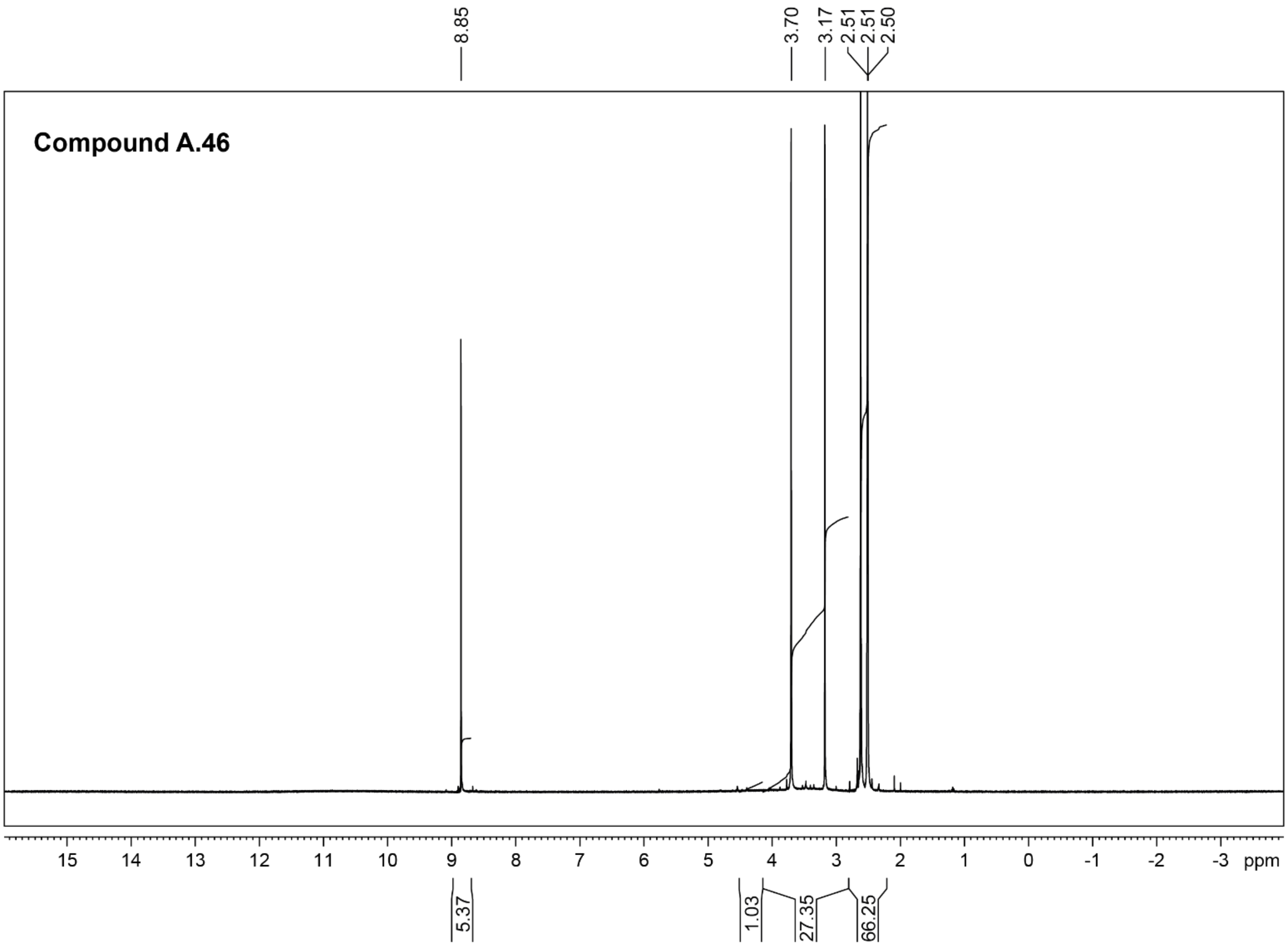

Synthesis Suppl. Figure 47. ^{1}H NMR of compound A.46.

2-(Dimethylamino)-*N*-(5-methoxythiazolo[5,4-*d*]pyrimidin-2-yl)acetamide (A.47)

2-(Dimethylamino)acetamide **A.47** was prepared following General procedure F, using alkyl chloride **38** (199 mg, 0.770 mmol, 1.0 eq) and dimethylamine (0.85 mL, 2 M in THF, 1.7 mmol, 2.2 eq). The mixture was stirred for 1 h and the extraction was conducted with $CHCl_3$/isopropanol (3:1, 5 x 15 mL). The crude product was purified by flash column chromatography (94:5:1 DCM/MeOH/triethylamine), yielding product **A.47** (80 mg, 0.30 mmol, 39%) as a colourless solid. Mp: 220 – 223 °C. ^{1}H NMR (500 MHz, DMSO-*d*$_6$): δ 11.90 (s, 1H), 8.89 (s, 1H), 3.96 (s, 3H), 3.39 (s, 2H), 2.36 (s, 6H). ^{13}C NMR (126 MHz, DMSO-*d*$_6$): δ 170.3, 165.3, 161.2, 156.1, 148.9, 136.4, 61.2, 55.0, 44.8. HRMS (EI): *m/z* = calculated for $C_{10}H_{13}N_5O_2S$ $[M]^{\cdot+}$: 267.0790; found: 267.0784. Purity (HPLC): 100% (λ = 210 nm), 100% (λ = 254 nm), Method 3b.

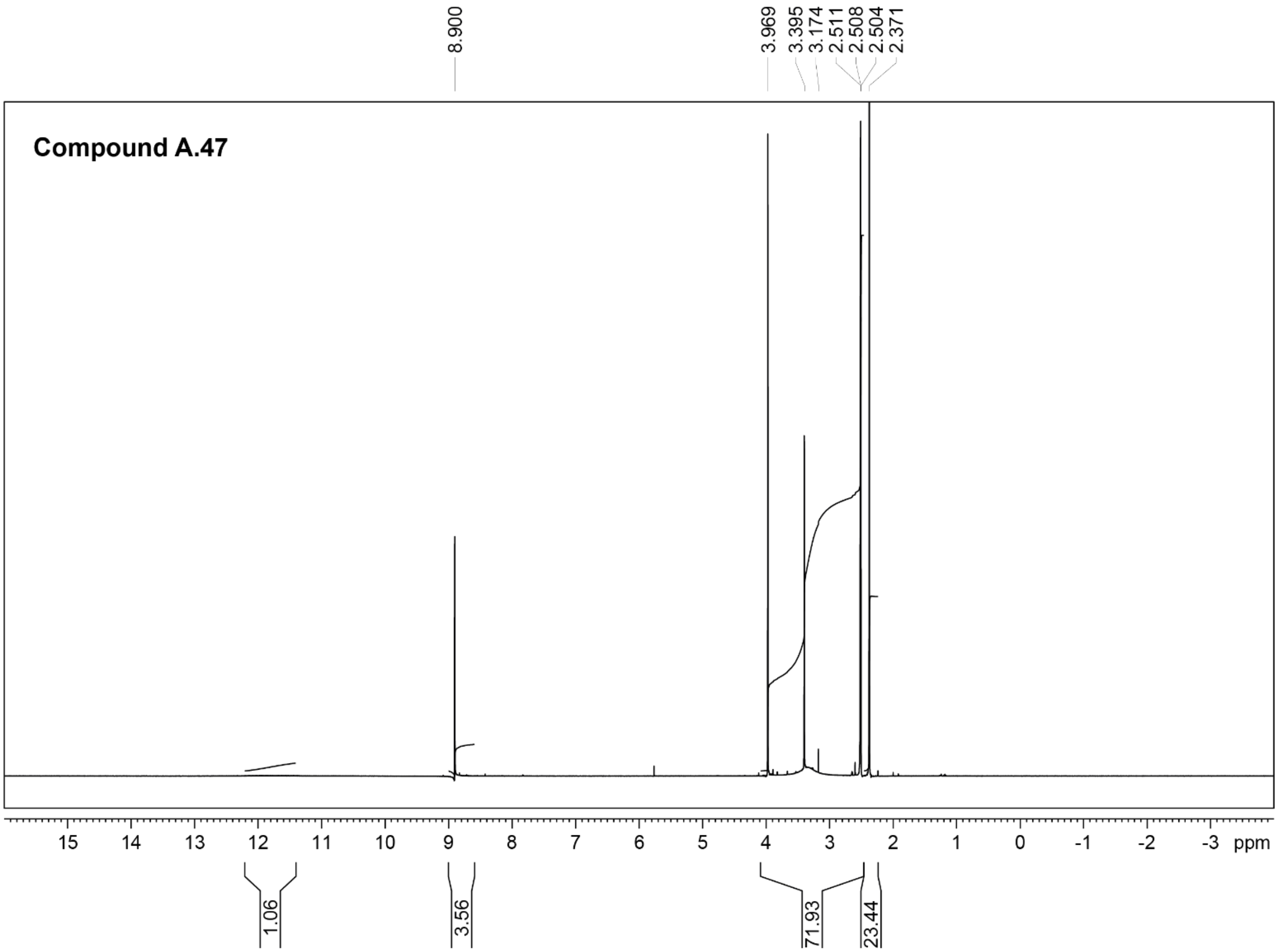

Synthesis Suppl. Figure 48. ^{1}H NMR of compound A.47.

(*S*)-2-(3-((2-((3-Hydroxyphenyl)(methyl)amino)ethyl)amino)piperidin-1-yl)-*N*-(6-methoxybenzo[*d*]thiazol-2-yl)acetamide (A.48)

Phenol **A.48** was prepared following General procedure N from nosyl derivative **45** (78 mg, 0.12 mmol, 1.0 eq). Off-white solid (43 mg, 0.091 mmol, 76%). Mp: 76 – 78 °C. ^{1}H NMR (500 MHz, DMSO-d_6) δ 8.94 (s, 1H), 7.63 (d, *J* = 8.8 Hz, 1H), 7.56 (d, *J* = 2.6 Hz, 1H), 7.03 (dd, *J* = 8.8, 2.6 Hz, 1H), 6.88 (t, *J* = 8.1 Hz, 1H), 6.12 (dd, *J* = 8.3, 2.4 Hz, 1H), 6.07 (t, *J* = 2.3 Hz, 1H), 6.01 (dd, *J* = 7.9, 2.1 Hz, 1H), 3.81 (s, 3H), 3.33 – 3.21 (m, 6H), 2.89 – 2.82 (m, 1H), 2.81 (s, 3H), 2.67 (t, *J* = 7.2 Hz, 3H), 2.58 (tt, *J* = 8.3, 3.7 Hz, 1H), 2.30 – 2.17 (m, 1H), 2.04 (t, *J* = 9.6 Hz, 1H), 1.74 (d, *J* = 12.4 Hz, 1H), 1.64 (dt, *J* = 12.9, 4.0 Hz, 1H), 1.47 (qd, *J* = 10.2, 6.0 Hz, 1H), 1.05 (d, *J* = 11.0 Hz, 1H). ^{13}C NMR (126 MHz, DMSO-d_6) δ 169.6, 158.2, 156.1, 155.5, 150.6, 142.6, 132.8, 129.5, 121.1, 114.9, 104.7, 103.2, 103.1, 98.9, 60.7, 59.1, 55.6, 53.8, 53.3, 52.6, 43.3, 38.2, 30.3, 23.3. HRMS (ESI): *m/z* = calculated for $C_{24}H_{32}N_5O_3S$ [M+H]$^+$: 470.2221; found: 470.2213. Specific rotation: $[\alpha]_D^{20}$ = + 8.8 (c = 0.19). Purity (HPLC): > 97% (λ = 210 nm), > 97% (λ = 254 nm), Method 1c.

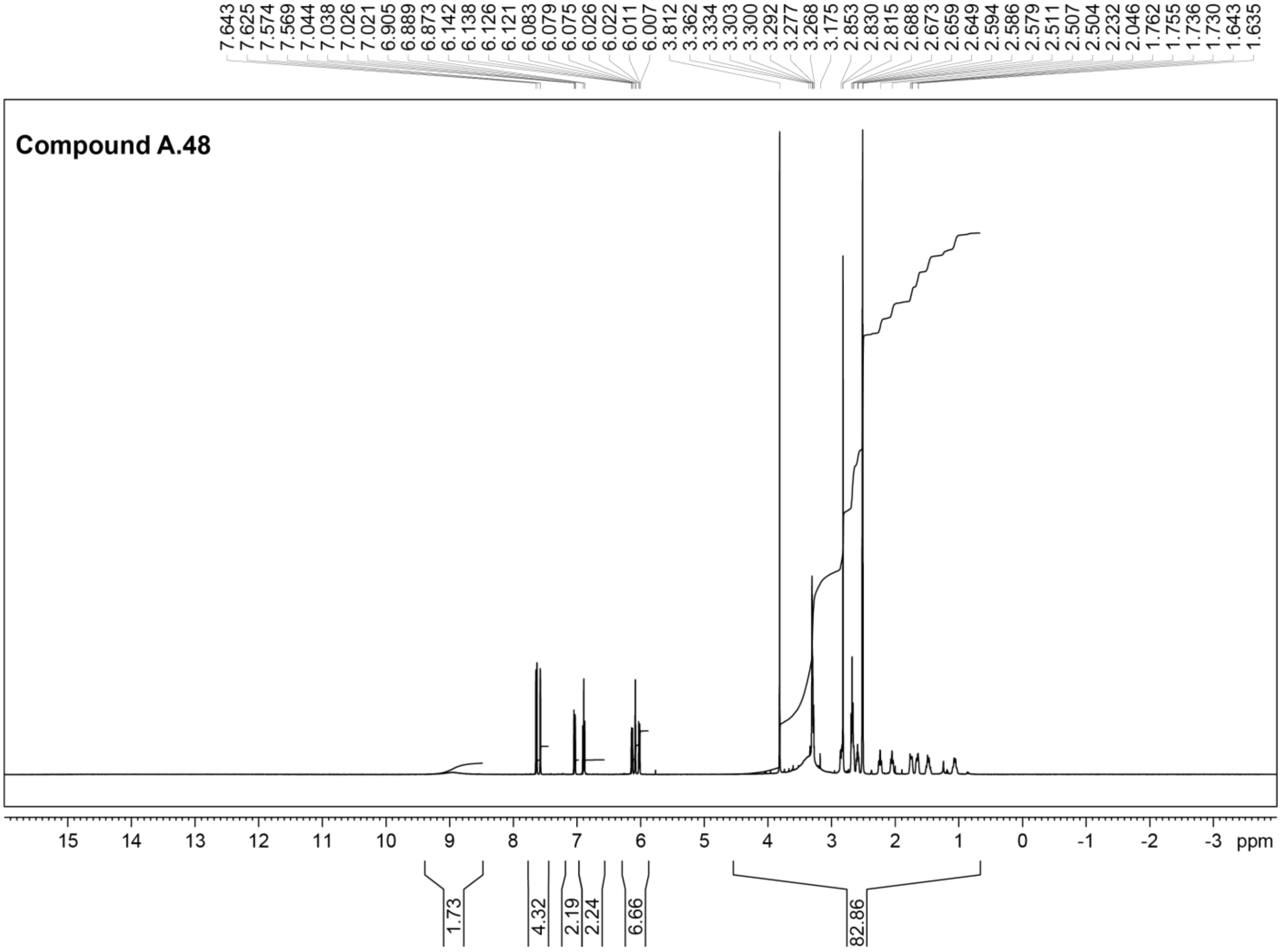

Synthesis Suppl. Figure 49. ^{1}H NMR of compound A.48.

(*S*)-2-(3-((2-(Ethyl(3-hydroxyphenyl)amino)ethyl)amino)piperidin-1-yl)-*N*-(6-methoxybenzo[*d*]thiazol-2-yl)acetamide (A.49)

Phenol **A.49** was prepared following General procedure N from nosyl derivative **47** (140 mg, 0.210 mmol, 1.0 eq). Off-white solid (86 mg, 0.18 mmol, 85%). Mp: 87 – 88 °C. ^{1}H NMR (500 MHz, DMSO-*d*$_6$) δ 7.63 (d, *J* = 8.7 Hz, 1H), 7.56 (d, *J* = 2.6 Hz, 1H), 7.03 (dd, *J* = 8.8, 2.6 Hz, 1H), 6.87 (t, *J* = 8.0 Hz, 1H), 6.09 (dd, *J* = 8.3, 2.4 Hz, 1H), 6.05 (t, *J* = 2.3 Hz, 1H), 5.97 (dd, *J* = 7.9, 2.1 Hz, 1H), 3.80 (s, 3H), 3.30 (d, *J* = 2.5 Hz, 2H), 3.28 – 3.18 (m, 5H), 2.84 (d, *J* = 10.7 Hz, 1H), 2.67 (t, *J* = 7.3 Hz, 3H), 2.59 (tt, *J* = 8.1, 3.4 Hz, 1H), 2.24 (t, *J* = 10.1 Hz, 1H), 2.05 (t, *J* = 9.4 Hz, 1H), 1.79 – 1.69 (m, 1H), 1.65 (dt, *J* = 13.6, 4.3 Hz, 1H), 1.47 (qd, *J* = 11.7, 6.7 Hz, 1H), 1.10 – 1.04 (m, 1H), 1.01 (t, *J* = 6.9 Hz, 3H). ^{13}C NMR (126 MHz, DMSO-*d*$_6$) δ 169.6, 158.3, 156.2, 155.5, 149.1, 142.6, 132.8, 129.7, 121.1, 114.9, 104.7, 102.9, 102.6, 98.5, 60.7, 59.1, 55.6, 53.8, 53.3, 50.5, 44.4, 44.1, 30.3, 23.3, 12.2. HRMS (ESI): *m/z* = calculated for $C_{25}H_{34}N_5O_3S$ [M+H]$^+$: 484.2377; found: 484.23768. Specific rotation: $[\alpha]_D^{20}$ = + 5.0 (c = 0.18). Purity (HPLC): > 98% (λ = 210 nm), > 98% (λ = 254 nm), Method 1c.

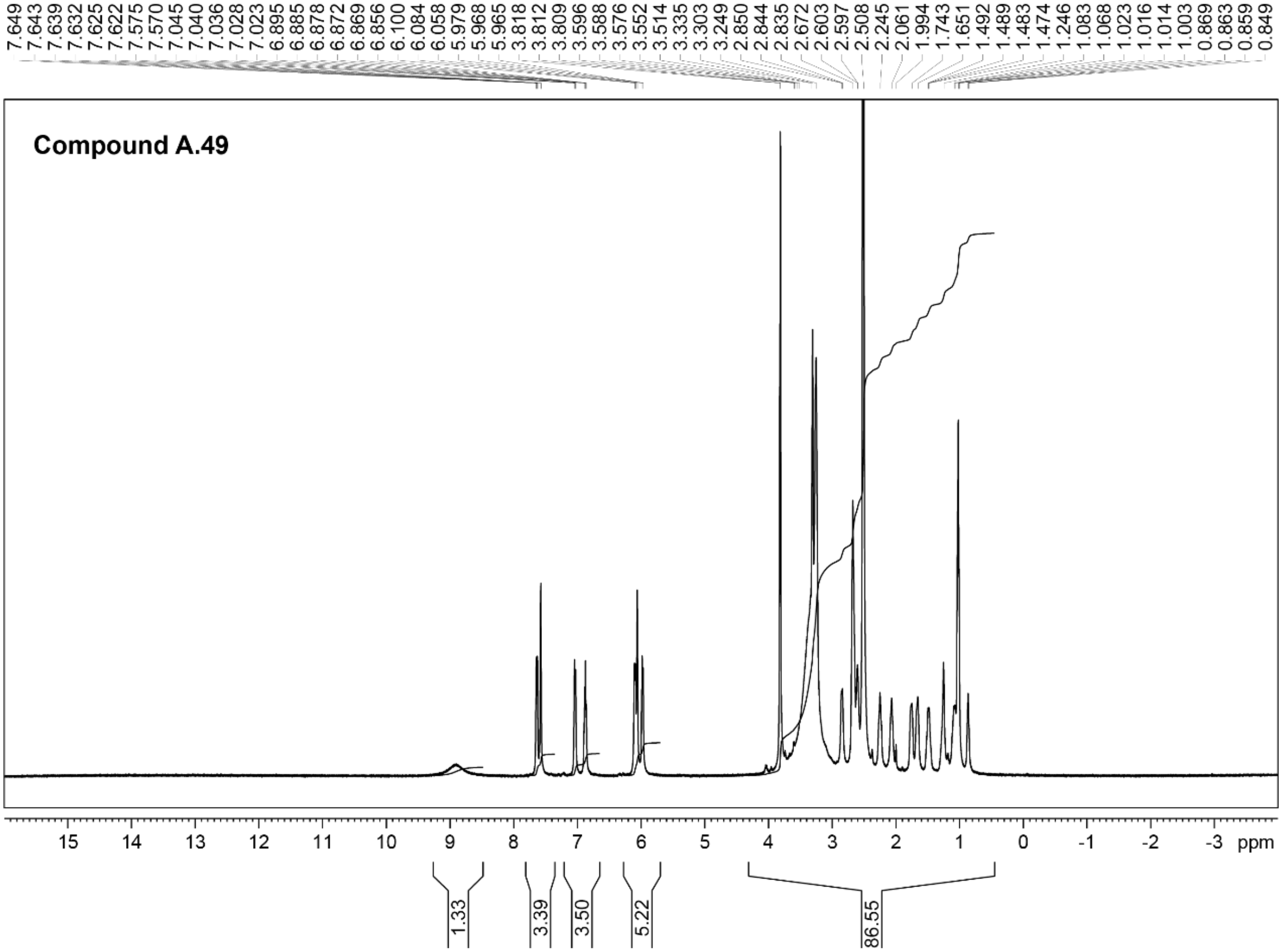

Synthesis Suppl. Figure 50. ^{1}H NMR of compound A.49.

(*R*)-2-(3-((2-((3-Hydroxyphenyl)(methyl)amino)ethyl)amino)piperidin-1-yl)-*N*-(6-methoxybenzo[*d*]thiazol-2-yl)acetamide (A.50)

Phenol **A.50** was prepared following General procedure N from nosyl derivative **48** (157 mg, 0.240 mmol, 1.0 eq). Off-white solid (95 mg, 0.20 mmol, 84%). Mp: 85 – 87 °C. ^{1}H NMR (400 MHz, DMSO-d_6) δ 7.63 (d, *J* = 8.8 Hz, 1H), 7.56 (d, *J* = 2.6 Hz, 1H), 7.03 (dd, *J* = 8.8, 2.6 Hz, 1H), 6.88 (t, *J* = 8.1 Hz, 1H), 6.12 (ddd, *J* = 8.4, 2.5, 0.8 Hz, 1H), 6.07 (t, *J* = 2.3 Hz, 1H), 6.01 (ddd, *J* = 8.0, 2.2, 0.8 Hz, 1H), 3.80 (s, 3H), 3.32 – 3.25 (m, 4H), 2.85 (s, 1H), 2.81 (s, 3H), 2.67 (t, *J* = 7.1 Hz, 3H), 2.58 (p, *J* = 4.7 Hz, 1H), 2.23 (t, *J* = 9.7 Hz, 1H), 2.04 (t, *J* = 9.5 Hz, 1H), 1.79 – 1.68 (m, 1H), 1.69 – 1.56 (m, 1H), 1.47 (dt, *J* = 13.5, 10.0 Hz, 1H), 1.05 (q, *J* = 10.3 Hz, 1H). ^{13}C NMR (101 MHz, DMSO-d_6) δ 169.6, 158.2, 156.1, 155.5, 150.6, 142.6, 132.8, 129.5, 121.1, 114.9, 104.7, 103.2, 103.1, 98.9, 60.7, 59.1, 55.6, 53.8, 53.3, 52.6, 43.3, 38.2, 30.3, 23.3. HRMS (ESI): *m/z* = calculated for $C_{24}H_{32}N_5O_3S$ $[M+H]^+$: 470.2221; found: 470.2214. Specific rotation: $[\alpha]_D^{20}$ = - 7.4 (c = 0.26). Purity (HPLC): > 98% (λ = 210 nm), > 98% (λ = 254 nm), Method 1c.

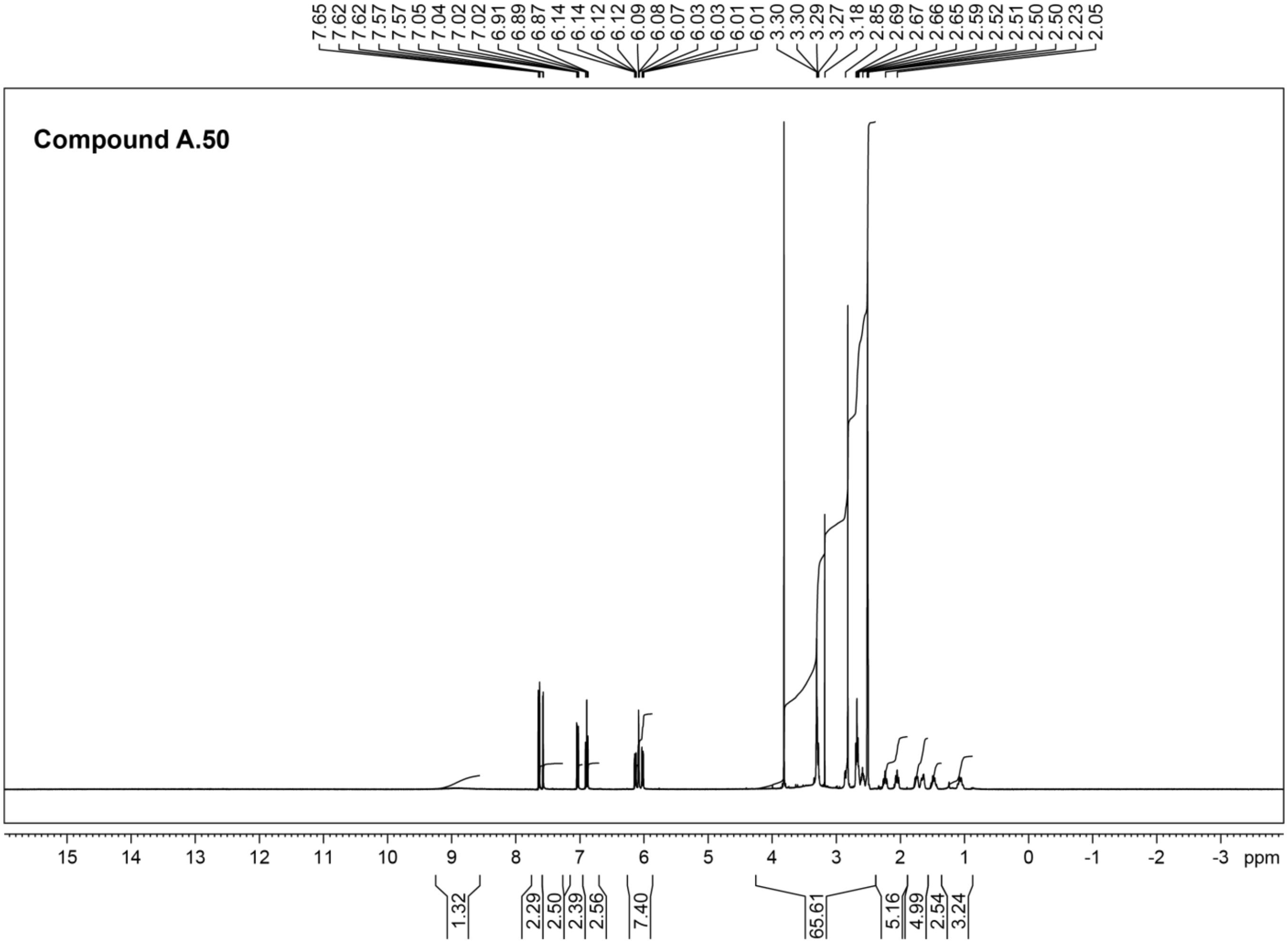

Synthesis Suppl. Figure 51. ^{1}H NMR of compound A.50.

(*R*)-2-(3-((2-(Ethyl(3-hydroxyphenyl)amino)ethyl)amino)piperidin-1-yl)-*N*-(6-methoxybenzo[*d*]thiazol-2-yl)acetamide (A.51)

Phenol **A.51** was prepared following General procedure N from nosyl derivative **49** (201 mg, 0.300 mmol, 1.0 eq). Off-white solid (88 mg, 0.27 mmol, 84%). Mp: 72 – 74 °C. ^{1}H NMR (400 MHz, DMSO-d_6) δ 7.63 (d, *J* = 8.8 Hz, 1H), 7.56 (d, *J* = 2.6 Hz, 1H), 7.03 (dd, *J* = 8.8, 2.6 Hz, 1H), 6.87 (t, *J* = 8.1 Hz, 1H), 6.09 (ddd, *J* = 8.4, 2.5, 0.8 Hz, 1H), 6.05 (t, *J* = 2.3 Hz, 1H), 5.97 (ddd, *J* = 7.9, 2.1, 0.8 Hz, 1H), 3.80 (s, 3H), 3.30 (d, *J* = 1.6 Hz, 2H), 3.28 – 3.19 (m, 4H), 2.84 (d, *J* = 10.7 Hz, 1H), 2.71 – 2.62 (m, 3H), 2.59 (dq, *J* = 8.2, 4.2 Hz, 1H), 2.24 (t, *J* = 9.9 Hz, 1H), 2.05 (t, *J* = 9.5 Hz, 1H), 1.82 – 1.70 (m, 1H), 1.65 (dd, *J* = 9.1, 4.6 Hz, 1H), 1.47 (dd, *J* = 10.0, 3.8 Hz, 1H), 1.08 (t, *J* = 10.8 Hz, 1H), 1.01 (t, *J* = 6.9 Hz, 3H). ^{13}C NMR (101 MHz, DMSO-d_6) δ 169.6, 158.3, 156.1, 155.5, 149.1, 142.6, 132.8, 129.7, 121.1, 114.9, 104.7, 102.9, 102.6, 98.5, 60.7, 59.1, 55.6, 53.8, 53.3, 50.5, 44.4, 44.1, 30.3, 23.3, 12.1. HRMS (ESI): *m/z* = calculated for $C_{25}H_{34}N_5O_3S$ $[M+H]^+$: 484.2377; found: 484.2367. Specific rotation: $[\alpha]_D^{20}$ = - 4.7 (c = 0.26). Purity (HPLC): > 98% (λ = 210 nm), > 99% (λ = 254 nm), Method 1c.

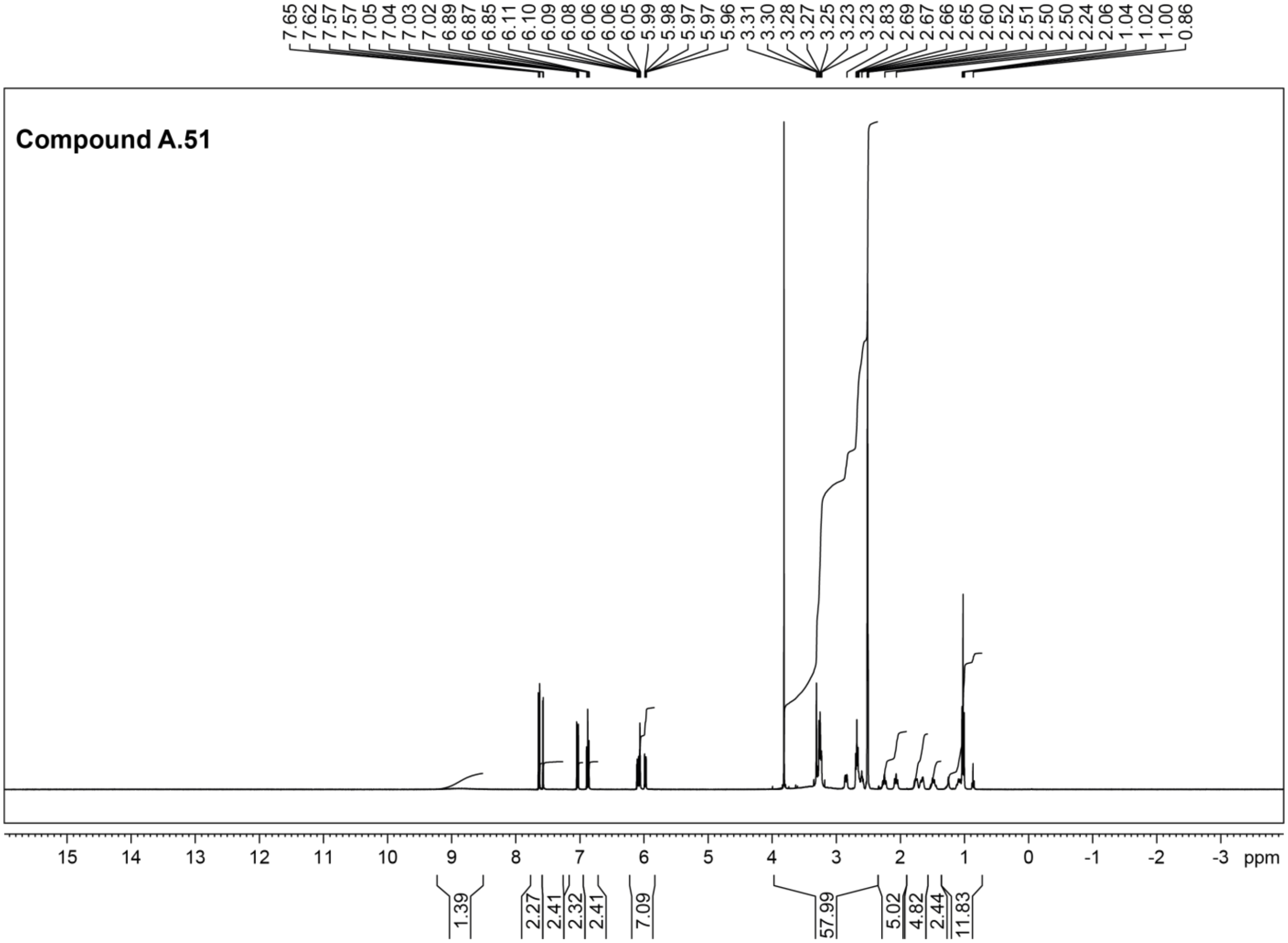

Synthesis Suppl. Figure 52. ^{1}H NMR of compound A.51.

(*S*)-2-(3-((3-Hydroxybenzyl)amino)piperidin-1-yl)-*N*-(6-methoxybenzo[*d*]thiazol-2-yl)acetamide (A.52)

N-Substituted 3-aminopiperidine **A.52** was prepared following General procedure I from 3-aminopiperidine **A.36**. Colourless solid (127 mg, 0.298 mmol, 60%). Mp: 68 – 70 °C. ^{1}H NMR (500 MHz, DMSO-d_6) δ 9.22 (s, 1H), 7.63 (d, *J* = 8.9 Hz, 1H), 7.57 (d, *J* = 2.6 Hz, 1H), 7.07 – 7.00 (m, 2H), 6.76 (t, *J* = 2.0 Hz, 1H), 6.72 (dt, *J* = 7.4, 1.2 Hz, 1H), 6.58 (ddd, *J* = 8.1, 2.6, 1.1 Hz, 1H), 4.05 (s, 1H), 3.80 (s, 3H), 3.64 (d, *J* = 2.1 Hz, 2H), 2.88 – 2.82 (m, 1H), 2.62 (dd, *J* = 15.3, 11.1 Hz, 1H), 2.56 (dq, *J* = 8.3, 4.2 Hz, 1H), 2.23 (t, *J* = 10.1 Hz, 1H), 2.10 (t, *J* = 9.3 Hz, 1H), 1.76 (dd, *J* = 13.0, 5.1 Hz, 1H), 1.65 (dt, *J* = 13.2, 4.4 Hz, 1H), 1.44 (qt, *J* = 10.0, 3.7 Hz, 1H), 1.15 – 1.02 (m, 1H). ^{13}C NMR (126 MHz, DMSO-d_6) δ 169.6, 157.2, 156.1, 155.5, 142.9, 142.6, 132.8, 129.0, 121.1, 118.5, 114.9, 114.7, 113.3, 104.7, 60.8, 59.0, 55.6, 53.4, 52.8, 49.9, 30.0, 23.3. HRMS (EI): m/z = calculated for $C_{22}H_{26}N_4O_3S$ $[M]^{\cdot+}$: 426.1712; found: 426.1714. Specific rotation: $[\alpha]_D^{20}$ = + 7.2 (c = 0.25). Purity (HPLC): 100% (λ = 210 nm), 100% (λ = 254 nm), Method 1a.

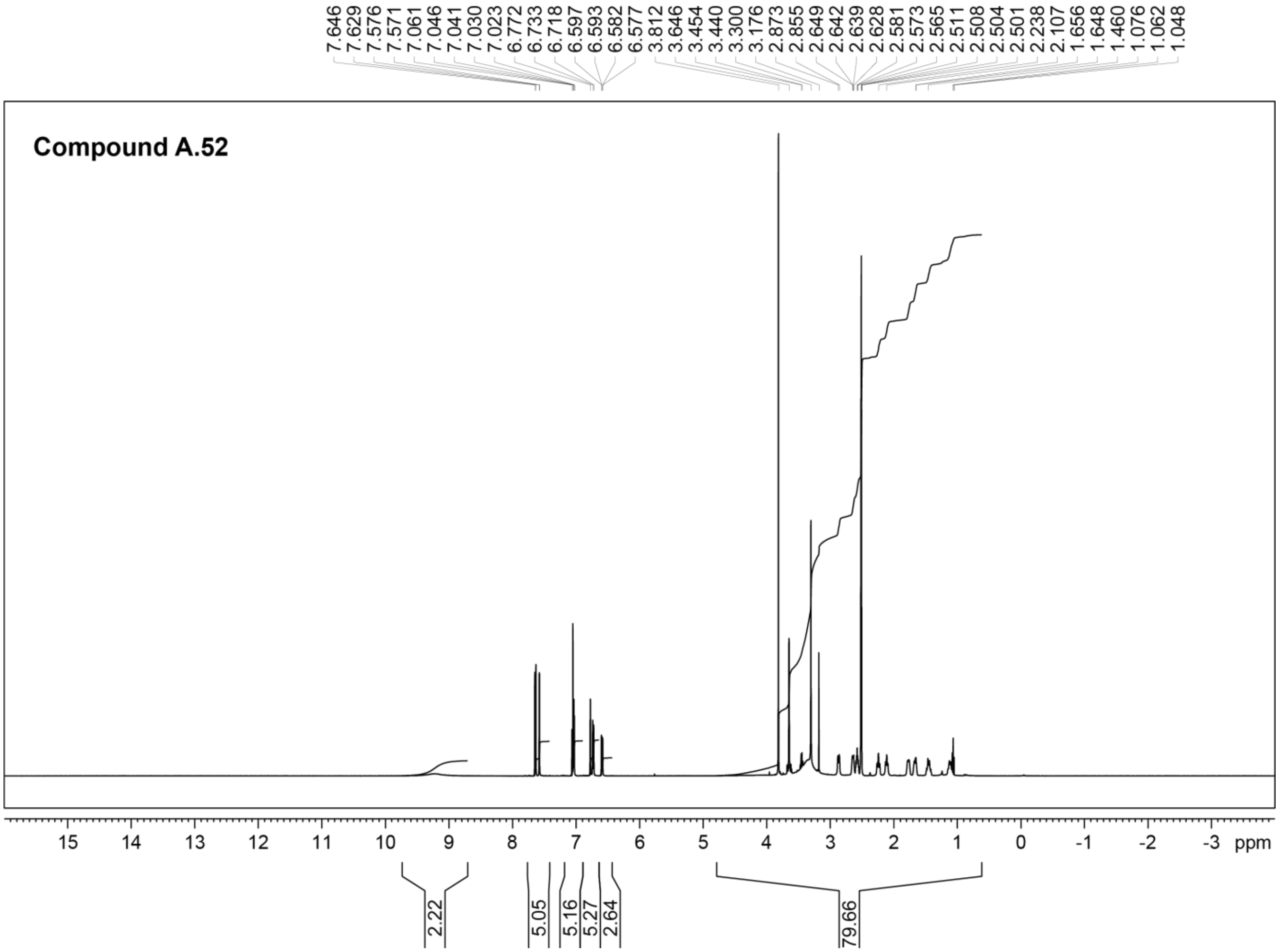

Synthesis Suppl. Figure 53. ^{1}H NMR of compound A.52.

(*R*)-2-(3-((3-hydroxybenzyl)amino)piperidin-1-yl)-*N*-(6-methoxybenzo[*d*]thiazol-2-yl)acetamide (A.53)

N-Substituted 3-aminopiperidine **A.53** was prepared following General procedure I from 3-aminopiperidine **A.37**. Colourless solid (149 mg, 0.349 mmol, 70%). Mp: 69 – 72 °C. ^{1}H NMR (500 MHz, DMSO-d_6) δ 9.22 (s, 1H), 7.63 (d, *J* = 8.8 Hz, 1H), 7.57 (d, *J* = 2.6 Hz, 1H), 7.07 – 7.00 (m, 2H), 6.76 (t, *J* = 2.1 Hz, 1H), 6.72 (dt, *J* = 7.5, 1.3 Hz, 1H), 6.58 (ddd, *J* = 8.1, 2.5, 1.0 Hz, 1H), 4.02 (s, 1H), 3.80 (s, 3H), 3.64 (d, *J* = 2.0 Hz, 2H), 3.44 (s, 1H), 2.89 – 2.82 (m, 1H), 2.63 (dd, *J* = 9.4, 5.8 Hz, 1H), 2.56 (tt, *J* = 8.1, 3.6 Hz, 1H), 2.27 – 2.19 (m, 1H), 2.10 (t, *J* = 9.4 Hz, 1H), 1.79 – 1.72 (m, 1H), 1.65 (dt, *J* = 13.1, 4.3 Hz, 1H), 1.44 (tdd, *J* = 13.8, 7.8, 3.8 Hz, 1H), 1.15 – 1.06 (m, 1H). ^{13}C NMR (126 MHz, DMSO-d_6) δ 169.5, 157.2, 156.1, 155.5, 142.9, 142.6, 132.8, 129.0, 121.1, 118.5, 114.9, 114.7, 113.3, 104.7, 60.8, 59.0, 55.6, 53.34, 52.8, 49.9, 30.0, 23.3. HRMS (EI): m/z = calculated for $C_{22}H_{26}N_4O_3S$ [M]$^{\cdot+}$: 426.1712; found: 426.1708. Specific rotation: $[\alpha]_D^{20}$ = - 8.7 (c = 0.23). Purity (HPLC): 100% (λ = 210 nm), 100% (λ = 254 nm), Method 1a.

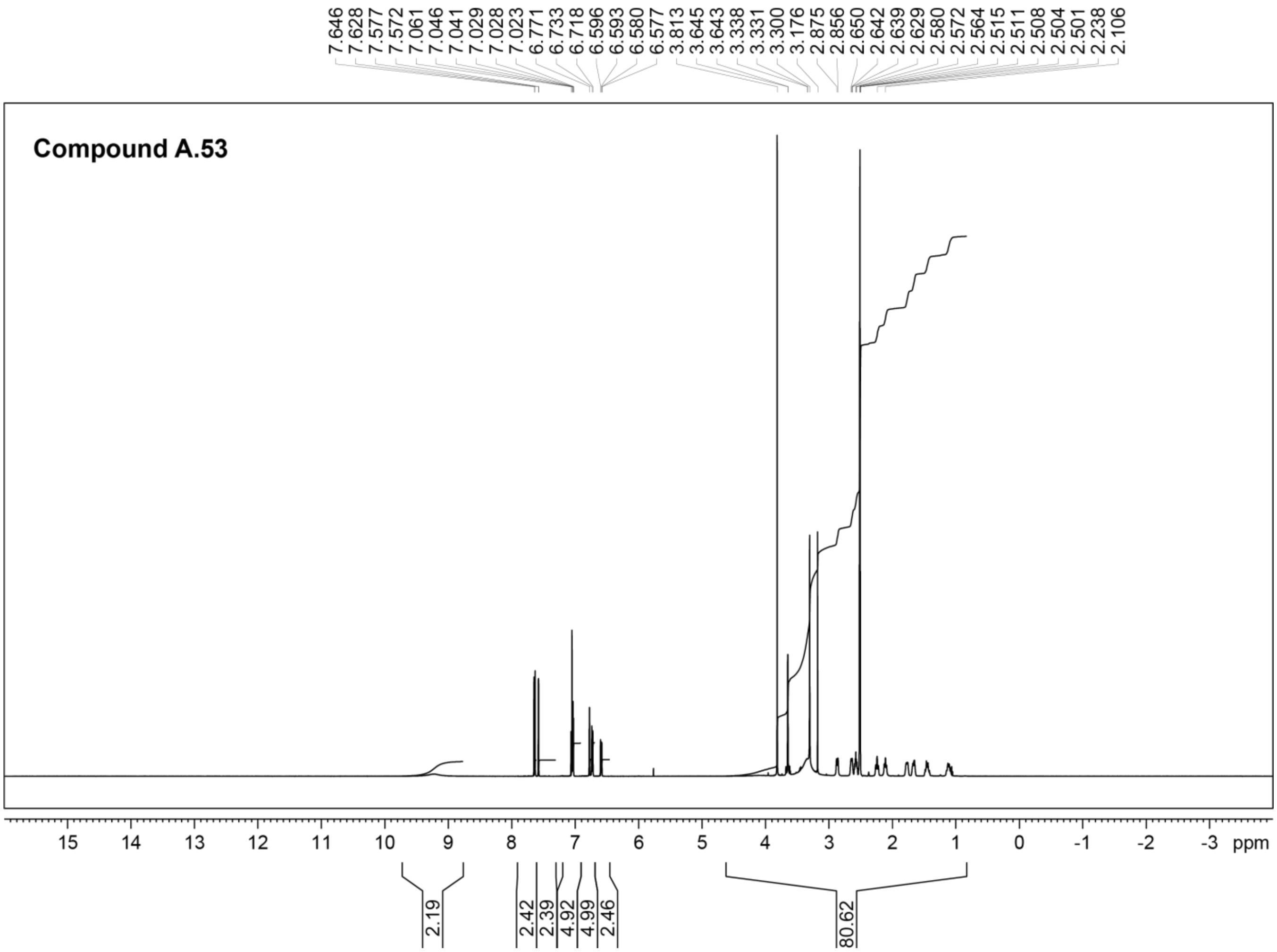

Synthesis Suppl. Figure 54. ^{1}H NMR of compound A.53.

(*S*)-2-(3-Aminopiperidin-1-yl)-*N*-(4,6-dimethoxybenzo[*d*]thiazol-2-yl)acetamide (A.54)

3-Aminopiperidine **A.54** was prepared following General procedure H from Boc-derivative **50** (162 mg, 0.360 mmol, 1.0 eq). The mixture was extracted with $CHCl_3$/isopropanol (3:1, 5 x 10 mL) and the residue purified by flash column chromatography (94:5:1 DCM/MeOH/triethylamine), yielding product **A.54** (125 mg, 0.357 mmol, 99%) as a colourless solid. Mp: 166 – 168 °C. ^{1}H NMR (400 MHz, DMSO-d_6) δ 7.10 (d, *J* = 2.2 Hz, 1H), 6.58 (d, *J* = 2.3 Hz, 1H), 4.43 (s, 2H), 3.88 (s, 3H), 3.80 (s, 3H), 3.32 – 3.18 (m, 2H), 2.79 – 2.67 (m, 2H), 2.67 – 2.59 (m, 1H), 2.16 (s br, 1H), 1.93 (t, *J* = 9.4 Hz, 1H), 1.73 – 1.58 (m, 2H), 1.47 (s br, 1H), 1.03 – 0.93 (m, 1H). ^{13}C NMR (101 MHz, DMSO-d_6) δ 169.4, 157.2, 154.1, 152.1, 133.5, 132.8, 98.0, 95.7, 62.1, 60.8, 55.8, 55.6, 53.1, 47.7, 33.1, 23.41. HRMS (ESI): *m/z* = calculated for $C_{16}H_{21}N_4O_2S$ [M-H]$^-$: 349.1339; found: 349.1337. Specific rotation: $[\alpha]_D^{20}$ = + 13.7 (c = 0.22). Purity (HPLC): > 99% (λ = 210 nm), 100% (λ = 254 nm), Method 1a.

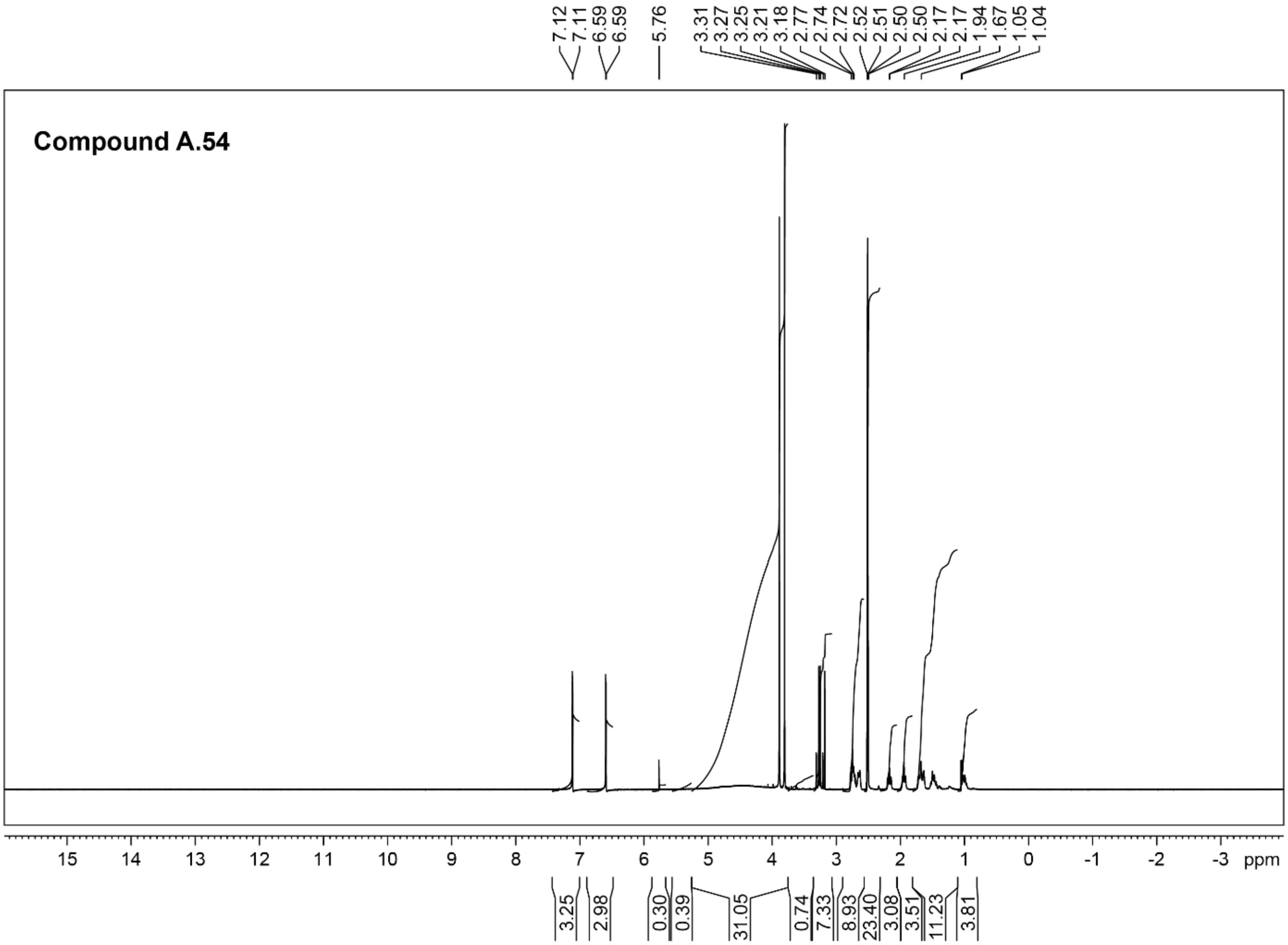

Synthesis Suppl. Figure 55. ^{1}H NMR of compound A.54.

(*R*)-2-(3-aminopiperidin-1-yl)-*N*-(4,6-dimethoxybenzo[*d*]thiazol-2-yl)acetamide (A.55)

3-Aminopiperidine **A.55** was prepared following General procedure H from Boc-derivative **51** (113 mg, 0.250 mmol, 1.0 eq). The mixture was extracted with $CHCl_3$/isopropanol (3:1, 5 x 10 mL) and the residue purified by flash column chromatography (94:5:1 DCM/MeOH/triethylamine), yielding product **A.55** (97 mg, 0.24 mmol, 99%) as a colourless solid. Mp: 169 – 170 °C. ^{1}H NMR (400 MHz, DMSO-d_6) δ 7.10 (d, *J* = 2.2 Hz, 1H), 6.58 (d, *J* = 2.3 Hz, 1H), 4.60 (s, 5H), 3.88 (s, 3H), 3.80 (s, 3H), 3.33 – 3.18 (m, 2H), 2.78 – 2.67 (m, 2H), 2.67 – 2.60 (m, 1H), 2.16 (t, *J* = 10.1 Hz, 1H), 1.93 (t, *J* = 9.2 Hz, 1H), 1.73 – 1.59 (m, 2H), 1.54 – 1.41 (m, 1H), 1.03 – 0.91 (m, 1H). ^{13}C NMR (101 MHz, DMSO-d_6) δ 169.5, 157.2, 154.1, 152.1, 133.5, 132.8, 98.0, 95.7, 62.2, 60.9, 55.8, 55.6, 53.1, 47.7, 33.1, 23.4. HRMS (ESI): *m/z* = calculated for $C_{16}H_{21}N_4O_2S$ [M-H]$^-$: 349.1339; found: 349.1338. Specific rotation: $[\alpha]_D^{20}$ = - 15.0 (c = 0.21). Purity (HPLC): > 99% (λ = 210 nm), > 99% (λ = 254 nm), Method 1a.

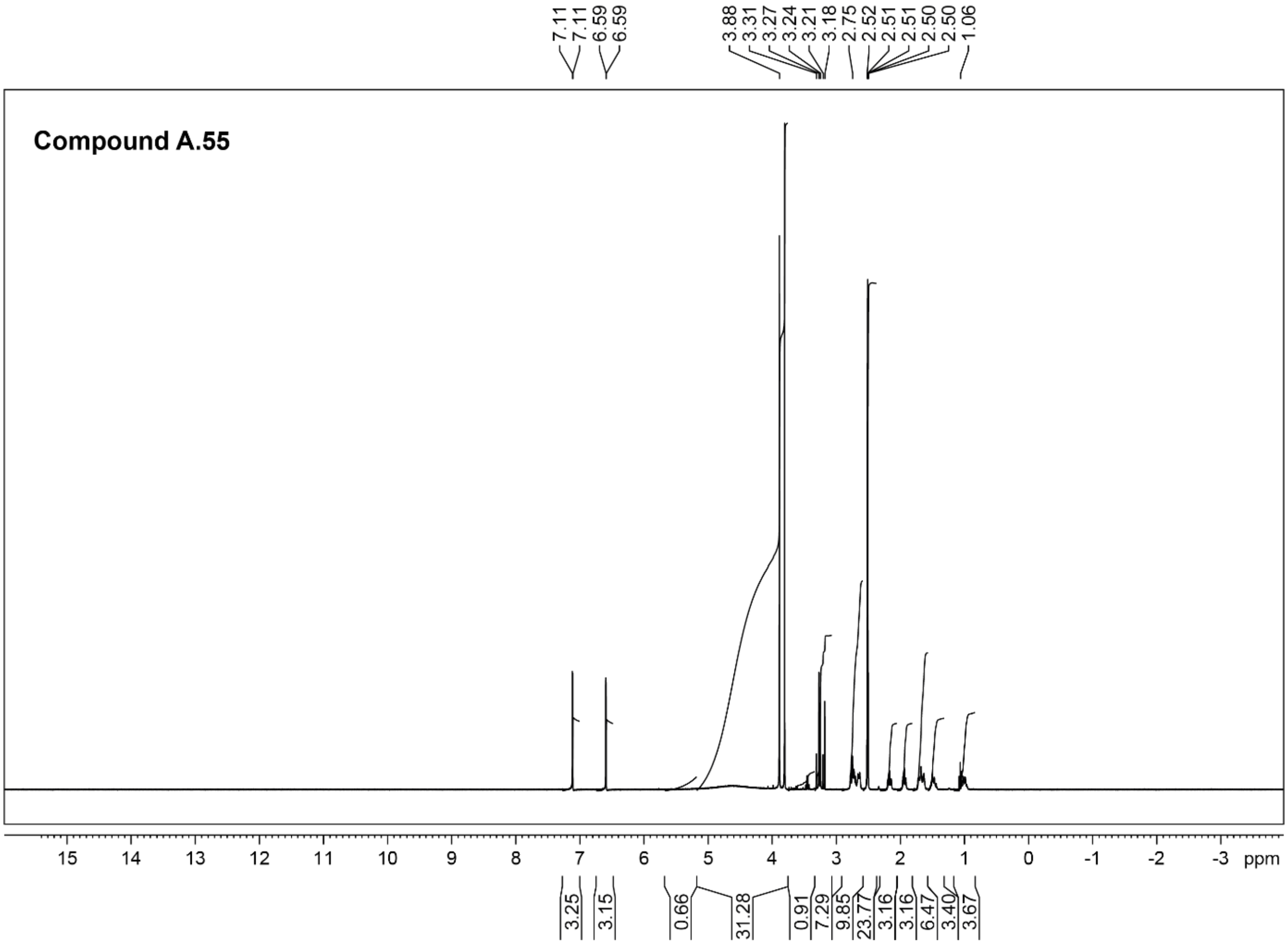

Synthesis Suppl. Figure 56. ^{1}H NMR of compound A.55.

_N_-(4,6-Dimethoxybenzo[_d_]thiazol-2-yl)-2-(4-methylpiperazin-1-yl)acetamide (A.56)

Amine **A.56** was prepared following General procedure F, using chloroacetamide **6** (143 mg, 0.500 mmol, 1.0 eq) and 1-methylpiperazine (0.122 mL, 1.10 mmol, 2.2 eq). The mixture was stirred for 2 h and the extraction was conducted with EtOAc. The residue was purified by flash column chromatography (93:6:1 DCM/MeOH/triethylamine), yielding product **A.56** (160 mg, 0.457 mmol, 91%) as a colourless solid. Mp: 162 – 164 °C. ^{1}H NMR (400 MHz, DMSO-d_6) δ 7.11 (d, J = 2.3 Hz, 1H), 6.59 (d, J = 2.2 Hz, 1H), 3.88 (s, 3H), 3.80 (s, 3H), 3.28 (s, 2H), 2.53 (s, 3H), 2.34 (s, 4H), 2.16 (s, 3H). ^{13}C NMR (101 MHz, DMSO-d_6) δ 168.9, 157.3, 153.7, 152.2, 133.5, 132.7, 98.0, 95.7, 60.2, 55.8, 55.7, 54.6, 52.5, 45.8. HRMS (ESI): m/z = calculated for $C_{16}H_{21}N_4O_3S$ $[M-H]^-$: 349.1339; found: 349.1335. Purity (HPLC): 100% (λ = 210 nm), 100% (λ = 254 nm), Method 1a.

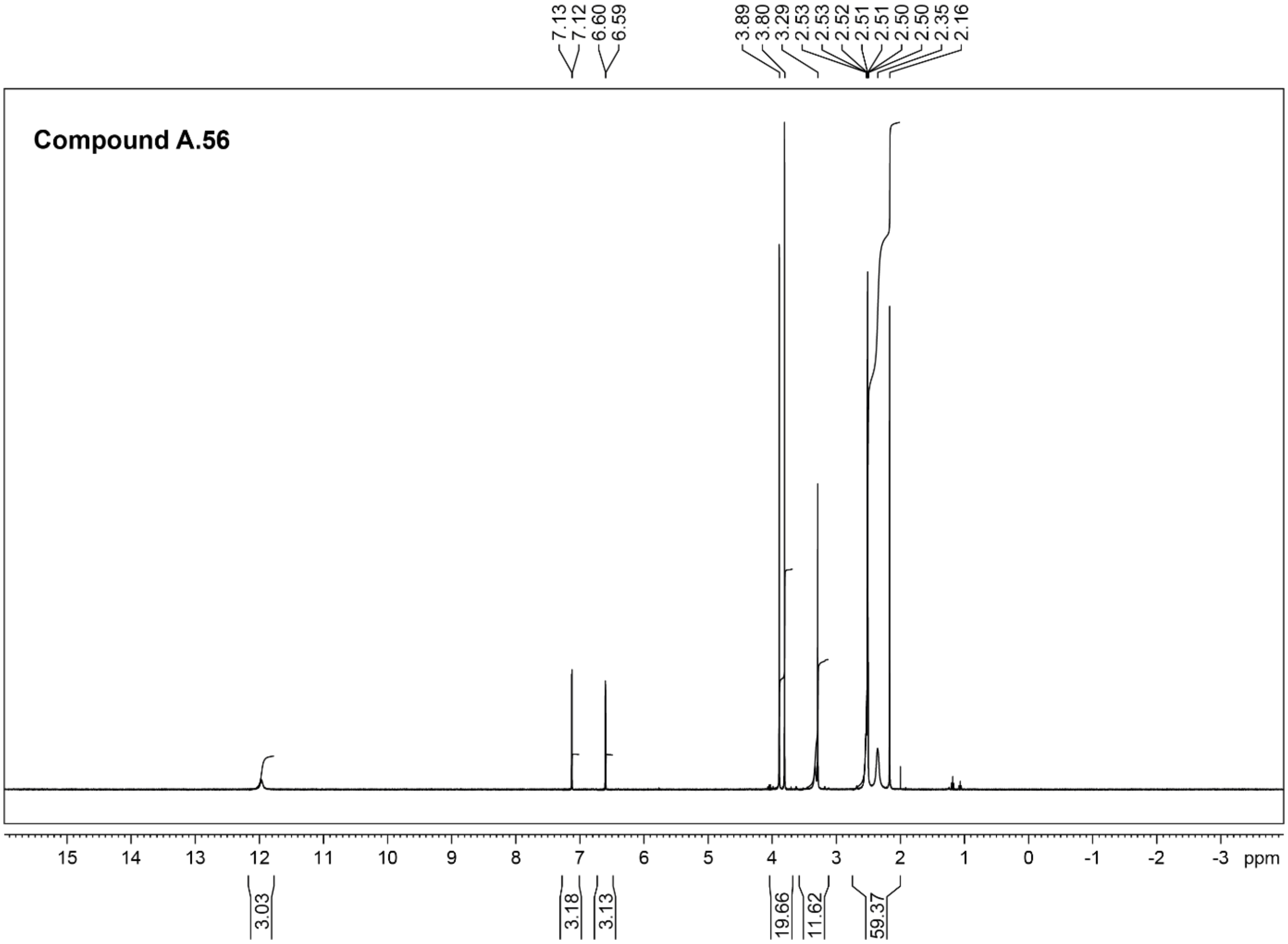

Synthesis Suppl. Figure 57. ^{1}H NMR of compound A.56.

N-(4,6-Dimethoxybenzo[d]thiazol-2-yl)-2-(piperazin-1-yl)acetamide (A.57)

Amine **A.57** was prepared following General procedure F, using chloroacetamide **6** (143 mg, 0.500 mmol, 1.0 eq), piperazine (48 mg, 0.55 mmol, 1.1 eq) and triethylamine (77 µL, 0.55 mmol, 1.1 eq). The mixture was stirred for 2 h and the extraction was conducted with EtOAc. The residue was purified by flash column chromatography (93:6:1 DCM/MeOH/triethylamine), yielding product **A.57** (75 mg, 0.22 mmol, 44%) as a colourless solid. Mp: 185 – 187 °C. ^{1}H NMR (500 MHz, DMSO-d_6) δ 7.11 (d, J = 2.3 Hz, 1H), 6.59 (d, J = 2.3 Hz, 1H), 3.88 (s, 2H), 3.80 (s, 2H), 3.25 (s, 1H), 2.73 (t, J = 4.8 Hz, 3H), 2.45 (t, J = 4.8 Hz, 3H). ^{13}C NMR (126 MHz, DMSO-d_6) δ 169.0, 157.3, 153.8, 152.2, 133.5, 132.7, 98.0, 95.7, 61.0, 55.8, 55.7, 53.7, 45.4. HRMS (EI): m/z = calculated for $C_{15}H_{20}N_4O_3S$ $[M]^{\cdot+}$: 336.1251; found: 336.1254. Purity (HPLC): 100% (λ = 210 nm), 100% (λ = 254 nm), Method 1a.

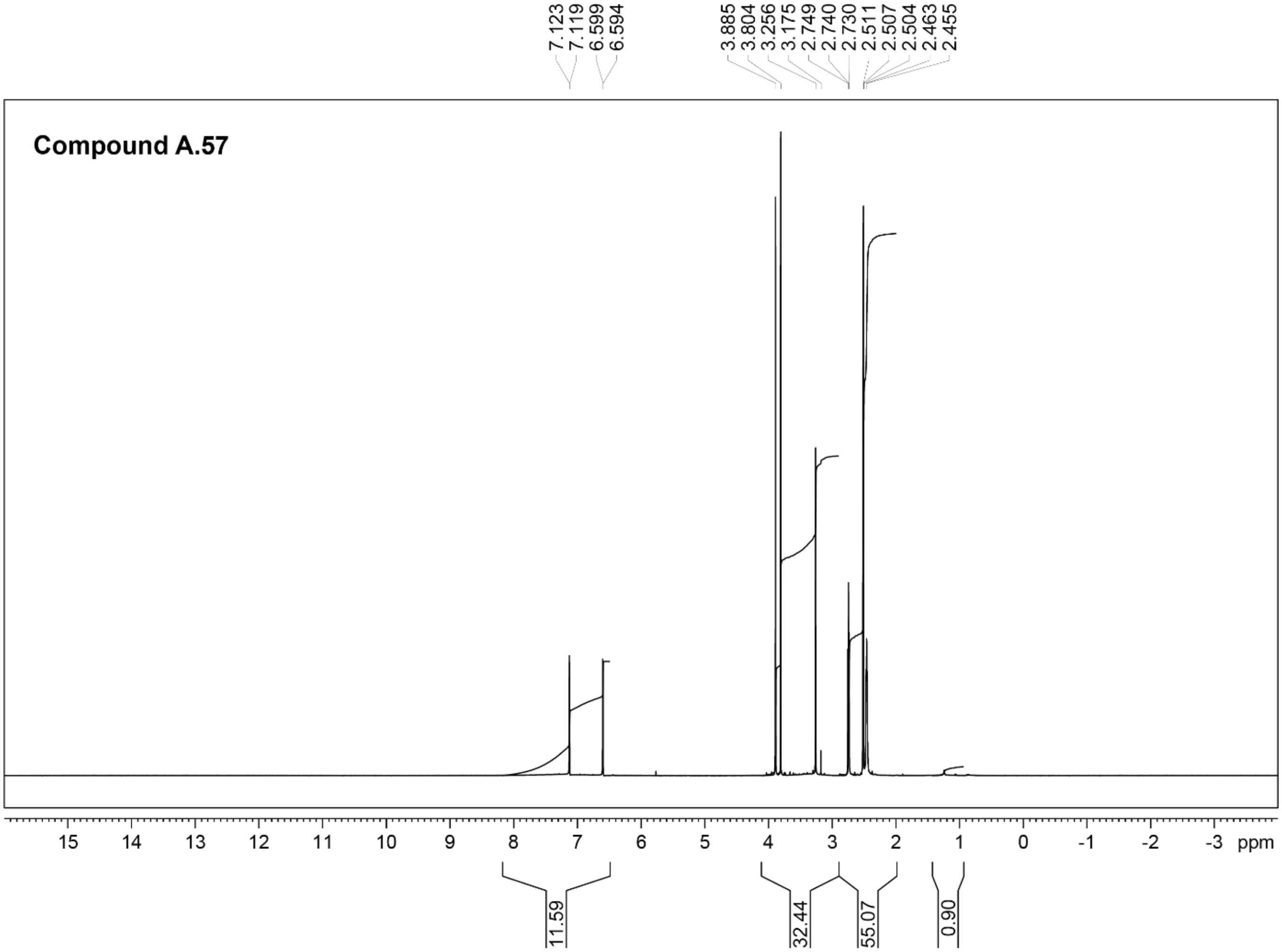

Synthesis Suppl. Figure 58. ^{1}H NMR of compound A.57.

(*S*)-2-(3-aminopiperidin-1-yl)-*N*-(5-(3,4-dimethoxyphenyl)-1,3,4-thiadiazol-2-yl)acetamide (A.58)

3-Aminopiperidine **A.58** was prepared following General procedure H from Boc-derivative **18** (250 mg, 0.523 mmol, 1.0 eq). The mixture was extracted with DCM (3:1, 5 x 10 mL) and the residue purified by flash column chromatography (84:15:1 DCM/MeOH/triethylamine), yielding product **A.58** (122 mg, 0.323 mmol, 62%) as a light yellow solid. Mp: 86 – 88 °C. ^{1}H NMR (500 MHz, DMSO-d_6) δ 7.48 (d, *J* = 2.0 Hz, 1H), 7.39 (dd, *J* = 8.4, 2.0 Hz, 1H), 7.06 (d, *J* = 8.4 Hz, 1H), 4.81 (s, 7H), 3.85 (s, 3H), 3.81 (s, 3H), 3.33 – 3.22 (m, 2H), 2.90 (tt, *J* = 8.1, 3.5 Hz, 1H), 2.77 (dd, *J* = 10.8, 3.4 Hz, 1H), 2.64 – 2.58 (m, 1H), 2.27 (t, *J* = 10.1 Hz, 1H), 2.13 (t, *J* = 9.5 Hz, 1H), 1.74 – 1.63 (m, 2H), 1.52 – 1.42 (m, 1H), 1.16 (d, *J* = 10.0 Hz, 1H). ^{13}C NMR (126 MHz, DMSO-d_6) δ 170.7, 160.9, 160.3, 150.4, 149.0, 123.8, 120.0, 112.0, 109.1, 61.68, 59.9, 55.6, 55.5, 52.9, 47.3, 31.2, 22.8. HRMS (ESI): *m/z* = calculated for $C_{17}H_{22}N_5O_3S$ $[M-H]^-$: 376.1446; found: 376.1446. Specific rotation: $[\alpha]_D^{20}$ = + 1.2 (c = 0.24). Purity (HPLC): > 95% (λ = 210 nm), > 96% (λ = 254 nm), Method 1a.

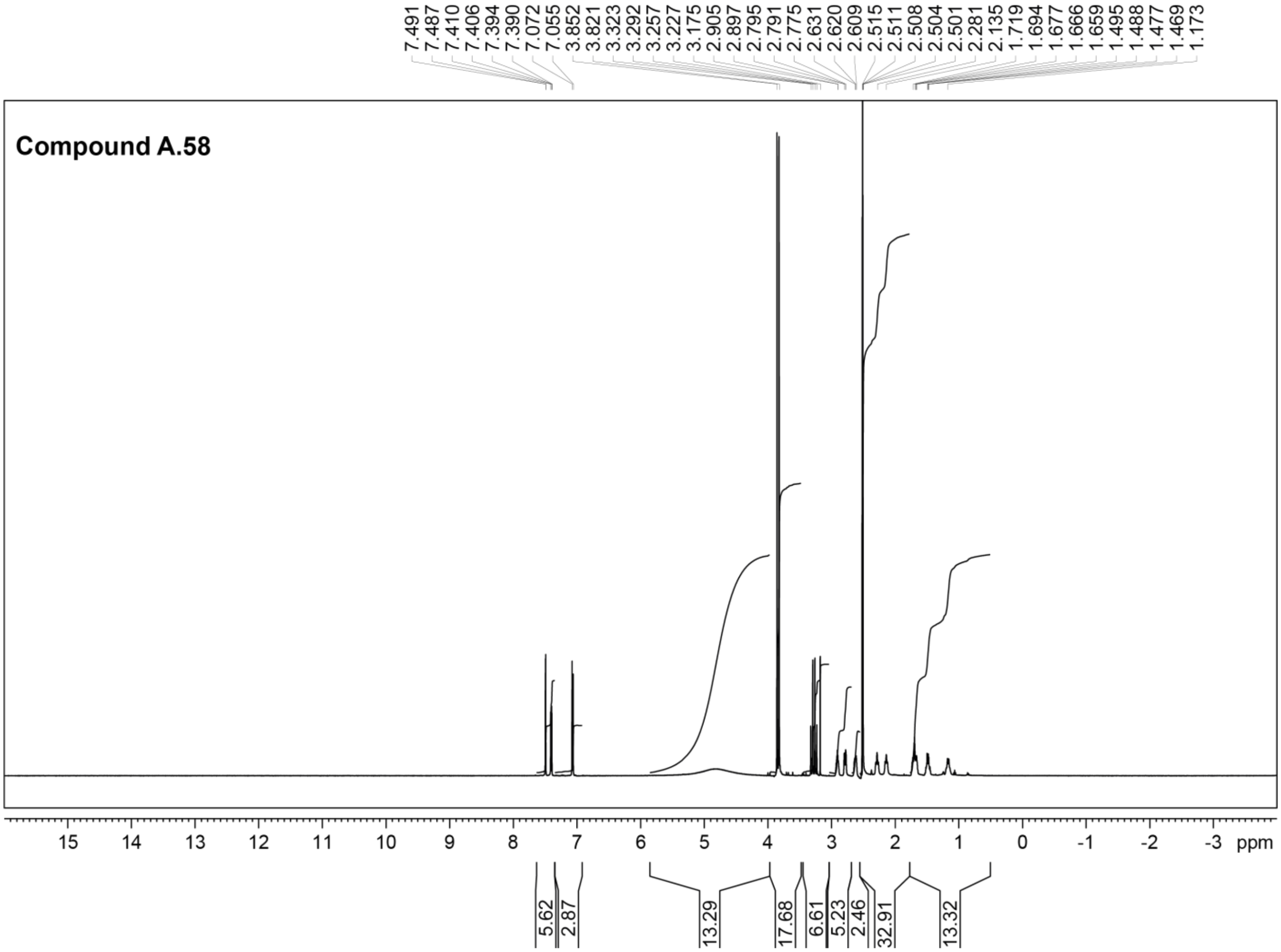

Synthesis Suppl. Figure 59. ^{1}H NMR of compound A.58.

(*R*)-2-(3-aminopiperidin-1-yl)-*N*-(5-(3,4-dimethoxyphenyl)-1,3,4-thiadiazol-2-yl)acetamide (A.59)

3-Aminopiperidine **A.59** was prepared following General procedure H from Boc-derivative **19** (250 mg, 0.523 mmol, 1.0 eq). The mixture was extracted with DCM (3:1, 5 x 10 mL) and the residue purified by flash column chromatography (84:15:1 DCM/MeOH/triethylamine), yielding product **A.59** (140 mg, 0.371 mmol, 71%) as a light yellow solid. Mp: 78 – 80 °C. ^{1}H NMR (500 MHz, DMSO-d_6) δ 7.48 (d, *J* = 2.0 Hz, 1H), 7.40 (dd, *J* = 8.3, 2.0 Hz, 1H), 7.06 (d, *J* = 8.4 Hz, 1H), 4.72 (s, 7H), 3.85 (s, 3H), 3.81 (s, 3H), 3.33 – 3.22 (m, 2H), 2.94 – 2.87 (m, 1H), 2.81 – 2.74 (m, 1H), 2.64 – 2.58 (m, 1H), 2.28 (t, *J* = 10.1 Hz, 1H), 2.14 (t, *J* = 9.5 Hz, 1H), 1.73 – 1.64 (m, 1H), 1.48 (qd, *J* = 9.3, 4.0 Hz, 1H), 1.17 (d, *J* = 9.4 Hz, 1H). ^{13}C NMR (126 MHz, DMSO-d_6) δ 170.6, 160.7, 160.4, 150.4, 149.1, 123.7, 120.0, 112.0, 109.1, 61.6, 59.79, 55.6, 55.5, 52.9, 47.3, 31.1, 22.8. HRMS (ESI): *m/z* = calculated for $C_{17}H_{22}N_5O_3S$ [M-H]$^-$: 376.1446; found: 376.1446. Specific rotation: $[\alpha]_D^{20}$ = - 1.1 (c = 0.19). Purity (HPLC): > 95% (λ = 210 nm), > 96% (λ = 254 nm), Method 1a.

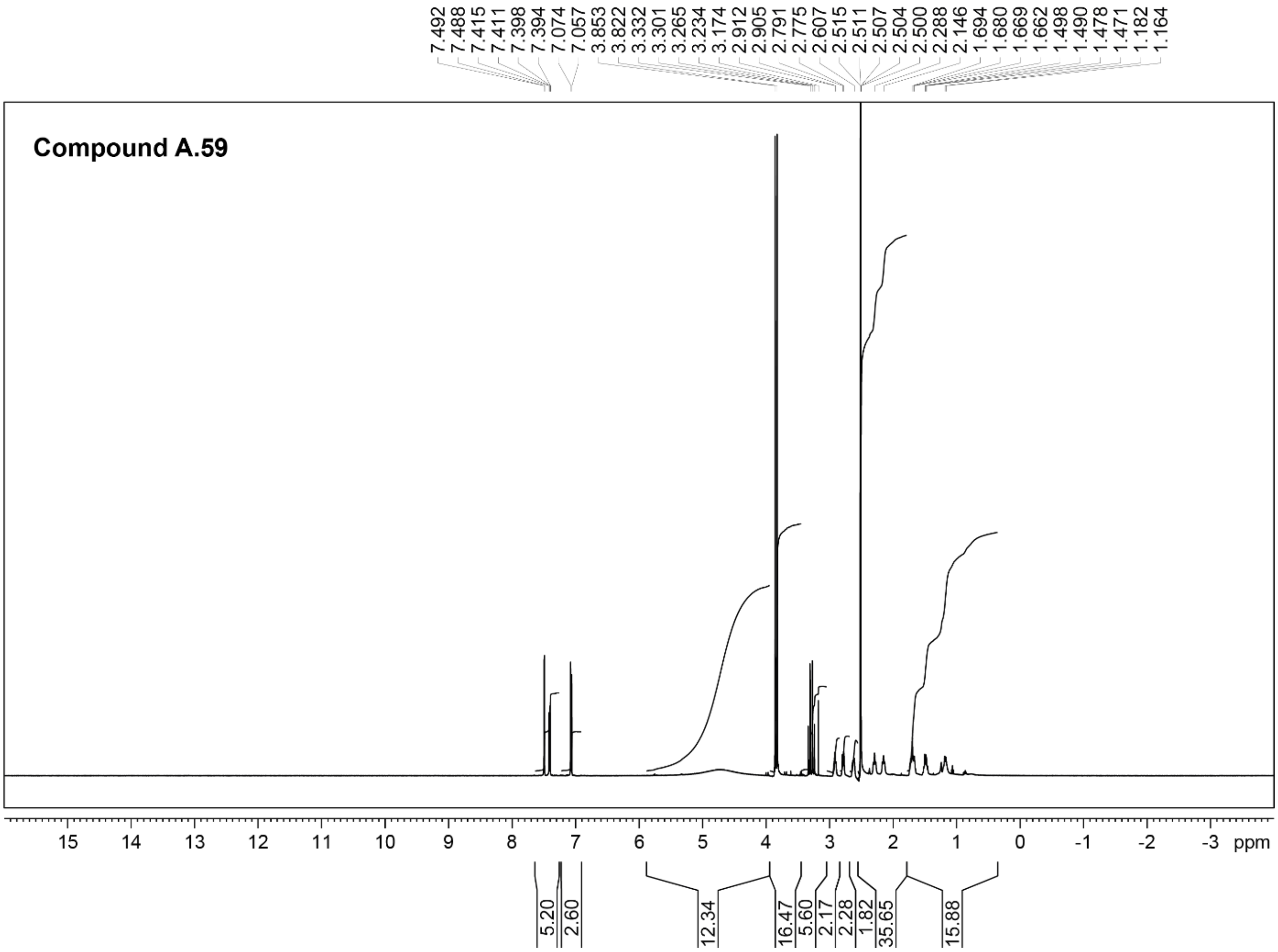

Synthesis Suppl. Figure 60. ^{1}H NMR of compound A.59.

REFERENCES

(1) Sağlık, B. N.; Ilgın, S.; Özkay, Y. Synthesis of New Donepezil Analogues and Investigation of Their Effects on Cholinesterase Enzymes. *Eur. J. Med. Chem.* **2016**, *124*, 1026–1040. https://doi.org/10.1016/j.ejmech.2016.10.042.

(2) Patel, R. V.; Kumari, P.; Rajani, D. P.; Chikhalia, K. H. Synthesis of Coumarin-Based 1,3,4-Oxadiazol-2ylthio-N-Phenyl/Benzothiazolyl Acetamides as Antimicrobial and Antituberculosis Agents. *Med. Chem. Res.* **2013**, *22* (1), 195–210. https://doi.org/10.1007/s00044-012-0026-x.

(3) Hirose, W.; Sato, K.; Matsuda, A. Fluorescence Properties of 5-(5,6-Dimethoxybenzothiazol-2-Yl)-2′-Deoxyuridine (DbtU) and Oligodeoxyribonucleotides Containing d BtU. *European J. Org. Chem.* **2011**, *2011* (31), 6206–6217. https://doi.org/10.1002/ejoc.201100818.

(4) Zhou, Q.; Xia, Z.; Zhang, Y.; Sun, Z.; Zeng, W.; Zhang, N.; Yuan, C.; Gong, C.; Zhou, Y.; Xue, W. Design of a Delivery Vehicle Chitosan-Based Self-Assembling: Controlled Release, High Hydrophobicity, and Safe Treatment of Plant Fungal Diseases. *J. Nanobiotechnology* **2024**, *22* (1), 1–21. https://doi.org/10.1186/s12951-024-02386-8.

(5) Potopnyk, M. A.; Volyniuk, D.; Ceborska, M.; Cmoch, P.; Hladka, I.; Danyliv, Y.; Gražulevičius, J. V. Benzo[4,5]Thiazolo[3,2-c][1,3,5,2]Oxadiazaborinines: Synthesis, Structural, and Photophysical Properties. *J. Org. Chem.* **2018**, *83* (19), 12129–12142. https://doi.org/10.1021/acs.joc.8b02098.

(6) Linciano, P.; Pozzi, C.; Iacono, L. Dello; Di Pisa, F.; Landi, G.; Bonucci, A.; Gul, S.; Kuzikov, M.; Ellinger, B.; Witt, G.; Santarem, N.; Baptista, C.; Franco, C.; Moraes, C. B.; Müller, W.; Wittig, U.; Luciani, R.; Sesenna, A.; Quotadamo, A.; Ferrari, S.; Pöhner, I.; Cordeiro-Da-Silva, A.; Mangani, S.; Costantino, L.; Costi, M. P. Enhancement of Benzothiazoles as Pteridine Reductase-1 Inhibitors for the Treatment of Trypanosomatidic Infections. *J. Med. Chem.* **2019**, *62* (8), 3989–4012. https://doi.org/10.1021/acs.jmedchem.8b02021.

(7) Kousaxidis, A.; Petrou, A.; Rouvim, P.; Bodo, P.; Stefek, M.; Nicolaou, I.; Geronikaki, A. A Molecular Hybridization Approach for the Design of Selective Aldose Reductase (ALR2) Inhibitors and Exploration of Their Activities against Protein Tyrosine Phosphatase 1B (PTP1B). *J. Mol. Struct.* **2023**, *1271*, 134116. https://doi.org/10.1016/j.molstruc.2022.134116.

(8) McPhillie, M. J.; Trowbridge, R.; Mariner, K. R.; O'Neill, A. J.; Johnson, A. P.; Chopra, I.; Fishwick, C. W. G. Structure-Based Ligand Design of Novel Bacterial RNA Polymerase Inhibitors. *ACS Med. Chem. Lett.* **2011**, *2* (10), 729–734. https://doi.org/10.1021/ml200087m.

(9) Kokot, M.; Weiss, M.; Zdovc, I.; Anderluh, M.; Hrast, M.; Minovski, N. Diminishing HERG Inhibitory Activity of Aminopiperidine-Naphthyridine Linked NBTI Antibacterials by Structural and Physicochemical Optimizations. *Bioorg. Chem.* **2022**, *128*. https://doi.org/10.1016/j.bioorg.2022.106087.

(10) Bebernitz, G. R.; Beaulieu, V.; Dale, B. A.; Deacon, R.; Duttaroy, A.; Gao, J.; Grondine, M. S.; Gupta, R. C.; Kakmak, M.; Kavana, M.; Kirman, L. C.; Liang, J.; Maniara, W. M.; Munshi, S.; Nadkarni, S. S.; Schuster, H. F.; Stams, T.; St. Denny, I.; Taslimi, P. M.; Vash, B.; Caplan, S. L. Investigation of Functionally Liver Selective Glucokinase Activators for the Treatment of Type 2 Diabetes. *J. Med. Chem.* **2009**, *52* (19), 6142–6152. https://doi.org/10.1021/jm900839k.

(11) Balijapalli, U.; Udayadasan, S.; Panyam Muralidharan, V.; Sukumarapillai, D. K.; Shanmugam, E.; Paduthapillai Gopal, A.; Rathore, R. S.; Kulathu Iyer, S. An Insight into the Photophysical Properties of Amide Hydrogen Bonded N-(Benzo[d]Thiazol-2-Yl) Acetamide Crystals. *Spectrochim. Acta - Part A Mol. Biomol. Spectrosc.* **2017**, *173*, 572–577. https://doi.org/10.1016/j.saa.2016.10.007.

(12) Jaryal, R.; Khullar, S.; Kumar, R. Benzothiazole-Derived Covalent Organic Framework for Multimedia Iodine Uptake. *J. Clust. Sci.* **2024**, *35* (2), 461–479. https://doi.org/10.1007/s10876-023-02495-8.

(13) Fois, B.; Skok, Ž.; Tomašič, T.; Ilaš, J.; Zidar, N.; Zega, A.; Peterlin Mašič, L.; Szili, P.; Draskovits, G.; Nyerges, Á.; Pál, C.; Kikelj, D. Dual Escherichia Coli DNA Gyrase A and B Inhibitors with Antibacterial

Activity. *ChemMedChem* **2020**, *15* (3), 265–269. https://doi.org/10.1002/cmdc.201900607.

(14) Panchaud, P.; Bruyère, T.; Blumstein, A. C.; Bur, D.; Chambovey, A.; Ertel, E. A.; Gude, M.; Hubschwerlen, C.; Jacob, L.; Kimmerlin, T.; Pfeifer, T.; Prade, L.; Seiler, P.; Ritz, D.; Rueedi, G. Discovery and Optimization of Isoquinoline Ethyl Ureas as Antibacterial Agents. *J. Med. Chem.* **2017**, *60* (9), 3755–3775. https://doi.org/10.1021/acs.jmedchem.6b01834.

(15) Drumm, J. E.; Deininger, D. D.; LeTiran, A.; Wang, T.; Grillot, A. L.; Liao, Y.; Ronkin, S. M.; Stamos, D. P.; Tang, Q.; Tian, S. K.; Oliver-Shaffer, P. Facile Preparation of Fused Ring Azolylureas. *Tetrahedron Lett.* **2007**, *48* (31), 5535–5538. https://doi.org/10.1016/j.tetlet.2007.05.159.

(16) Srivastava, P. K.; Srivastava, P. N. Synthesis of Some Local Anesthetics. *Journal of Medicinal Chemistry*. American Chemical Society 1970, pp 977–979. https://doi.org/10.1021/jm00299a045.

(17) Al-Janabi, A. S.; Al-Jumaili, W. A.; Al-Hayaly, L. J.; Al-Jibori, S. A.; Schmidt, H.; Wagner, C.; Hogarth, G. Synthesis and in Vitro Cytotoxicity Studies of Pd(II) and Pt(II) Acetamide Complexes: Molecular Structures of Trans-[PdCl2(Bzmta)2].DMF (Bzmta = 2-Acetylamino-6-Methylbenzothiazole) and Cis-[PtCl2(Bzta)2].2DMF (Bzta = 2-Acetylaminobenzothiazole). *Polyhedron* **2020**, *185*, 114591. https://doi.org/10.1016/j.poly.2020.114591.
